# Synthesis and in vitro cytotoxicity of acetylated 3-fluoro, 4-fluoro and 3,4-difluoro analogs of D-glucosamine and D-galactosamine

**DOI:** 10.3762/bjoc.12.75

**Published:** 2016-04-20

**Authors:** Štěpán Horník, Lucie Červenková Šťastná, Petra Cuřínová, Jan Sýkora, Kateřina Káňová, Roman Hrstka, Ivana Císařová, Martin Dračínský, Jindřich Karban

**Affiliations:** 1 Institute of Chemical Process Fundamentals of the CAS, Rozvojová 135, 165 02 Praha, Czech Republic; 2 Regional Centre for Applied and Molecular Oncology, Masaryk Memorial Cancer Institute, Žlutý kopec 7, 656 53 Brno, Czech Republic; 3 Department of Inorganic Chemistry, Charles University, Hlavova 2030, 128 43 Praha 2, Czech Republic; 4 Institute of Organic Chemistry and Biochemistry, Flemingovo nám. 2, 166 10 Praha 6, Czech Republic

**Keywords:** amino sugars, cytotoxicity, fluorinated carbohydrates, fluorine, hexosamines

## Abstract

**Background:** Derivatives of D-glucosamine and D-galactosamine represent an important family of the cell surface glycan components and their fluorinated analogs found use as metabolic inhibitors of complex glycan biosynthesis, or as probes for the study of protein–carbohydrate interactions. This work is focused on the synthesis of acetylated 3-deoxy-3-fluoro, 4-deoxy-4-fluoro and 3,4-dideoxy-3,4-difluoro analogs of D-glucosamine and D-galactosamine via 1,6-anhydrohexopyranose chemistry. Moreover, the cytotoxicity of the target compounds towards selected cancer cells is determined.

**Results:** Introduction of fluorine at C-3 was achieved by the reaction of 1,6-anhydro-2-azido-2-deoxy-4-*O*-benzyl-β-D-glucopyranose or its 4-fluoro analog with DAST. The retention of configuration in this reaction is discussed. Fluorine at C-4 was installed by the reaction of 1,6:2,3-dianhydro-β-D-talopyranose with DAST, or by fluoridolysis of 1,6:3,4-dianhydro-2-azido-β-D-galactopyranose with KHF_2_. The amino group was introduced and masked as an azide in the synthesis. The 1-*O*-deacetylated 3-fluoro and 4-fluoro analogs of acetylated D-galactosamine inhibited proliferation of the human prostate cancer cell line PC-3 more than cisplatin and 5-fluorouracil (IC_50_ 28 ± 3 μM and 54 ± 5 μM, respectively).

**Conclusion:** A complete series of acetylated 3-fluoro, 4-fluoro and 3,4-difluoro analogs of D-glucosamine and D-galactosamine is now accessible by 1,6-anhydrohexopyranose chemistry. Intermediate fluorinated 1,6-anhydro-2-azido-hexopyranoses have potential as synthons in oligosaccharide assembly.

## Introduction

**Figure 1:**
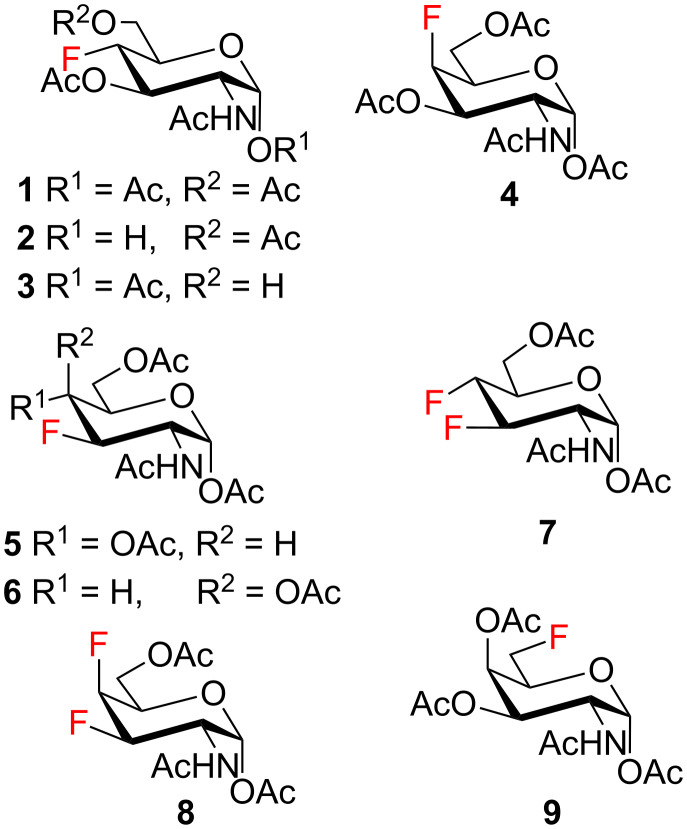
Examples of deoxofluorinated hexosamines.

Derivatives of D-glucosamine (GlcN) and D-galactosamine (GalN) are essential amino sugar components of glycans in glycoproteins, glycolipids and proteoglycans. As such, they participate in functions performed by cell-surface glycans including cell adhesion and signaling [1]. Unnatural analogs of these amino sugars prepared by a selective replacement of a hydroxy group by fluorine have proved valuable tools to perturb glycan and glycosaminoglycan biosynthesis [2–3], to inhibit amino sugars processing enzymes [4–5], to probe interactions of amino sugars with their target enzymes and lectins [6–7], or to increase the hydrolytic stability of the glycosidic bond in amino sugar glycosides [8–9]. For example, acetylated 4-fluoro analogs of D-glucosamines **1**–**3**, D-galactosamine **4**, and 6-fluoro-D-galactosamine **9** (Figure 1) acted as metabolic inhibitors of the biosynthesis of cell-surface O- and N-linked glycans (including their lactosamine and sialyl Lewis^X^ terminal epitopes) [2–3,10–11], and glycosaminoglycans [3]. The resulting disruption of protein–(glycosamino)glycan interactions had important biomedical consequences such as reduced selectin-mediated tumor cell adhesion [12–13], suppressed selectin-mediated leukocyte migration [11,14–15], reduced angiogenesis [3], or inhibition of tumor growth by decreased galectin-mediated antitumor T cell apoptosis [16].

Some acetylated fluoro hexosamines also displayed antiproliferative properties in vitro [17]. For example, 3- and 4-fluoro-D-glucosamine analogs **5** [18] and **1** [19], and 4-fluoro-D-galactosamine analog **4** [19] (Figure 1), inhibited the growth of murine L1210 leukemia cells in micromolar range (IC_50_ 27–35 μM). Compound **5** also inhibited the proliferation of the human pancreatic cancer cell line KP1-NL (IC_50_ 30 μM) [20] and 4-fluoro-D-glucosamine analogs **1** and **2** were reported to inhibit the proliferation of the human prostate cancer cell line PC-3 (IC_50_ 61 µm for **2**) [2].

**Scheme 1:**
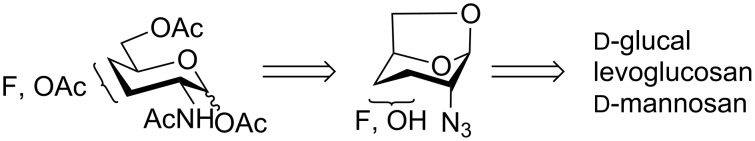
Retrosynthetic plan.

The remarkable ability of acetylated fluoro analogs of GlcNAc and GalNAc to perturb the (glycosamino)glycan biosynthesis and their antiproliferative properties aroused our interest in developing a methodology for the preparation of a complete series of acetylated 3-fluoro, 4-fluoro-, and 3,4-difluoro analogs **1**, **4**–**8** (Figure 1) including previously unknown members of this class of hexosamine mimics: acetylated 3-fluoro-D-GalNAc **6**, 3,4-difluoro-D-GlcNAc **7** and 3,4-difluoro-D-GalNAc **8**, as well as 3-fluoro-D-GlcNAc **5**, in which case the reported synthesis was troublesome and low-yielding [20]. To carry out the synthesis, flexible synthetic methods for the stereoselective introduction of fluorine at one or more designated positions of the hexosamine skeleton are necessary. The elaborated chemistry of 1,6-anhydrohexopyranose derivatives is suitable for this purpose [21–23]. Building on previous results from our [24] and other groups [25–28], we designed an approach based on stereoselective introduction of an azide as a masked amine group at C-2, and fluorine at C-3 and C-4 by nucleophilic displacement. Resulting 3-fluoro, 4-fluoro, and 3,4-difluoro analogs of 2-azido-1,6-anhydrohexopyranoses were then converted into the target fluoro analogs of D-glucosamine and D-galactosamine (Scheme 1). Dual protection of the anomeric and primary hydroxy groups in the form of the 1,6-anhydro bridge reduced the number of protecting groups, and the rigid bicyclic skeleton of 1,6-anhydrohexopyranoses enabled a high degree of regio- and stereocontrol necessary for the introduction of heteroatomic substituents at C-2, C-3, and C-4. The synthesis of the analogs **6**–**8** has not yet been reported to our knowledge, while the synthesis of **1**, **4** and **5** represents an alternative to the published procedures [2,20,29]. Herein we also report on the cytotoxicity of prepared fluoro analogs in the human ovarian cancer A2780 and prostate cancer PC-3 cell lines. Preliminary results for the synthesis of compounds **5** and **6** were communicated earlier in a letter [30].

## Results and Discussion

### Synthesis

**Scheme 2:**
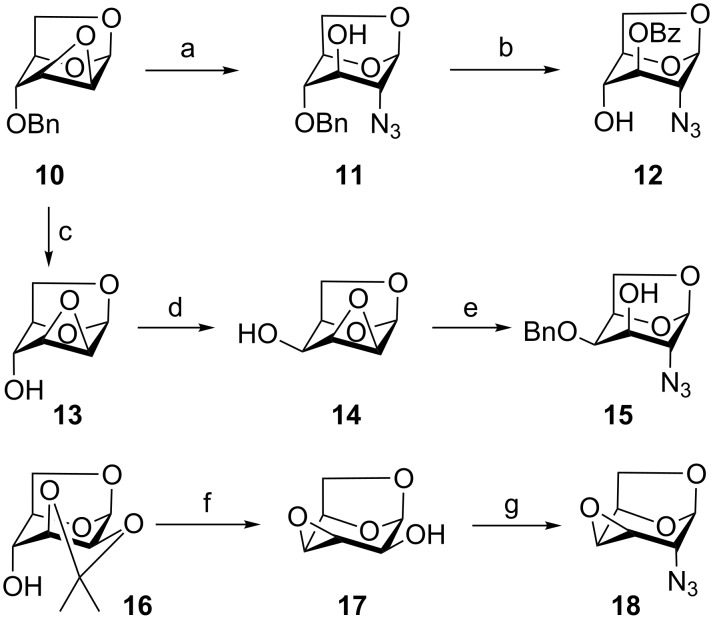
Preparation of starting 2-azido compounds. Reagents and conditions: (a) NaN_3_, NH_4_Cl, MeOC_2_H_4_OH, 79%; (b) i) BzCl, py; ii) NaBrO_3_, Na_2_S_2_O_4_, AcOEt, H_2_O, 47% over 2 steps; (c) Pd/C, H_2_, EtOH, 96%; (d) Tf_2_O, then (Bu)_4_NNO_2_, 69%; (e) (i) NaH, BnBr, THF, 79%; (ii) LiN_3_, NH_4_Cl, MeOC_2_H_4_OH, 100 °C, 81%; (f) (i) TsCl, py, (ii) AcOH, H_2_O; (iii) IRA 410 (OH^−^), MeOH, 78% over 3 steps; (g) (i) Tf_2_O, py, CH_2_Cl_2_; (ii) LiN_3_, DMF, 82% over 2 steps.

The synthesis commenced with the introduction of an azide at the 2α-position of the 1,6-anhydropyranose skeleton by nucleophilic cleavage of a 2β,3β-epoxide or the displacement of the C-2β triflate ester using mainly a combination of published procedures (Scheme 2). 2-Azido alcohols **11**, **12** and **15** were obtained from 1,6:2,3-dianhydro-4-*O*-benzyl-β-D-mannopyranose (**10**, one of the Černý epoxides available from D-glucal [31–33] or levoglucosan [34]). Regioselective azidolysis [21,35–36] of **10** yielded 2-azido alcohol **11** which was converted to **12** by benzoylation at O-3 and debenzylation at O-4. To prevent azide reduction, oxidative debenzylation [24] was applied instead of the more common hydrogenation. Debenzylation of **10** gave dianhydro derivative **13** [34,37] (available also directly from D-glucal [31,33] or from levoglucosan [38]) which was converted to **14** by Latrell–Dax inversion at C-4 [39]. *O*-Benzylation [40] of **14** followed by azidolysis [41] furnished **15**. 2-Azido-3,4-epoxide **18** was prepared from readily available [42] 2,3-isopropylidene-D-mannosan (**16**) in five steps (Scheme 2). Tosylation of **16** [43], followed by hydrolysis of the benzylidene acetal [44] and oxirane ring closure [45] at C-4 delivered 1,6:3,4-dianhydro derivative **17**. Formation of the triflate ester and azidolysis furnished 1,6:3,4-dianhydro-2-azido derivative **18**. Lithium azide was found superior to sodium azide in conversion of the triflate to the azide **18**, and the yield of **18** increased from 48% reported earlier [46] to 82%.

**Scheme 3:**
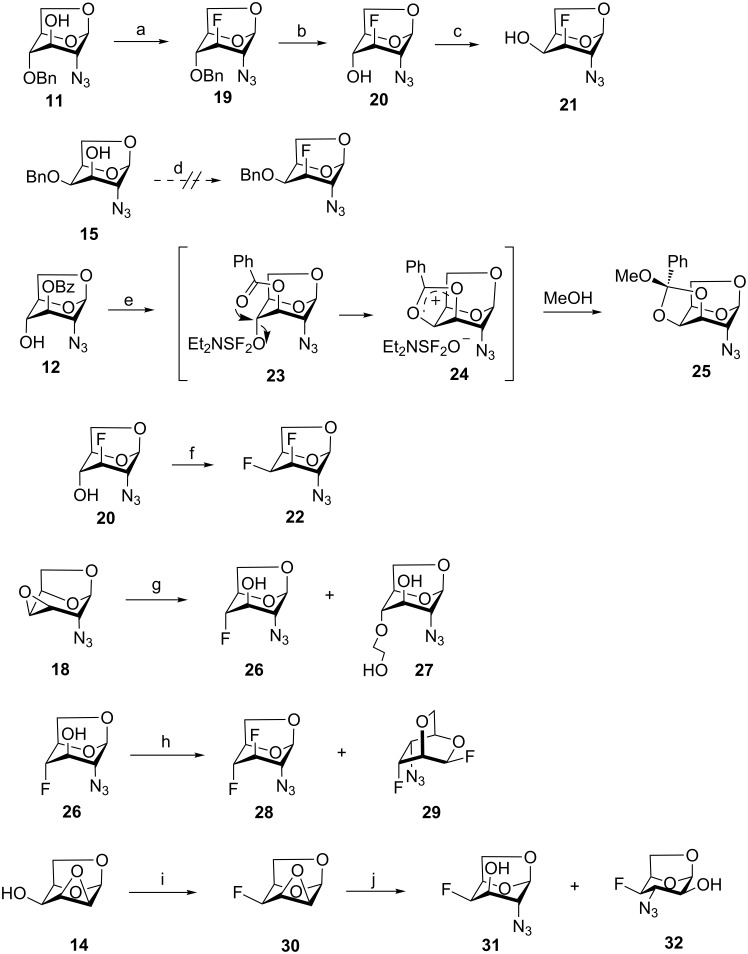
Preparation of mono and difluoro analogs of 2-azido-2-deoxy-1,6-anhydro-β-D-gluco- and galactopyranoses. Reagents and conditions: (a) DAST, benzene, 75–81 °C, 88%; (b) NaBrO_3_, Na_2_S_2_O_4_, EtOAc, H_2_O, 75%; (c) Tf_2_O, py, CH_2_Cl_2_; then (Bu)_4_NNO_2_, DMF, 67%; (d) DAST, benzene, 75–81 °C; or DAST, CH_2_Cl_2_, −25 °C to rt; (e) DAST, CH_2_Cl_2_, −25 °C to rt, 78%; (f) DAST, CH_2_Cl_2_, −25 °C to rt, 65%; (g) KHF_2_, C_2_H_4_(OH)_2_, 175 °C, 40–60% (**26**); (h) DAST, benzene, 75–81 °C, 46% (**28**), 12% (**29**); (i) DAST, CH_2_Cl_2_, −50 °C to rt, 52 %, (j) LiN_3_, NH_4_Cl, MeOC_2_H_4_OH, 100 °C, 74% (**31**).

The synthesis of mono- and difluoro analogs of 2-azido-2-deoxy-1,6-anhydrohexopyranoses, which are key intermediates, is shown in Scheme 3. We first explored the reactions of azido alcohols **11**, **12** and **15** (Scheme 3) with diethylaminosulfur trifluoride (DAST) to achieve the introduction of a nucleophilic fluorine atom. Reaction of **11** with DAST using a minor modification of the reported procedure [28] provided the D-*gluco*-configured 3-fluoro-derivative **19** (Scheme 3) with clean retention of configuration. To prepare the D-*galacto*-configured analog of **19**, deoxofluorination of **15** by reaction with DAST in benzene at 75 °C (conditions used for fluorination of **11**), or in dichloromethane at rt was attempted. Disappointingly, only unreacted **15** was recovered. A very slow formation of several unidentified fluorine-containing compounds (^19^F NMR) was observed on prolonged reaction times. Oxidative debenzylation of **19** gave fluorhydrin **20** which was subjected to epimerization at C-4 by Latrell–Dax inversion to furnish 2-azido-3-fluoro compound **21** with the desired D-*galacto* configuration (Scheme 3). The geminal fluorine–carbon coupling value ^2^*J*_C4,F_ = 17.7 Hz in **21** is characteristic for a gauche relationship between the C3–F and C4–O bonds whereas ^2^*J*_C4,F_ = 31.5 Hz in **20** is consistent with an antiperiplanar arrangement. The inversion at C-4 is also manifested by an increase in the ^3^*J*_H4,H3/5_ values (1.7 → 4.5 Hz) together with an increase of the ^3^*J*_F,H4_ coupling (13.9 → 26.4 Hz).

An attempt to introduce fluorine at C-4 on reaction of **12** with DAST furnished orthoester **25** instead of the desired 4-fluoro derivative. The structure of orthoester **25** was confirmed by single crystal X-ray analysis. The formation of **25** resulted from an intramolecular displacement of the unstable alkoxysulfur intermediate **23** with the oxygen of the benzoyl group and a subsequent reaction of the salt **24** with methanol upon quenching. A similar participation of a vicinal *O*-acetyl protecting group on reaction with DAST leading to the formation of an orthoacetate was reported [47]. Reaction of fluorohydrin **20** with DAST proceeded with inversion at C-4 giving 3,4-difluoro analog **22**. The D-*galacto* configuration of **22** was manifested by the values of the vicinal coupling constants ^3^*J*_H2,H3_ = 1.6 Hz and ^3^*J*_H3,H4_ = 4.5 Hz, and the large value of ^2^*J*_C5,F4_ = 27.2 Hz confirmed the equatorial position of fluorine at C-4.

Cleavage of the oxirane ring in **18** on reaction with KHF_2_ in ethylene glycol [48–49] furnished the 4-fluoro derivative **26** (Scheme 3). The moderate yield of **26** (40–60%) can be attributed to the formation of the 4-*O*-(2-hydroxyethyl) derivative **27** arising from solvent participation, and to a partial decomposition (indicated by TLC) at high temperatures necessary for the reaction to proceed. The low values of the vicinal coupling constant ^3^*J*_H3,H4_ = 2.0 Hz and the large value of the geminal coupling constant ^2^*J*_C3,F_ = 29.6 Hz evidenced a *trans*-diaxial relationship between the C-3 and C-4 substituents in **26**. The reaction of fluorohydrin **26** with DAST in benzene under heating afforded, as the main product, 3,4-difluoro-D-*gluco* analog **28** (46%), and the rearranged 2,6-anhydro compound **29** (12%) as a side product. Products **28** and **29** can be separated by careful chromatography and their structures were verified by single crystal X-ray analysis.

Since fluorination of **12** with DAST failed to introduce fluorine at C-4β due to formation of orthoester **25**, the reaction of 1,6:2,3-dianhydro-β-D-talopyranose **14** with DAST was utilized to give 1,6:2,3-dianhydro-4-deoxy-4-fluoro-β-D-talopyranose (**30**) with retention of the configuration at C-4 [39]. Azidolysis of the oxirane ring in the reaction with lithium azide furnished 2-azido derivative **31**. Although nucleophilic cleavage of a three-membered ring annulated to the 1,6-anhydrohexopyranose skeleton usually occurs solely in *trans*-diaxial fashion [50], formation of the *trans*-diequatorial side-product **32** in ca. 8% was observed in NMR spectra. The desired D-*galacto*-configured 2-azido derivative **31** was separated from **32** by crystallization in 74% yield. The D-*galacto* configuration of **31** is evidenced by the vicinal coupling values ^3^*J*_H4,H3/5_ = 4.5 Hz and the long range coupling between equatorial protons H-1, H-3, and H-5 (^4^*J*_H3,H1/5_ = 1.4 Hz).

**Scheme 4:**
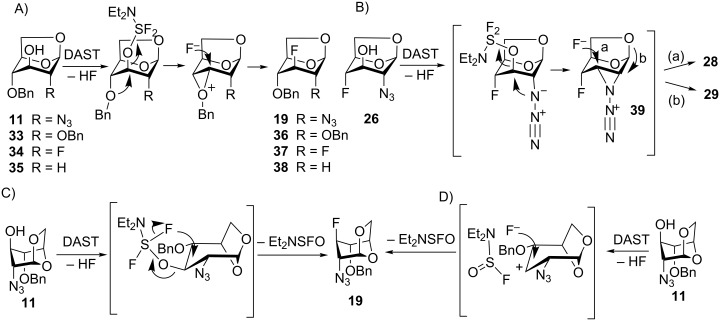
Suggested mechanisms for deoxofluorination at C-3 of 1,6-anhydro-β-D-glucohexopyranose derivatives. A) participation of the *O*-benzyl group; B) participation of the azide group; C) internal fluorine attack, concerted mechanism; D) internal fluorine attack, contact ion pair mechanism.

The reaction of the D-*gluco-*configured 3-hydroxy derivatives **11**, and **26** with DAST, and the previously reported reactions of D-*gluco*-configured 3-hydroxy derivatives **33** [26], **34** [27], and **35** [25] (Scheme 4) with DAST are an important means of obtaining 3-deoxy-3-fluoro derivatives of D-*gluco*-configured aldohexopyranoses which are difficult to prepare otherwise. These reactions characteristically proceed with a clean retention of configuration which can be explained by an anchimeric assistance of the *trans*-diaxially positioned (with respect to C3–OH) polar groups at C-2 or C-4, or by an internal fluorine attack as in S_N_i substitution. A simple S_N_2 displacement leading to configurational inversion is probably suppressed by the steric effects of the axially positioned groups at C-2 and C-4, and repulsive effects of their aligned dipoles [51]. Compounds **11**, and **33**–**35**, possess a *trans*-diaxially positioned benzyloxy group at C-4 capable of participation through an oxiranium intermediate species (Scheme 4A) [52–54]. The formation of the rearranged difluoride **29** from alcohol **26** (Scheme 3) suggests an anchimeric assistance of the vicinal C-2 azido group. Although rare, azide participation was postulated before [52]. The main product **28** and rearranged difluoride **29** can arise from the same intermediate species **39** (Scheme 4B, pathways a and b, respectively). Compound **19** can, in principle, be also formed from **11** through azide participation (not shown). In such a case the reaction is unexpectedly sensitive to minor steric alterations of the substrate because the C-4 epimer **15** (Scheme 3) did not react. An internal fluorine attack from the β-face of the tetrahydropyran ring through a concerted (Scheme 4C) or contact ion-pair (Scheme 4D) S_N_i mechanism cannot be ruled out [55–56] because the bulky Et_2_NSF_2_O substituent at C-3 might force the substrate to adopt boat *B*_3,O_ conformation [57] bringing the C-2, C-3 and C-4 substituents into a *trans*-equatorial arrangement unfavorable for anchimeric assistance.

**Scheme 5:**
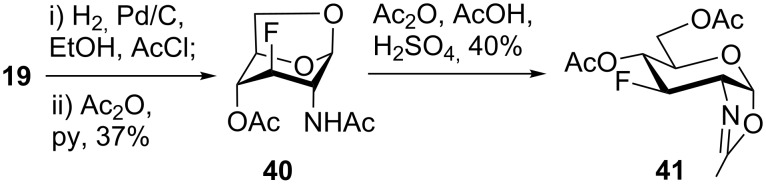
Formation of oxazoline **41** from **19**.

With the D-*gluco*- and D-*galacto*-configured deoxofluoro derivatives of 2-azido-1,6-anhydrohexopyranoses in hand, we examined their conversion to the target acetylated hexosamine analogs. Initially, compound **19** was hydrogenated (Pd/C) and acetylated to afford acetamide **40** in modest yield (37%). Sulfuric acid-catalyzed [35] cleavage of the internal acetal with acetic anhydride gave a mixture containing 1,2-oxazoline **41** as the main product (Scheme 5). The structure of oxazoline **41** was confirmed by single crystal X-ray diffraction analysis which also confirmed the retention of configuration during the preceding fluorine introduction.

To prevent oxazoline formation, the order of reactions was reversed, and triethylsilyl triflate (TESOTf)-catalyzed [58] acetolysis of the 1,6-anhydro bridge in **19** gave **42** (Table 1) as a mixture of anomers from which the α-anomer crystallized. TESOTf as a catalyst for acetolysis gave better results than sulfuric acid in terms of product purity. Hydrogenolysis of **42** on palladium in ethanol/HCl followed by acetylation of the amino group furnished the target acetylated 3-fluoro-D-GlcNAc **5** as a chromatographically separable mixture of anomers (Table 1). Addition of HCl was found necessary to effect a clean hydrogenolytic removal of the *O*-benzyl group in **42**. The D-*gluco* configuration of **42** and **5** was reflected in the large values of ^3^*J*_H3,H2/4_ (7.6–10.2 Hz), and the chair inversion accompanying the cleavage of the 1,6-anhydro bridge (^1^*C*_4_ → ^4^*C*_1_) in a decrease of the geminal fluorine–carbon coupling value ^2^*J*_C4,F_ (31.5 Hz in **40** → 18.4 Hz in **5** (α-anomer)) [59]. ^1^H and ^13^C NMR spectra of **5** reported in [20] are comparable with our data for the α-anomer (**5α**). A discrepancy in the values of specific optical rotation 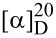 (reported +10°, +81° obtained by us) can be explained assuming that the value for the β-anomer (**5β**) was reported in [20]. Our synthesis of **5** is more practical (55% from **10** in 3 steps) than the earlier preparations which were reported troublesome owing to low yields (28% and 10%, respectively) of the key fluorination step [18,20].

**Table 1:** Acetolysis of the fluorine-containing intermediates and hydrogenation.

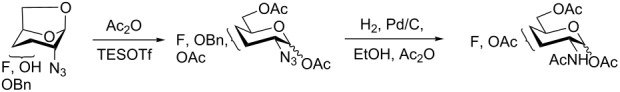					

entry	starting compound	product of acetolysis	yield (%)^a^	target compound	yield (%)^a^

1	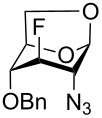 **19**	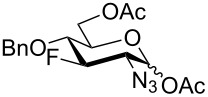 **42**	96	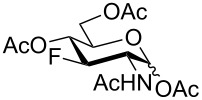 **5**	82^b^
2	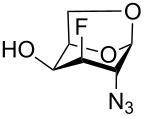 **21**	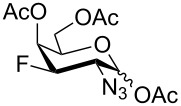 **43**	83	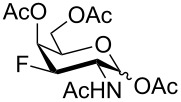 **6**	74
3	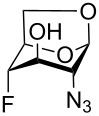 **26**	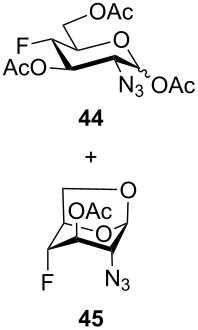	78	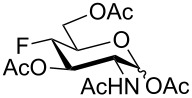 **1**	86
			16	n.a.	
4	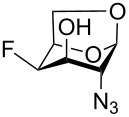 **31**	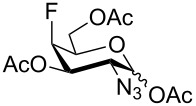 **46**	94	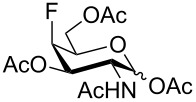 **4**	78
5	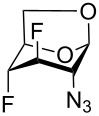 **28**	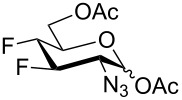 **47**	^c^	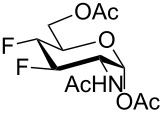 **7**	44^d^
6	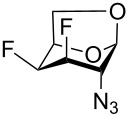 **22**	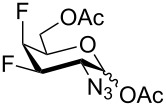 **48**	86	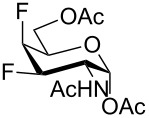 **8**	70

^a^Isolated yield; ^b^H_2_, Pd/C, EtOH, AcCl, then Ac_2_O, py was used for hydrogenation of **42**; ^c^**47** was not isolated as pure compound, see text; ^d^overall from **28**; n.a. – not available.

For the remaining fluoro derivatives of 1,6-anhydro-2-azidohexopyranoses, TESOTf-catalyzed acetolysis and subsequent hydrogenation (Pd/C in a mixture of ethanol and acetic anhydride) was applied to obtain the target acetylated fluoro analogs (Table 1). Compound **21** was acetolyzed to **43** which was obtained as a mixture of anomers and characterized by NMR and finally hydrogenated to furnish acetylated 3-fluoro-GalNAc **6** isolated as a separable mixture of anomers. The D-*galacto* configuration of **6** is manifested by the lower ^3^*J*_H3,H4_ coupling value (3.4 Hz, α-anomer) in comparison with that of its C-4 epimer **5** (8.6 Hz, α-anomer). Acetolysis of the internal acetal in **26** proceeded more slowly and beside the desired product **44** (78% yield), 3-*O*-acetyl derivative **45**, in which the 1,6-anhydro bridge remained intact, was also isolated. Hydrogenation of **44** furnished the known acetylated 4-fluoro-D-GlcNAc **1** [2]. The large values of the vicinal coupling constants ^3^*J*_H3,H2/4_ and ^3^*J*_H4,H5_ (8.9–11.1 Hz) confirmed the D-*gluco* configuration of **1** and the chair inversion when going from **26** to **1**, which was also manifested by a decrease in the geminal coupling constant ^2^*J*_C3,F_ (29.6 → 18.9 Hz, α-anomer). Acetolysis and subsequent hydrogenation of **31** provided the known peracetylated 4-deoxy-4-fluoro-D-galactosamine **4** [29]. The chair inversion of the tetrahydropyran ring associated with the cleavage of the 1,6-anhydro bridge is indicated by the increase in the coupling value ^3^*J*_H2,H3_ (1.4 → 11.6 Hz) and decrease in the coupling value ^2^*J*_C5,F_ (27.4 → 18.4 Hz) when going from **31** to **4** (α-anomer). Acetolysis of **28** afforded diacetate **47** containing chromatographically inseparable impurities (NMR). They were removed in the next hydrogenation step to yield the peracetylated 3,4-difluoro analog of D-glucosamine **7** isolated as an α-anomer in 44% yield from **28**. The D-*gluco* configuration of **7** is reflected in the large values of the vicinal coupling constants ^3^*J*_H2,H3_ = 10.5 Hz, ^3^*J*_H3,H4_ = 8.2 Hz, and ^3^*J*_H4,H5_ = 9.8 Hz. Acetolysis of **22** gave diacetate **48** as a separable mixture of anomers. Hydrogenation of the α-anomer furnished the peracetylated 3,4-difluoro analog of D-galactosamine **8**. The D-*galacto* configuration of **8** was confirmed by an increase in the value of ^3^*J*_H2,H3_ (1.6 → 11.1 Hz), a decrease in the value of ^2^*J*_C5,F4_ (27.2 → 18.3 Hz), and an increase of ^3^*J*_H3,F4_ coupling (4.3 → 26.0 Hz) between **22** and **8**.

**Scheme 6:**
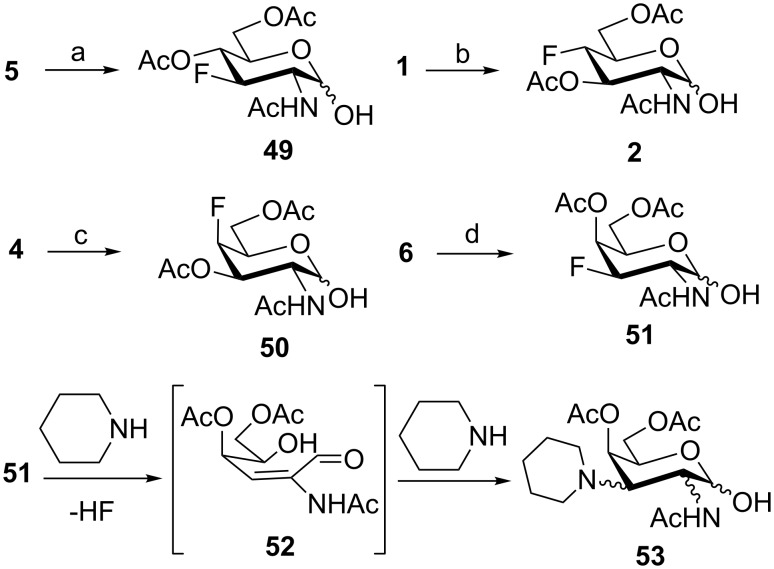
1-*O*-Deacetylation of monofluorinated hexosamines. Reagents and conditions: (a) BnNH_2_, THF, 62%; (b) C_5_H_10_NH, THF, 74%; (c) C_5_H_10_NH, THF, 60%; (d) silica gel, MeOH, 30 days, 40%.

To study the influence of 1-*O*-deacetylation on the cytotoxicity, the monofluorinated analogs **1**, and **4**–**6** were subjected to anomeric deacetylation (Scheme 6). Compound **5** provided 1-*O*-deacetylated product **49** by treatment with BnNH_2_ in THF. Since acetylated 4-fluoro-D-GlcNAc **1** under these conditions did not react cleanly, we used piperidine-promoted [60] deacetylation to prepare **2** in 74% yield. Similarly, acetylated 4-fluoro-D-GalNAc **4** gave **50** in 60% yield. The attempted anomeric deacetylation of 3-fluoro-D-GalNAc **6** by treatment with piperidine followed by chromatography gave a fraction containing an inseparable side-product in addition to the expected deacetylated product **51**. The side-product showed no fluorine resonance in ^19^F NMR and its molecular formula C_17_H_28_N_2_O_7_ assigned by LC–HRMS corresponded to a formal displacement of fluorine by piperidine, leading probably to compound **53**. When pure **51** (prepared by another method, see below) was reacted with excess piperidine, high resolution ESIMS analysis of the reaction detected transient formation of an adduct ion corresponding to a supposed intermediate enal **52** (Scheme 6), while the adduct ion corresponding to **53** was the final product (see Supporting Information File 1). Presumably, piperidine as a relatively strong base effected dehydrofluorination of **51** to enal **52** which then added piperidine to give **53** as a byproduct (Scheme 6). To avoid the action of basic amines, a silica gel mediated anomeric deacetylation, recommended for 2-aminosugars [61], was tried. The reaction proceeded extremely slowly with our substrate **6** and the product **51** was obtained in only 40% yield after chromatography and recrystallization.

### Cytotoxicity

Some acetylated fluorinated hexosamines (HexN), including peracetates of the α-methyl glycoside of 3-fluoro-D-ManNAc [18], 3-fluoro-D-GlcNAc **5** [18], 4-fluoro-D-GlcNAc **1** [19], 4-fluoro-D-GalNAc **4** [19], 4,4-difluoro-D-*xylo*-HexNAc [19], and 4,6-difluoro-D-GalNAc [19] were reported to exhibit antiproliferative properties against L1210 leukemia cells in micromolar concentrations (IC_50_ 24–43 μM). It was found that *O*-deacetylated amino sugars were often inactive due to low lipophilicity and poor cellular uptake [19]. Compound **5** was also cytotoxic to the human pancreatic cancer cell line KP1-NL (IC_50_ = 30 μM) [20], and **1** and its 1-*O*-deacetylated derivative **2** inhibited cell proliferation of the human prostate cancer cell line PC-3 (IC_50_ 61 µM for **2**) [2]. Interestingly, while all 4-fluoro analogs **1**–**3** (Figure 1) reduced the expression of highly branched N-glycans in PC-3 cells, the 6-*O*-deacetylated analog **3** showed only negligible cytotoxicity [2] implying that the inhibition of proliferation and perturbation of N-glycan biosynthesis occur by different mechanisms.

Increased cytotoxicity as a result of 1-*O*-deacylation was noted for a variety of acylated (nonfluorinated) D-mannosamine and D-glucosamine derivatives [62–63]. Acylated hexosamine derivatives were subsequently studied as possible templates for the development of anticancer therapeutics [64–65]. While the ability of hexosamine derivatives and analogs to inhibit cell growth creates an avenue for their use in the development of anticancer drugs, it also limits their utility as agents to modify the cellular glycome [62]. The cytotoxic activity of peracetylated monofluoro analogs **1**, and **4**–**6**, their 1-*O*-deacetylated derivatives **2**, and **49**–**51**, difluoro analogs **7** and **8**, and oxazoline **41** was therefore tested for 24 h on the human prostate cancer PC-3 cell line, and human ovarian cancer A2780 cell line using the MTT assay, and the obtained IC_50_ values were compared with those obtained for cisplatin and 5-fluorouracil.

All of the tested compounds induced only moderate to weak inhibition of A2780 cell proliferation (IC_50_ values ranging from 78 μM to 327 μM, Table 2) in comparison to cisplatin (IC_50_ 12.9 μM). Anomeric deacetylation resulted in a higher activity in the case of 3-fluoro-D-GlcNAc (**49**, IC_50_ 84 μM, Table 2, entry 6), and especially 4-fluoro-D-GlcNAc (**2**, IC_50_ 78 μM, ca. 4-fold higher activity than that of the peracetylated **1**, Table 2, entries 1 and 2), while 1-*O*-deacetylated **50** had a lower activity (IC_50_ 142 μM, Table 2, entry 4) then the parent peracetate **4** (IC_50_ 78 μM, Table 2, entry 2). All of the tested fluorinated D-glucosamine analogs also induced only weak inhibition on PC-3 cells (IC_50_ ranging from 134 μM to 337 μM, Table 2, entries 1, 2, 5, 6, and 9), and 1-*O*-deacetylation seemed to have a negative effect here (Table 2, entries 2 and 6). On the other hand, the 1-*O*-deacetylated form of 3-fluoro-D-galactosamine **51** (IC_50_ 28 μM, Table 2, entry 8) and 4-fluoro-D-galactosamine **50** (IC_50_ 54 μM, Table 2, entry 4) reduced proliferation of PC-3 cells more than cisplatin (IC_50_ 182 μM, Table 2, entry 12) and 5-fluorouracil (IC_50_ 69 μM, Table 2, entry 13). The effect of anomeric deacetylation was particularly pronounced in 3-fluoro-D-galactosamine **51** owing to inactivity of the corresponding peracetate **6**. Finally, difluoro analogs **7** and **8**, and oxazoline **41** exhibited weak (compound **7**) to none (compounds **8** and **41**) activity in the PC-cell line (Table 2, entries 9–11). Taken together, our results seem to corroborate in part the observations by Yarema at al. that the liberation of the anomeric hydroxy group in acylated hexosamines resulted in an enhancement of their cytotoxicity [62–63]. The effect of anomeric deacetylation was, however, found much more cell-line and substrate-specific for our fluoro analogs than it was for acylated natural hexosamines [62].

**Table 2:** IC_50_ mean values (μM) of selected fluorinated analogs after 24 h treatment using PC-3 and A2780 cancer cell lines.

entry	compd. no.	abbreviation^a^	PC-3	A2780

1	**1**	4F-Ac_3_GlcNAc	235 ± 26	327 ± 48
2	**2**	4F-Ac_2_GlcNAc-1-OH	337 ± 36	78 ± 9
3	**4**	4F-Ac_3_GalNAc	101 ± 15	78 ± 9
4	**50**	4F-Ac_2_GalNAc-1-OH	54 ± 5	142 ± 28
5	**5**	3F-Ac_3_GlcNAc	134 ± 17	218 ± 29
6	**49**	3F-Ac_2_GlcNAc-1-OH	308 ± 38	84 ± 6
7	**6**	3F-Ac_3_GalNAc	>500	249 ± 36
8	**51**	3F-Ac_2_GalNAc-1-OH	28 ± 3	137 ± 24
9	**7**	3F,4F-Ac_2_GlcNAc	199 ± 34	183 ± 26
10	**8**	3F,4F-Ac_2_GalNAc	>500	250 ± 41
11	**41**	3F-Ac_2_Glc-1,2-oxazoline	>1000	108 ± 14
12	cisplatin		182 ± 13	12.9 ± 1.5
13	5-fluorouracil		69 ± 6	52 ± 7

^a^These abbreviations are provided for quick orientation, anomeric deacetylation is indicated by '-1-OH'.

## Conclusion

We have developed a synthetic route for the preparation of a series of 3- and 4-deoxofluorinated analogs of D-glucosamine and D-galactosamine. The choice of O-benzylated-1,6:2,3-dianhydro-β-D-mannopyranose **10** and 2,3-isopropylidene-D-mannosan **16** as starting material permits regio- and stereoselective introduction of both fluorine and nitrogen (as an azide) by nucleophilic substitution before acetolysis of the 1,6-anhydro bridge. The characteristic feature of the synthesis is the introduction of fluorine at C-3 by the reaction of D-*gluco*-configured 3-hydroxy derivatives with DAST with retention of configuration. The 1-*O*-deacetylated 3-fluoro and 4-fluoro analogs **51** and **50** of acetylated D-galactosamine were shown to be more cytotoxic in the PC-3 cell line than cisplatin and 5-fluorouracil. Most of the other fluoro analogs displayed moderate to low cytotoxicity. Fluoro analogs **6**–**8** are new compounds and their influence on the cell-surface glycan biosynthesis is currently being studied. We anticipate that 1,6-anhydro-2-azido-fluorohydrins **20**, **21**, **26** and **31** will find use as building blocks for the synthesis of fluorinated oligosaccharides and other glycoconjugates because they can be immediately employed as glycosyl acceptors or readily converted into glycosyl donors. Research in this way is now in progress in our laboratory.

## Supporting Information

File 1 Experimental procedures for compounds **1**, **2**, **4**–**8**, **12**, **18**–**22**, **25**, **26**, **28**, **29**, **31**, **40**–**42**, **44**, **45**, and **48**–**51**, HRMS results for reaction of **51** with piperidine, cell culture conditions and MTT assay, crystallographic data for compounds **25**, **28**, **29**, and **41**, and dose-response curves.

File 2 NMR spectra for compounds **1**, **2**, **4**–**8**, **12**, **18**–**22**, **25**, **26**, **28**, **29**, **31**, **40**–**46**, and **48**–**51**.
