# Supplementary material for: Synthesis and in vitro cytotoxicity of acetylated 3-fluoro, 4-fluoro and 3,4-difluoro analogs of D-glucosamine and D-galactosamine
Source: Beilstein J Org Chem. 2016 Apr 20;12:750–9. doi: 10.3762/bjoc.12.75 (PMC4901990; doi:10.3762/bjoc.12.75)
Supplement: File 1 — Experimental procedures for compounds 1, 2, 4–8, 12, 18–22, 25, 26, 28, 29, 31, 40–42, 44, 45, and 48–51, HRMS results for reaction of 51 with piperidine, cell culture conditions and MTT assay, crystallographic data for compounds 25, 28, 29, and 41, and dose-response curves. [file Beilstein_J_Org_Chem-12-750-s001.pdf]

Supporting Information File 1

for

Synthesis and in vitro cytotoxicity of acetylated 3-fluoro, 4-fluoro and 3,4-difluoro analogs of D-glucosamine and D-galactosamine

Štěpán Horník[1], Lucie Červenková Šťastná[1], Petra Cuřínová[1], Jan Sýkora[1], Kateřina Káňová[2,] Roman Hrstka[2], Ivana Císařová[3], Martin Dračínský[4] and Jindřich Karban[*1]

Address: [1]Institute of Chemical Process Fundamentals of the CAS, Rozvojová 135, 165 02 Praha, Czech Republic, [2]Regional Centre for Applied and Molecular Oncology, Masaryk Memorial Cancer Institute, Žlutý kopec 7, 656 53 Brno, Czech Republic, [3]Department of Inorganic Chemistry, Charles University, Hlavova 2030, 128 43 Praha 2, Czech Republic and [4]Institute of Organic Chemistry and Biochemistry, Flemingovo nám. 2, 166 10 Praha 6, Czech Republic
Email: Jindřich Karban - karban@icpf.cas.cz

* Corresponding author

Experimental procedures for compounds 1, 2, 4–8, 12, 18–22, 25, 26, 28, 29, 31, 40–42, 44, 45, and 48–51, HRMS results for reaction of 51 with piperidine, cell culture conditions and MTT assay, crystallographic data for compounds 25, 28, 29, and 41, and dose-response curves.

I. Synthesis S2

II. Reaction of 3-fluoro derivative 51 with piperidine – HRMS analysis S27

III. Cell lines and culture conditions and MTT assay S30

IV. Dose-response curves S31

V. Crystallographic data S40

I. Synthesis

General methods

The ^{1}H (299.9 or 499.9 MHz), ^{13}C (75.4 or 125.7 MHz) and ^{19}F (282.2 or 470.3 MHz) NMR spectra were measured on a Varian Mercury 300 or Varian Inova 500 spectrometer at 25 °C. The ^{1}H and ^{13}C NMR spectra were referenced to the line of the solvent (δ/ppm; δ_H/δ_C : $CDCl_3$, 7.26/76.99; DMSO-d_6, 2.51/39.52; CD_3OD, 3.31/49.00). The ^{19}F NMR spectra were referenced to external $CFCl_3$. HRMS analyses were done using Bruker MicrOTOF-QIII, ESI in positive mode, under the following conditions: *m*/*z* 100–2000, capillary 4200 V, end plate −500 V, collision cell RF 350Vpp, nebulizer 1.6 bar, heater 180 °C, dry gas 8 L/min, calibration – sodium formate; APCI in positive mode, 100-2000m/z, capillary 4000V, end plate -500V, corona 3000 nA, collision cell RF 350Vpp, nebulizer 3 bar, dry heater 350 °C, dry gas 3 L/min, calibration Tuning mix APCI-TOF (pos). TLC was carried out with Merck DC Alufolien with Kiesegel F254 and spots were detected with an anisaldehyde solution in EtOH/H_2SO_4. UV detection at 254 nm was also used where appropriate. Anomeric ratios α/β were determined by NMR or GC/MS. Commonly used solvent systems were: S1 ethyl acetate, S12 ethyl acetate/petroleum ether 1:2, S14 ethyl acetate/petroleum ether 1:4. Column chromatography was performed with silica gel 60 (70–230 mesh, Merck). The solutions were concentrated using a vacuum rotary evaporator at less than 45 °C if not otherwise stated. Anhydrous sodium sulfate was used to dry solutions during workup. DAST was purchased from Acros Organics and stored at −15 °C.

General procedure for acetolysis of the 1,6-anhydro bridge

A fluoroderivative was dissolved in a specified volume of a solution of triethylsilyl triflate (TESOTf) in acetic acid anhydride prepared by addition of 2 drops of TESOTf to 5 mL of Ac_2O, and the resulting solution was stirred for a given time at rt until TLC or GC/MS indicated disappearance of the starting compound. A saturated solution of $NaHCO_3$ was added in portions at 0 °C until gas evolution ceased and the resulting mixture was extracted with ethyl acetate (3×), the combined extracts were washed with brine, dried and concentrated.

General procedure for hydrogenation of azido derivatives on Pd/C

The specified volume of acetic acid anhydride and the specified amount of 10% Pd/C were added to a solution of the starting azido derivative in ethanol. The resulting mixture was degassed under reduced pressure and flushed with hydrogen (3×) and then hydrogenated at rt for a given time until TLC indicated full conversion of the starting material. The reaction mixture was filtered and concentrated.

1,6-Anhydro-2-azido-3-*O*-benzoyl-2-deoxy-β-D-glucopyranose (12)

Benzoyl chloride (350 μL, 3.02 mmol) was added to a solution of azide **11** [1] (415 mg, 1.50 mmol) in pyridine (7 mL) and the resulting solution was stirred overnight. It was then poured onto ice and extracted with dichloromethane (3×). The extracts were combined, dried and concentrated to afford crude 3-O-benzoate (506 mg). The benzoate was dissolved in ethyl acetate (20 mL) and a solution of sodium bromate (681 mg, 4.51 mmol) in water (15 mL) was added. The resulting mixture was stirred vigourously and a solution of sodium dithionite (784 mg, 4.51 mmol) was added dropwise and stirring continued for 2.5 h. Sodium thiosulfate was then added to destroy excess of bromine, the organic layer was separated and the water layer was extracted with ethyl acetate (3×). The combined organic phases were dried and concentrated, the residue dissolved in chloroform, the solution was washed with saturated $NaHCO_3$ and water, dried and concentrated. Chromatography in S12 followed by recrystallization from hot ethyl acetate/heptane gave benzoate **12** (203 mg, 47%); mp 149–150 °C; $[\alpha]^{20}_{D}$ +33 ($CHCl_3$, 0.29); (ref [2] gives 143–145 °C; $[\alpha]^{20}_{D}$ +24 (*c* 0.9, CH_2Cl_2)); ^{1}H NMR (500 MHz, $CDCl_3$) agrees with that reported [2], additional coupling constants $^3J_{2,1}$ = 1.5 Hz, $^3J_{2,3}$ = 1.6 Hz, $^2J_{6ex,6en}$ = 7.7 Hz, $^3J_{6ex,5}$ = 5.8 Hz, $^2J_{6en,6ex}$ = 7.7 Hz, $^3J_{5,6ex}$ = 5.8 Hz, $^3J_{3,2}$ = 1.6 Hz, $^3J_{3,4}$ = 1.6 Hz, $^4J_{3,1}$ = 1.6 Hz, $^4J_{3,5}$ = 1.6 Hz, $^3J_{1,2}$ = 1.5 Hz, $^4J_{1,3}$ = 1.6 Hz; ^{13}C {^{1}H} NMR (125 MHz, $CDCl_3$) δ 59.4 (C-2), 65.2 (C-6), 68.6 (C-4), 72.2 (C-3), 76.1 (C-5), 100.0 (C-1), 128.7 (*C*H, Bz), 129.1 (C_{ipso}), 129.7, 133.7 (2 × *C*H, Bz), 165.3 (*C*O, Bz). HRMS (ESI) *m/z* $[M + Na]^+$ calcd for $C_{13}H_{13}N_3NaO_5$: 314.0747; found: 314.0752.

1,6:3,4-Dianhydro-2-azido-2-deoxy-β-D-galactopyranose (18)

Triflic anhydride (1.4 mL, 8.32 mmol) was added dropwise under cooling (−30 °C) and stirring to a solution of 1,6:3,4-dianhydro-β-D-talopyranose (**17**, 978 mg, 6.76 mmol) [3] in anhydrous dichloromethane (7 mL) and anhydrous pyridine (1 mL). The temperature was allowed to reach 0 °C under stirring in about 40 min. The reaction mixture was poured into a saturated ice cold solution of $NaHCO_3$, extracted with dichloromethane, dried and concentrated to afford crude triflate (1.750 g, 93%). The triflate was dissolved in anhydrous DMF (7 mL), lithium azide (800 mg, 16.34 mmol) was immediately added and the resulting mixture was stirred at rt for 3 d. The reaction mixture was then diluted with water, extracted with chloroform (5×), the combined extracts were dried and concentrated. Chromatography in S12 gave **18** (944 mg, 82%), $[\alpha]_D^{20}$ −88 ($CHCl_3$, 0.205), ref. [4] gives $[\alpha]^{23}_D$ −88 ($CHCl_3$, 1.0), mp 75–81 °C (ref. [4] 80–81 °C). Anal. Calcd for $C_6H_7N_3O_3$: C, 42.61; H, 4.17; N, 24.84. Found: C, 42.65; H, 4.05; N, 24.76.

1,6-Anhydro-2-azido-4-*O*-benzyl-2,3-dideoxy-3-fluoro-β-D-glucopyranose (19)

DAST (0.8 mL, 6.05 mmol) was added to a stirred mixture of **11** [1] (600 mg, 2.16 mmol) in anhydrous benzene (8 mL) at rt. The starting **11** dissolves completely on addition of DAST. The reaction was stirred and heated under argon at 75–81 °C for 30 min. TLC in S12 showed the absence of **11**. The reaction mixture was cooled (ice-water), and quenched by addition of methanol (0.5 mL). After 15 min, saturated solution of $NaHCO_3$ was added in portions until gas evolution ceased. The resulting mixture was extracted with ethyl acetate (3×) and the combined extracts were successively washed with 1 % aqueous HCl, water and brine, dried and concentrated to afford crude product (619 mg). Chromatography in S13 gave **19** as yellowish syrup (532 mg, 88%), $[\alpha]_D^{20}$ +22 ($CHCl_3$, 0.17), ref [5] gives $[\alpha]^{22}_D$ +22 ($CHCl_3$, 1.5), NMR data agree with ref. [5]. Anal. Calcd for $C_{13}H_{14}FN_3O_3$: C, 55.91; H, 5.05; N, 15.05. Found: C, 55.71; H, 4.84; N, 14.87.

1,6-Anhydro-2-azido-2,3-dideoxy-3-fluoro-β-D-glucopyranose (20)

A solution of sodium bromate (936 mg, 6.20 mmol) in water (19 mL) was added to a solution of azide **19** (574 mg, 2.06 mmol) in ethyl acetate (29 mL) and to the resulting mixture a solution of sodium dithionite (1.084 g, 6.23 mmol) in water (42 mL) was added dropwise under vigorous stirring. Stirring continued for 2 h. TLC in S12 showed absence of the starting **19**. Sodium thiosulfate was added to destroy an excess of bromine, the organic layer was separated, the water phase was extracted with ethyl acetate (3×) and the combined organic extracts washed with brine, dried and concentrated. Chromatography in freshly prepared heptane/AcOEt/ethanol/25% aq NH_3 40:45:9:2 yielded **20** [6] (292 mg, 75%), mp 102–105 °C (MeOH); $[\alpha]_D^{20}$ −6.0 (*c* 0.18, $CHCl_3$); ^{1}H NMR (300 MHz, $CDCl_3$) δ 2.59 (1 H, d, $^3J_{4,OH}$ = 10.7 Hz, OH), 3.61 (1H, dddd, $^3J_{2,1}$ = 1.9 Hz, $^3J_{2,3}$ = 1.7 Hz, $^3J_{2,F}$ = 13.9 Hz, $^4J_{2,4}$ = 1.7 Hz, H-2), 3.77 (1 H, ddddd, $^3J_{4,OH}$ = 10.7 Hz, $^3J_{4,F}$ = 13.9 Hz, $^3J_{4,5}$ = 1.8 Hz, $^3J_{4,3}$ = 1.7 Hz, $^4J_{2,4}$ = 1.7 Hz, H-4), 3.84 (1 H, ddd, $^2J_{6en,6ex}$ = 7.7 Hz, $^3J_{6ex,5}$ = 5.8 Hz, $^5J_{6ex,F}$ = 2.5 Hz, H-6*ex*), 4.11 (1 H, dd, $^2J_{6ex,6en}$ = 7.7 Hz, $^3J_{6en,5}$ = 1.3 Hz, H-6*en*), 4.59 (1H, ddd, $^3J_{6en,5}$ = 1.3 Hz, $^3J_{6ex,5}$ = 5.8 Hz, $^3J_{4,5}$ = 1.8 Hz, H-5), 4.62 (1H, ddt, $^4J_{3,1}$ = 1.9 Hz, $^3J_{2,3}$ = 1.7 Hz, $^3J_{4,3}$ = 1.7 Hz, $^2J_{3,F}$ = 44.2 Hz, H-3), 5.50 (1 H, dd, $^3J_{2,1}$ = 1.9 Hz, $^4J_{3,1}$ = 1.9 Hz, H-1); ^{13}C {^{1}H} NMR (75 MHz, CDCl3) δ 58.8 (d, $^2J_{C,F}$ = 25.4 Hz, C-2), 64.9 (d, $^4J_{C,F}$ = 4.7 Hz, C-6), 67.7 (d, $^2J_{C,F}$ = 26.6 Hz, C-4), 75.7 (s, C-5), 89.9 (d, $^1J_{C,F}$ = 185.5 Hz, C-3), 99.8 (s, C-1); ^{19}F NMR (282 MHz, CDCl3) δ −180.40 (dt, $^2J_{3,F}$ = 44.2 Hz, $^3J_{4,F}$ = 13.9 Hz, $^3J_{2,F}$ = 13.9 Hz). HRMS (ESI) *m/z* $[M + Na]^+$ calcd for $C_6H_8FN_3NaO_3$: 212.0441; found: 212.0448. Anal. Calcd for $C_6H_8FN_3O_3$: C, 38.10; H, 4.26; N, 22.22. Found: C, 38.27; H, 4.23; N, 21.84.

1,6-Anhydro-2-azido-2,3-dideoxy-3-fluoro-β-D-galactopyranose (21)

Triflic anhydride (0.28 mL, 1.60 mmol) was added under cooling (−40 °C) and stirring to a solution of **20** (141 mg, 0.71 mmol) in anhydrous dichloromethane (1.6 mL) and anhydrous pyridine (0.4 mL). The reaction was allowed to warm to 0 °C in one hour. TLC in S12 indicated complete conversion. The reaction mixture was poured onto ice and extracted with dichloromethane (4×). The combined extracts were dried and concentrated to give crude triflate (234 mg, 98%) which was dissolved in anhydrous DMF (2.5 mL), tetrabutylammonium nitrite (0.3 g, 2.07 mmol) was

immediately added and the reaction was stirred at rt for 24 h. TLC in S12 indicated a full conversion of the triflate. The reaction was concentrated to about 1/3 of its volume and the residue chromatographed in S11 to give galacto derivative **21** [6] (108 mg) contaminated by **20** (3% by GC/MS) which was removed by chromatography in chloroform/ethanol 18:1 to afford **21** for analyses (94 mg, 67%); mp 44–46 °C; $[\alpha]_D^{20}$ +47 (*c* 0.14, $CHCl_3$); ^{1}H NMR (300 MHz, $CDCl_3$) δ 2.40 (1H, dd, $^3J_{4,OH}$ = 8.8 Hz, $^4J_{F,OH}$ = 4.0 Hz, O*H*), 3.70 (1H, dt, $^3J_{2,1}$ = 1.5 Hz, $^3J_{2,3}$ = 1.5 Hz, $^3J_{2,F}$ = 14.7 Hz, H-2), 3.73 (1H, ddd, $^2J_{6en,6ex}$ = 7.9 Hz, $^3J_{6ex,5}$ = 4.5 Hz, $^5J_{6ex,F}$ = 1.2 Hz, H-6*ex*), 4.06 (1H, ddt, $^3J_{4,OH}$ = 8.8 Hz, $^3J_{4,F}$ = 26.4 Hz, $^3J_{4,5}$ = 4.5 Hz, $^3J_{4,3}$ = 4.5 Hz, H-4), 4.20 (1H, dd, $^2J_{6ex,6en}$ = 7.9 Hz, $^3J_{6en,5}$ = 1.5 Hz, H-6*en*), 4.51 (1H, tt, $^3J_{6en,5}$ = 1.5 Hz, $^3J_{6ex,5}$ = 4.5 Hz, $^3J_{4,5}$ = 4.5 Hz, $^4J_{3,5}$ = 1.5 Hz, H-5), 4.80 (1H, ddddd, $^4J_{3,1}$ = 1.5 Hz, $^3J_{2,3}$ = 1.5 Hz, $^3J_{4,3}$ = 4.5 Hz, $^3J_{3,F}$ = 48.2 Hz, $^4J_{3,5}$ = 1.5 Hz, H-3), 5.48 (1H, t, $^3J_{2,1}$ = 1.5 Hz, $^4J_{3,1}$ = 1.5 Hz, H-1); ^{13}C {^{1}H} NMR (75 MHz, $CDCl_3$) δ 60.8 (d, $^2J_{C,F}$ = 25.1 Hz, C-2), 63.8 (d, $^4J_{C,F}$ = 3.4 Hz, C-6), 65.0 (d, $^2J_{C,F}$ = 17.7 Hz, C-4), 74.2 (s, C-5), 88.9 (d, $^1J_{C,F}$ = 183.0 Hz, C-3), 99.5 (s, C-1); ^{19}F NMR (282 MHz, $CDCl_3$): δ −199.16 (ddddd, $^2J_{3,F}$ = 48.2 Hz, $^3J_{4,F}$ = 26.4 Hz, $^3J_{2,F}$ = 14.7 Hz, $^5J_{6ex,F}$ = 1.2 Hz, $^4J_{F,OH}$ = 4.0 Hz). HRMS (ESI) *m/z* $[M + Na]^+$ calcd for $C_6H_8FN_3NaO_3$: 212.0441; found: 212.0446.

1,6-Anhydro-2-azido-2,3,4-trideoxy-3,4-difluoro-β-D-galactopyranose (22)

DAST (0.37 mL, 2.80 mmol) was added under cooling (−25 °C) and stirring to a suspension of fluorohydrin **20** (100 mg, 0.53 mmol) in anhydrous dichloromethane (0.3 mL). The cooling bath was removed after 5 min and stirring continued at rt for 4 days until TLC in S12 indicated a full conversion of **21**. The reaction mixture was diluted with dichloromethane, and methanol (0.5 mL) was added under cooling (0 °C) to destroy the excess of DAST. After 20 min was the reaction mixture washed with 1% HCl, saturated $NaHCO_3$, dried and concentrated. Chromatography in AcOEt/petroleum ether 1:6 afforded difluoro derivative **22** (90 mg, 89%). The product was shortly dried at a pressure about 20 mbar to avoid losses by evaporation. Product for analyses was obtained by recrystallization from ethyl acetate/heptane (66 mg, 65%); mp 64–65 °C; $[\alpha]_D^{20}$ +58 (*c* 0.31, $CHCl_3$); ^{1}H NMR (500 MHz, $CDCl_3$) δ 3.78–3.86 (2H, m, H-2, H-6*ex*), 4.39 (1H, dd, $^3J_{5,6en}$ = 1.0 Hz, $^2J_{6ex,6en}$ = 7.9 Hz, H-6*en*), 4.68 (1H,

dddd, $^3J_{4,5}$ = 4.5 Hz, $^3J_{5,6en}$ = 1.0 Hz, $^3J_{5,6ex}$ = 4.8 Hz, $^3J_{5,F4}$ = 4.7 Hz, H-5), 4.80 (1H, dddd, $^2J_{4,F4}$ = 43.6 Hz, $^3J_{4,5}$ = 4.4 Hz, $^3J_{4,3}$ = 4.5 Hz, $^3J_{4,F3}$ = 24.5 Hz, H-4), 4.90 (1H, ddddd, $^4J_{3,1}$ = 1.6 Hz, $^3J_{3,F4}$ = 4.3 Hz, $^3J_{3,4}$ = 4.5 Hz, $^2J_{3,F3}$ = 48.8 Hz, $^3J_{3,2}$ = 1.6 Hz, H-3), 5.50 (1H, dt, $^3J_{2,1}$ = 1.7 Hz, $^4J_{3,1}$ = 1.7 Hz, $^5J_{1,F4}$ = 5.2 Hz, H-1); ^{13}C {^{1}H} NMR (75 MHz, $CDCl_3$) δ 62.0 (dd, $^2J_{C,F3}$ = 24.2 Hz, $^3J_{C,F4}$ = 2.5 Hz, C-2), 64.4 (dd, $^3J_{C,F4}$ = 4.2 Hz, $^4J_{C,F3}$ = 1.6 Hz,C-6), 71.9 (dd, $^2J_{C,F4}$ = 27.2 Hz, $^3J_{C,F3}$ = 1.1 Hz, C-5), 82.6 (dd, $^1J_{C,F4}$ = 194.9 Hz, $^2J_{C,F3}$ = 16.1 Hz, C-4), 86.3 (dd, $^1J_{C,F3}$ = 190.9 Hz, $^2J_{C,F4}$ = 15.3 Hz, C-3), 99.6 (s, C-1); ^{19}F NMR (470 MHz, $CDCl_3$) δ −207.18 (1F, ddddd, $^2J_{F4,4}$ = 43.8 Hz, $^3J_{F4,3}$ = 4.3 Hz, $^3J_{F4,5}$ = 4.7 Hz, $^3J_{F4,F3}$ = 5.3 Hz, $^5J_{F4,1}$ = 5.0 Hz, F-4), −199.54 (1F, dddd, $^2J_{F3,3}$ = 48.8 Hz, $^3J_{F3,4}$ = 24.7 Hz, $^3J_{F3,2}$ = 14.3 Hz, $^3J_{F3,F4}$ = 5.3 Hz, F-3). HRMS (ESI) *m/z* $[M + Na]^+$ calcd for $C_6H_7F_2N_3NaO_2$: 214.0398; found: 214.0401. Anal. Calcd for $C_6H_7F_2N_3O_2$: C, 37.70; H, 3.39; N, 21.98. Found: C, 37.76; H, 3.51; N, 21.85.

1,6-Anhydro-2-azido-2-deoxy-3,4-di-*O*-(1-methoxybenzylidene)-β-D-galactopyranose (25)

DAST (0.32 mL, 2.42 mmol) was added dropwise to a stirred and cooled (−25 °C) solution of **12** (176 mg, 0.60 mmol) in dichloromethane (2 mL), the cooling bath was removed after 20 min and stirring continued at rt for 60 h. The reaction was diluted with dichloromethane, quenched by addition of methanol (0.6 mL) and washed with aqueous $NaHCO_3$. The water phase was reextracted with dichloromethane, and the combined organic layers were dried and concentrated. Chromatography in S12 furnished orthoester **25** (144 mg, 78%); mp 53–56 °C; $[\alpha]_D^{20}$ +80 ($CHCl_3$, 0.23); ^{1}H NMR (500 MHz, $CDCl_3$) δ 3.08, (s, 3H, C*H*$_3$), 3.46 (1H, ddt, $^2J_{6ex,6en}$ = 7.9 Hz, $^3J_{6ex,5}$ = 5.3 Hz, $^4J_{6ex,1}$ = 0.7 Hz, $^4J_{6ex,4}$ = 0.7 Hz, H-6*ex*), 3.73 (1H, bs, H-2), 3.77 (1H, dd, $^2J_{6en,6ex}$ = 7.8 Hz, $^3J_{6en,5}$ = 0.7 Hz, H-6*en*), 4.55 - 4.59 (2H, m, H-3, H-5), 4.71 (1H, t, $^3J_{4,3}$ = 6.6 Hz, $^3J_{4,5}$ = 6.6 Hz, H-4), 5.49 (1H, bs, H-1), 7.40 – 7.43, (3H, m, C_5H_6), 7.57 – 7.61 (2H, m, C_5H_6); ^{13}C {^{1}H} NMR (125 MHz, $CDCl_3$) δ 51.4 (*C*H$_3$), 60.7 (C-2), 63.8 (C-6), 70.3 (C-4), 72.1 (C-5), 73.3 (C-3), 99.8 (C-1), 120.6 (C), 126.3, 128.4, 129.6 (3 × *C*H, C_6H_5), 134.3 (C_{ipso}, C_6H_5). HRMS (ESI) *m/z* $[M + Na]^+$ calcd for $C_{14}H_{15}N_3NaO_5$: 328.0903; found: 328.0901. Anal. Calcd for $C_{14}H_{15}N_3O_5$: C, 55.08; H, 4.95; N, 13.76. Found: C, 55.07; H, 4.86; N, 13.58.

1,6-Anhydro-2-azido-2,4-dideoxy-4-fluoro-β-D-glucopyranose (26)

A mixture of **18** (400 mg, 2.36 mmol), KHF_2 (950 mg, 12.16 mmol) and ethylene glycol (3.5 mL) was stirred and heated to 175 °C under argon for 45 min. The reaction turned dark brown and TLC in S11 showed an almost complete conversion of **18**. The reaction mixture was poured into saturated aqueous $NaHCO_3$ (25 mL), silica gel (10 g) was added and the resulting mixture was concentrated at 60 °C to near dryness. Chromatography in S11 afforded first the starting azido epoxide **18** (25 mg, 6%), followed by 4-fluoro derivative **26** (225 mg, 50%, 40−60% in repeated experiments); mp 87–89 °C (ethyl acetate); $[\alpha]_D^{20}$ +9 (*c* 0.20, $CHCl_3$); ^1H NMR (300 MHz, $CDCl_3$) δ 2.48 (1H, d, $^3J_{3,OH}$ = 6.3 Hz, OH), 3.25 (1H, bs, H-2), 3.80 (1H, ddd, $^2J_{6ex,6en}$ = 7.9 Hz, $^3J_{6ex,5}$ = 5.5 Hz, $^4J_{6ex,F}$ = 4.3 Hz, H-6*ex*), 4.04 (1H, bd, $^3J_{3,F}$ = 16.5 Hz, H-3), 4.09 (1H, d, $^2J_{6ex,6en}$ = 7.9 Hz, H-6*en*), 4.48 (1H, ddd, $^2J_{4,F}$ = 46.3 Hz, $^3J_{4,5}$ = 2.0 Hz, $^3J_{4,3}$ = 2.0 Hz, H-4), 4.77 (1H, dd, $^3J_{6ex,5}$ = 5.6 Hz, $^3J_{5,F}$ = 12.9 Hz, H-5), 5.55 (1H, dd, $^3J_{2,1}$ = 1.4 Hz, $^4J_{1,3}$ = 1.4 Hz, H-1); ^{13}C {^1H} NMR (75 MHz, $CDCl_3$): δ 61.6 (d, $^3J_{C,F}$ = 4.0 Hz, C-2), 64.9 (d, $^3J_{C,F}$ = 9.4 Hz, C-6), 70.0 (d, $^2J_{C,F}$ = 29.6 Hz, C-3), 74.5 (d, $^2J_{C,F}$ = 22.3 Hz, C-5), 89.9 (d, $^1J_{C,F}$ = 182.1 Hz, C-4), 100.9 (s, C-1); ^{19}F NMR (282 MHz, $CDCl_3$) δ −186.44 (dddd, $^2J_{F,4}$ = 46.3 Hz, $^3J_{F,3}$ = 16.7 Hz, $^3J_{F,5}$ = 12.9 Hz, $^4J_{F,6ex}$ = 4.4 Hz). HRMS (ESI) *m/z* $[M + Na]^+$ calcd $C_6H_8FN_3NaO_3$: 212.0441; found: 212.0445. Anal. Calcd for $C_6H_8FN_3O_3$: C, 38.10; H, 4.26; N, 22.22. Found: C, 38.29; H, 4.14; N, 22.16. A continued elution in another experiment starting from 254 mg gave 1,6-anhydro-2-azido-2-deoxy-4-*O*-(2-hydroxyethyl)-β-D-glucopyranose (**27**, 20 mg, 6%); ^1H NMR (500 MHz, $CDCl_3$) δ 2.94 (bs, 1H, CH_2OH), 3.23 (1H, dd, $^3J_{2,1}$ = 1.4 Hz, $^3J_{2,3}$ = 3.6 Hz, H-2), 3.35 (1H, dd, $^3J_{4,5}$ = 1.5 Hz, $^3J_{4,3}$ = 3.6 Hz, H-4), 3.48 (bs, 1H, C3-OH), 3.67 - 3.71 (m, 1H, $OCHHCH_2$), 3.76 (1H, dd, $^3J_{5,6ex}$ = 5.2 Hz, $^2J_{6ex,6en}$ = 7.5 Hz, H-6*ex*), 3.76 – 3.82 (m, 3H, $OCHHCH_2$), 3.88 – 3.90 (1H, m, H-3), 4.02 (1H, dd, $^3J_{5,6en}$ = 0.9 Hz, $^2J_{6ex,6en}$ = 7.6 Hz, H-6*en*), 4.66 – 4.67 (1H, m, H-5), 5.47 (1H, bs, H-1); ^{13}C {^1H} NMR (125 MHz, $CDCl_3$) δ 61.9 (OCH_2CH_2OH), 62.8 (C-2), 66.2 (C-6), 70.3 (C-3), 71.2 (OCH_2CH_2OH), 74.8 (C-5), 80.2 (C-4), 101.1 (C-1). HRMS (ESI): *m/z* $[M + Na]^+$ calcd $C_8H_{13}N_3NaO_5$: 254.0747; found: 254.0744.

1,6-Anhydro-2-azido-2,3,4-trideoxy-3,4-difluoro-β-D-glucopyranose (28) and 2,6-anhydro-3-azido-3,4-dideoxy-4-fluoro-β-D-altropyranosyl fluoride (29)

The reaction was carried out as described for the synthesis of **19** using fluorhydrine **26** (198 mg, 1.05 mmol) as the starting material and DAST (0.6 mL, 4.54 mmol) in anhydrous benzene (5 mL). After quenching with methanol, the reaction mixture was diluted with ethyl acetate, washed with saturated $NaHCO_3$, the water phase reextracted with ethyl acetate, the combined organic extracts washed with 2% HCl, the water phase was reextracted with ethyl acetate and the combined organic extracts washed with brine, dried and concentrated. Chromatography in S13 gave first D-*gluco*-configured product **28** (146 mg, 73%) which was briefly dried at 20 mbar to avoid losses by evaporation. The product for analyses was obtained by recrystallization from ethyl acetate-heptane (91 mg, 46%); mp 56 °C; $[\alpha]_D^{20}$ +28 (*c* 0.33, $CHCl_3$); ^{1}H NMR (300 MHz, $CDCl_3$) δ 3.38 (1H, dt, $^3J_{2,1}$ = 1.7 Hz, $^3J_{2,3}$ = 1.7 Hz, $^3J_{2,F3}$ = 18.3 Hz, H-2), 3.85 (1H, dddd, $^3J_{6ex,5}$ = 5.9 Hz, $^4J_{6ex,F4}$ = 4.7 Hz, $^5J_{6ex,F3}$ = 1.9 Hz, $^2J_{6ex,6en}$ = 8.0 Hz, H-6ex), 4.02 (1H, dd, $^3J_{6en,5}$ = 1.2 Hz, $^2J_{6en,6ex}$ = 8.0 Hz, H-6en), 4.59 (1H, dddd, $^2J_{4,F4}$ = 44.5 Hz, $^3J_{4,3}$ = 3.5 Hz, $^3J_{4,F3}$ = 13.5 Hz, $^3J_{4,5}$ = 1.8 Hz, H-4), 4.78 (1H, dddt, $^3J_{3,F4}$ = 13.1 Hz, $^3J_{3,4}$ = 3.5 Hz, $^2J_{3,F3}$ = 43.6 Hz, $^3J_{3,2}$ = 1.7 Hz, $^4J_{3,1}$ = 1.7 Hz, H-3), 4.78–4.82 (1H, m, H-5), 5.58 (1H, t, $^3J_{1,2}$ = 1.7 Hz, $^4J_{1,3}$ = 1.7 Hz, H-1); ^{13}C {^{1}H} NMR (75 MHz, $CDCl_3$) δ 58.4 (dd, $^2J_{C,F3}$ = 25.5 Hz, $^3J_{C,F4}$ = 3.0 Hz, C-2), 64.1 (dd, $^3J_{C,F4}$ = 8.9 Hz, $^4J_{C,F3}$ = 3.1 Hz, C-6), 73.5 (dd, $^2J_{C,F4}$ = 21.2 Hz, $^3J_{C,F3}$ = 0.9 Hz, C-5), 85.9 (dd, $^1J_{C,F4}$ = 181.9 Hz, $^2J_{C,F3}$ = 31.1 Hz, C-4), 87.8 (dd, $^1J_{C,F3}$ = 182.6 Hz, $^2J_{C,F4}$ = 34.1 Hz, C-3), 100.1 (s, C-1); ^{19}F NMR (470 MHz, $CDCl_3$) δ −189.75 (1F, ddddd, $^2J_{F4,4}$ = 44.3 Hz, $^3J_{F4,3}$ = 12.9 Hz, $^3J_{F4,5}$ = 11.9 Hz, $^3J_{F4,F3}$ = 12.3 Hz, $^4J_{F4,6ex}$ = 4.7 Hz, F-4), −184.03 (1F, dddd, $^2J_{F3,3}$ = 43.6 Hz, $^3J_{F3,4}$ = 13.3 Hz, $^3J_{F3,2}$ = 18.1 Hz, $^3J_{F3,F4}$ = 12.3 Hz, F-3). HRMS (ESI) *m/z* $[M + Na]^+$ calcd for $C_6H_7F_2N_3NaO_2$: 214.0398; found: 214.0395. Anal. Calcd for $C_6H_7F_2N_3O_2$: C, 37.70; H, 3.39; N, 21.98. Found: C, 37.65; H, 3.71; N, 21.84. A continued elution gave rearranged fluoride **29** (23 mg, 12%); mp 92–95 °C; ^{1}H NMR (500 MHz, $CDCl_3$) δ 3.84 (1H, ddd, $^3J_{6a,5}$ = 1.6 Hz, $^2J_{6a,6b}$ = 11.0 Hz, H-6a), 3.99 (1H, td, $^3J_{2,1}$ = 2.0 Hz, $^3J_{2,3}$ = 4.4 Hz, $^3J_{2,F4}$ = 4.4 Hz, H-2), 4.33 – 4.42 (2H, m, H-6b, H-5), 4.42 (1H, td, $^3J_{3,2}$ = 4.4 Hz, $^3J_{3,4}$ = 8.1 Hz, $^3J_{3,F4}$ = 7.9 Hz, H-3), 5.05 (1H, dddd, $^2J_{4,F4}$ = 49.0 Hz, $^3J_{4,3}$ = 8.1 Hz, $^5J_{4,F1}$ = 1.4 Hz, $^3J_{4,5}$ = 1.9 Hz, H-4), 5.78 (1H, ddd, $^3J_{1,2}$ = 2.1 Hz, $^2J_{1,F1}$ = 62.4 Hz, H-1); ^{13}C

{^{1}H} NMR (75 MHz, $CDCl_3$) δ 56.7 (dd, $^2J_{C,F4}$ = 15.8 Hz, $^3J_{C,F1}$ = 5.7 Hz, C-3), 65.7 (d, $^3J_{C,F4}$ = 7.5 Hz, C-6), 66.4 (d, $^2J_{C,F1}$ = 20.7 Hz, C-2), 70.0 (dd, $^2J_{C,F4}$ = 20.3 Hz, $^3J_{C,F1}$ = 2.3 Hz, C-5), 86.1 (dd, $^1J_{C,F4}$ = 197.2 Hz, $^4J_{C,F1}$ = 4.8 Hz, C-4), 104.9 (d, $^1J_{C,F1}$ = 227.8 Hz,C-1); ^{19}F NMR (470 MHz, $CDCl_3$): δ −203.58 (1F, dddt, $^2J_{F4,4}$ = 49.0 Hz, $^3J_{F4,3}$ = 7.9 Hz, $^3J_{F4,5}$ = 9.9 Hz, $^4J_{F4,6en}$ = 3.8 Hz, $^5J_{F4,F1}$ = 3.9 Hz, F-4), −127.13 (1F, ddq, $^2J_{F1,1}$ = 62.6 Hz, $^3J_{F1,2}$ = 0.7 Hz, $^5J_{F1,4}$ = 1.4 Hz, $^5J_{F1,F4}$ = 3.9 Hz, $^5J_{F1,6ex}$ = 0.7 Hz, F-1). HRMS (APCI): *m/z* $[M + H − N_2]^+$ calcd for $C_6H_8F_2NO_2$: 164.0518; found: 164.0519.

1,6-Anhydro-2-azido-2,4-dideoxy-4-fluoro-β-D-galactopyranose (31)

Lithium azide (0.3 g, 6.1 mmol), ammonium chloride (1.4 g, 26.2 mmol), 1,6:2,3-dianhydro-4-deoxy-4-fluoro-β-D-talopyranose [7] (**30**, 250 mg, 1.71 mmol) and 2-methoxyethanol (2 mL) were stirred and heated to 100 °C for 12 h when TLC in freshly prepared ethyl acetate/heptane/ethanol/NH_3(aq) 40:45:9:2 and GC/MS indicated absence of the starting **30**. The reaction mixture was diluted with ethyl acetate, filtered through silica gel and concentrated. Chromatography in S12 afforded a chromatographically homogenous mixture of **31** and **32** (310 mg, 96%). Recrystallization from ethyl acetate-heptane gave **31** (257 mg, 74%); mp 105–107 °C; $[\alpha]_D^{20}$ +53 (*c* 0.20, $CHCl_3$); ^{1}H NMR (300 MHz, $CDCl_3$) δ 2.45 (dd, 1H, $^3J_{OH,3}$ = 3.8 Hz, $^4J_{OH,F}$ = 3.8 Hz, O*H*), 3.69 (1H, dt, $^3J_{2,1}$ = 1.4 Hz, $^3J_{3,2}$ = 1.4 Hz, $^4J_{F,2}$ = 3.6 Hz, H-2), 3.75 (1H, dd, $^3J_{5,6ex}$ = 5.0 Hz, $^2J_{6ex,6en}$ = 7.8 Hz, H-6*ex*), 4.28 (1H, dddq, $^4J_{3,1}$ = 1.4 Hz, $^4J_{3,5}$ = 1.4 Hz, $^3J_{3,4}$ = 4.5 Hz, $^3J_{3,F}$ = 3.7 Hz, $^3J_{3,2}$ = 1.4 Hz, $^3J_{3,OH}$ = 3.8 Hz, H-3), 4.46 (1H, ddd, $^3J_{5,6en}$ = 0.6 Hz, $^2J_{6ex,6en}$ = 7.8 Hz, $^4J_{6en,F}$ = 0.6 Hz, H-6*en*), 4.64 (1H, ddd, $^3J_{5,4}$ = 4.5 Hz, $^3J_{5,6en}$ = 0.6 Hz, $^3J_{5,6ex}$ = 5.0 Hz, H-5), 4.81 (1H, ddd, $^2J_{4,F}$ = 45.4 Hz, $^3J_{4,5}$ = 4.5 Hz, $^3J_{4,3}$ = 4.5 Hz, H-4), 5.48 (1H, ddd, $^3J_{2,1}$ = 1.4 Hz, $^4J_{3,1}$ = 1.4 Hz, $^5J_{1,F}$ = 4.9 Hz, H-1); ^{13}C {^{1}H} NMR (75 MHz, $CDCl_3$) δ 63.8 (d, $^3J_{C,F}$ = 2.5 Hz, C-2), 64.3 (d, $^3J_{C,F}$ = 1.2 Hz, C-6), 68.3 (d, $^2J_{C,F}$ = 16.6 Hz, C-3), 72.0 (d, $^2J_{C,F}$ = 27.4 Hz, C-5), 84.3 (d, $^1J_{C,F}$ = 187.1 Hz, C-4), 100.4 (d, $^4J_{C,F}$ = 1.1 Hz, C-1); ^{19}F NMR (282 MHz, $CDCl_3$): δ −205.70 (dq, $^2J_{4,F}$ = 45.6 Hz, $^5J_{1,F}$ = 4.7 Hz, $^4J_{F,OH}$ = 3.7 Hz, $^3J_{F,3}$ = 3.7 Hz). HRMS (ESI) *m/z* $[M + Na]^+$ calcd for $C_6H_8FN_3NaO_3$: 212.0441; found: 212.0447. Anal. Calcd for $C_6H_8FN_3O_3$: C, 38.10; H, 4.26; N, 22.22. Found: C, 38.04; H, 4.11; N, 21.79. NMR of the concentrated mother liquor (56 mg) showed also the resonances of 1,6-anhydro-3-azido-3,4-dideoxy-

4-fluoro-β-D-idopyranose (**32, 31**/**32** ca 1:1), ^{1}H NMR (500 MHz, $CDCl_3$) δ 3.54 (1H, bd, $^3J_{2,3}$ = 8.4 Hz, H-2), 3.62 (1H, dt, $^3J_{3,4}$ = 8.5 Hz, $^3J_{3,F}$ = 18.3 Hz, $^3J_{3,2}$ = 8.5 Hz, H-3), 3.84 (1H, dd, $^3J_{5,6ex}$ = 4.9 Hz, $^2J_{6ex,6en}$ = 8.4 Hz, H-6*ex*), 4.13 (1H, dd, $^3J_{5,6en}$ = 2.0 Hz, $^2J_{6ex,6en}$ = 8.4 Hz, H-6*en*), 4.53 (1H, dddd, $^2J_{4,F}$ = 49.0 Hz, $^3J_{4,5}$ = 4.4 Hz, $^3J_{4,3}$ = 8.3 Hz, $^4J_{4,6ex}$ = 1.1 Hz, H-4), 4.62 - 4.64 (1H, m, H-5), 5.34 (1H, dd, $^3J_{2,1}$ = 1.8 Hz, $^5J_{1,F}$ = 5.6 Hz, H-1); ^{13}C {^{1}H} NMR (125 MHz, $CDCl_3$) δ 65.2 (d, $^3J_{C,F}$ = 1.6 Hz, C-6), 65.4 (d, $^2J_{C,F}$ = 17.9 Hz, C-3), 72.6 (d, $^2J_{C,F}$ = 25.2 Hz, C-5), 74.2 (d, $^3J_{C,F}$ = 4.8 Hz, C-2), 88.3 (d, $^1J_{C,F}$ = 189.0 Hz, C-4), 101.1 (d, $^4J_{C,F}$ = 2.0 Hz, C-1); ^{19}F NMR (470 MHz, $CDCl_3$) δ −193.00 (dddd, $^2J_{4,F}$ = 48.9 Hz, $^3J_{3,F}$ = 18.4 Hz, $^5J_{1,F}$ = 5.6 Hz, $^3J_{5,F}$ = 1.3 Hz).

1,6-Anhydro-2-acetamido-4-*O*-acetyl-2,3-dideoxy-3-fluoro-β-D-glucopyranose (40)

Starting **19** (574 mg, 2.06 mmol) was dissolved in ethanol (10 mL) to which acetyl chloride had been added (0.3 mL, 4.2 mmol) and hydrogenated in the presence of 10% Pd/C (250 mg) at rt for 5 h. The catalyst was filtered off and the filtrate neutralized with methanolic MeONa (1 M). Acetic acid anhydride (1.9 mL) was added and the resulting solution stirred overnight, and then concentrated. TLC showed incomplete acetylation and the residue was reacetylated with acetic acid anhydride (0.4 mL, 4.2 mmol) in anhydrous pyridine (1.5 mL) for 4 h, the pyridine solution was concentrated, the residue codistilled with toluene and chromatographed in S1 to afford **40** (187 mg, 37%); mp 113–115 °C; $[\alpha]_D^{20}$ −89 ($CHCl_3$, c 0.20); ^{1}H NMR (500 MHz, $CDCl_3$) δ 2.02, 2.15 (2 × 3H, 2 × s, COCH_3), 3.84 (1H, ddd, $^3J_{5,6ex}$ = 5.8 Hz, $^2J_{6ex,6en}$ = 7.9 Hz, $^5J_{6ex,F}$ = 2.1 Hz, H-6ex), 4.17 (1H, dt, $^3J_{5,6en}$ = 1.1 Hz, $^2J_{6ex,6en}$ = 7.9 Hz, $^5J_{6en,F}$ = 1.1 Hz, H-6en), 4.29 (1H, ddq, $^3J_{2,1}$ = 1.8 Hz, $^3J_{2,NH}$ = 9.7 Hz, $^3J_{2,3}$ = 1.8 Hz, $^3J_{2,F}$ = 14.7 Hz, $^4J_{2,4}$ = 1.8 Hz, H-2), 4.41 (1H, dp, $^4J_{3,1}$ = 1.8 Hz, $^3J_{3,4}$ = 1.8 Hz, $^4J_{3,5}$ = 1.8 Hz, $^2J_{3,F}$ = 43.1 Hz, $^3J_{3,2}$ = 1.8 Hz, H-3), 4.61 (1H, dq, $^3J_{5,6ex}$ = 5.7 Hz, $^3J_{5,6en}$ = 1.1 Hz, $^3J_{5,4}$ = 1.8 Hz, $^4J_{5,3}$ = 1.8 Hz, H-5), 4.86 (1H, dp, $^3J_{4,5}$ = 1.8 Hz, $^3J_{4,3}$ = 1.8 Hz, $^4J_{4,2}$ = 1.8 Hz, $^5J_{4,1}$ = 1.0 Hz, $^3J_{4,F}$ = 14.2 Hz, H-4), 5.39 (1H, t, $^3J_{1,2}$ = 1.9Hz, $^4J_{1,3}$ = 1.9 Hz, H-1), 6.25 (1H, d, $^3J_{NH,2}$ = 9.7 Hz, NH); ^{13}C {^{1}H} NMR (125 MHz, $CDCl_3$) δ 20.6, 22.9 (2 × s, CO*C*H_3), 48.3 (d, $^2J_{C,F}$ = 26.7 Hz, C-2), 65.2 (d, $^4J_{C,F}$ = 4.0 Hz, C-6), 69.2 (d, $^2J_{C,F}$ = 31.5 Hz, C-4), 73.1 (s, C-5), 87.9 (d, $^1J_{C,F}$ = 184.5 Hz, C-3), 100.7 (d, $^3J_{C,F}$ = 0.9 Hz, C-1), 169.6, 169.7 (2 × s, *C*OCH_3); ^{19}F NMR (470 MHz, $CDCl_3$) δ −180.11 (dt, $^2J_{F,3}$ = 43.1 Hz, $^3J_{F,4}$ = 14.4

Hz, ${}^{3}J_{F,2}$ = 14.4 Hz); HRMS (ESI) *m/z* [M + Na]$^+$ calcd for $C_{10}H_{14}FNNaO_5$: 270.0748; found: 270.0749. Anal. Calcd for $C_{10}H_{14}FNO_5$: C, 48.58; H, 5.71; N, 5.67. Found: C, 48.54; H, 5.52; N, 5.50.

2-Methyl-(4,6-di-*O*-acetyl-1,2,3-trideoxy-3-fluoro-α-D-glucopyrano)-[2,1-*d*]-2-oxazoline (41)

A solution of acetamide **40** (160 mg, 0.65 mmol) in a mixture of acetic anhydride-acetic acid-sulfuric acid (25:10:0.25, 1 mL) was stirred at rt for 24 h. A solution of sulfuric acid (20 µL) in acetic anhydride (0.5 mL) was added and stirring continued for another 24 h until GC/MS indicated absence of the starting **40**. The reaction was cooled (−5 °C) and a saturated solution of $NaHCO_3$ was added in portions until gas evolution ceased. The reaction mixture was extracted with ethyl acetate (5×), the combined extracts dried and concentrated. NMR showed the presence of at least 3 products. Crystallization from ethyl acetate-heptane afforded oxazoline **41** (75 mg, 40%); mp 95–101 °C; $[\alpha]_D^{20}$ +35 ($CHCl_3$, c 0.21); ^{1}H NMR (500 MHz, $CDCl_3$) δ 2.09, 2.10 (2 × s, 3 × 3H, C*H*$_3$), 3.58 (1H, ddd, ${}^{3}J_{5,4}$ = 9.3 Hz, ${}^{3}J_{5,6a}$ = 3.3 Hz, ${}^{3}J_{5,6b}$ = 5.3 Hz, H-5), 4.14–4.20 (2H, m, H-6), 4.30–4.35 (1H, m, H-2), 4.92 (1H, ddd, ${}^{2}J_{3,F}$ = 43.7 Hz, ${}^{3}J_{3,4}$ = 1.6 Hz, ${}^{3}J_{3,2}$ = 2.8 Hz, H-3), 5.08 (1H, ddt, ${}^{3}J_{4,F}$ = 22.3 Hz, ${}^{3}J_{4,5}$ = 9.3 Hz, ${}^{3}J_{4,3}$ = 1.6 Hz, ${}^{4}J_{4,2}$ = 1.6 Hz, H-4), 6.02 (1H, d, ${}^{3}J_{1,2}$ = 7.4 Hz, H-1); ^{13}C {^{1}H} NMR (125 MHz, $CDCl_3$) δ 13.9, 20.7, 20.8 (3 × s, *C*H$_3$), 63.2 (s, C-6), 64.7 (d, ${}^{2}J_{C,F}$ = 28.7 Hz, C-2), 66.9 (d, ${}^{3}J_{C,F}$ = 2.4 Hz, C-5), 67.8 (d, ${}^{2}J_{C,F}$ = 30.9 Hz, C-4), 88.1 (d, ${}^{1}J_{C,F}$ = 178.0 Hz, C-3), 99.30 (s, C-1), 167.2, 169.5, 170.6 (3 × s, *C*OCH$_3$); ^{19}F NMR (470 MHz, $CDCl_3$) δ −173.71 (ddd, ${}^{2}J_{3,F}$ = 43.7 Hz, ${}^{3}J_{4,F}$ = 22.3 Hz, ${}^{3}J_{2,F}$ = 12.8 Hz); HRMS (ESI) *m/z* [M + Na]$^+$ calcd for $C_{12}H_{16}FNNaO_6$: 312.0854; found: 312.0854. Anal. Calcd for $C_{12}H_{16}FNO_6$: C, 49.83; H, 5.58; N, 4.84. Found: C, 49.75; H, 5.47; N, 4.64.

1,6-Di-*O*-acetyl-2-azido-4-*O*-benzyl-2,3-dideoxy-3-fluoro-D-glucopyranose (42)

Azide **19** (421 mg, 1.51 mmol) was acetolysed in Ac_2O/TESOTf solution (4 mL) following the general procedure. The reaction was stirred for 4 h, then left to stand overnight at 5 °C. GC/MS indicated absence of **19**. Work-up afforded product **42** (553 mg, 96%) as a mixture of anomers (77/23 α/β) that was used in the next reaction. In another experiment, crystallization from methyl-*tert*-butyl ether gave the α-anomer **42α** (118 mg, 39%, starting from 219 mg) for characterization; mp 72–75

°C; $[\alpha]_D^{20}$ +141 ($CHCl_3$, 0.25); ^{1}H NMR (300 MHz, $CDCl_3$) δ 1.99, 2.14 (2 × 3H, 2 × s, $COCH_3$), 3.66–3.77 (2H, m, H-2, H-4), 3.89 (1H, ddd, $^3J_{6b,5}$ = 3.7 Hz, $^3J_{6a,5}$ = 2.0 Hz, $^3J_{4,5}$ = 10.1 Hz, H-5), 4.23 (1H, dt, $^2J_{6a,6b}$ = 12.2 Hz, $^3J_{6a,5}$ = 2.0 Hz, $^5J_{6a,F}$ = 2.0 Hz, H-6a), 4.29 (1H, dd, $^3J_{6b,5}$ = 3.7 Hz, $^2J_{6a,6b}$ = 12.2 Hz, H-6b), 4.63 (1H, d, ^2J = 11.1 Hz, CH_2-Bn), 4.89 (1H, d, ^2J = 11.1 Hz, CH_2-Bn), 4.98 (1H, ddd, $^2J_{3,F}$ = 53.1 Hz, $^3J_{3,2}$ = 10.0 Hz, $^3J_{4,3}$ = 8.3 Hz, H-3), 6.22 (1H, dd, $^3J_{2,1}$ = 3.9 Hz, $^4J_{F,1}$ = 3.9 Hz, H-1), 7.31–7.37 (5H, m, Bn); ^{13}C {^{1}H} NMR (75 MHz, $CDCl_3$) δ 20.7, 20.8 (2 × s, $COCH_3$), 60.8 (d, $^2J_{C,F}$ = 17.7 Hz, C-2), 62.0 (s, C-6), 70.4 (d, $^3J_{C,F}$ = 8.8 Hz, C-5), 74.2 (d, $^2J_{C,F}$ = 16.7 Hz, C-4), 74.2 (d, $^4J_{C,F}$ = 3.2 Hz, CH_2Bn), 90.1 (d, $^3J_{C,F}$ = 9.6 Hz, C-1), 94.5 (d, $^1J_{C,F}$ = 185.2 Hz, C-3), 128.3, 128.5, 128.6, 136.8 (4 × s, *C*-Bn), 168.5, 170.4 (2 × s, $COCH_3$); ^{19}F NMR (282 MHz, $CDCl_3$) δ −191.66 (ddd, $^2J_{3,F}$ = 53.1 Hz, $^3J_{4,F}$ = 12.8 Hz, $^3J_{2,F}$ = 12.8 Hz). HRMS (ESI) *m/z* $[M + Na]^+$ calcd for $C_{17}H_{20}FN_3NaO_6$: 404.1228; found: 404.1229. Anal. Calcd for $C_{17}H_{20}FN_3O_6$: C, 53.54; H, 5.29; N, 11.02. Found: C, 53.61; H, 5.14; N, 10.75. NMR data for *β*-anomer **42β**: ^{1}H NMR (300 MHz, $CDCl_3$) δ 1.99, 2.18 (2 × 3H, 2 × s, $COCH_3$), 3.59–3.79 (3H, m, H-2, H-4, H-5), 4.20- 4.32 (2H, m, H-6a,b), 4.60 (1H, d, ^2J = 11.2 Hz, CH_2-Bn), 4.61 (1H, ddd, $^2J_{3,F}$ = 50.8 Hz, $^3J_{3,2}$ = 9.5 Hz, $^3J_{4,3}$ = 7.6 Hz, H-3), 4.89 (1H, d, ^2J = 11.2 Hz, CH_2-Bn), 5.45 (1H, dd, $^3J_{2,1}$ = 8.6 Hz, $^3J_{F,1}$ = 0.6 Hz, H-1), 7.31–7.38 (5H, m, Bn); ^{13}C {^{1}H} NMR (75 MHz, $CDCl_3$) δ 20.7, 20.9 (2 × s, $COCH_3$), 62.1 (d, $^4J_{C,F}$ = 1.5 Hz, C-6), 63.2 (d, $^2J_{C,F}$ = 18.4 Hz, C-2), 72.7 (d, $^3J_{C,F}$ = 9.9 Hz, C-5), 74.0 (d, $^2J_{C,F}$ = 16.9 Hz, C-4), 74.2 (d, $^4J_{C,F}$ = 2.9 Hz, CH_2Bn), 91.9 (d, $^3J_{C,F}$ = 11.3 Hz, C-1), 96.2 (d, $^1J_{C,F}$ = 187.9 Hz, C-3), 128.3, 128.5, 128.6, 136.8 (4 × s, *C*-Bn), 168.6, 170.5 (2 × s, $COCH_3$); ^{19}F NMR (282 MHz, $CDCl_3$) δ −187.18 (dt, $^2J_{3,F}$ = 50.8 Hz, $^3J_{4,F}$ = 12.7 Hz, $^3J_{2,F}$ = 12.7 Hz).

2-Acetamido-1,4,6-tri-*O*-acetyl-2,3-dideoxy-3-fluoro-D-glucopyranose (5)

Starting **42** (553 mg, 1.45 mmol) was dissolved in ethanol (15 mL) to which acetyl chloride had been added (0.2 mL, 2.81 mmol) and hydrogenated in the presence of 10% Pd/C (200 mg) at rt for 24 h. The catalyst was filtered off and the filtrate concentrated. The residue was dissolved in pyridine (5 mL) containing acetic acid anhydride (0.7 mL) and stirred at rt for 24 h, then additional acetic acid anhydride (0.2 mL) was added and stirring continued for 24 h until TLC in S1 showed one product.

The reaction was concentrated and chromatography in S1 afforded syrupy **5** [6] (414 mg, 82%) as a mixture of anomers (α/β 87:13). Individual anomers for characterization were obtained by a second chromatography in isopropylalcohol/chloroform/petroleum ether 2:5:5 which gave first the β-anomer **5β** (40 mg, 9%) followed by the α-anomer **5α** (322 mg, 64%). Data for **5α**: mp 135–137 °C (ethyl acetate-heptane); $[\alpha]_D^{20}$ +81 ($CHCl_3$, 0.12); ^1H NMR (500 MHz, $CDCl_3$) δ 2.02 (3H, s, NHCOCH_3), 2.08, 2.10, 2.16 (3 × 3H, 3 × s, COCH_3), 3.93–3.96 (1H, m, H-5), 4.08 (1H, dt, $^2J_{6a,6b}$= 12.5 Hz, $^3J_{6a,5}$ = 2.0 Hz, $^5J_{6a,F}$ = 2.0 Hz, H-6a), 4.24 (1H, dd, $^2J_{6a,6b}$ = 12.5 Hz, $^3J_{6b,5}$ = 4.2 Hz, H-6b), 4.68 (2H, overlap 2 × ddd, $^2J_{3,F}$ = 55.8 Hz, $^3J_{3,2}$ = 10.2 Hz, $^3J_{4,3}$ = 8.6 Hz, H-3, H-2), 5.28 (1H, ddd, $^3J_{4,F}$ = 12.5 Hz, $^3J_{4,5}$ = 10.3 Hz, $^3J_{4,3}$ = 8.6 Hz, H-4), 5.55 (1H, d, $^3J_{2,NH}$ = 8.1 Hz, N*H*), 6.20 (1H, dd, $^3J_{2,1}$ = 3.6 Hz, $^4J_{1,F}$ = 3.6 Hz, H-1); ^{13}C {^1H} NMR (125 MHz, $CDCl_3$) δ 20.6, 20.7, 20.8 (3 × s, CO*C*H_3), 23.1 (s, NHCO*C*H_3), 50.9 (d, $^2J_{C,F}$ = 17.9 Hz, C-2), 61.3 (d, $^4J_{C,F}$ = 1.2 Hz, C-6), 68.1 (d, $^2J_{C,F}$ = 18.4 Hz, C-4), 69.5 (d, $^3J_{C,F}$ = 6.8 Hz, C-5), 89.6 (d, $^1J_{C,F}$ = 190.1 Hz, C-3), 91.1 (d, $^3J_{C,F}$ = 9.2 Hz, C-1), 168.4, 169.0, 170.0, 171.7 (4 × s, *C*OCH_3); ^{19}F NMR (282 MHz, $CDCl_3$) δ −199.57–199.74 (m). HRMS (ESI) *m/z* $[M + Na]^+$ calcd for $C_{14}H_{20}FNNaO_8$: 372.1065; found: 372.1063. Anal. Calcd for $C_{14}H_{20}FNO_8$: C, 48.14; H, 5.77; N, 4.01. Found: C, 48.03; H, 5.67; N, 3.92. Data for **5β**: mp 177–179 °C (ethyl acetate-heptane); $[\alpha]_D^{20}$ +3.5 ($CHCl_3$, 0.34); ^1H NMR (300 MHz, $CDCl_3$) δ 2.00 (3H, s, NHCOCH_3), 2.08, 2.10, 2.12 (3 × 3H, 3 × s, COCH_3), 3.75–3.82 (1H, m, H-5), 3.88 –3.95 (1H, m, H-2), 4.12 (1H, ddd, $^2J_{6b,6a}$ = 12.5 Hz, $^3J_{6a,5}$ = 2.0 Hz, $^5J_{6a,F}$ = 1.5 Hz, H-6a), 4.28 (1H, dd, $^2J_{6a,6b}$ = 12.5 Hz, $^3J_{6b,5}$ = 4.5 Hz, H-6b), 4.92 (1H, ddd, $^2J_{3,F}$ = 50.9 Hz, $^3J_{3,2}$ = 10.0 Hz, $^3J_{4,3}$ = 8.8 Hz, H-3), 5.18 (1H, ddd, $^3J_{4,F}$ = 12.5 Hz, $^3J_{4,5}$ = 10.0 Hz, $^3J_{4,3}$ = 8.8 Hz, H-4), 5.78 (1H, d, $^3J_{2,NH}$ = 8.3 Hz, N*H*), 5.99 (1H, d, $^3J_{2,1}$ = 8.6 Hz, H-1); ^{13}C {^1H} NMR (75 MHz, $CDCl_3$) δ 20.6, 20.7, 20.8 (3 × s, CO*C*H_3), 23.4 (s, NHCO*C*H_3), 54.9 (d, $^2J_{C,F}$ = 18.6 Hz, C-2), 61.5 (d, $^4J_{C,F}$ = 1.5 Hz, C-6), 68.3 (d, $^2J_{C,F}$ = 18.8 Hz, C-4), 71.8 (d, $^3J_{C,F}$ = 8.0 Hz, C-5), 90.2 (d, $^1J_{C,F}$ = 189.1 Hz, C-3), 91.1 (d, $^3J_{C,F}$ = 10.2 Hz, C-1), 169.2, 169.3, 170.5, 170.7 (4 × s, *C*OCH_3); ^{19}F NMR (282 MHz, $CDCl_3$) δ −194.24 (dt, $^2J_{3,F}$ = 50.9 Hz, $^3J_{4,F}$ = 12.5 Hz, $^3J_{2,F}$ = 12.4 Hz); HRMS (ESI) *m/z* $[M + Na]^+$ calcd for $C_{14}H_{20}FNNaO_8$: 372.1065; found: 372.1067. Anal. Calcd for $C_{14}H_{20}FNO_8$: C, 48.14; H, 5.77; N, 4.01. Found: C, 47.97; H, 5.56; N, 3.82.

2-Acetamido-1,4,6-tri-*O*-acetyl-2,3-dideoxy-3-fluoro-D-galactopyranose (6)

Azide **21** (182 mg, 0.97 mmol) was acetolysed in Ac_2O/TESOTf solution (1.5 mL) for 2 h following the general procedure. The reaction was monitored by TLC in S12. Chromatography in S12 gave **43** (268 mg, 83%) as an inseparable mixture of anomers (α/β 72:28) characterized by NMR and then subjected to hydrogenation. Data for the α-anomer **43α**: ^{1}H NMR (300 MHz, $CDCl_3$) δ 2.03, 2.14, 2.16 (3 × 3H, 3 × s, COC*H*$_3$), 4.06 (3H, m, H-6, H-2), 4.22 (1H, t, $^3J_{6a,5}$ = 6.7 Hz $^3J_{6b,5}$ = 6.7 Hz, H-5), 4.93 (1H, ddd, $^2J_{3,F}$ = 47.8 Hz, $^3J_{3,2}$ = 10.3 Hz, $^3J_{4,3}$ = 3.6 Hz, H-3), 5.64 (1H, ddd, $^3J_{4,F}$ = 5.2 Hz, $^3J_{4,5}$ = 1.0 Hz, $^3J_{4,3}$ = 3.7 Hz, H-4), 6.32 (1H, t, $^3J_{2,1}$ = 4.3 Hz, $^4J_{F,1}$ = 4.3 Hz, H-1); ^{13}C NMR (75 MHz, $CDCl_3$) δ 20.26, 20.29, 20.5 (3 × s, CO*C*H$_3$), 58.0 (d, $^2J_{C,F}$ = 18.6 Hz, C-2), 61.0 (d, $^4J_{C,F}$ = 2.4 Hz, C-6), 66.6 (d, $^2J_{C,F}$ = 17.0 Hz, C-4), 68.6 (d, $^3J_{C,F}$ = 5.3 Hz, C-5), 87.2 (d, $^1J_{C,F}$ = 191.9 Hz, C-3), 90.6 (d, $^3J_{C,F}$ = 9.2 Hz, C-1), 169.0, 170.1, 170.9 (3 × s, *C*OCH$_3$); ^{19}F NMR (282 MHz, $CDCl_3$) δ −200.78 (dddd, $^2J_{3,F}$ = 47.8 Hz, $^3J_{4,F}$ = 5.2 Hz, $^3J_{2,F}$ = 10.6 Hz, $^4J_{1,F}$ = 4.3 Hz). Data for the β-anomer **43β**: ^{1}H NMR (300 MHz, $CDCl_3$) δ 2.05, 2.18, 2.21 (3 × 3H, 3 × s, COC*H*$_3$), 3.91 (2H, m, H-5, H-2), 4.10 (2H, m, H-6), 4.52 (1H, ddd, $^2J_{3,F}$ = 46.5 Hz, $^3J_{3,2}$ = 10.1 Hz, $^3J_{4,3}$ = 3.7 Hz, H-3), 5.47 (1H, d, $^3J_{2,1}$ = 8.5 Hz, H-1), 5.55 (1H, ddd, $^3J_{4,F}$ = 5.2 Hz, $^3J_{4,5}$ = 1.1 Hz, $^3J_{4,3}$ = 3.7 Hz, H-4); ^{13}C NMR (75 MHz, $CDCl_3$) δ 20.56, 20.62, 20.8 (3 × s, CO*C*H$_3$), 60.89 (d, $^2J_{C,F}$ = 18.9 Hz, C-2), 60.94 (d, $^4J_{C,F}$ = 2.8 Hz, C-6), 65.9 (d, $^2J_{C,F}$ = 16.7 Hz, C-4), 71.0 (d, $^3J_{C,F}$ = 6.0 Hz, C-5), 89.2 (d, $^1J_{C,F}$ = 194.3 Hz, C-3), 92.2 (d, $^3J_{C,F}$ = 10.9 Hz, C-1), 168.6, 169.6, 170.3 (3 × s, *C*OCH$_3$); ^{19}F NMR (282 MHz, $CDCl_3$) δ −196.51 (ddd, $^2J_{3,F}$ = 46.5 Hz, $^3J_{4,F}$ = 5.2 Hz, $^3J_{2,F}$ = 11.3 Hz). The product **43** (216 mg, 0.65 mmol) was hydrogenated in ethanol (12 mL) containing Ac_2O (1 mL) and 10% Pd/C (100 mg) for 3 h following the general procedure. The reaction was monitored by TLC in S1. Chromatography of the crude product in ethyl acetate-petroleum ether-isopropyl alcohol 1:1:0.3 gave first β-anomer **6β** [6] (32 mg after recrystallization from ethyl acetate-diethyl ether-heptane, 14%), mp 158–160 °C; $[\alpha]_D^{20}$ +35 (*c* 0.23, $CHCl_3$); ^{1}H NMR (500 MHz, $CDCl_3$) δ 2.00 (3H, s, NHCOC*H*$_3$), 2.05, 2.14, 2.17 (3 × 3H, 3 × s, COC*H*$_3$), 4.00 (1H, ddd, $^3J_{4,5}$ = 1.3 Hz, $^3J_{6b,5}$ = 6.4 Hz, $^3J_{6a,5}$ = 5.0 Hz, H-5), 4.18 (1H, dd, $^2J_{6b,6a}$ = 11.4 Hz, $^3J_{6b,5}$ = 6.3 Hz, H-6b), 4.09–4.15 (2H, m, H-6a, H-2), 4.97 (1H, ddd, $^2J_{3,F}$ = 46.9 Hz, $^3J_{3,2}$ = 10.6

Hz, ${}^3J_{4,3}$ = 3.6 Hz, H-3), 5.57 (1H, ddd, ${}^3J_{4,5}$ = 1.2 Hz, ${}^3J_{4,3}$ = 3.7 Hz, ${}^3J_{4,F}$ = 5.3 Hz, H-4), 5.70 (1H, d, ${}^3J_{2,NH}$ = 8.3 Hz, N*H*), 5.91 (1H, d, ${}^2J_{2,1}$ = 8.6 Hz, H-1); ^{13}C {^{1}H} NMR (125 MHz, $CDCl_3$) δ 20.6, 20.7, 20.9 (3 × s, CO*C*H_3), 23.4 (s, NHCO*C*H_3), 51.9 (d, ${}^2J_{C,F}$ = 19.7 Hz, C-2), 61.3 (d, ${}^4J_{C,F}$ = 2.7 Hz, C-6), 66.3 (d, ${}^2J_{C,F}$ = 16.6 Hz, C-4), 70.9 (d, ${}^3J_{C,F}$ = 6.1 Hz, C-5), 87.4 (d, ${}^1J_{C,F}$ = 193.0 Hz, C-3), 91.5 (d, ${}^3J_{C,F}$ = 10.7 Hz, C-1), 169.2, 169.9, 171.4, 171.7 (4 × s, *C*OCH_3); ^{19}F NMR (470 MHz, $CDCl_3$) δ −198.23 (ddd, ${}^2J_{3,F}$ = 46.8, ${}^3J_{2,F}$ = 10.3 Hz, ${}^3J_{4,F}$ = 5.5 Hz). HRMS (ESI): *m/z* $[M + Na]^+$ calcd for $C_{14}H_{20}FNaNO_8$: 372.1065; found: 372.1064. Continued elution afforded the α-anomer **6α** [6] (135 mg, 60%); mp 177–178 °C; $[\alpha]^{20}_{D}$ +148 (*c* 0.20, $CHCl_3$); ^{1}H NMR (300 MHz, $CDCl_3$) δ 2.01 (3H, s, NHCOC*H*$_3$), 2.04, 2.15, 2.17 (3 × 3H, 3 × s, COC*H*$_3$), 4.06 –4.19 (3H, m, H-5, H-6), 4.74 (2H, overlap 2 × ddd, ${}^3J_{3,2}$ = 11.1 Hz, ${}^3J_{4,3}$ = 3.4 Hz, H-3, H-2), 5.47 (1H, d, ${}^3J_{2,NH}$ = 8.7 Hz, NH), 5.62 – 5.63 (1H, m, H-4), 6.25 (1H, dd, ${}^2J_{2,1}$ = 3.9 Hz, ${}^4J_{1,3}$ = 3.7 Hz, H-1); ^{13}C {^{1}H} NMR (75 MHz, $CDCl_3$): δ 20.3, 20.3, 20.5 (3 × s, CO*C*H_3), 22.9 (s, NHCO*C*H_3), 47.5 (d, ${}^2J_{C,F}$ = 19.2 Hz, C-2), 61.2 (d, ${}^4J_{C,F}$ = 2.1 Hz, C-6), 66.4 (d, ${}^2J_{C,F}$ = 16.9 Hz, C-4), 68.4 (d, ${}^3J_{C,F}$ = 5.5 Hz, C-5), 86.4 (d, ${}^1J_{C,F}$ = 192.1 Hz, C-3), 91.6 (d, ${}^3J_{C,F}$ = 9.3 Hz, C-1), 169.1, 170.5, 170.7, 170.9 (4 × s, *C*OCH_3); ^{19}F NMR (282 MHz, $CDCl_3$): δ −202.53 (ddd, ${}^2J_{3,F}$ = 46.7 Hz, ${}^3J_{2,F}$ = 12.4 Hz, ${}^3J_{4,F}$ = 5.2 Hz). HRMS (ESI): *m/z* $[M + Na]^+$ calcd for $C_{14}H_{20}FNO_8Na$: 372.1071; found: 372.1065. Anal. Calcd for $C_{14}H_{20}FNO_8$: C, 48.14; H, 5.77; N, 4.01. Found: C, 47.92; H, 5.58; N, 3.80.

1,3,6-Tri-*O*-acetyl-2-azido-2,4-dideoxy-4-fluoro-D-glucopyranose (44)
and 1,6-anhydro-3-*O*-acetyl-2-azido-2,4-dideoxy-4-fluoro-β-D-glucopyranose (45)

Azide **26** (363 mg, 1.92 mmol) was acetolysed in Ac_2O/TESOTf solution (2.0 mL) for 4 h following the general procedure. The reaction was monitored by GC/MS. Chromatography in S12 gave **44** (497 mg, 78%) as a chromatographically inseparable mixture of anomers (α/β 67:33) which was used in the next reaction. Recrystallization from methanol (2×) afforded the α-anomer **44α** in 98% purity (by ^{19}F NMR) for characterization (163 mg, 26%); mp 74–77 °C; $[\alpha]^{20}_{D}$ +66 (*c* 0.25, $CHCl_3$); ^{1}H NMR (300 MHz, $CDCl_3$) δ 2.09, 2.19, 2.21 (3 × 3H, 3 × s, COC*H*$_3$), 3.53 (1H, ddd, ${}^4J_{2,F}$ = 1.0 Hz,

$^3J_{1,2}$ = 3.7 Hz, $^3J_{2,3}$ = 10.7 Hz, H-2), 4.11 (1H, dddd, $^3J_{6b,5}$ = 4.3 Hz, $^3J_{5,F}$ = 3.4 Hz, $^3J_{4,5}$ = 10.0 Hz, $^3J_{6a,5}$ = 2.3 Hz, H-5), 4.26 (1H, ddd, $^2J_{6a,6b}$ = 12.4 Hz, $^3J_{6b,5}$ = 4.3 Hz, $^4J_{6b,F}$ = 1.7 Hz, H-6b), 4.35 (1H, ddd, $^2J_{6a,6b}$= 12.4 Hz, $^3J_{6a,5}$ = 2.3 Hz, $^4J_{6a,F}$ = 2.3 Hz, H-6a), 4.50 (1H, ddd, $^2J_{4,F}$ = 50.2 Hz, $^3J_{4,5}$ = 10.0 Hz, $^3J_{4,3}$ = 8.9 Hz, H-4), 5.59 (1H, ddd, $^3J_{3,F}$ = 12.9 Hz, $^3J_{3,2}$ = 10.7 Hz, $^3J_{4,3}$ = 8.9 Hz, H-3), 6.27 (1H, t, $^3J_{2,1}$ = 3.7 Hz, $^5J_{1,F}$ = 3.7 Hz, H-1); ^{13}C {^{1}H} NMR (75 MHz, $CDCl_3$) δ 20.66, 20.71, 20.9 (3 × s, CO*C*H_3), 60.0 (d, $^3J_{C,F}$ = 6.8 Hz, C-2), 61.6 (s, C-6), 69.2 (d, $^2J_{C,F}$ = 27.7 Hz, C-5), 70.0 (d, $^2J_{C,F}$ = 19.4 Hz, C-3), 86.5 (d, $^1J_{C,F}$ = 188.7 Hz, C-4), 89.8 (d, $^4J_{C,F}$ = 1.5 Hz, C-1), 168.4, 169.7, 170.4 (3 × s, *C*OCH_3); ^{19}F NMR (282 MHz, $CDCl_3$) δ −199.18 (bdd, $^2J_{F,4}$ = 50.3 Hz, $^3J_{F,3}$ = 13.1 Hz). Anal. Calcd for $C_{12}H_{16}FN_3O_7$: C, 43.25; H, 4.84; N, 12.61. Found: C, 43.24; H, 4.72; N, 12.49. NMR data for the *β*-anomer **44β**: ^{1}H NMR (300 MHz, $CDCl_3$) δ 2.09, 2.18 (3 × 3H, 3 × s, COC*H*$_3$), 3.59 (1H, ddd, $^4J_{2,F}$ = 1.0 Hz, $^3J_{1,2}$ = 8.5 Hz, $^3J_{2,3}$ = 10.4 Hz, H-2), 3.84 (1H, dddd, $^3J_{6b,5}$ = 5.0 Hz, $^3J_{5,F}$ = 2.5 Hz, $^3J_{4,5}$ = 9.9 Hz, $^3J_{6a,5}$ = 2.5 Hz, H-5), 4.33 (2H, m, H-6), 4.44 (1H, ddd, $^2J_{4,F}$ = 49.9 Hz, $^3J_{4,5}$ = 9.9 Hz, $^3J_{4,3}$ = 9.0 Hz, H-4), 5.23 (1H, ddd, $^3J_{3,F}$ = 13.6 Hz, $^3J_{3,2}$ = 10.4 Hz, $^3J_{4,3}$ = 9.0 Hz, H-3), 5.57 (1H, d, $^3J_{2,1}$ = 8.5 Hz, H-1); ^{13}C NMR (75 MHz, $CDCl_3$) δ 20.7, 20.8 (2 × s, 3 × CO*C*H_3), 62.3 (d, $^3J_{C,F}$ = 7.6 Hz, C-2), 61.6 (s, C-6), 71.9 (d, $^2J_{C,F}$ = 20.0 Hz, C-5), 72.1 (d, $^2J_{C,F}$ = 24.0 Hz, C-3), 86.3 (d, $^1J_{C,F}$ = 188.7 Hz, C-4), 92.5 (d, $^4J_{C,F}$ = 1.5 Hz, C-1), 168.4, 169.3, 170.4 (3 × s, *C*OCH_3); ^{19}F NMR (282 MHz, $CDCl_3$) δ −201.29 (m). A continued elution gave **45** (70 mg, 16%), mp 58–63 °C; $[\alpha]_D^{20}$ +26 (*c* 0.22, $CHCl_3$); ^{1}H NMR (300 MHz, $CDCl_3$) δ 2.13 (3H, s, COC*H*$_3$), 3.22 (1H, bs, H-2), 3.83 (1H, ddd, $^2J_{6en,6ex}$ = 7.9 Hz, $^3J_{6ex,5}$ = 5.9 Hz, $^4J_{6ex,F}$ = 4.6 Hz, H-6*ex*), 4.03 (1H, dt, $^2J_{6en,6ex}$= 7.9 Hz, $^3J_{6en,5}$ = 1.1 Hz, $^4J_{6en,F}$ = 1.1 Hz, H-6*en*), 4.42 (1H, dddd, $^2J_{4,F}$ = 44.5 Hz, $^3J_{4,5}$ = 1.8 Hz, $^3J_{4,3}$ = 1.8 Hz, $^4J_{4,2}$ = 0.8 Hz, H-4), 4.77 (1H, ddddd, $^3J_{5,6ex}$ = 5.8 Hz, $^3J_{5,F}$ = 10.7 Hz, $^3J_{4,5}$ = 1.8 Hz, $^3J_{5,6en}$ = 0.9 Hz, $^4J_{5,3}$ = 0.8 Hz, H-5), 5.06 (1H, ddddd, $^3J_{3,F}$ = 16.4 Hz, $^3J_{3,2}$ = 1.6 Hz, $^3J_{4,3}$ = 1.6 Hz, $^4J_{3,1}$ = 1.6 Hz, $^4J_{3,5}$ = 0.8 Hz, H-3), 5.52 (1H, dd, $^3J_{1,2}$ = 1.6 Hz, $^4J_{1,3}$ = 1.6 Hz, H-1); ^{13}C {^{1}H} NMR (75 MHz, $CDCl_3$) δ 20.8 (s, CO*C*H_3), 58.7 (d, $^3J_{C,F}$ = 2.3 Hz, C-2), 64.0 (d, $^3J_{C,F}$ = 8.9 Hz, C-6), 69.5 (d, $^2J_{C,F}$ = 34.2 Hz, C-3), 73.6 (d, $^2J_{C,F}$ = 21.3 Hz, C-5), 86.6 (d, $^1J_{C,F}$ = 182.5 Hz, C-4), 100.2 (s, C-1), 169.1 (s, *C*OCH_3); ^{19}F NMR (282 MHz, $CDCl_3$) δ −184.30 (dddd, $^2J_{F,4}$ = 44.6 Hz, $^3J_{F,3}$ = 15.9 Hz, $^3J_{F,5}$ = 10.4 Hz, $^4J_{F,6ex}$ = 4.6 Hz). HRMS (ESI): *m/z* [M + Na]$^+$ calcd for $C_8H_{10}FN_3NaO_8$: 254.0547; found: 254.0549.

2-Acetamido-1,3,6-tri-*O*-acetyl-2,4-dideoxy-4-fluoro-D-glucopyranose (1)

Starting azide **44** (287 mg, 0.86 mmol) was hydrogenated in ethanol (10 mL) containing Ac_2O (1 mL) and 10% Pd/C (100 mg) for 2 h following the general procedure. The reaction was monitored by TLC in S12. Chromatography in ethyl acetate gave amide **1** (260 mg, 86%, α/β 77:23) as a foam. In another experiment, the α-anomer **44α** (141 mg, 0.42 mmol) was hydrogenated to provide the α-anomer **1α** (119 mg, 80%) for characterization; $[\alpha]_D^{20}$ +26 (*c* 0.23, $CHCl_3$); ^{1}H NMR (300 MHz, $CDCl_3$) comparable to the reported data [8]; ^{13}C {^{1}H} NMR (75 MHz, $CDCl_3$) δ 20.7, 20.8, 20.9, 23.00 (4 × s, CO*C*H_3), 50.9 (d, $^3J_{C,F}$ = 7.1 Hz, C-2), 61.60 (s, C-6), 69.1 (d, $^2J_{C,F}$ = 23.8 Hz, C-5), 70.6 (d, $^2J_{C,F}$ = 18.9 Hz, C-3), 86.0 (d, $^1J_{C,F}$ = 186.8 Hz, C-4), 90.4 (d, $^4J_{C,F}$ = 1.6 Hz, C-1), 168.6, 170.1, 171.5, 171.6 (4 × s, *C*OCH_3); ^{19}F NMR (282 MHz, $CDCl_3$) δ −197.60 (bdd, $^2J_{F,4}$ = 50.3 Hz, $^3J_{F,3}$ = 13.7 Hz). HRMS (ESI): *m/z* $[M + Na]^+$ calcd for $C_{14}H_{20}FNNaO_8$: 372.1065; found: 372.1063. Anal. Calcd for $C_{14}H_{20}FNO_8$: C, 48.14; H, 5.77; N, 4.01. Found: C, 48.39; H, 5.89; N, 3.85. NMR data for β-anomer **1β**: ^{1}H NMR (500 MHz, $CDCl_3$) agrees with the reported data [9]. ^{13}C {^{1}H} NMR (125 MHz, $CDCl_3$): δ 20.7, 20.8, 20.9, 23.1 (4 × s, CO*C*H_3), 52.7 (d, $^3J_{C,F}$ = 7.0 Hz, C-2), 61.7 (s, C-6), 72.2 (d, $^2J_{C,F}$ = 24.0 Hz, C-5), 72.4 (d, $^2J_{C,F}$ = 19.0 Hz, C-3), 86.0 (d, $^1J_{C,F}$ = 187.6 Hz, C-4), 92.5 (d, $^4J_{C,F}$ = 1.4 Hz, C-1), 169.4, 170.2, 171.1, 171.6 (4 × s, *C*OCH_3). ^{19}F NMR (470 MHz, $CDCl_3$): δ −199.47 (bdd, $^3J_{3,F}$ = 14.1 Hz, $^2J_{4,F}$ = 50.6 Hz).

2-Acetamido-1,3,6-tri-*O*-acetyl-2,4-dideoxy-4-fluoro-D-galactopyranose (4)

Azide **31** (148 mg, 0.78 mmol) was acetolysed in Ac_2O/TESOTf solution (1.5 mL) for 4 h following the general procedure. The reaction was monitored by GC/MS. Chromatography in S12 gave 1,3,6-tri-*O*-acetyl-2-azido-2,4-dideoxy-4-fluoro-D-galactopyranose (**46**, 246 mg, 94%) as an inseparable mixture of anomers (α/β 79:21) characterized by NMR spectra. Data for the α-anomer **46α**: ^{1}H NMR (300 MHz, $CDCl_3$) δ 2.07, 2.16, 2.21 (3 × 3H, 3 × s, COC*H*$_3$), 4.02 (1H, dd, $^3J_{2,1}$ = 3.6 Hz, $^3J_{3,2}$ = 11.0 Hz, H-2) 4.22 (3H, m, H-5, H-6), 4.95 (1H, dd, $^2J_{4,F}$ = 50.4 Hz, $^3J_{4,3}$ = 2.5 Hz, H-4), 5.23 (1H, ddd, $^3J_{3,F}$ = 26.3 Hz, $^3J_{3,2}$ = 11.0 Hz, $^3J_{4,3}$ = 2.5 Hz, H-3), 6.33 (1H, d, $^3J_{2,1}$ = 3.6 Hz, H-1);

^{13}C NMR (75 MHz, $CDCl_3$) δ 20.7, 20.8, 20.9 (3 × s, COCH_3), 56.6 (d, $^3J_{C,F}$ = 2.4 Hz, C-2), 61.2 (d, $^3J_{C,F}$ = 5.9 Hz, C-6), 68.9 (d, $^2J_{C,F}$ = 18.3 Hz, C-5), 69.3 (d, $^2J_{C,F}$ = 17.7 Hz, C-3), 85.8 (d, $^1J_{C,F}$ = 185.7 Hz, C-4), 90.2 (s, C-1), 168.5, 170.2, 170.3 (3 × s, $COCH_3$); ^{19}F NMR (282 MHz, $CDCl_3$) δ −220.04 (ddd, $^3J_{3,F}$ = 26.4 Hz, $^3J_{5,F}$ = 27.4 Hz, $^2J_{4,F}$ = 50.5 Hz). Data for the β-anomer **46β**: ^{1}H NMR (300 MHz, $CDCl_3$) δ 2.07, 2.20 (3 × 3H, 2 × s, COCH_3), 3.90 (2H, m, H-2, H-5), 4.22 (1H, dd, $^3J_{5,6a}$ = 6.3 Hz, $^2J_{6a,6b}$ = 11.5 Hz, H-6a), 4.29 (1H, ddd, $^3J_{5,6a}$ = 6.7 Hz, $^2J_{6a,6b}$ = 11.5 Hz, $^4J_{6b,F}$ = 1.1 Hz, H-6b), 4.79 (1H, ddd, $^3J_{3,F}$ = 26.5 Hz, $^3J_{3,2}$ = 10.9 Hz, $^3J_{4,3}$ = 2.6 Hz, H-3), 4.87 (1H, dd, $^2J_{4,F}$ = 50.0 Hz, $^3J_{4,3}$ = 2.6 Hz, H-4), 5.53 (1H, dd, $^5J_{1,F}$ = 1.0 Hz, $^3J_{2,1}$ = 8.5 Hz, H-1); ^{13}C NMR (75 MHz, $CDCl_3$) δ 20.67, 20.71, 20.9 (3 × s, COCH_3), 59.3 (s, C-2), 61.1 (d, $^3J_{C,F}$ = 5.7 Hz, C-6), 71.7 (d, $^2J_{C,F}$ = 17.7 Hz, C-3), 71.8 (d, $^2J_{C,F}$ = 18.1 Hz, C-5), 84.9 (d, $^1J_{C,F}$ = 186.2 Hz, C-4), 92.6 (s, C-1), 168.6, 169.9, 170.3 (3 × s, $COCH_3$); ^{19}F NMR (282 MHz, $CDCl_3$) δ −218.11 (ddd, $^3J_{3,F}$ = 26.5 Hz, $^3J_{5,F}$ = 24.0 Hz, $^2J_{4,F}$ = 50.0 Hz). The product **46** (242 mg, 0.73 mmol) was hydrogenated in ethanol (12 mL) containing Ac_2O (1.5 mL) and 10% Pd/C (100 mg) for 3 h following the general procedure. The reaction was monitored by TLC in S12 and S1. Chromatography in dichloromethane/ethanol 25:1gave first the α-anomer **4α** (169 mg, 67%); mp 143–144 °C (ethyl acetate/petroleum ether, ref. [9] 140–142 °C); $[\alpha]_D^{20}$ +117 (*c* 0.27, $CHCl_3$, ref. [9] $[\alpha]_D$ +114 (*c* 1, $CHCl_3$); ^{1}H NMR (300 MHz, $CDCl_3$) δ 1.94, 2.07, 2.14, 2.17 (4 × 3H, 4 × s, COCH_3), 4.10 (1H,dt, $^3J_{5,6b}$ = 6.5 Hz, $^3J_{5,6a}$ = 6.5 Hz, $^3J_{5,F}$ = 27.2 Hz, H-5), 4.21 (1H, dd, $^2J_{6b,6a}$ = 11.5 Hz, $^3J_{6a,5}$ = 6.5 Hz, H-6a), 4.29 (1H, ddd, $^2J_{6b,6a}$ = 11.6 Hz, $^3J_{6b,5}$ = 6.5 Hz, $^4J_{6b,F}$ = 1.2 Hz, H-6b), 4.78 (1H, ddd, $^3J_{3,2}$ = 11.6 Hz, $^3J_{2,1}$ = 3.6 Hz, $^3J_{2,NH}$ = 9.2 Hz, H-2), 4.87 (1H, dd, $^2J_{4,F}$ = 49.9 Hz, $^3J_{4,3}$ = 2.4 Hz, H-4), 5.18 (1H, ddd, $^3J_{3,F}$ = 26.9 Hz, $^3J_{3,2}$ = 11.6 Hz, $^3J_{4,3}$ = 2.4 Hz, H-3), 5.50 (1H, d, $^3J_{2,NH}$ = 9.2 Hz, NH), 6.20 (1H, d, $^3J_{2,1}$ = 3.6 Hz, H-1); ^{13}C {^{1}H} NMR (75 MHz, $CDCl_3$) δ 20.7, 20.8, 20.9, 23.1 (4 × s, COCH_3), 46.9 (d, $^3J_{C,F}$ = 3.1 Hz, C-2), 61.4 (d, $^3J_{C,F}$ = 6.1 Hz, C-6), 68.1 (d, $^2J_{C,F}$ = 18.0 Hz, C-3), 68.9 (d, $^2J_{C,F}$ = 18.4 Hz, C-5), 85.8 (d, $^1J_{C,F}$ = 187.4 Hz, C-4), 91.2 (s, C-1), 168.7, 170.0, 170.4, 171.5 (4 ×s, $COCH_3$); ^{19}F NMR (282 MHz, $CDCl_3$) δ −218.70 (ddd, $^3J_{3,F}$ = 27.0 Hz, $^3J_{5,F}$ = 27.3 Hz, $^2J_{4,F}$ = 50.0 Hz). HRMS (ESI): *m/z* $[M + H]^+$ calcd for $C_{14}H_{21}FNO_8$: 350.1246; found: 350.1241. Anal. Calcd for $C_{14}H_{20}FNO_8$: C, 48.14; H, 5.77; N, 4.01. Found: C, 48.00; H, 5.61; N, 3.88. Continued chromatography furnished β-anomer **4β** (28 mg, 11%); mp 214 °C (ethanol/ethyl acetate); $[\alpha]_D^{20}$ +19 (*c*

0.17, $CHCl_3$); ^{1}H NMR (300 MHz, $CDCl_3$) δ 1.94, 2.08, 2.12, 2.13 (4 × 3H, 4 × s, COCH_3), 3.95 (1H, dt, $^3J_{5,F}$ = 26.2 Hz, $^3J_{5,6b}$ = 6.7 Hz, $^3J_{5,6a}$ = 6.2 Hz, H-5), 4.27 (1H, dd, $^2J_{6a,6b}$ = 11.5 Hz, $^3J_{6a,5}$ = 6.2 Hz, H-6a), 4.31 (1H, dd, $^2J_{6a,6b}$ = 11.5 Hz, $^3J_{6b,5}$ = 6.7 Hz, H-6b), 4.37 (1H, ddd, $^3J_{3,2}$ = 11.3 Hz, $^3J_{2,1}$ = 8.8 Hz, $^3J_{2,NH}$ = 9.4 Hz, H-2), 4.82 (1H, ddd, $^2J_{4,F}$ = 50.1 Hz, $^3J_{4,5}$ = 1.3 Hz, $^3J_{4,3}$ = 2.4 Hz, H-4), 5.17 (1H, ddd, $^3J_{3,F}$ = 27.2 Hz, $^3J_{3,2}$ = 11.3 Hz, $^3J_{4,3}$ = 2.4 Hz, H-3), 5.50 (1H, d, $^3J_{2,NH}$ = 9.4 Hz, NH), 5.80 (1H, d, $^3J_{2,1}$ = 8.8 Hz, H-1); ^{13}C {^{1}H} NMR (75 MHz, $CDCl_3$): δ 20.7, 20.9, 23.3 (3 × s, 4 × COCH_3), 49.9 (d, $^3J_{C,F}$ = 1.4 Hz, C-2), 61.4 (d, $^3J_{C,F}$ = 5.6 Hz, C-6), 70.2 (d, $^2J_{C,F}$ = 18.0 Hz, C-3), 71.9 (d, $^2J_{C,F}$ = 18.2 Hz, C-5), 85.4 (d, $^1J_{C,F}$ = 187.2 Hz, C-4), 92.5 (s, C-1), 169.5, 170.2, 170.4, 171.8 (4 × s, *C*OCH_3); ^{19}F NMR (282 MHz, $CDCl_3$): δ −217.45 (ddd, $^3J_{3,F}$ = 27.2 Hz, $^3J_{5,F}$ = 26.6 Hz, $^2J_{4,F}$ = 50.1 Hz). HRMS (ESI): *m/z* $[M + H]^+$ calcd for $C_{14}H_{21}FNO_8$: 350.1246; found: 350.1245. Anal. Calcd for $C_{14}H_{20}FNO_8$: C, 48.14; H, 5.77; N, 4.01. Found: C, 48.25; H, 5.70; N, 3.92.

2-Acetamido-1,6-di-*O*-acetyl-2,3,4-trideoxy-3,4-difluoro-α-D-glucopyranose (7)

Difluoro derivative **28** (100 mg, 0.52 mmol) was acetolysed in Ac_2O/TESOTf solution (1.5 mL) for 6 h following the general procedure. The reaction was monitored by GC/MS and TLC in S14. Chromatography in S14 gave first 1,6-di-*O*-acetyl-2-azido-2,3,4-trideoxy-3,4-difluoro-D-glucopyranose (**47**, 44 mg, 29%) as a mixture enriched in β-anomer (β/α = 93:7), further elution gave a mixture enriched in α-anomer **47α** (α/β = 96:4, 94 mg, 61%,) contaminated by chromatographically inseparable impurities (ca 5%, ^{1}H NMR). This fraction was taken over into the next reaction without further purification. Data for α-anomer **47α** ^{1}H NMR (500 MHz, $CDCl_3$): δ 2.10, 2.18 (2 x 3H, 2 x s, COCH_3), 3.74 (1H, dddd, $^3J_{2,1}$ = 3.8 Hz, $^3J_{2,3}$ = 9.9 Hz, $^3J_{2,F3}$ = 11.5 Hz, $^4J_{2,F4}$ = 0.9 Hz, H-2), 4.05 (1H, dddd, $^3J_{5,6a}$ = 4.4 Hz, $^3J_{5,6b}$ = 2.4 Hz, $^3J_{5,F4}$ = 4.8 Hz, $^3J_{5,4}$ = 10.0 Hz, H-5), 4.27 (1H, ddd, $^3J_{5,6a}$ = 4.4 Hz, $^2J_{6a,6b}$ = 12.5 Hz, $^4J_{6a,F4}$ = 1.7 Hz, H-6a), 4.37 (1H, dddd, $^3J_{5,6b}$ = 2.3 Hz, $^2J_{6a,6b}$ = 12.5 Hz, $^4J_{6b,F4}$ = 1.9 Hz, $^5J_{6b,F3}$ = 1.9 Hz, H-6b), 4.65 (1H, dddd, $^2J_{4,F4}$ = 50.9 Hz, $^3J_{4,F3}$ = 14.3 Hz, $^3J_{4,3}$ = 8.3 Hz, $^3J_{4,5}$ = 10.1 Hz, H-4), 4.95 (1H, dddd, $^2J_{3,F3}$ = 53.4 Hz, $^3J_{3,F4}$ = 15.0 Hz, $^3J_{3,4}$ = 8.3 Hz, $^3J_{3,2}$ = 9.9 Hz, H-3), 6.25 (1H, ddd, $^3J_{2,1}$ = 3.8 Hz, $^4J_{1,F3}$ = 3.5 Hz, $^5J_{1,F4}$ = 3.5 Hz, H-1). ^{13}C {^{1}H} NMR (125 MHz, $CDCl_3$): δ 20.7, 20.8 (2 x s, 2 x COCH_3), 60.6 (dd, $^2J_{C,F3}$ = 17.7 Hz, $^3J_{C,F4}$ = 6.8 Hz, C-2), 61.4 (s, C-6), 68.9

(dd, $^2J_{C,F4}$ = 24.0 Hz, $^3J_{C,F3}$ = 6.7 Hz, C-5), 86.5 (dd, $^1J_{C,F4}$ = 187.7 Hz, $^2J_{C,F3}$ = 18.6 Hz, C-4), 89.7 (dd, $^3J_{C,F3}$ = 9.3 Hz, $^4J_{C,F4}$ = 1.2 Hz, C-1), 90.5 (dd, $^1J_{C,F3}$ = 188.4 Hz, $^2J_{C,F4}$ = 19.6 Hz, C-3), 168.2, 170.4 (2 x s, 2 x *C*OCH$_3$). ^{19}F NMR (470 MHz, CDCl$_3$): δ −198.70 (1F, ddddddd, $^2J_{F4,4}$ = 51.0 Hz, $^3J_{F4,F3}$ = 13.3 Hz, $^3J_{F4,3}$ = 15.0 Hz, $^5J_{F4,1}$ = 3.5 Hz, $^4J_{F4,6b}$ = 1.9 Hz, $^3J_{F4,5}$ = 4.8 Hz, $^4J_{F4,2}$ = 0.9 Hz, F-4), −195.94 (1F, dddddd, $^2J_{F3,3}$ = 53.2 Hz, $^3J_{F3,F4}$ = 13.3 Hz, $^3J_{F3,4}$ = 14.5 Hz, $^3J_{F3,2}$ = 11.6 Hz, $^4J_{F3,1}$ = 3.5 Hz, $^5J_{F3,6b}$ = 1.9 Hz, F-3). Hydrogenation of **47α** (84 mg, 0.17 mmol) in ethanol (4 mL) containing Ac$_2$O (0.8 mL) and 10% Pd/C (55 mg) for 4 h was carried out following the general procedure. The reaction was monitored by TLC in S12. Chromatography in ethyl acetate gave acetamide **7** (71 mg, 44% from **28**); mp 151–154 °C (ethyl acetate-heptane); $[\alpha]_D^{20}$ +146 (*c* 0.27, CHCl$_3$); ^{1}H {^{19}F} NMR (500 MHz, CDCl$_3$) δ 2.04, 2.11, 2.17 (3 × 3H, 3 × s, COC*H*$_3$), 4.00 (1H, dddd, $^3J_{5,4}$ = 9.8 Hz,$^3J_{5,6b}$ = 4.2 Hz, $^3J_{5,6a}$ = 2.4 Hz, $^4J_{5,1}$ = 0.6 Hz, H-5), 4.25 (1H, dd, $^2J_{6b,6a}$ = 12.4 Hz, $^3J_{5,6b}$ = 4.2 Hz, H-6b), 4.38 (1H, dd, $^2J_{6b,6a}$ = 12.4 Hz, $^3J_{5,6a}$ = 2.4 Hz, H-6a), 4.55 (1H, ddd, $^3J_{2,1}$ = 3.7 Hz, $^3J_{2,3}$ = 10.5 Hz, $^3J_{2,NH}$ = 8.8 Hz, H-2), 4.68 (1H, dd, $^3J_{4,3}$ = 8.2 Hz, $^3J_{4,5}$ = 9.8 Hz, H-4), 4.73 (1H, dd, $^3J_{3,4}$ = 8.2 Hz, $^3J_{3,2}$ = 10.5 Hz, H-3), 5.64 (1H, d, $^3J_{NH,2}$ = 8.8 Hz, NH), 6.15 (1H, d, $^3J_{2,1}$ = 3.7 Hz, H-1); The following H-F couplings were extracted from ^{1}H NMR spectrum (without ^{19}F decoupling): $^4J_{6b,F4}$ = 1.6 Hz, $^4J_{1,F3}$ = 3.4 Hz and $^5J_{1,F4}$ = 3.4 Hz. ^{13}C {^{1}H} NMR (125 MHz, CDCl$_3$) δ 20.7, 20.8, 23.1 (3 × s, CO*C*H$_3$), 50.4 (dd, $^2J_{C,F3}$ = 18.4 Hz, $^3J_{C,F4}$ = 7.5 Hz, C-2), 61.4 (s, C-6), 68.8 (dd, $^2J_{C,F4}$ = 23.9 Hz, $^3J_{C,F3}$ = 6.8 Hz, C-5), 86.8 (dd, $^1J_{C,F4}$ = 186.3 Hz, $^2J_{C,F3}$ = 18.4 Hz, C-4), 89.6 (dd, $^1J_{C,F3}$ = 189.5 Hz, $^2J_{C,F4}$ = 19.0 Hz, C-3), 90.7 (dd, $^3J_{C,F3}$ = 9.2 Hz, $^4J_{C,F4}$ = 1.3 Hz, C-1), 168.3, 170.2, 170.5 (3 × s, *C*OCH$_3$); ^{19}F NMR (470 MHz, CDCl$_3$) δ −198.80 (1F, dtdd, $^2J_{F3,3}$ = 53.0 Hz, $^3J_{F3,2}$ = 13.8 Hz, $^3J_{F3,4}$ = 13.8 Hz, $^4J_{F3,1}$ = 3.4 Hz, $^3J_{F3,F4}$ = 13.7 Hz, F-3), −197.80 (1F, dddddd, $^2J_{F4,4}$ = 49.9 Hz, $^3J_{F4,3}$ = 14.1 Hz, $^3J_{F4,5}$ = 4.8 Hz, $^4J_{F4,1}$ = 3.4 Hz, $^4J_{F4,6b}$ = 1.6 Hz, $^3J_{F4,F3}$ = 13.7 Hz, F-4). HRMS (APCI): *m/z* [M + H]$^+$ calcd for $C_{12}H_{18}F_2NO_6$: 310.1097; found: 310.1096. Anal. Calcd for $C_{12}H_{17}F_2NO_6$: C, 46.60; H, 5.54; N, 4.53. Found: C, 46.57; H, 5.69; N, 4.38.

1,6-Di-*O*-acetyl-2-azido-2,3,4-trideoxy-3,4-difluoro-D-galactopyranose (48)

Difluoro derivative **22** (88 mg, 0.46 mmol) was acetolysed in Ac_2O/TESOTf solution (1.5 mL) for 9 h following the general procedure. The reaction was monitored by GC/MS and TLC in S12. Chromatography in S12 gave first the β-anomer **48β** (28 mg, 21%) as a colorless oil. ^{1}H NMR (500 MHz, $CDCl_3$) δ 2.09, 2.21 (2 × 3H, 2 × s, COC*H*$_3$), 3.82 (1H, dtd, $^3J_{5,6b}$ = 6.6 Hz, $^3J_{5,6a}$ = 6.3 Hz, $^3J_{5,F4}$ = 25.8 Hz, $^4J_{5,F3}$ = 1.9 Hz, H-5), 3.99 (1H, tdd, $^3J_{2,1}$ = 8.4 Hz, $^3J_{2,3}$ = 10.2 Hz, $^3J_{2,F3}$ = 11.3 Hz, $^4J_{2,F4}$ = 1.0 Hz, H-2), 4.27 (1H, dd, $^2J_{6a,6b}$ = 11.5 Hz, $^3J_{6a,5}$ = 6.3 Hz, H-6a), 4.32 (1H, ddd, $^2J_{6b,6a}$ = 11.5 Hz, $^3J_{6b,5}$ = 6.6 Hz, $^4J_{6b,F4}$ = 1.7 Hz, H-6b), 4.49 (1H, dddd, $^3J_{3,F4}$ = 25.9 Hz, $^3J_{3,4}$ = 2.7 Hz, $^2J_{3,F3}$ = 46.1 Hz, $^3J_{3,2}$ = 10.2 Hz, H-3), 4.93 (1H, ddd, $^2J_{4,F4}$ = 50.4 Hz, $^3J_{4,3}$ = 2.7 Hz, $^3J_{4,F3}$ = 6.6 Hz, H-4), 5.56 (1H, bd, $^3J_{2,1}$ = 8.4 Hz, H-1); ^{13}C {^{1}H} NMR (125 MHz, $CDCl_3$) δ 20.7, 20.8 (2 × s, CO*C*H$_3$), 60.4 (dd, $^2J_{C,F3}$ = 18.9 Hz, $^3J_{C,F4}$ = 0.9 Hz, C-2), 61.1 (dd, $^3J_{C,F4}$ = 5.8 Hz, $^4J_{C,F3}$ = 2.7 Hz, C-6), 71.1 (dd, $^2J_{C,F4}$ = 18.1 Hz, $^3J_{C,F3}$ = 6.2 Hz, C-5), 84.9 (dd, $^1J_{C,F4}$ = 187.7 Hz, $^2J_{C,F3}$ = 16.5 Hz, C-4), 89.4 (dd, $^1J_{C,F3}$ = 194.9 Hz, $^2J_{C,F4}$ = 18.1 Hz, C-3), 92.0 (d, $^3J_{C,F3}$ = 10.7 Hz, C-1), 168.6, 170.3 (2 × s, *C*OCH$_3$); ^{19}F NMR (470 MHz, $CDCl_3$) δ −218.05 (1F, dtd, $^2J_{F4,4}$ = 50.4 Hz, $^3J_{F4,3}$ = 25.8 Hz, $^3J_{F4,5}$ = 25.8 Hz, $^3J_{F4,F3}$ = 15.2 Hz, F-4), −197.23 (1F, dddd, $^2J_{F3,3}$ = 46.0 Hz, $^3J_{F3,4}$ = 6.6 Hz, $^3J_{F3,2}$ = 11.3 Hz, $^3J_{F3,F4}$ = 15.2 Hz, F-3). HRMS (APCI): *m/z* [M + H – N_2]$^+$ calcd for $C_{10}H_{14}F_2NO_5$: 266.0835; found: 266.0835. A continued elution gave the α-anomer (**48α**, 88 mg, 65%) as a colorless oil, $[\alpha]_D^{20}$ +76 (*c* 0.19, $CHCl_3$); ^{1}H NMR (300 MHz, $CDCl_3$) δ 2.09, 2.15 (2 × 3H, 2 × s, COC*H*$_3$), 4.03–4.17 (2H, m, H-5, H-2), 4.23 (1H, dd, $^3J_{5,6a}$ = 6.3 Hz, $^2J_{6b,6a}$ = 11.2 Hz, H-6a), 4.31 (1H, ddt, $^3J_{5,6b}$ = 6.7 Hz, $^2J_{6b,6a}$ = 11.3 Hz, $^4J_{6b,F4}$ = 1.3 Hz, $^5J_{6b,F3}$ = 1.3 Hz, H-6b), 4.85 (1H, dddd, $^3J_{3,F4}$ = 25.5 Hz, $^3J_{3,4}$ = 2.7 Hz, $^2J_{3,F3}$ = 47.4 Hz, $^3J_{3,2}$ = 10.4 Hz, H-3), 5.04 (1H, ddd, $^2J_{4,F4}$ = 50.6 Hz, $^3J_{4,3}$ = 2.7 Hz, $^3J_{4,F3}$ = 7.8 Hz, H-4), 6.33 (1H, t, $^3J_{2,1}$ = 4.3 Hz, $^4J_{1,F3}$ = 4.3 Hz, H-1); ^{13}C {^{1}H} NMR (75 MHz, $CDCl_3$) δ 20.7, 20.8 (2 × s, CO*C*H$_3$), 57.6 (dd, $^2J_{C,F3}$ = 18.4 Hz, $^3J_{C,F4}$ = 2.5 Hz, C-2), 61.1 (dd, $^3J_{C,F4}$ = 6.1 Hz, $^4J_{C,F3}$ = 2.2 Hz, C-6), 68.9 (dd, $^2J_{C,F4}$ = 18.3 Hz, $^3J_{C,F3}$ = 5.5 Hz, C-5), 85.7 (dd, $^1J_{C,F4}$ = 187.4 Hz, $^2J_{C,F3}$ = 16.8 Hz, C-4), 87.2 (dd, $^1J_{C,F3}$ = 192.4 Hz, $^2J_{C,F4}$ = 18.1 Hz, C-3), 90.4 (d, $^3J_{C,F3}$ = 9.0 Hz, C-1), 168.3, 170.3 (2 × s, *C*OCH$_3$); ^{19}F NMR (282 MHz, $CDCl_3$) δ −220.56 (1F, dddd, $^2J_{F4,4}$ = 51.0 Hz, $^3J_{F4,3}$ = 25.5 Hz, $^3J_{F4,5}$ = 25.6 Hz, $^3J_{F4,F3}$ = 15.0 Hz,

F-4), −201.52 (1F, dddd, $^{2}J_{F3,3}$ = 47.4 Hz, $^{3}J_{F3,4}$ = 7.8 Hz, $^{3}J_{F3,2}$ = 14.6 Hz, $^{3}J_{F3,F4}$ = 15.0 Hz, F-3). HRMS (ESI) *m/z* $[M + Na]^{+}$ calcd for $C_{10}H_{13}F_{2}N_{3}NaO_{5}$: 316.0715; found: 316.0713. Anal. Calcd for $C_{10}H_{13}F_{2}N_{3}O_{5}$: C, 40.96; H, 4.47; N, 14.33. Found: C, 41.38; H, 4.51; N, 13.95.

2-Acetamido-1,6-di-*O*-acetyl-2,3,4-trideoxy-3,4-difluoro-α-D-galactopyranose (8)

The α-anomer **48α** (50 mg, 0.17 mmol) was hydrogenated in ethanol (3 mL) containing Ac_2O (0.6 mL) and 10% Pd/C (43 mg) for 4 h following the general procedure. The reaction was monitored by TLC in S1. Chromatography in S1 → ethyl acetate-ethanol 20:1 furnished **8** (37 mg, 70%), mp 176–177 °C; $[\alpha]_{D}^{20}$ +137 (*c* 0.35, $CHCl_3$); ^{1}H NMR (300 MHz, $CDCl_3$) δ 2.03 (3H, s, C1-OCOC*H*$_3$), 2.08, (3H, s, NHCOC*H*$_3$), 2.15 (3H, s, C6-OCOC*H*$_3$), 4.08 (1H, dtd, $^{3}J_{5,6b}$ = 6.8 Hz, $^{3}J_{5,6a}$ = 6.4 Hz, $^{3}J_{5,F4}$ = 26.2 Hz, $^{4}J_{5,F3}$ = 2.5 Hz, H-5), 4.24 (1H, dd, $^{3}J_{5,6a}$ = 6.4 Hz, $^{2}J_{6b,6a}$ = 11.3 Hz, H-6a), 4.31 (1H, dd, $^{3}J_{5,6b}$ = 6.7 Hz, $^{2}J_{6b,6a}$ = 11.4 Hz, H-6b), 4.68 (1H, dddd, $^{3}J_{3,F4}$ = 26.0 Hz, $^{3}J_{3,4}$ = 2.5 Hz, $^{2}J_{3,F3}$ = 46.1 Hz, $^{3}J_{3,2}$ = 11.1 Hz, H-3), 4.86 (1H, dddd, $^{3}J_{2,1}$ = 4.3 Hz, $^{3}J_{2,NH}$ = 8.6 Hz, $^{3}J_{2,3}$ = 11.1 Hz, $^{3}J_{2,F3}$ = 14.5 Hz, H-2), 5.02 (1H, ddd, $^{2}J_{4,F4}$ = 51.1 Hz, $^{3}J_{4,3}$ = 2.5 Hz, $^{3}J_{4,F3}$ = 8.0 Hz, H-4), 5.69 (1H, d, $^{3}J_{2,NH}$ = 8.6 Hz, N*H*), 6.25 (1H, t, $^{3}J_{2,1}$ = 4.3 Hz, $^{4}J_{1,F3}$ = 4.3 Hz, H-1); ^{13}C {^{1}H} NMR (75 MHz, $CDCl_3$) δ 20.7 (s, C6-OCO*C*H$_3$), 20.8 (s, C1-OCO*C*H$_3$), 23.2 (s, NHCO*C*H$_3$), 47.2 (dd, $^{2}J_{C,F3}$ = 19.1 Hz, $^{3}J_{C,F4}$ = 3.2 Hz, C-2), 61.3 (dd, $^{3}J_{C,F4}$ = 6.0 Hz, $^{4}J_{C,F3}$ = 2.2 Hz, C-6), 68.7 (dd, $^{2}J_{C,F4}$ = 18.3 Hz, $^{3}J_{C,F3}$ = 5.6 Hz, C-5), 85.5 (dd, $^{1}J_{C,F4}$ = 187.9 Hz, $^{2}J_{C,F3}$ = 16.8 Hz, C-4), 86.3 (dd, $^{1}J_{C,F3}$ = 193.1 Hz, $^{2}J_{C,F4}$ = 18.7 Hz, C-3), 91.4 (d, $^{3}J_{C,F3}$ = 9.1 Hz, C-1), 168.5 (s, C1-O*C*OCH$_3$), 170.2 (s, NH*C*OCH$_3$), 170.4 (s, C6-O*C*OCH$_3$); ^{19}F NMR (282 MHz, $CDCl_3$) δ −220.78 (1F, dddd, $^{2}J_{4,F4}$ = 51.1 Hz, $^{3}J_{3,F4}$ = 26.0 Hz, $^{3}J_{5,F4}$ = 26.2 Hz, $^{3}J_{F3,F4}$ = 14.7 Hz, F-4), −203.35 (1F, dddd, $^{2}J_{3,F3}$ = 46.1 Hz, $^{3}J_{4,F3}$ = 8.0 Hz, $^{3}J_{2,F3}$ = 14.5 Hz, $^{3}J_{F3,F4}$ = 14.7 Hz, F-3). HRMS (APCI): *m/z* $[M + H]^{+}$ calcd for $C_{12}H_{18}F_{2}NO_{6}$: 310.1097; found: 310.1108. Anal. Calcd for $C_{12}H_{17}F_{2}NO_{6}$: C, 46.60; H, 5.54; N, 4.53. Found: C, 46.43; H, 5.66; N, 4.28.

2-Acetamido-4,6-di-*O*-acetyl-2,3-dideoxy-3-fluoro-D-glucopyranose (49)

Benzylamine (60 μl, 0.55 mmol) was added to a solution of **5** (70 mg, 0.20 mmol) in THF (0.7 mL) and the resulting solution was stirred at rt for 3 h. TLC in chloroform/ethanol 10:1 showed

almost complete conversion of **5**. The reaction mixture was diluted with chloroform (10 mL) and washed with 2.5% HCl (2.5 mL), the water phase was reextracted with chloroform (2 × 3 mL) and the combined chloroform extracts were dried and concentrated. Chromatography in chloroform/ethanol 10:1 gave **16** (38 mg, 62%) as a crystalline mixture of anomers (α/β 12:1); mp 175–182 °C (decomp.); $[\alpha]_D^{20}$ +63 ($CHCl_3$, 0.22); HRMS (ESI): *m/z* $[M + Na]^+$ calcd for $C_{12}H_{18}FNO_7Na$: 330.0960; found: 330.0975. Anal. Calcd for $C_{12}H_{18}FNO_7$: C, 46.91; H, 5.90; N, 4.56. Found: C, 47.32; H, 5.94; N, 4.26. NMR data for the α-anomer (**49α**): ^{1}H NMR (500 MHz, CD_3OD) δ 1.99, 2.05, 2.09 (3 × 3H, 3 × s, $COCH_3$), 4.08 (1H, dt, $^3J_{5,6a}$ = 2.0 Hz, $^2J_{6a,6b}$ = 12.1 Hz, $^5J_{6a,F}$ = 2.0 Hz, H-6a), 4.14–4.19 (2H, m, H-2, H-5), 4.24 (1H, dd, $^3J_{5,6b}$ = 4.5 Hz, $^2J_{6b,6a}$ = 12.1 Hz, H-6b), 4.73 (1H, ddd, $^3J_{3,4}$ = 8.9 Hz, $^2J_{3,F}$ = 53.4 Hz, $^3J_{3,2}$ = 11.5 Hz, H-3), 5.10 (1H, ddd, $^3J_{4,5}$ = 10.2 Hz, $^3J_{4,3}$ = 8.9 Hz, $^3J_{4,F}$ = 13.5 Hz, H-4), 5.13 (1H, t, $^3J_{1,2}$ = 3.7 Hz, $^4J_{1,F}$ = 3.7 Hz, H-1); ^{13}C {^{1}H} NMR (125 MHz, CD_3OD) δ 20.6, 20.7, 22.5 (3 × s, $COCH_3$), 54.3 (d, $^2J_{C,F}$ = 16.3 Hz, C-2), 63.5 (d, $^4J_{C,F}$ = 1.5 Hz, C-6), 68.1 (d, $^3J_{C,F}$ = 7.0 Hz, C-5), 70.8 (d, $^2J_{C,F}$ = 18.1 Hz, C-4), 91.3 (d, $^1J_{C,F}$ = 185.9 Hz, C-3), 92.8 (d, $^3J_{C,F}$ = 9.7 Hz, C-1), 171.3, 172.5, 173.5 (3 × s, $COCH_3$); ^{19}F NMR (470 MHz, $CDCl_3$) δ −200.60 (ddddd, $^2J_{F,3}$ = 53.5 Hz, $^3J_{F,4}$ = 13.5 Hz, $^3J_{F,2}$ = 10.7 Hz, $^4J_{F,1}$ = 3.7 Hz, $^5J_{F,6a}$ = 2.1 Hz). NMR data for the β-anomer **49β** (selected resonances): ^{1}H NMR (500 MHz, CD_3OD) δ 1.99, 2.04, 2.09 (3 × 3H, 3 × s, $COCH_3$), 3.70–3.73 (1H, m H-5), 3.84 (1H, ddd, $^3J_{2,1}$ = 8.4 Hz, $^3J_{2,3}$ = 10.3 Hz, $^3J_{2,F}$ = 12.0 Hz, H-2), 4.10–4.13 (1H, m, H-6a), 4.25 (1H, dd, $^3J_{5,6b}$ = 4.6 Hz, $^2J_{6b,6a}$ = 12.2 Hz, H-6b), 4.61 (1H, ddd, $^3J_{3,4}$ = 8.9 Hz, $^2J_{3,F}$ = 51.9 Hz, $^3J_{3,2}$ = 10.2 Hz, H-3), 4.73 (1H, d, $^3J_{1,2}$ = 8.4 Hz, H-1), 5.09 (1H, ddd, $^3J_{4,5}$ = 10.0 Hz, $^3J_{4,3}$ = 8.9 Hz, $^3J_{4,F}$ = 12.3 Hz, H-4); ^{19}F NMR (470 MHz, $CDCl_3$) δ −195.50 (dt, $^2J_{F,3}$ = 51.7 Hz, $^3J_{F,4}$ = 12.3 Hz, $^3J_{F,2}$ = 12.3 Hz).

2-Acetamido-3,6-di-*O*-acetyl-2,4-dideoxy-4-fluoro-D-glucopyranose (2)

Piperidine (60 µl, 0.61 mmol) was added to a solution of fluoro derivative **1** (60 mg, 0.17 mmol) in THF (1.1 mL) and the resulting solution was stirred for 7 h. TLC in chloroform-aceton 9:7 showed almost complete conversion of **1**. The reaction mixture was applied to a column of silica gel, and chromatography in chloroform-aceton 9:7 afforded **2** as syrup from which the α-anomer crystallized

upon addition of chloroform, (39 mg, 74%), mp could not be determined owing to a rapid loss of cocrystallizing chloroform, $[\alpha]_D^{20}$ +25 (MeOH, 0.36); ^{1}H and ^{13}C NMR data correspond to the reported ones [8], ^{19}F NMR (470 MHz, $CDCl_3$): δ −197.15 (m); HRMS (ESI): *m/z* $[M + Na]^+$ calcd for $C_{12}H_{18}FNNaO_7$: 330.0960; found: 330.0959.

2-Acetamido-3,6-di-*O*-acetyl-2,4-dideoxy-4-fluoro-D-galactopyranose (50)

Piperidine (80 µl, 0.81 mmol) was added to a solution of fluoro derivative **4** (80 mg, 0.23 mmol) in THF (1.5 mL) and the resulting solution was stirred for 3 h whereupon the reaction mixture turned cloudy and TLC in dichloromethane/ethanol 10:1 showed complete conversion of **4**. The reaction mixture was applied to a column of silica gel, and chromatography in dichloromethane/ethanol 100:9 followed by recrystallization from hot ethanol gave **50** (43 mg, 60%, α/β 9:1), mp 190–208 °C, $[\alpha]_D^{20}$ +74 (*c* 0.27, MeOH), HRMS (ESI): *m/z* $[M + Na]^+$ calcd for $C_{12}H_{18}FNNaO_7$: 330.0960; found: 330.0968. Anal. Calcd for $C_{12}H_{18}FNO_7$: C, 46.91; H, 5.90; N, 4.56. Found: C, 46.79; H, 5.90; N, 4.25. NMR data for α-anomer **50α**: ^{1}H NMR (500 MHz, $(CD_3)_2SO$) δ 1.83 (3H, s, NHCOCH_3), 2.03, 2.04 (2 × 3H, 2 × s, COCH_3), 4.12–4.16 (2H, m, H-6), 4.20–4.29 (2H, m, H-2, H-5), 4.92 (1H, dd, $^2J_{4,F}$ = 51.5 Hz, $^3J_{4,3}$ = 2.5 Hz, H-4), 5.05 (1H, dd, $^3J_{1,2}$ = 3.0 Hz, $^3J_{1,OH}$ = 4.7 Hz, H-1), 5.06 (1H, ddd, $^3J_{3,F}$ = 29.0 Hz, $^3J_{3,2}$ = 11.7 Hz, $^3J_{3,4}$ = 2.5 Hz, H-3), 7.10 (1H, dd, $^3J_{OH,1}$ = 4.6 Hz, $^4J_{OH,2}$ = 1.2 Hz, OH), 7.84 (1H, d, $^3J_{NH,2}$ = 8.9 Hz, NH); ^{13}C {^{1}H} NMR (125 MHz, $(CD_3)_2SO$) δ 20.6 (s, 2 × CO*C*H_3), 22.4 (s, NHCO*C*H_3), 47.3 (d, $^3J_{C,F}$ = 2.3 Hz, C-2), 62.1 (d, $^3J_{C,F}$ = 6.0 Hz, C-6), 65.9 (d, $^2J_{C,F}$ = 17.5 Hz, C-5), 68.0 (d, $^2J_{C,F}$ = 17.7 Hz, C-3), 87.2 (d, $^1J_{C,F}$ = 180.6 Hz, C-4), 91.1 (s, C-1), 169.6 (s, NH*C*OCH_3), 169.9, 170.1 (2 × s, *C*OCH_3); ^{19}F NMR (470 MHz, $(CD_3)_2SO$) δ −219.02 (dt, $^2J_{F,4}$ = 51.0 Hz, $^3J_{F,3}$ = 29.5 Hz, $^3J_{F,5}$ = 29.5 Hz). NMR data for the β-anomer **50β** (selected resonances): ^{1}H NMR (500 MHz, $(CD_3)_2SO$) δ 1.79 (3H, s, NHCOCH_3), 2.03, 2.04 (2 × 3H, 2 × s, COCH_3), 3.84 (1H, td, $^3J_{2,1}$ = 8.6 Hz, $^3J_{2,3}$ = 11.0 Hz, $^3J_{2,NH}$ = 9.2 Hz, H-2), 3.94 (1H, dt, $^3J_{5,6b}$ = 6.3 Hz, $^3J_{5,6a}$ = 6.3 Hz, $^3J_{5,F}$ = 28.3 Hz, H-5), 4.12–4.16 (2H, m, H-6), 4.68 (1H, dd, $^3J_{1,2}$ = 8.5 Hz, $^3J_{1,OH}$ = 6.9 Hz, H-1), 4.83 (1H, dd, $^2J_{4,F}$ = 51.0 Hz, $^3J_{4,3}$ = 2.7 Hz, H-4), 4.98 (1H, ddd, $^3J_{3,F}$ = 28.9 Hz, $^3J_{3,2}$ = 11.0 Hz, $^3J_{3,4}$ = 2.7 Hz, H-3), 7.00 (1H,

d, $^{3}J_{OH,1}$ = 6.7 Hz, OH), 7.80 (1H, d, $^{3}J_{NH,2}$ = 9.2 Hz, N*H*); ^{19}F NMR (470 MHz, $(CD_3)_2SO$) δ −216.48 (dt, $^{2}J_{F,4}$ = 50.9 Hz, $^{3}J_{F,3}$ = 28.8 Hz, $^{3}J_{F,5}$ = 28.8 Hz).

2-Acetamido-4,6-di-*O*-acetyl-2,3-dideoxy-3-fluoro-D-galactopyranose (51)

A mixture of fluoro analog **6** (46 mg, 0.13 mmol), column chromatography silica gel 60 (70–230 mesh, 200 mg) and methanol (2 mL, HPLC grade) was stirred at rt. The reaction was monitored by TLC in dichloromethane-ethanol 100:9. Silica gel (100 mg) and methanol (0.4 mL) were added after one week. The conversion of starting **6** ceased after about 30 days according to TLC. The reaction mixture was diluted with dichloromethane, filtered, concentrated and chromatographed in dichloromethane/ethanol 100:9 to yield **51** (32 mg) contaminated with unknown fluorine-containing compounds (^{19}F NMR and ^{1}H NMR). Recrystallization from ethyl acetate/heptane afforded **51** (16 mg, 40%) as a mixture of anomers, (α/β 20:1) in ca 97% purity (estimated by ^{19}F NMR); mp 113–116 °C; HRMS (ESI): *m/z* $[M + Na]^+$ calcd for $C_{12}H_{18}FNO_7Na$: 330.0960; found: 330.0968. NMR data for the α-anomer **51α**: ^{1}H NMR (500 MHz, $CDCl_3$) δ 2.04 (3H, s, NHCOCH_3), 2.06 (3H, s C6-OCOCH_3), 2.17 (3H, s, C4-OCOCH_3), 4.06 (1H, ddd, $^{3}J_{6b,5}$ = 6.7 Hz, $^{2}J_{6b,6a}$ = 11.4 Hz, $^{5}J_{6b,F}$ = 1.2 Hz, H-6b), 4.15 (1H, dd, $^{3}J_{6a,5}$ = 6.4 Hz, $^{2}J_{6a,6b}$ = 11.4 Hz, H-6a), 4.22 (1H, d, $^{3}J_{OH,1}$ = 3.6 Hz, OH), 4.35 (1H, tt, $^{3}J_{5,6b}$ = 6.7 Hz, $^{3}J_{5,6a}$ = 6.5 Hz, $^{3}J_{5,4}$ = 1.3 Hz, $^{4}J_{5,F}$ = 1.6 Hz, H-5), 4.58 (1H, qd, $^{3}J_{2,1}$ = 3.6 Hz, $^{3}J_{2,3}$ = 10.8 Hz, $^{3}J_{2,NH}$ = 9.4 Hz, $^{3}J_{2,F}$ = 9.4 Hz, H-2), 4.78 (1H, ddd, $^{3}J_{3,4}$ = 3.6 Hz, $^{2}J_{3,F}$ = 48.0 Hz, $^{3}J_{3,2}$ = 10.8 Hz, H-3), 5.33 (1H, t, $^{3}J_{1,2}$ = 3.4 Hz, $^{3}J_{1,OH}$ = 3.4 Hz, H-1), 5.58 (1H, ddd, $^{3}J_{4,5}$ = 1.3 Hz, $^{3}J_{4,3}$ = 3.5 Hz, $^{3}J_{4,F}$ = 6.5 Hz, H-4), 5.91 (1H, d, $^{3}J_{NH,2}$ = 9.1 Hz, NH); ^{13}C {^{1}H} NMR (125 MHz, $CDCl_3$) δ 20.7 (s, C4 and C6 CO*C*H_3), 23.3 (s, NHCO*C*H_3), 49.0 (d, $^{2}J_{C,F}$ = 18.1 Hz, C-2), 62.0 (d, $^{4}J_{C,F}$ = 2.6 Hz, C-6), 66.4 (d, $^{3}J_{C,F}$ = 5.7 Hz, C-5), 67.4 (d, $^{2}J_{C,F}$ = 16.7 Hz, C-4), 86.7 (d, $^{1}J_{C,F}$ = 191.7 Hz, C-3), 92.4 (d, $^{3}J_{C,F}$ = 9.4 Hz, C-1), 170.2 (s, C4-O*C*OCH_3), 170.6 (s, C6-O*C*OCH_3), 170.8 (s, NH*C*OCH_3); ^{19}F NMR (470 MHz, $CDCl_3$):δ −202.97 (dp, $^{2}J_{F,3}$ = 48.0 Hz, $^{3}J_{F,4}$ = 6.5 Hz, $^{3}J_{F,2}$ = 9.7 Hz, $^{4}J_{F,5}$ = 1.7 Hz, $^{5}J_{F,6b}$ = 1.2 Hz). NMR data for the β-anomer **51β** (selected resonances): ^{1}H NMR (500 MHz, $CDCl_3$) δ 3.84 (1H, tt, $^{3}J_{5,6b}$ = 6.4 Hz, $^{3}J_{5,6a}$ = 6.4 Hz, $^{3}J_{5,4}$ = 1.4 Hz, $^{4}J_{5,F}$ = 1.4 Hz, H-5), 4.04–4.15 (2H, m, H-2, H-6b), 4.19 (1H, dd, $^{3}J_{6a,5}$ = 6.4 Hz, $^{2}J_{6a,6b}$ = 11.4 Hz, H-6a), 4.66 (1H, t, $^{3}J_{1,2}$ = 7.0 Hz, $^{3}J_{1,OH}$ = 7.0 Hz, H-1), 4.73

(1H, ddd, $^3J_{3,4}$ = 3.7 Hz, $^2J_{3,F}$ = 47.5 Hz, $^3J_{3,2}$ = 10.8 Hz, H-3), 5.53 (1H, ddd, $^3J_{4,5}$ = 1.4 Hz, $^3J_{4,3}$ = 3.7 Hz, $^3J_{4,F}$ = 5.5 Hz, H-4), 5.67 (1H, d, $^3J_{OH,1}$ = 7.1 Hz, OH), 6.37 (1H, d, $^3J_{NH,2}$ = 6.5 Hz, NH); ^{13}C {^{1}H} NMR (125 MHz, $CDCl_3$) δ 54.6 (d, $^2J_{C,F}$ = 17.5 Hz, C-2), 61.6 (d, $^4J_{C,F}$ = 2.9 Hz, C-6), 66.2 (d, $^2J_{C,F}$ = 16.3 Hz, C-4), 69.9 (d, $^3J_{C,F}$ = 6.4 Hz, C-5), 87.6 (d, $^1J_{C,F}$ = 193.2 Hz, C-3), 96.8 (d, $^3J_{C,F}$ = 8.8 Hz, C-1); ^{19}F NMR (470 MHz, $CDCl_3$) δ −198.35 (ddd, $^2J_{F,3}$ = 47.5 Hz, $^3J_{F,4}$ = 5.5 Hz, $^3J_{F,2}$ = 9.5 Hz).

References for synthesis

(1) Karban, J.; Buděšínský, M.; Černý, M.; Trnka, T. *Collect. Czech. Chem. Commun.* **2001**, 66, 799.

(2) Auzanneau, F. I.; Mialon, M.; Prome, D.; Prome, J. C.; Gelas, J. *J. Org. Chem.* **1998**, 63, 6460.

(3) Staněk, J.; Černý, M. *Synthesis* **1972**, 698.

(4) Ogawa, S.; Aso, D. *Carbohydr. Res.* **1993**, 250, 177.

(5) Faghih, R.; Escribano, F. C.; Castillon, S.; Garcia, J.; Lukacs, G.; Olesker, A.; Thang, T. T. *J. Org. Chem.* **1986**, 51, 4558.

(6) Karban, J.; Horník, Š.; Červenková Šťastná, L.; Sýkora, J. *Synlett* **2014**, 25, 1253–1256.

(7) Karban, J.; Císařová, I.; Strašák, T.; Červenková Šťastná, L.; Sýkora, J. *Org. Biomol. Chem.* **2012**, 10, 394.

(8) Nishimura, S. I.; Hato, M.; Hyugaji, S.; Feng, F.; Amano, M. *Angew. Chem., Int. Ed.* **2012**, 51, 3386.

(9) Berkin, A.; Szarek, W. A.; Kisilevsky, R. *Carbohydr. Res.* **2000**, 326, 250.

II. Reaction of 3-fluoro derivative 51 with piperidine – HRMS analysis

Compound **51** (6 mg, 0.02 mmol) was dissolved in THF (0.5 mL), piperidine (6 μL, 0.06 mmol) was added and the reaction mixture was stirred at rt for 24 h. One drop was taken from the mixture at *t* = 10 min, 2 h and 24 h, diluted with THF and analyzed by HRMS. HRMS analysis detected adduct ions whose *m*/*z* corresponded to the starting 1-O-deacetylated compound **51**, the putative intermediate enal **52** resulting from dehydrofluorination of **51**, and piperidine derivative **53** arising from conjugate

addition of piperidine to **52** (Figures **S1–S4**). The relative abundance of the **52** adduct ion with respect to the **53** adduct ion decreased as the reaction progressed.

Figure S1 HRMS analysis at *t* = 10 min and suggested interpretation

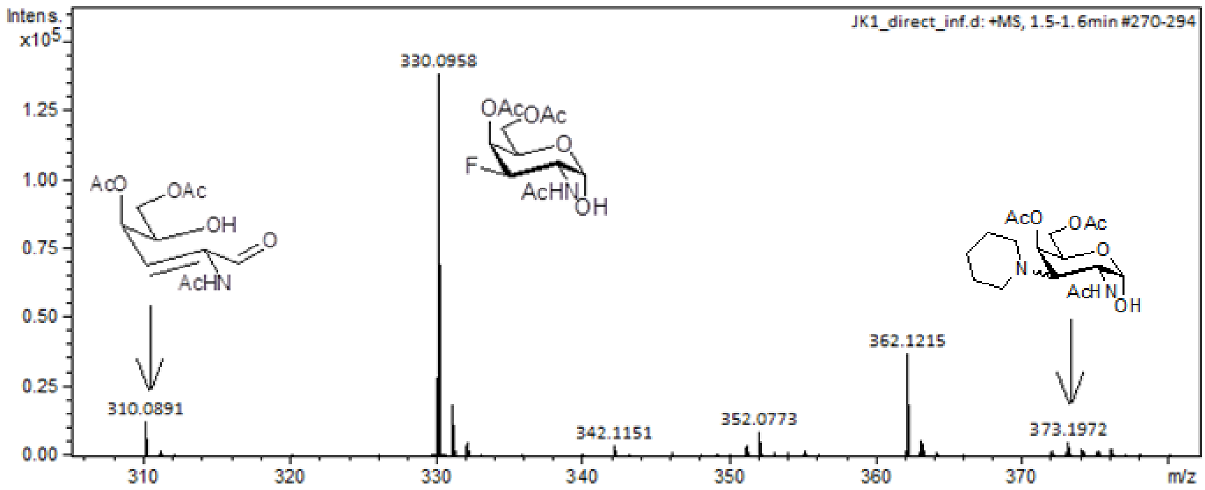

Figure S2 HRMS analysis at *t* = 2 h and suggested interpretation

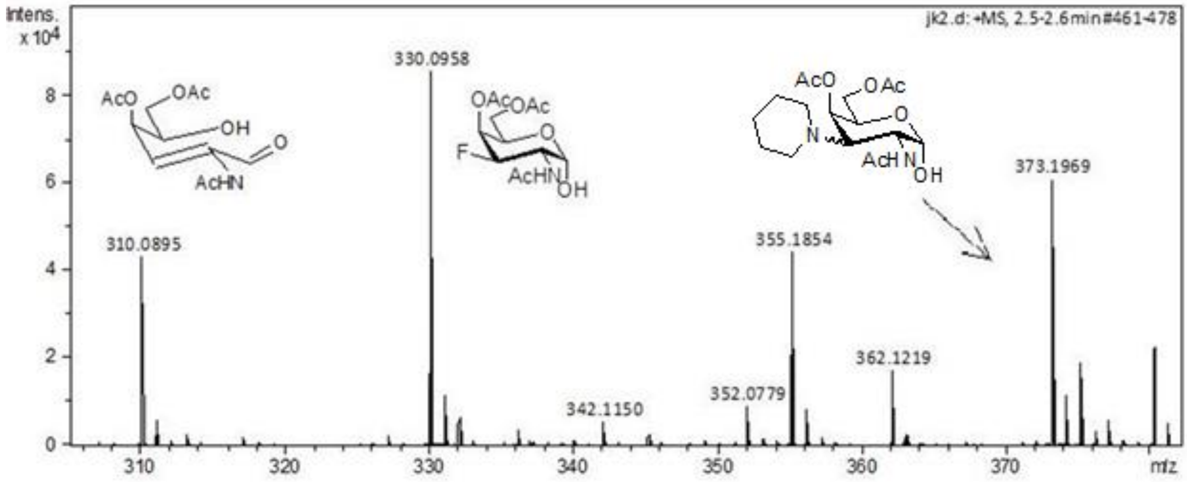

Figure S3 HRMS analysis at *t* = 24 h and suggested interpretation

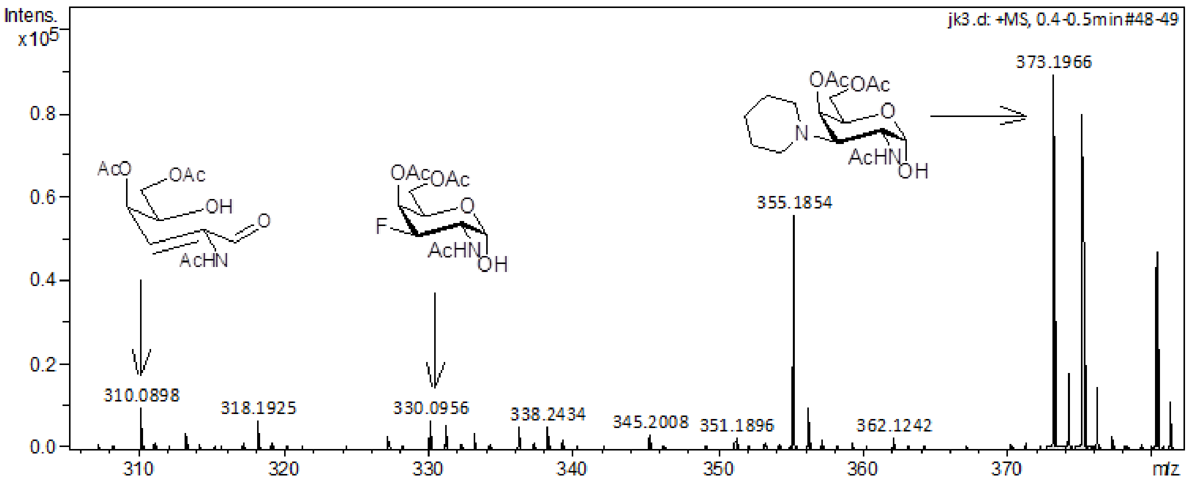

Figure S4 Calculated *m/z* for the suggested compounds

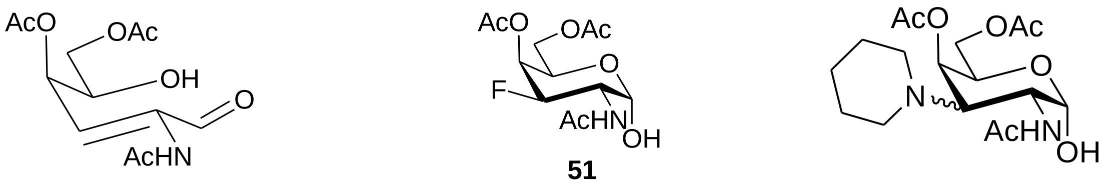

52	51	53
$C_{12}H_{17}NO_7Na^+$ calc. 310.0897, $[M+Na]^+$	$C_{12}H_{18}FNO_7Na^+$ calc. 330.0959,$[M+Na]^+$	$C_{17}H_{28}N_2O_7H^+$ calc. 373.1969, $[M+H]^+$

III. Cell lines and culture conditions and MTT assay

All tested compounds were prepared as 65 mM stock solutions in DMSO and stored at −20 °C. Cisplatin [*cis*-diamminedichloroplatinum(II)] was obtained from Ebewe Pharma GmbH, Unterach, Austria. A2780 ovarian and PC-3 prostate cancer cell lines were obtained from the American Type Culture Collection (ATCC) and grown in RPMI 1640 medium supplemented with 10% heat-inactivated fetal bovine serum (BIOCHROM AG; Berlin, Germany), 100 U/ml penicillin and 0,1 mg/ml streptomycin (Hyclone Laboratories, Utah, USA) and L-glutamine 2 mM (Gibco, Life Technologies, New York, USA) in humidified incubator at 37°C in 5% CO_2 atmosphere and subcultured twice a week.

MTT assay

Cells were seeded in 96-well plates at a density of 10,000 cells per well and incubated overnight. Subsequently cells were treated with tested D-glucosamine and D-galactosamine analogs for 24 h. Then 20 μL per well of MTT (thiazolyl blue tetrazolium bromide; 2.5 mg/mL; Sigma-Aldrich Co.; St. Louis, MO, USA) was added and incubated for 3 h under culture conditions. The medium was then removed and the formazan product was dissolved in 50 μL of DMSO (SERVA Electrophoresis GmbH, Heidelberg, Germany) and optical densities were measured at 595 nm using a microplate spectrophotometer reader (Tecan GENios, TECAN Austria GmbH, Austria). Absorbance values were used to count IC_{50} values of each tested compound using GraphPad Prism 5 software.

IV. Dose-response curves for compounds 1, 2, 4, 5, 25, 35, cisplatine, and 5-fluorouracil

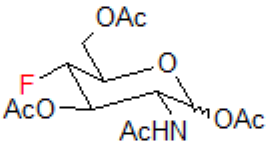

A2780

1

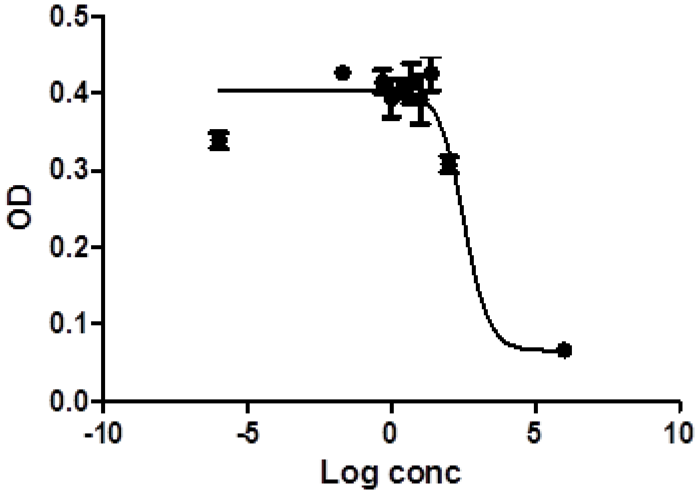

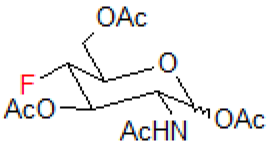

PC-3

1

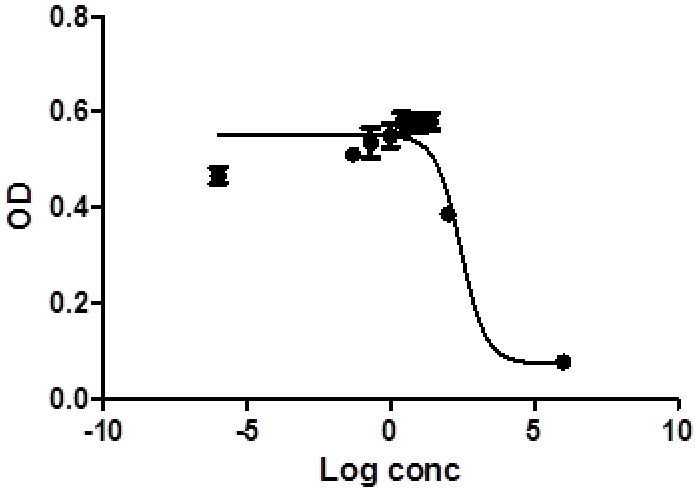

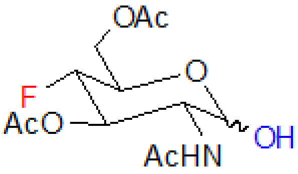
OAc
O
F
AcO
AcHN
OH

A2780

2

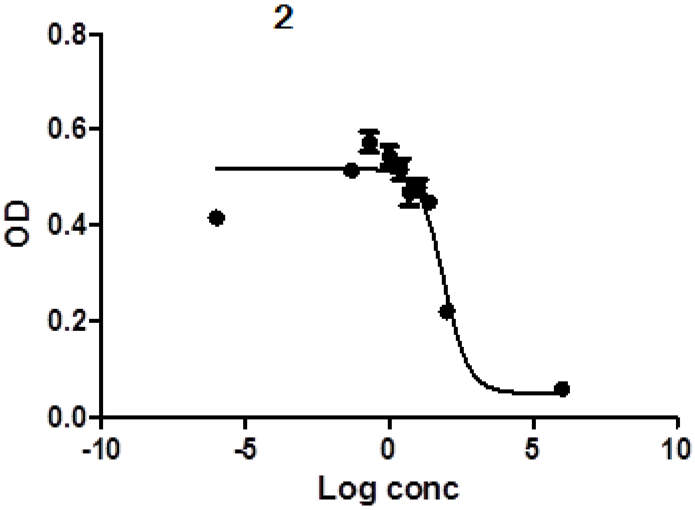
OD
Log conc
0.0
0.2
0.4
0.6
0.8
-10
-5
0
5
10

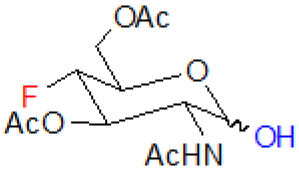
OAc
O
F
AcO
AcHN
OH

PC-3

2

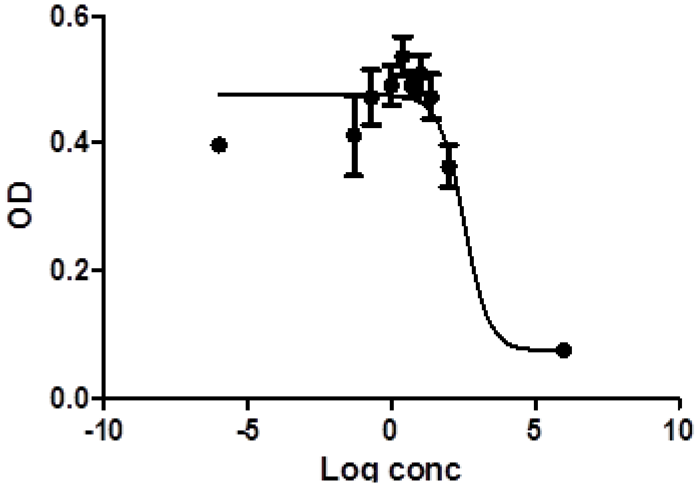
OD
Log conc
0.0
0.2
0.4
0.6
-10
-5
0
5
10

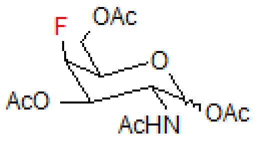
F
OAc
O
AcO
AcHN
OAc

4

A2780

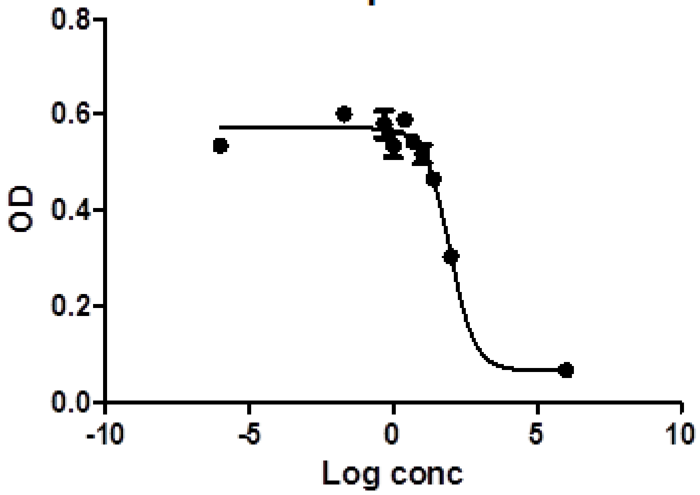
0.8
0.6
0.4
0.2
0.0
OD
-10
-5
0
5
10
Log conc

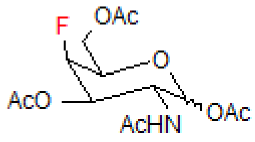
F
OAc
O
AcO
AcHN
OAc

4

PC-3

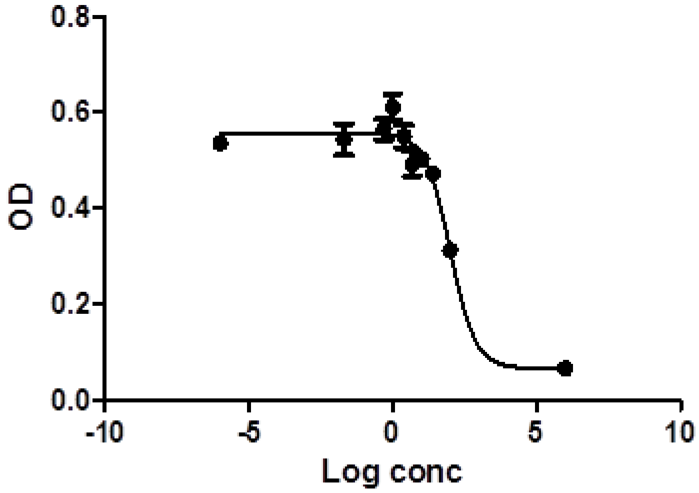
0.8
0.6
0.4
0.2
0.0
OD
-10
-5
0
5
10
Log conc

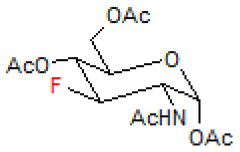
OAc
AcO
O
F
AcHN
OAc

5

A2780

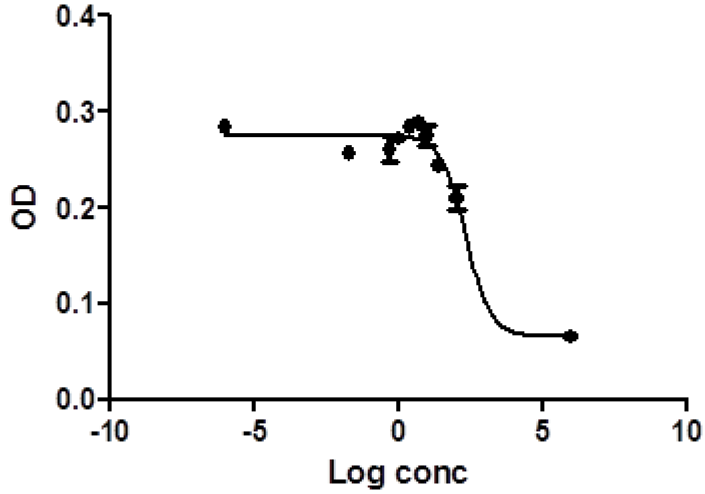
0.4
0.3
0.2
0.1
0.0
OD
-10
-5
0
5
10
Log conc

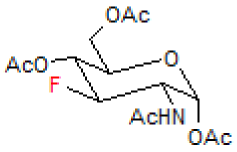
OAc
AcO
O
F
AcHN
OAc

5

PC-3

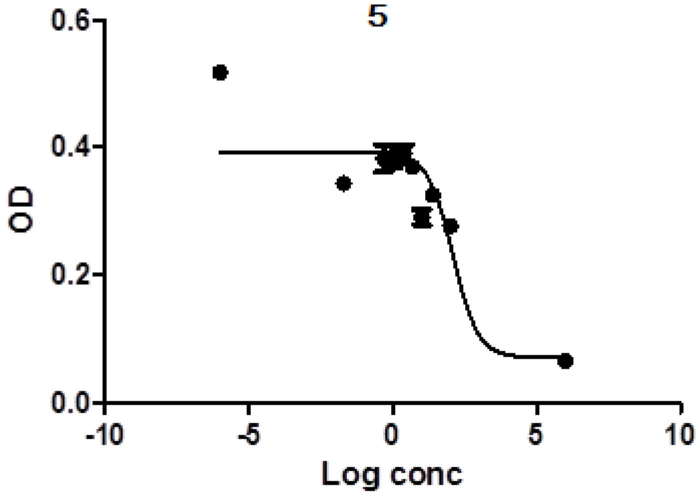
0.6
0.4
0.2
0.0
OD
-10
-5
0
5
10
Log conc

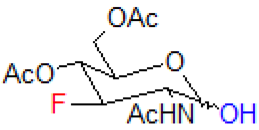

49

A2780

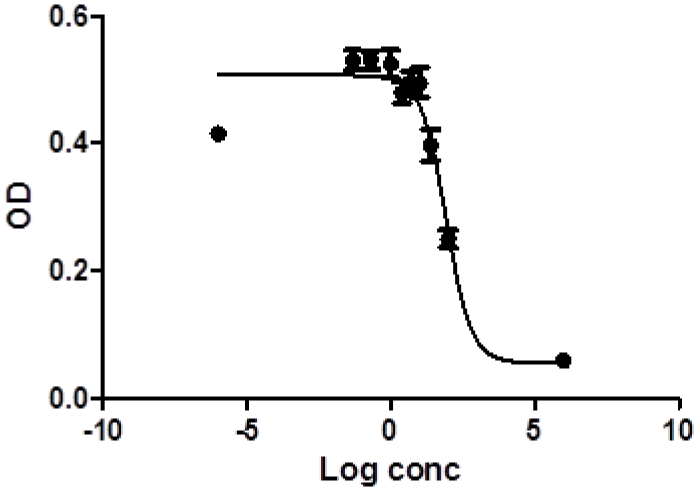

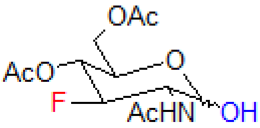

49

PC-3

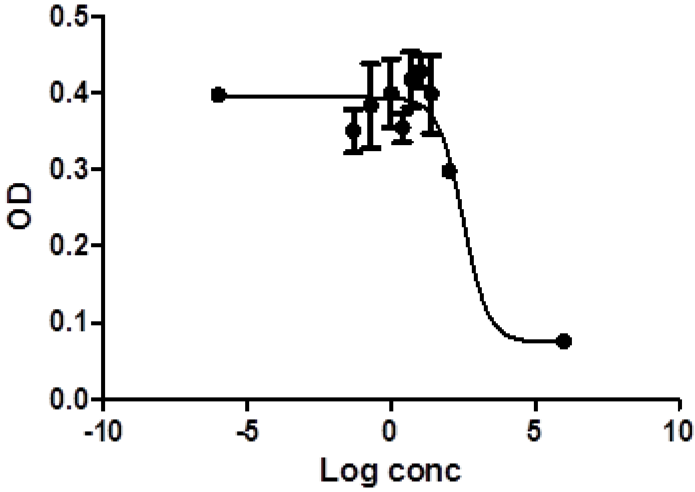

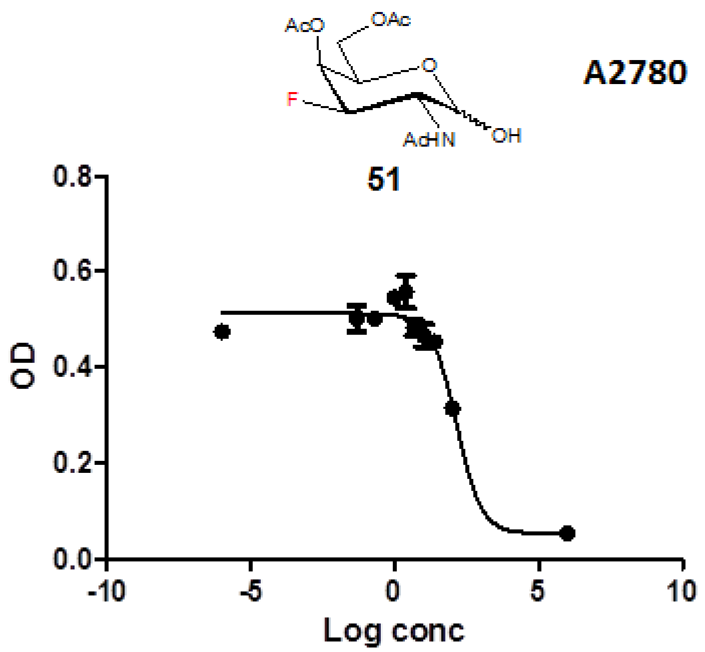
AcO
OAc
O
F
AcHN
OH
A2780
51
0.8
0.6
0.4
0.2
0.0
OD
-10
-5
0
5
10
Log conc

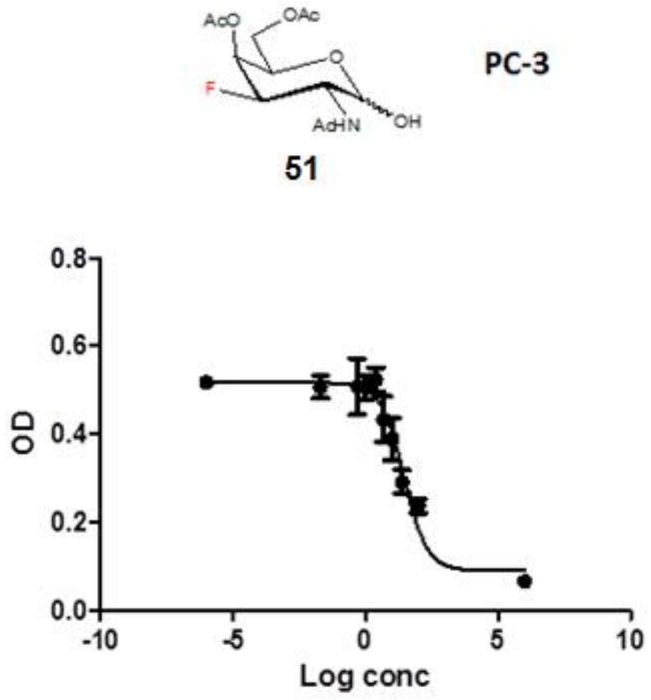
AcO
OAc
O
F
AcHN
OH
PC-3
51
0.8
0.6
0.4
0.2
0.0
OD
-10
-5
0
5
10
Log conc

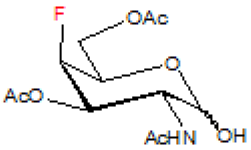

A2780

50

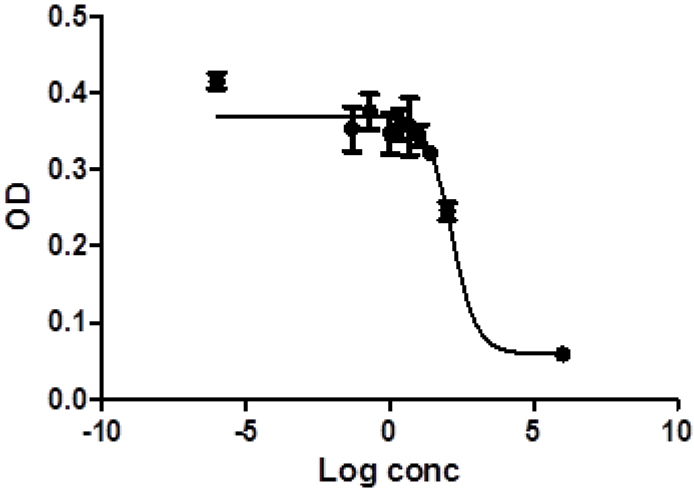

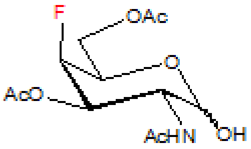

PC-3

50

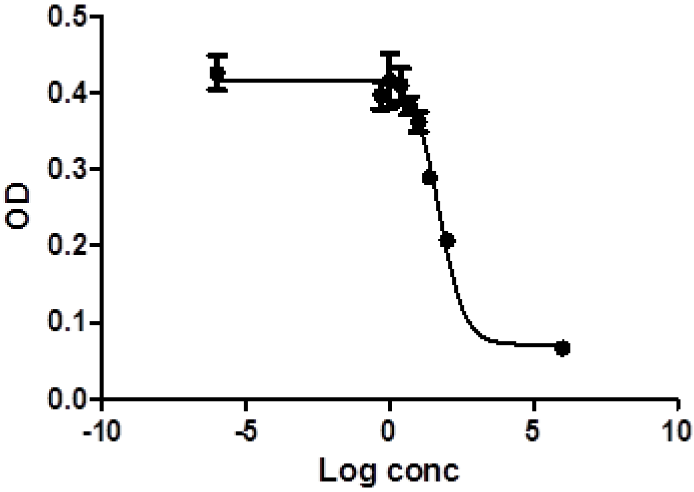

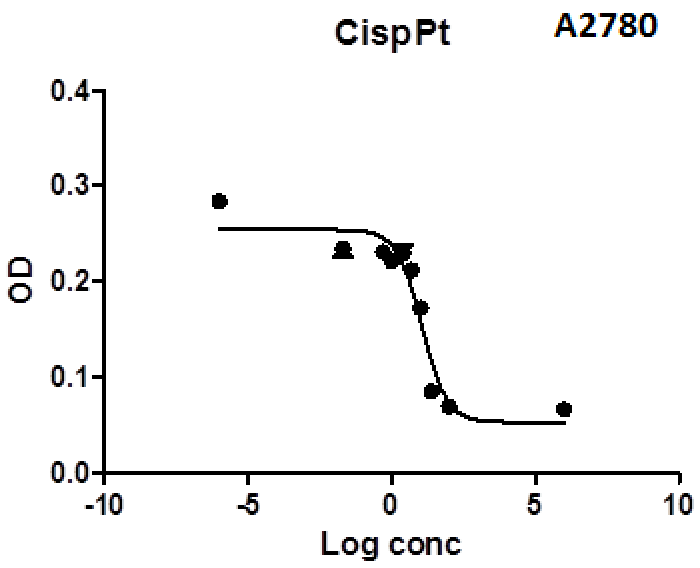
CispPt
A2780
0.4
0.3
0.2
0.1
0.0
OD
-10
-5
0
5
10
Log conc

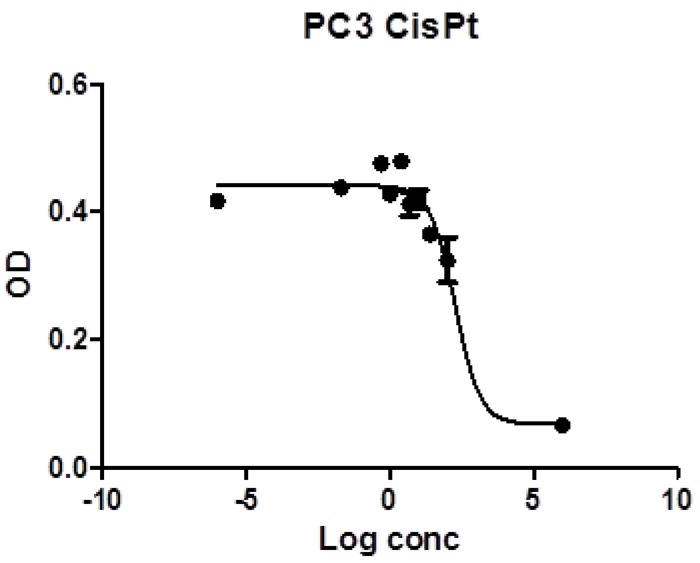
PC3 CisPt
0.6
0.4
0.2
0.0
OD
-10
-5
0
5
10
Log conc

5-fluorouracil A2780

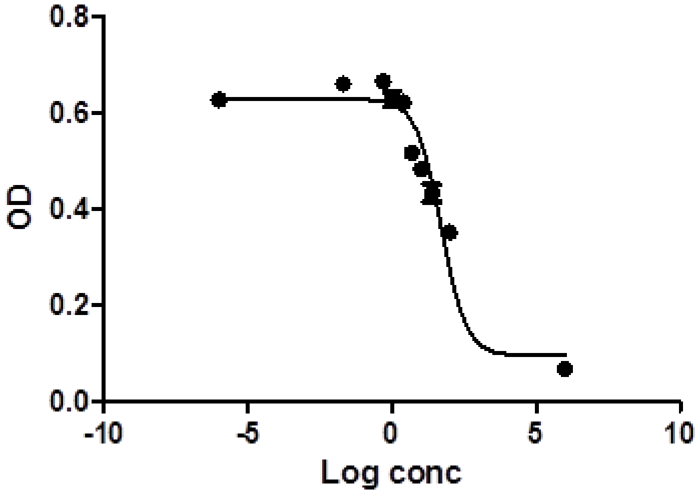

5-fluorouracil PC-3

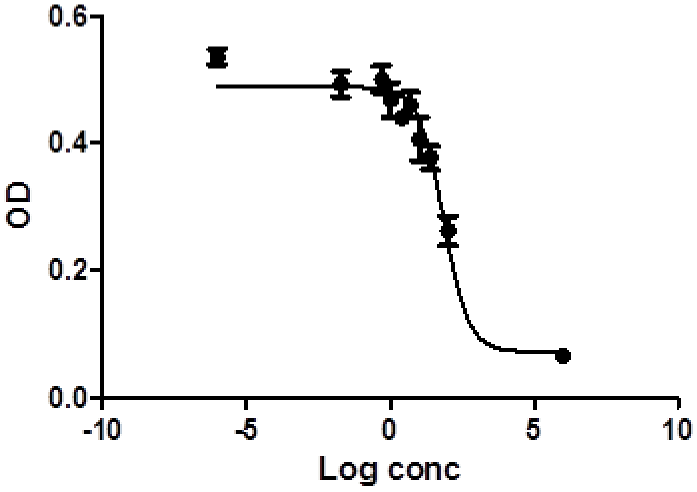

V. Crystallographic data

Diffraction data were collected at 150 K on a Nonius KappaCCD diffractometer (Enraf-Nonius) with the graphite monochromated Mo Kα radiation. Cryostream Cooler (Oxford Cryosystem) was used for the low temperature measurements. The structures were solved by direct methods (SIR92[1], SHELXL97[2]) and refined by full-matrix least-squares on F^2 values (CRYSTALS[3]). All heavy atoms were refined anisotropically. Hydrogen atoms were localized from the expected geometry and difference electron density maps and were refined isotropically. ORTEP-3[4] was used for structure presentation.

The crystallographic data for the structures reported in this paper have been deposited with the Cambridge Crystallographic Data Centre as supplementary publication. Copies of the data can be obtained free of charge on application to CCDC, e-mail: deposit@ccdc.cam.ac.uk.

X-ray data of compound **25**: $C_{14}H_{15}N_3O_5$, M = 305.29 g/mol, orthorhombic system, space group $P2_12_12_1$, a = 6.6321(2), b = 6.6477(2), c = 31.8805(11) Å, Z = 4, V = 1405.55(8) $Å^3$, Dc = 1.44 g·cm^{-3}, μ(Mo Kα) = 0.11 mm^{-1}, T = 150 K, crystal dimensions of 0.22 × 0.24 × 0.56 mm. The structure converged to the final R = 0.0311 and R_w = 0.0586 using 1735 independent reflections (θ_{max} = 27.50°). CCDC registration number 1414228.

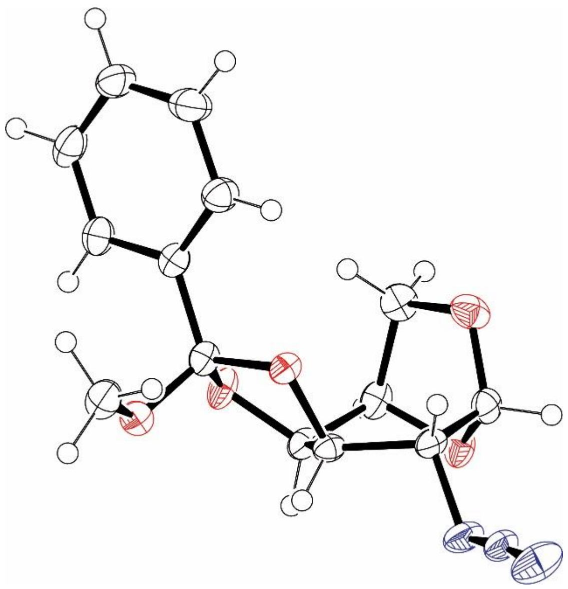

Figure S6. ORTEP projection of compound 25, ellipsoid probability 50%

X-ray data of compound **28**: $C_6H_7F_2N_3O_2$, M = 191.14 g/mol, monoclinic system, space group $P2_1$, a = 6.2155(5), b = 6.9162(6), c = 18.1759(17) Å, β = 99.855(3)°, Z = 2, V = 769.81(12) Å^3, Dc = 1.65 g.cm^{-3}, μ(Mo Kα) = 0.16 mm^{-1}, T = 150 K, crystal dimensions of 0.21 × 0.28 × 0.63 mm. The independent part of the crystal cell is formed by two molecules. The structure was refined as a 2-component twin and converged to the final R = 0.0197 and R_w = 0.0403 using 5391 independent reflections (θ_{max} = 27.48°). CCDC registration number 1414229.

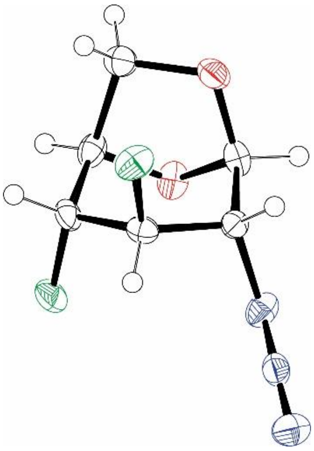

Figure S7. ORTEP projection of compound 28, ellipsoid probability 50%

X-ray data of compound **29**: $C_6H_7F_2N_3O_2$, M = 191.14 g/mol, orthorombic system, space group $P2_12_12_1$, a = 6.7543(3), b = 10.0876(4), c = 10.8762(5) Å, Z = 4, V = 741.05(6) Å^3, Dc = 1.71 g.cm^{-3}, μ(Mo Kα) = 0.16 mm^{-1}, T = 150 K, crystal dimensions of 0.26 × 0.27 × 0.46 mm. The structure converged to the final R = 0.0271 and R_w = 0.0679 using 970 independent reflections (θ_{max}=27.53°). CCDC registration number 1414230.

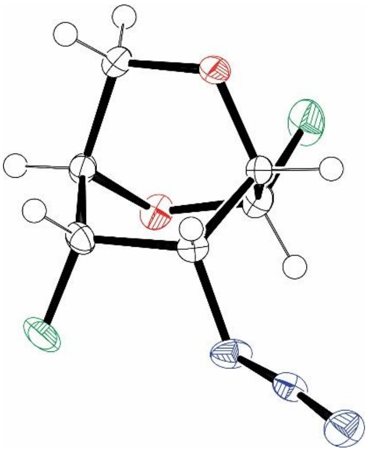

Figure S4. **ORTEP projection of compound 29, ellipsoid probability 50%.**

X-ray data of compound **41**: $C_{12}H_{16}F_1N_1O_6$, M = 289.26 g/mol, trigonal system, space group *R*3, *a* = 19.4644(7), *b* = 19.4544(7), *c* = 8.9962(5) Å, γ = 120°, *Z* = 9, *V* = 2951.7(2) Å^3, *Dc* = 1.46 g.cm^{-3}, μ(Mo Kα) = 0.13 mm^{-1}, *T* = 150 K, crystal dimensions of 0.19 × 0.23 × 0.61 mm. The structure converged to the final *R* = 0.0321 and R_w = 0.0647 using 1385 independent reflections (θ_{max}=27.48°). CCDC registration number 1414227.

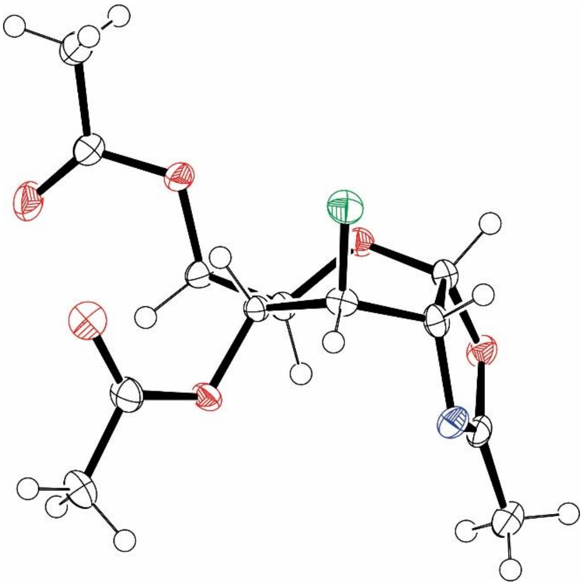

Figure S5. ORTEP projection of compound 41, ellipsoid probability 50%

References for crystallographic data

(1) Altomare, A.; Burla, M. C.; Camalli, M.; Cascarano, G.; Giacovazzo, C.; Guagliardi. A.; Polidori, G. *J. Appl. Crystallogr.*, **1994**, *27*, 435-435.

(2) Sheldrick, G. M.: SHELXL97. Program for Crystal Structure Refinement from Diffraction Data, University of Göttingen, Göttingen **1997**.

(3) Betteridge, P. W.; Carruthers, J. R.; Cooper, R. I.; Prout, K.; Watkin, D. J. *J. Appl. Cryst.* **2003**, 36, 1487.

(4) Farrugia L. J. *J. Appl. Cryst.* **1997**, *30*, 565.
