# Supplementary material for: Synthesis and in vitro cytotoxicity of acetylated 3-fluoro, 4-fluoro and 3,4-difluoro analogs of D-glucosamine and D-galactosamine
Source: Beilstein J Org Chem. 2016 Apr 20;12:750–9. doi: 10.3762/bjoc.12.75 (PMC4901990; doi:10.3762/bjoc.12.75)
Supplement: File 2 — NMR spectra for compounds 1, 2, 4–8, 12, 18–22, 25, 26, 28, 29, 31, 40–46, and 48–51. [file Beilstein_J_Org_Chem-12-750-s002.pdf]

Supporting Information File 2

for

Synthesis and in vitro cytotoxicity of acetylated 3-fluoro, 4-fluoro and 3,4-difluoro analogs of D-glucosamine and D-galactosamine

Štěpán Horník[1], Lucie Červenková Šťastná[1], Petra Cuřínová[1], Jan Sýkora[1], Kateřina Káňová[2,] Roman Hrstka[2], Ivana Císařová[3], Martin Dračínský[4] and Jindřich Karban[*1]

Address: [1]Institute of Chemical Process Fundamentals of the CAS, Rozvojová 135, 165 02 Praha, Czech Republic, [2]Regional Centre for Applied and Molecular Oncology, Masaryk Memorial Cancer Institute, Žlutý kopec 7, 656 53 Brno, Czech Republic, [3]Department of Inorganic Chemistry, Charles University, Hlavova 2030, 128 43 Praha 2, Czech Republic and [4]Institute of Organic Chemistry and Biochemistry, Flemingovo nám. 2, 166 10 Praha 6, Czech Republic
Email: Jindřich Karban - karban@icpf.cas.cz

* Corresponding author

NMR spectra for compounds 1, 2, 4–8, 12, 18–22, 25, 26, 28, 29, 31, 40–46, and 48–51

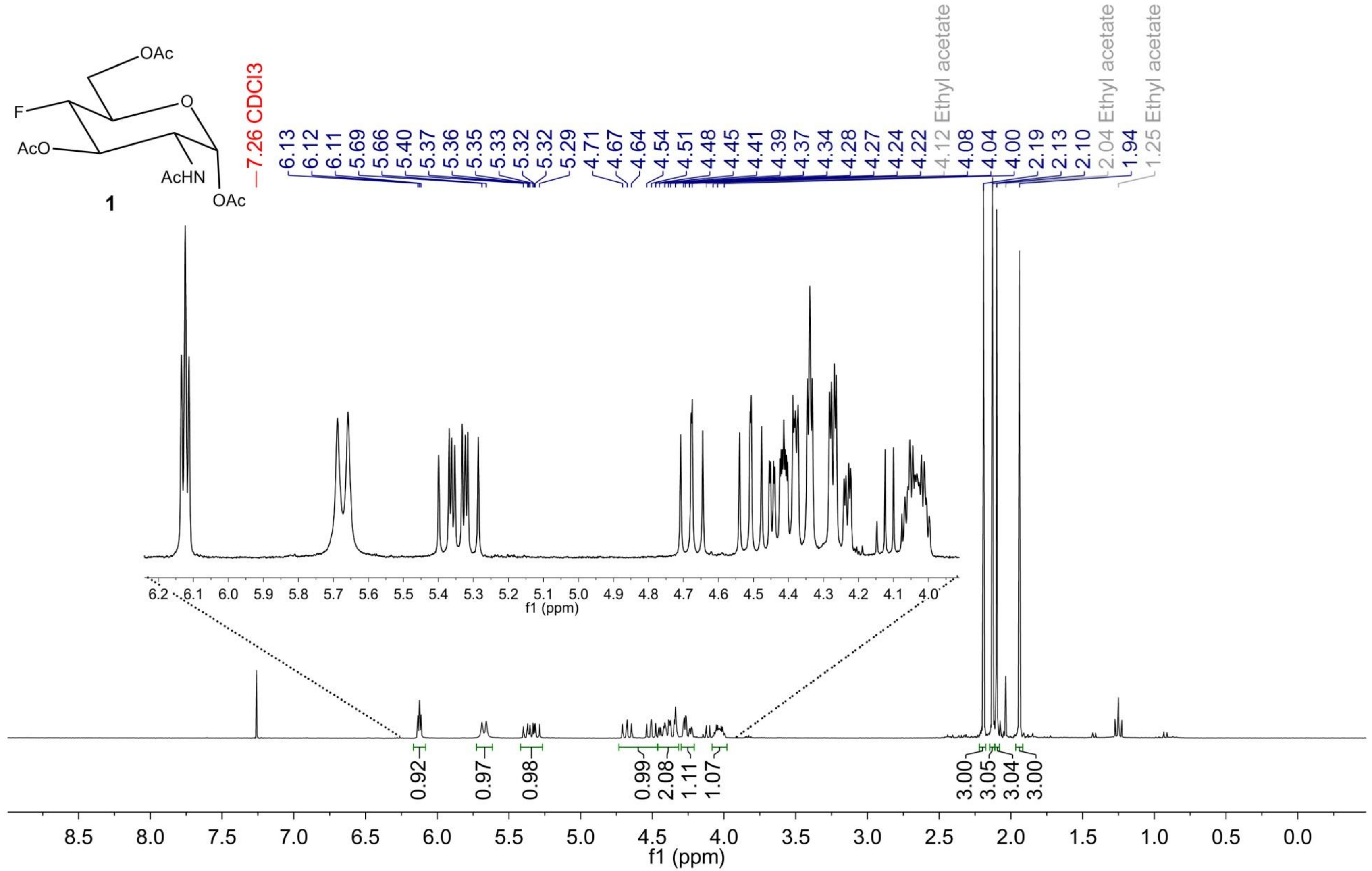

^{1}H NMR (300 MHz, $CDCl_3$) of **1α** (α-anomer).

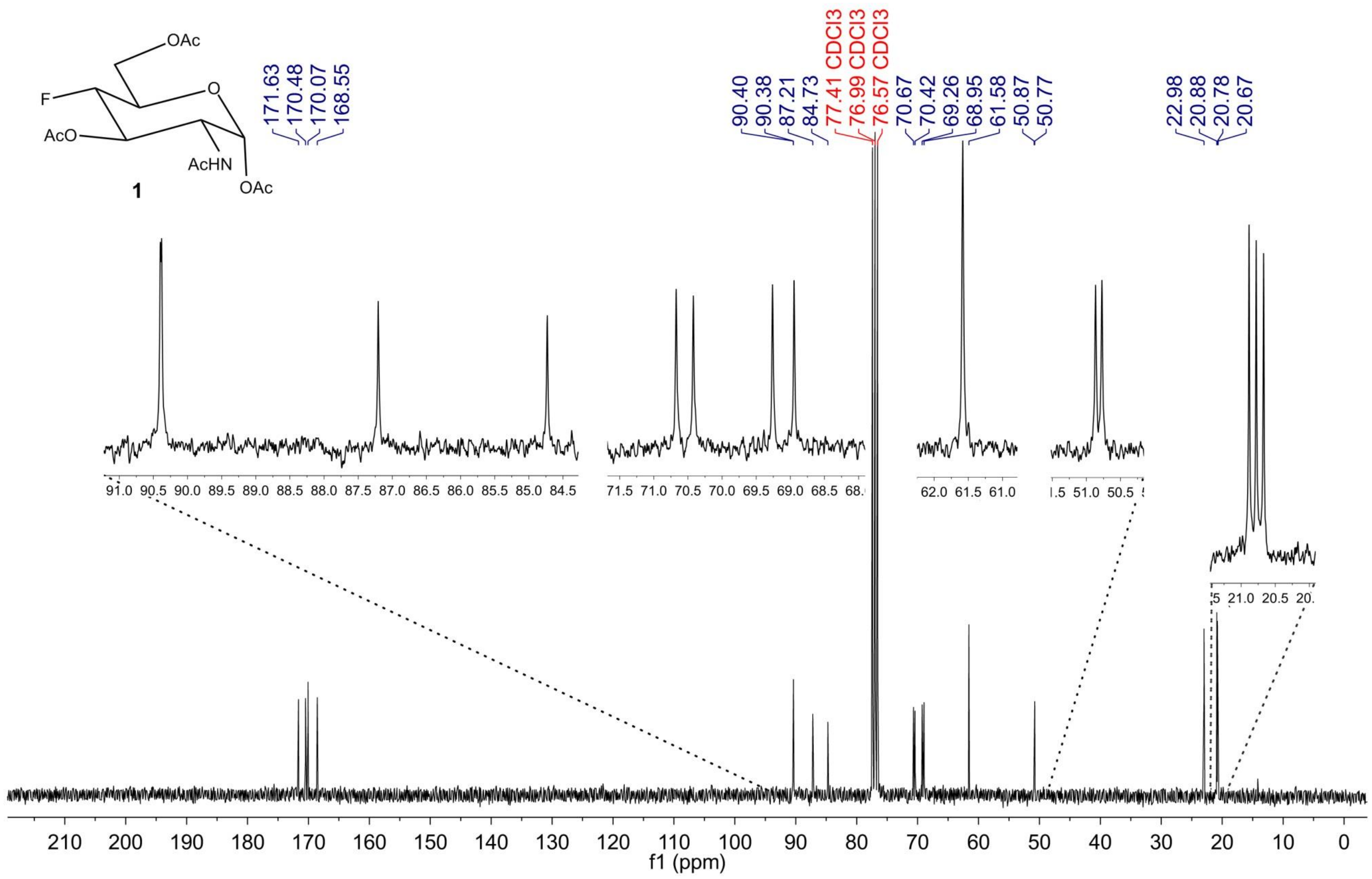

^{13}C {^{1}H} NMR (75 MHz, $CDCl_3$) of **1α** (α-anomer).

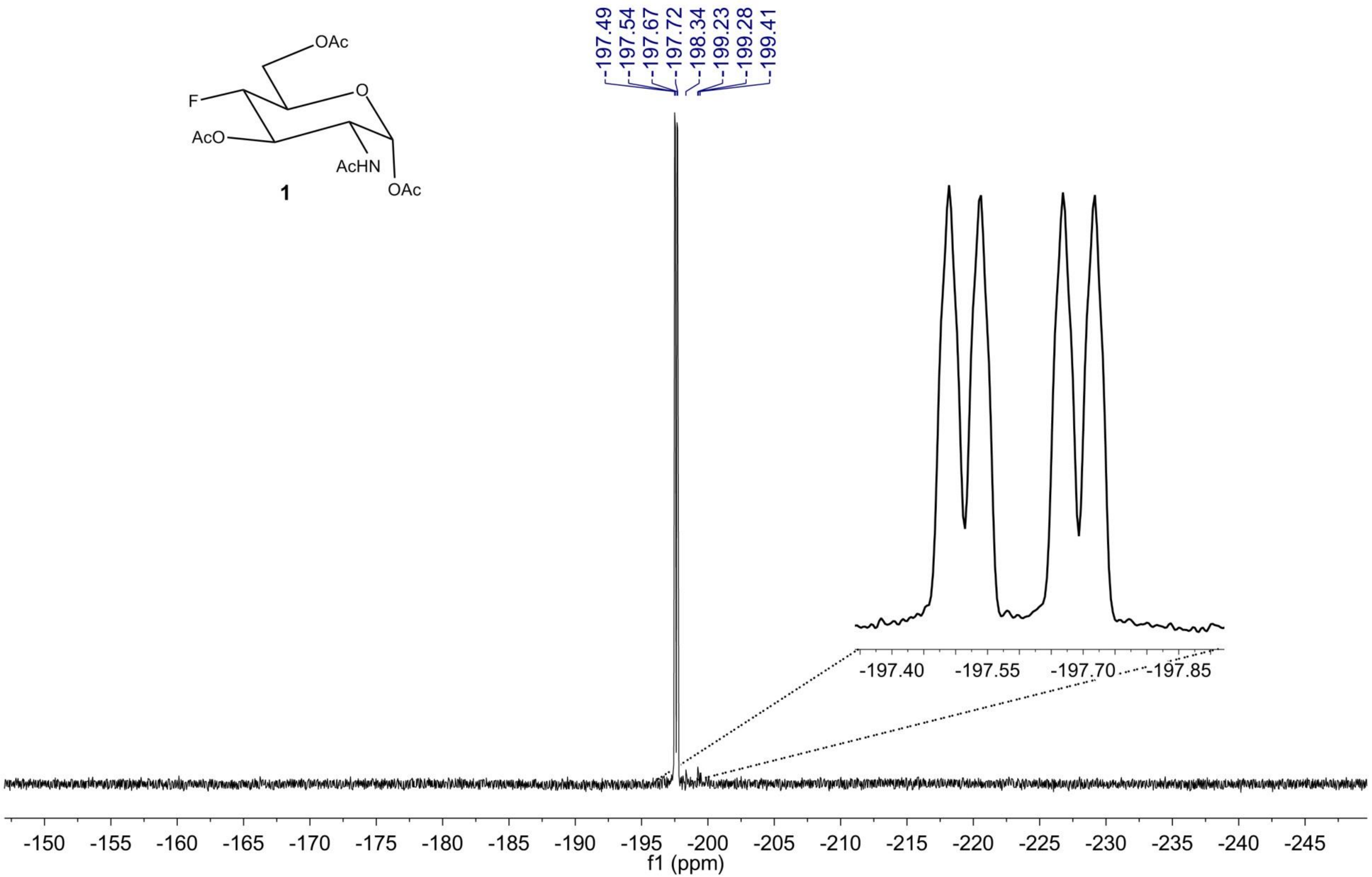

^{19}F NMR (282 MHz, $CDCl_3$) of **1α** (α-anomer).

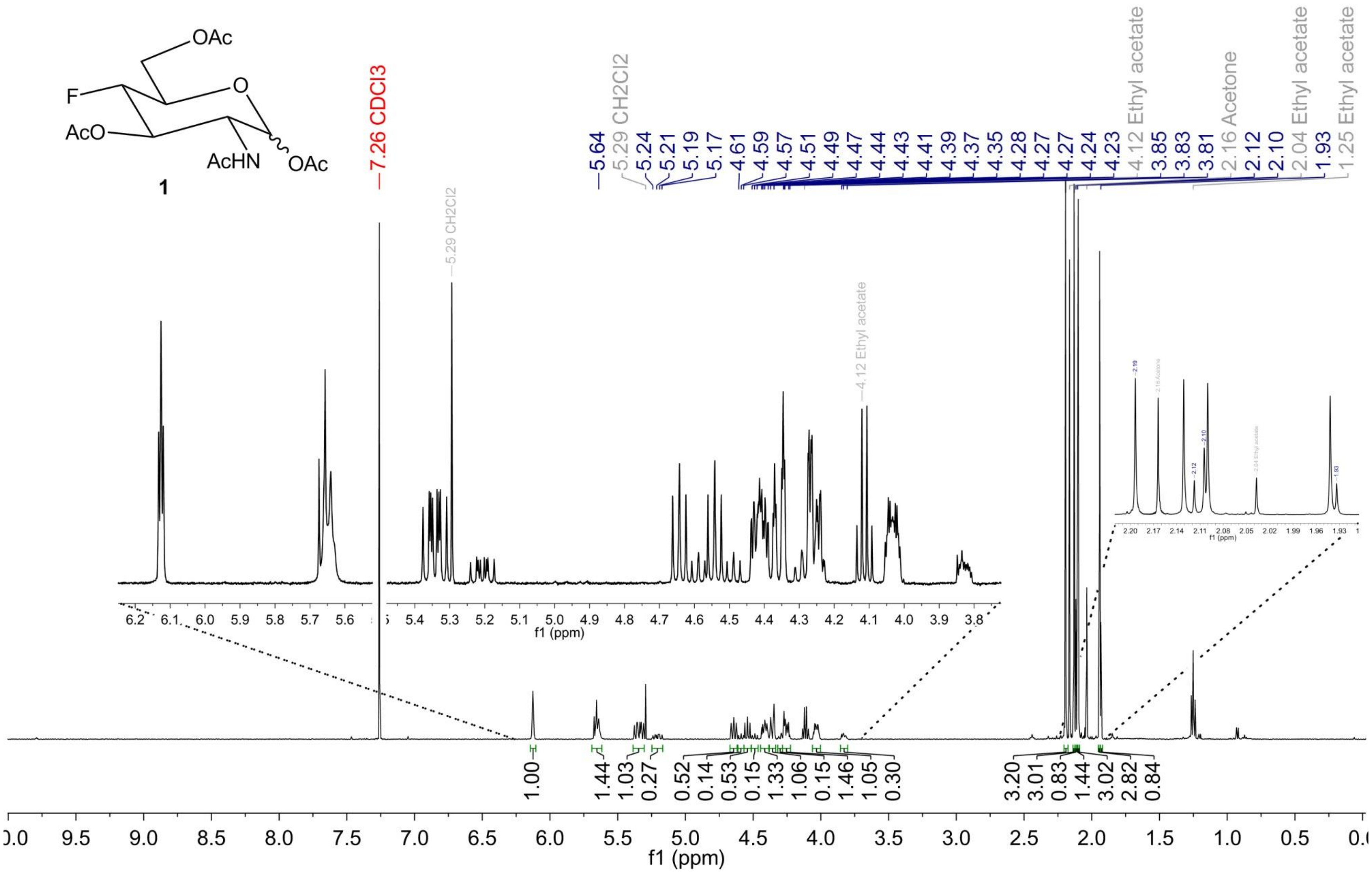

^{1}H NMR (500 MHz, $CDCl_3$) of **1** (α-anomer/β-anomer ca 1:0.3).

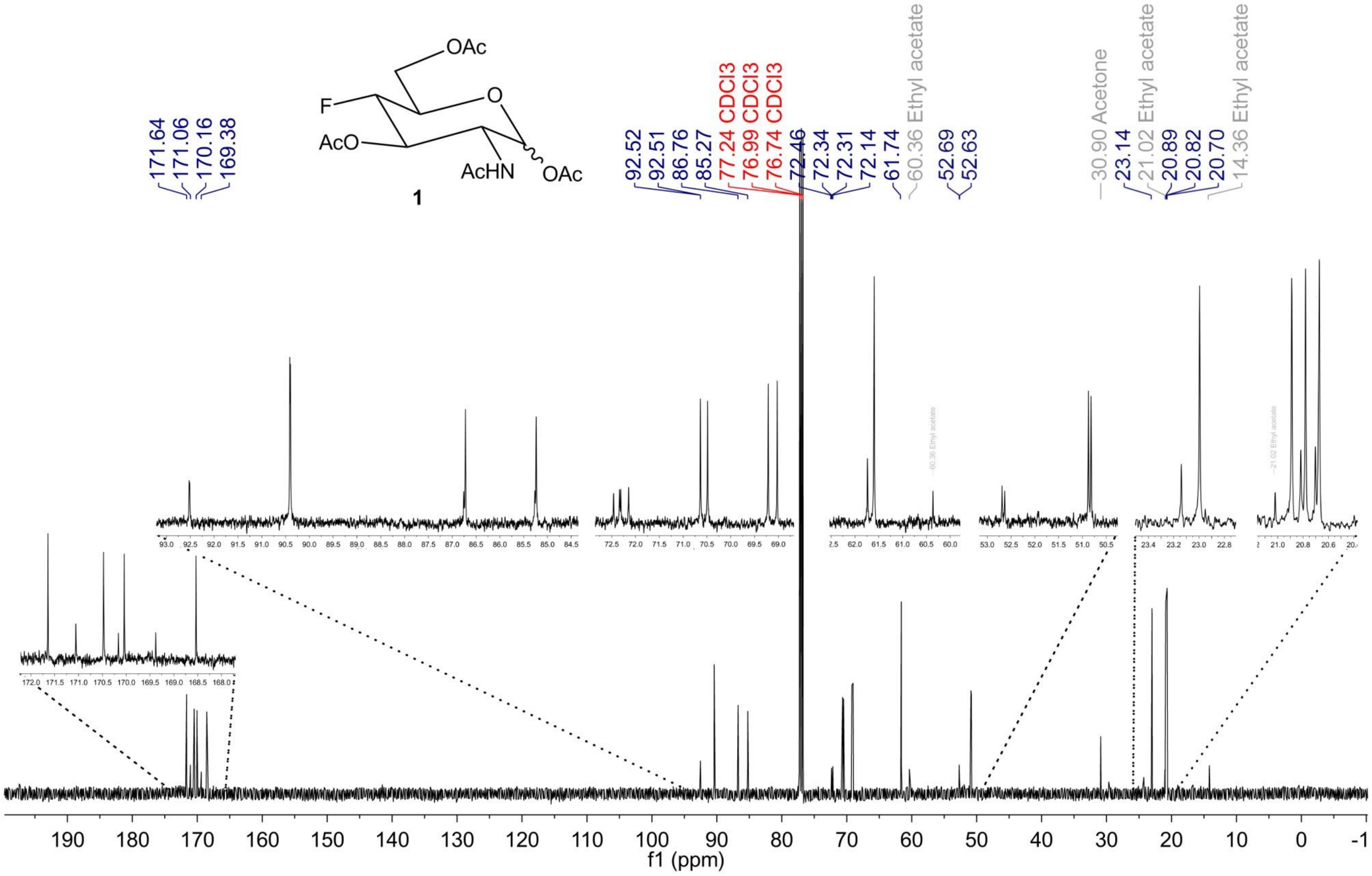

$^{13}C\ \{^1H\}$ NMR (125 MHz, $CDCl_3$) of **1** (α-anomer/β-anomer ca 1:0.3).

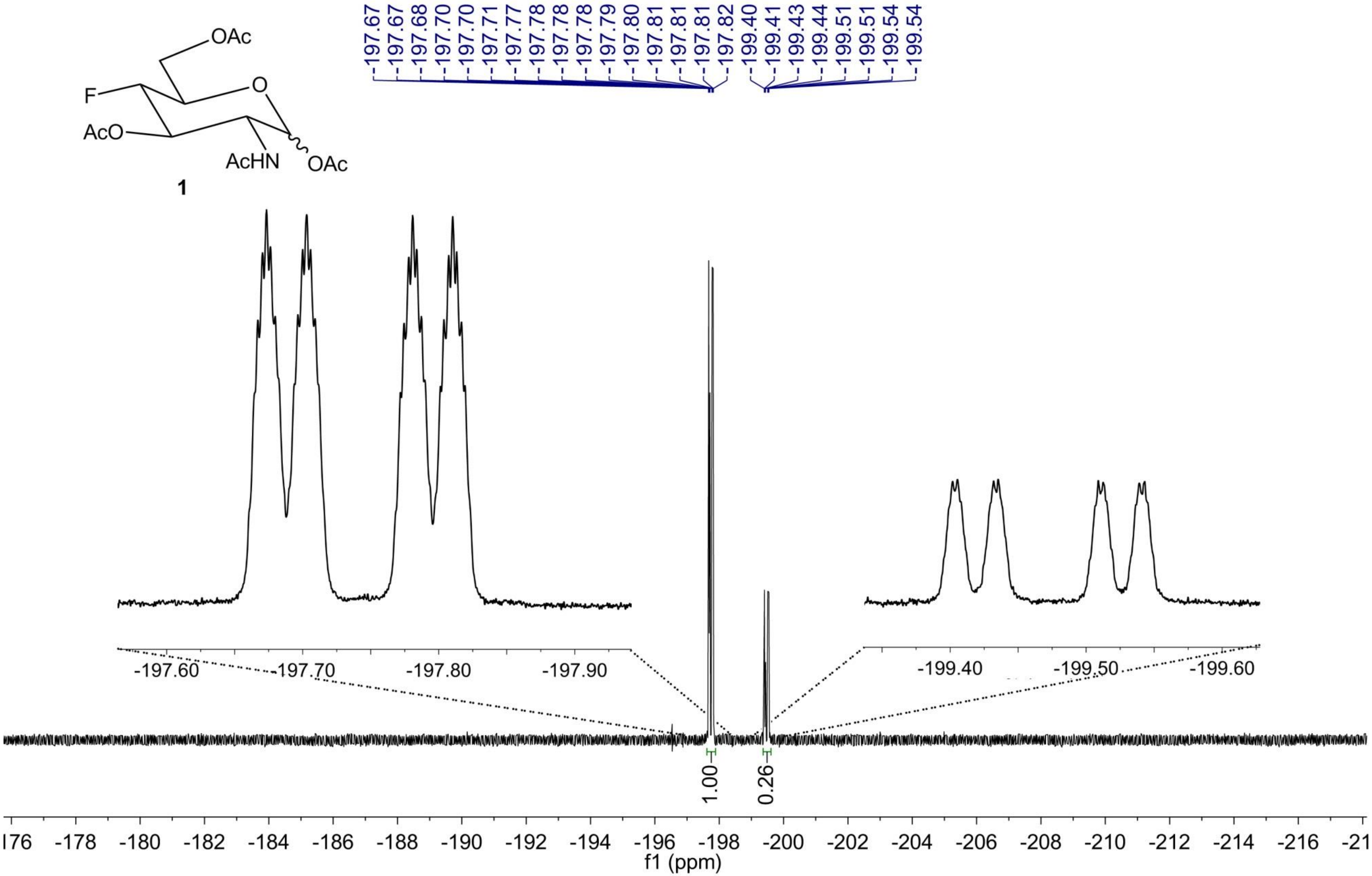

^{19}F NMR (470 MHz, $CDCl_3$) of **1** (α-anomer/β-anomer ca 1:0.3)

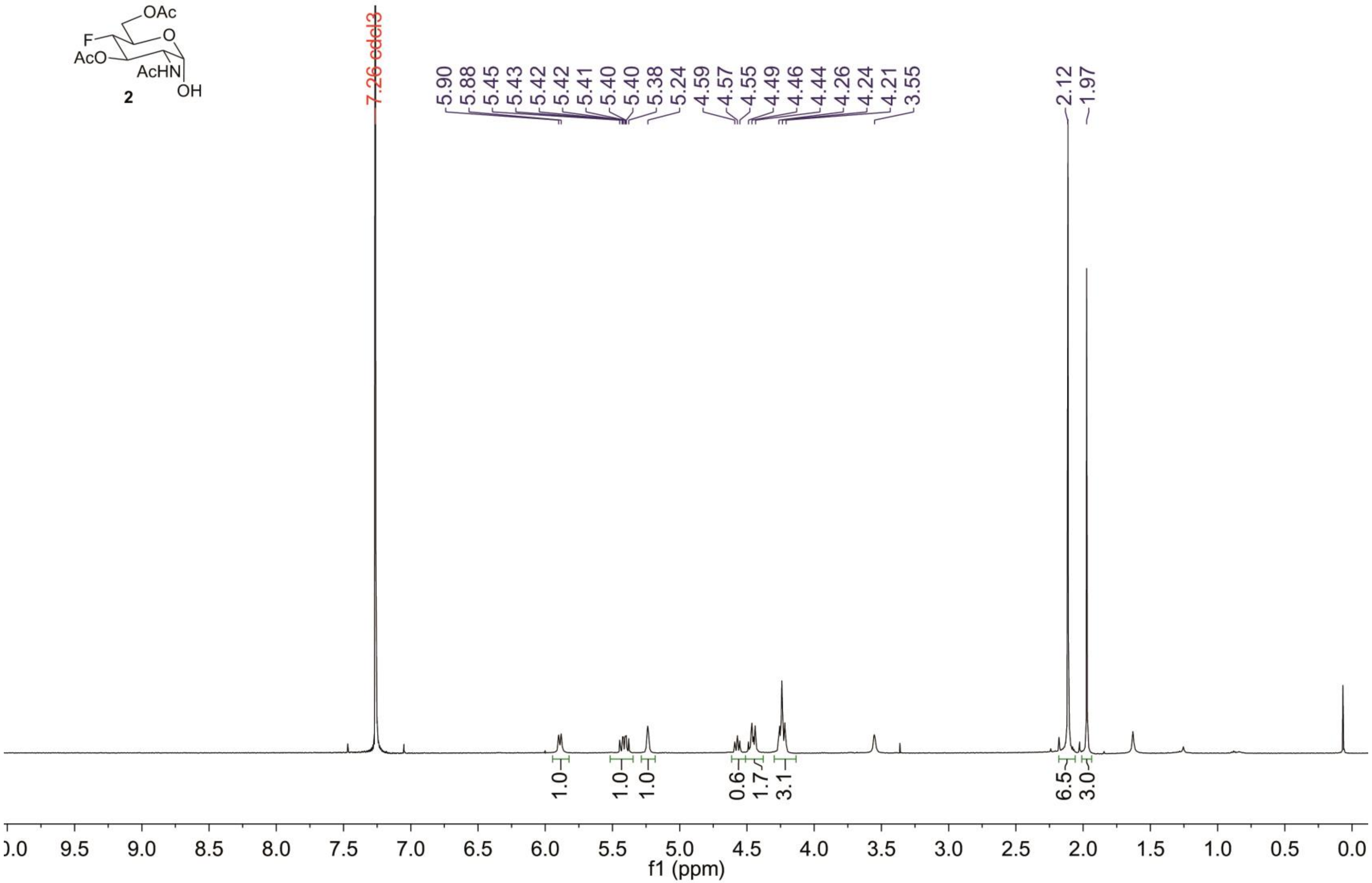

^{1}H NMR (500 MHz, $CDCl_3$) of **2** (α-anomer)

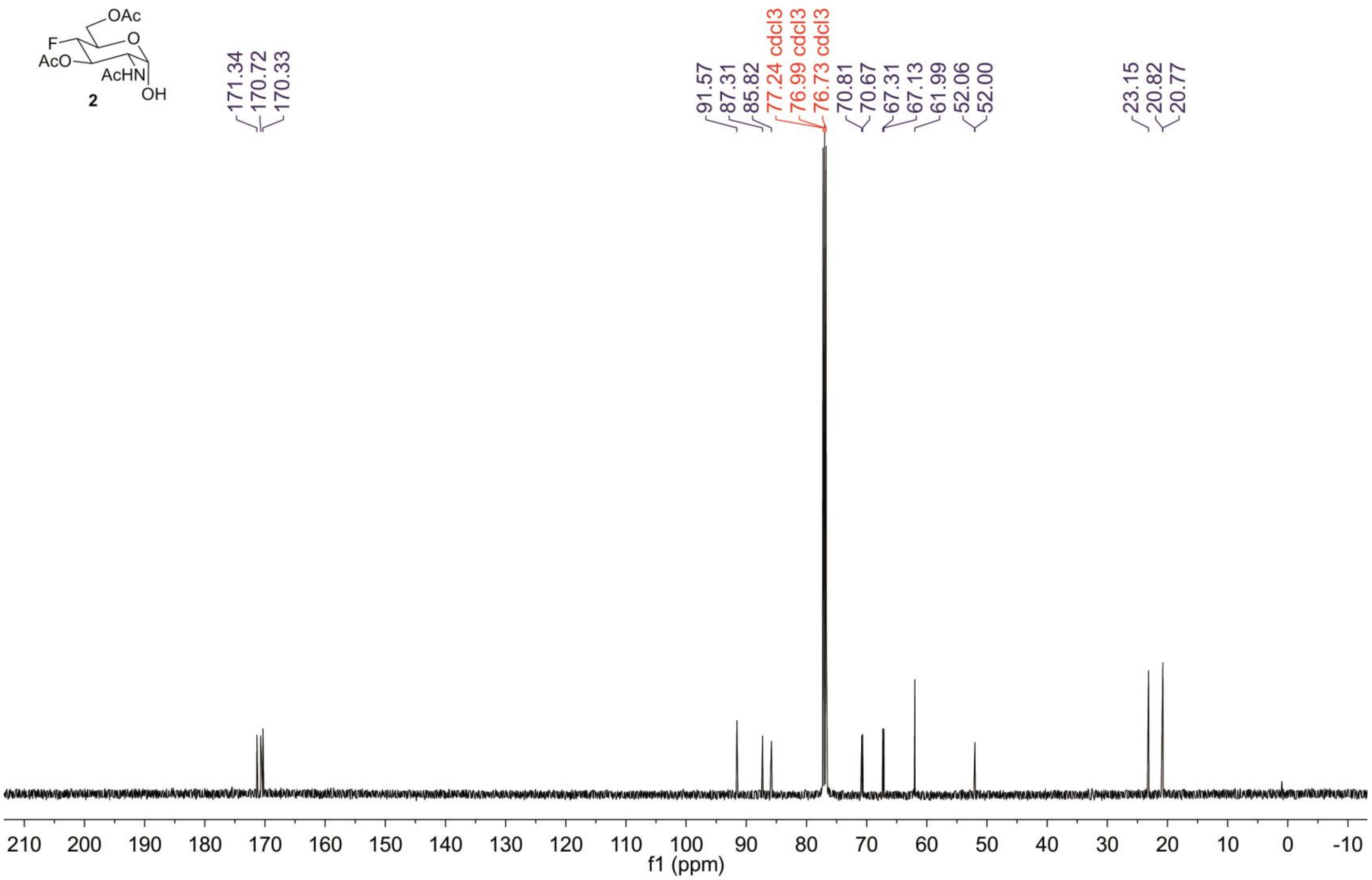

^{13}C {^{1}H} NMR (125 MHz, $CDCl_3$) of **2** (α-anomer)

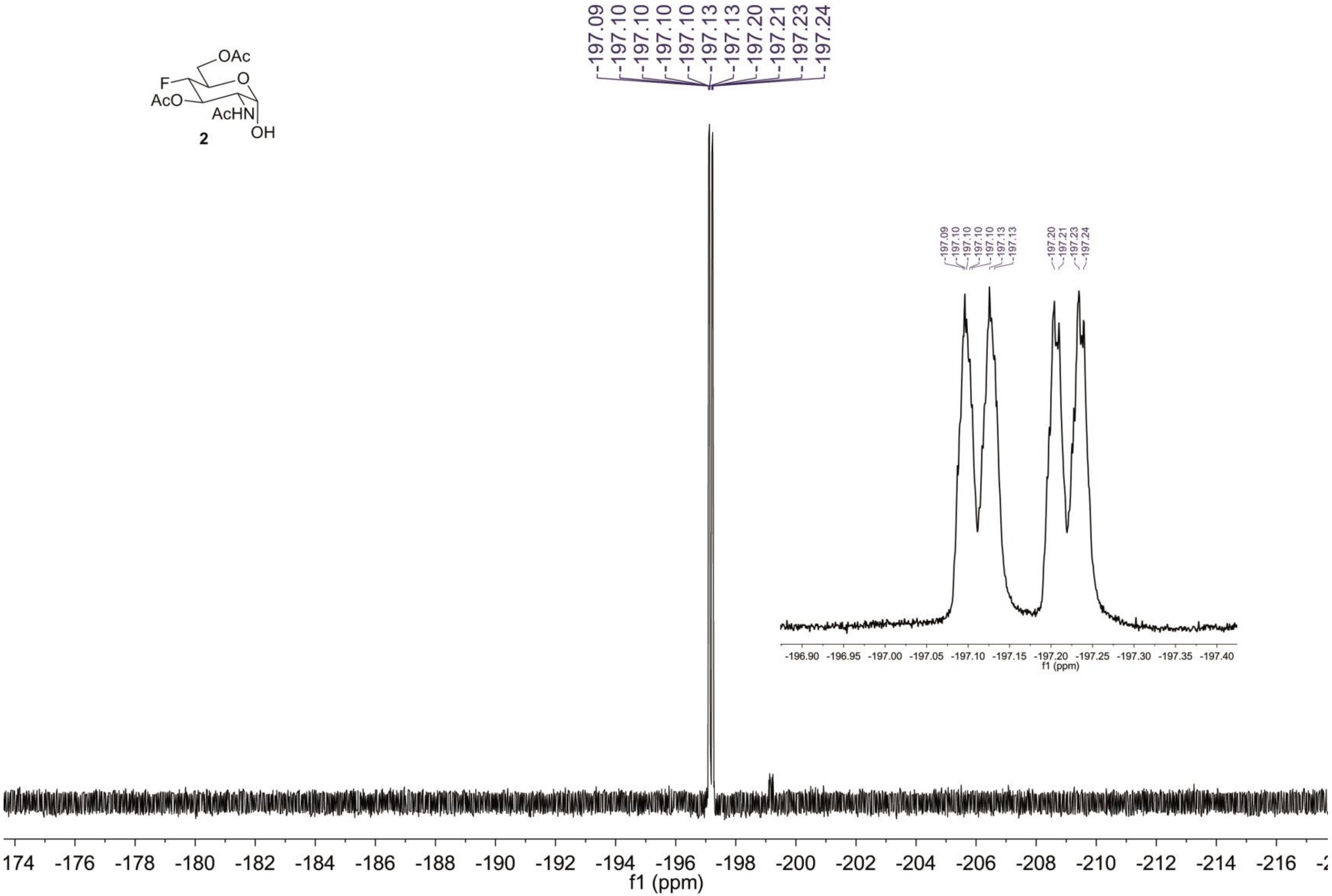

^{19}F NMR (470 MHz, $CDCl_3$) of **2** (α-anomer)

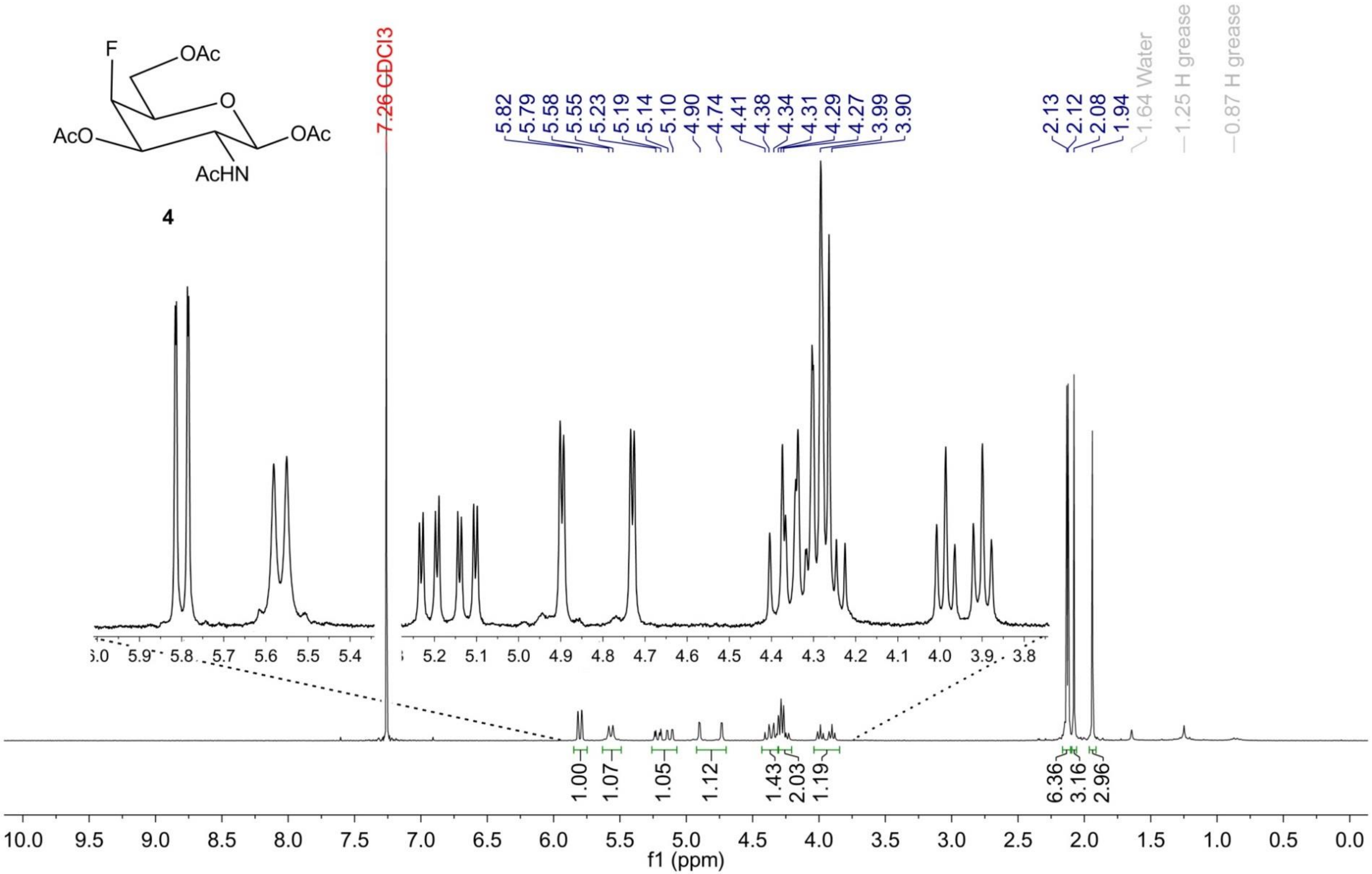

^{1}H NMR (300 MHz, $CDCl_3$) of **4β** (β-anomer).

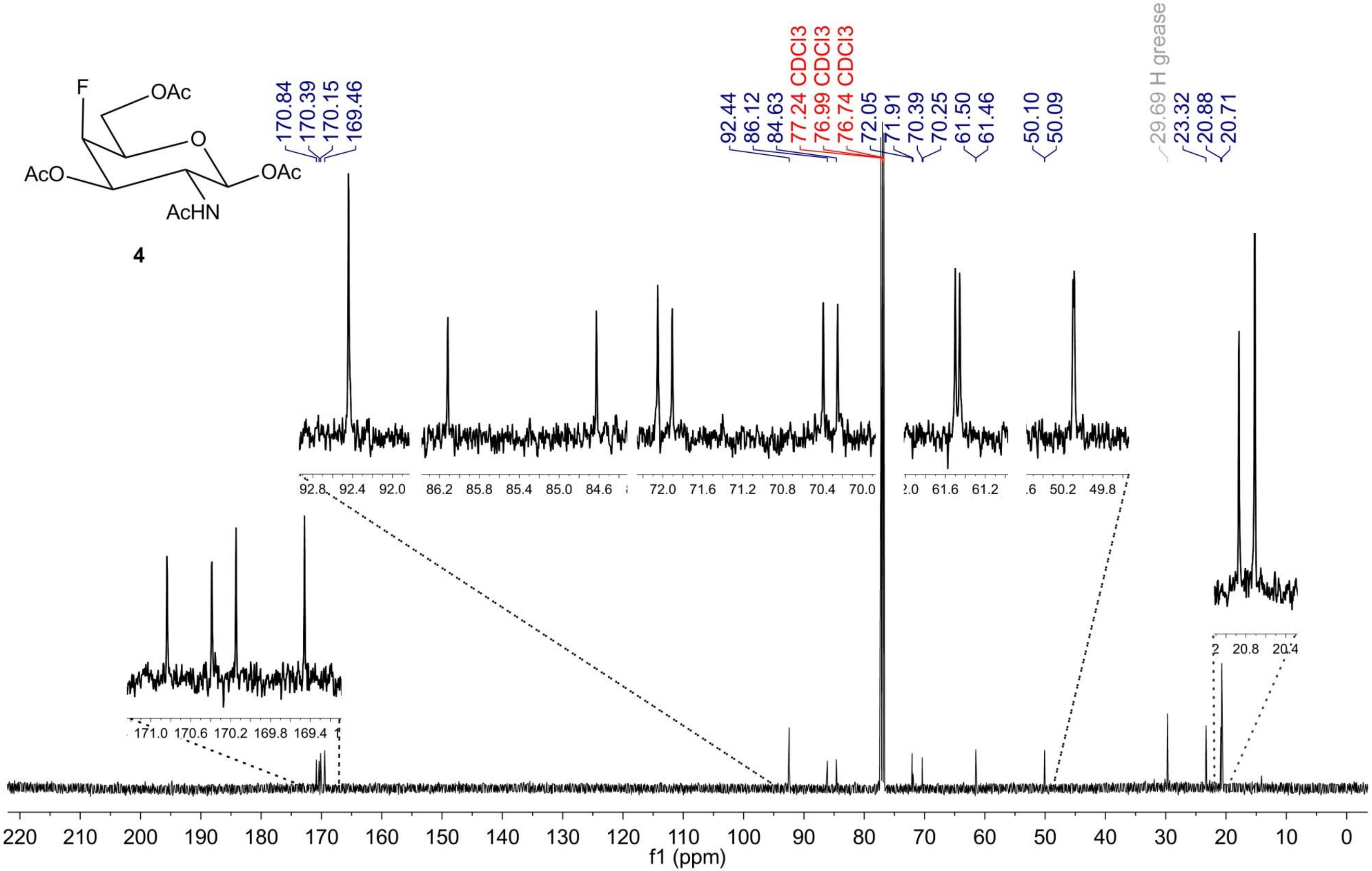

^{13}C {^{1}H} NMR (75 MHz, $CDCl_3$) of **4β** (β-anomer).

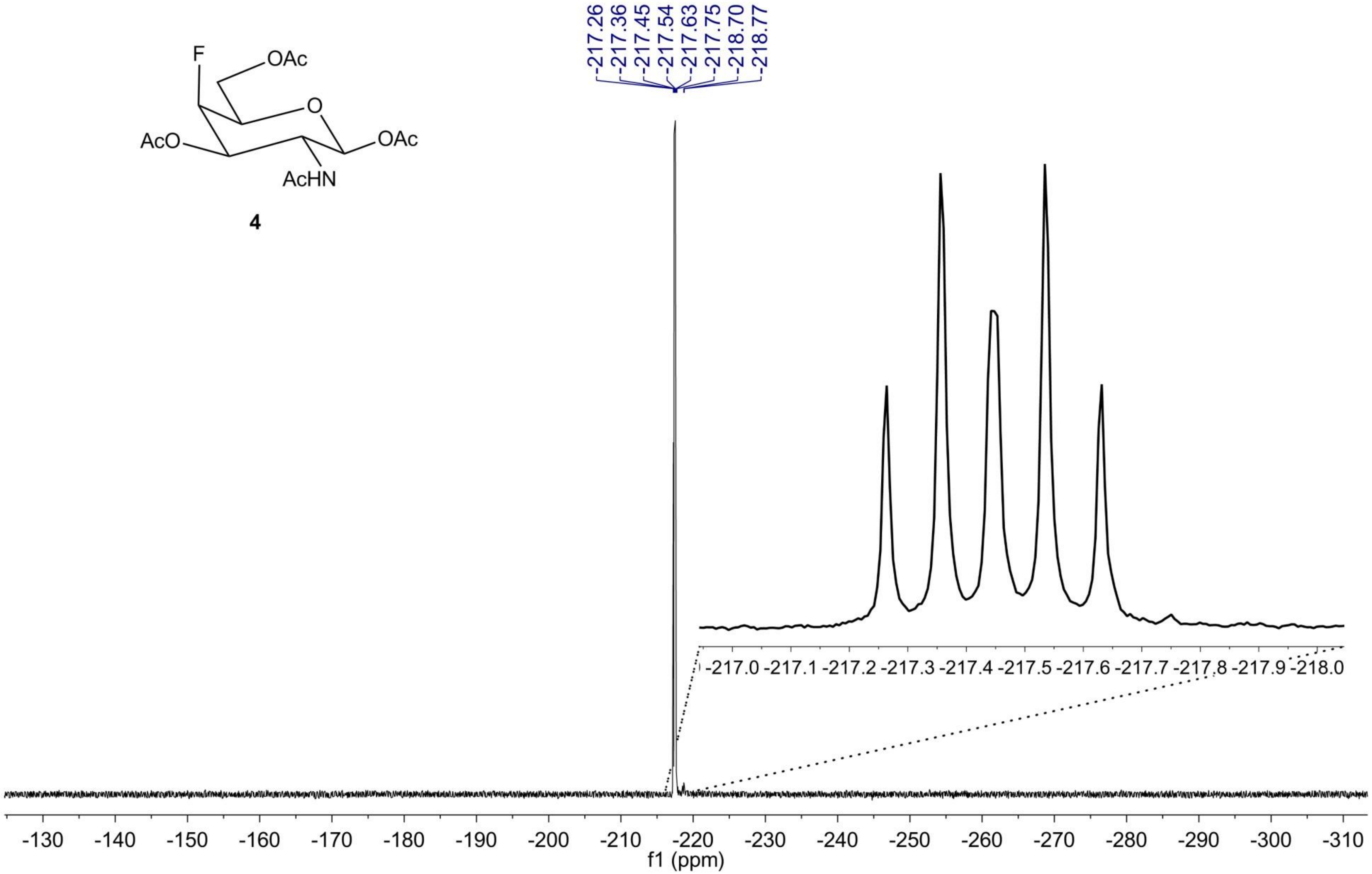

^{19}F NMR (282 MHz, $CDCl_3$) of **4β** (β-anomer).

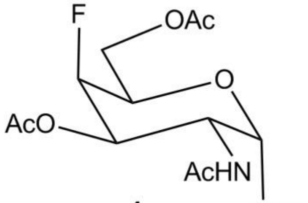

4

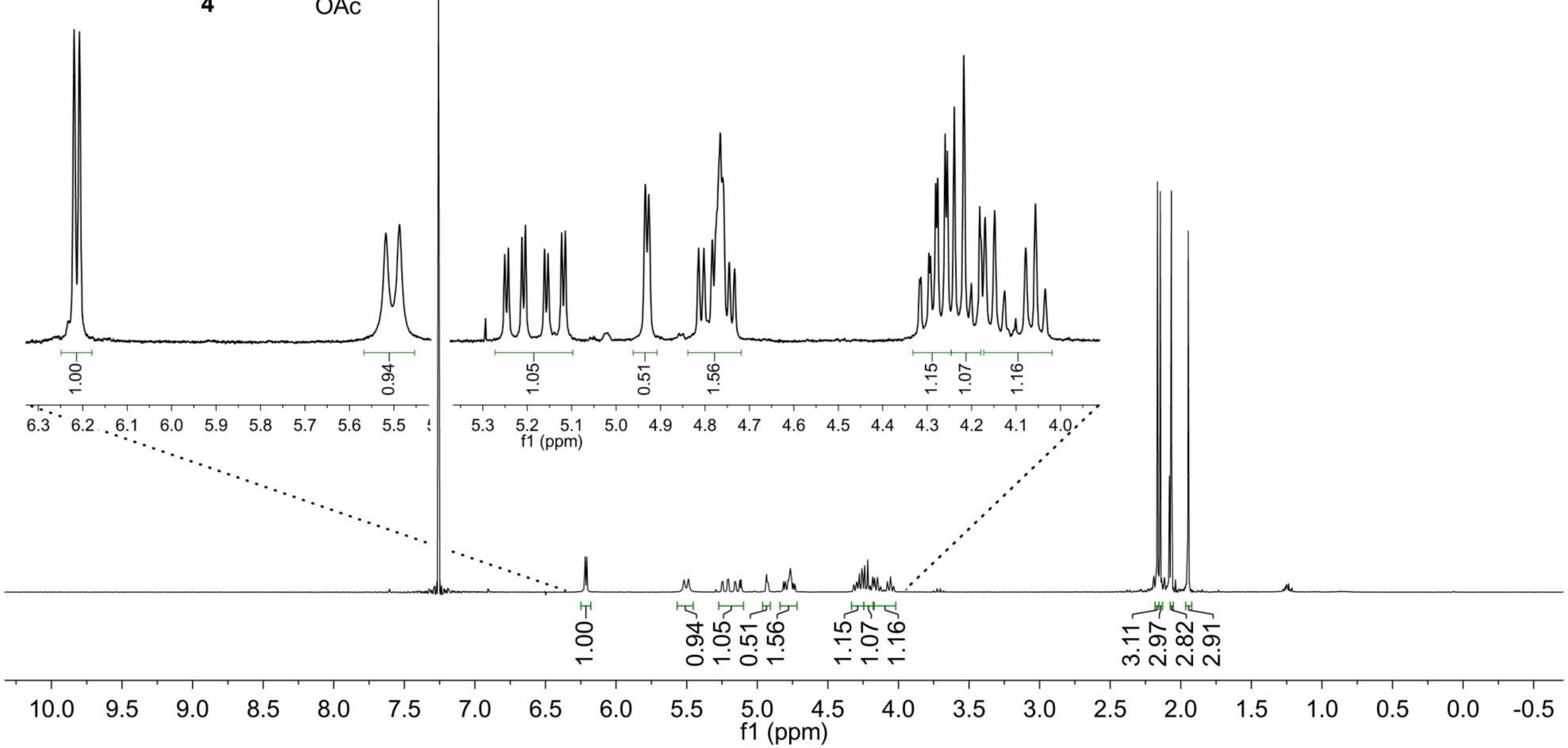

^{1}H NMR (300 MHz, $CDCl_3$) of **4α** (α-anomer).

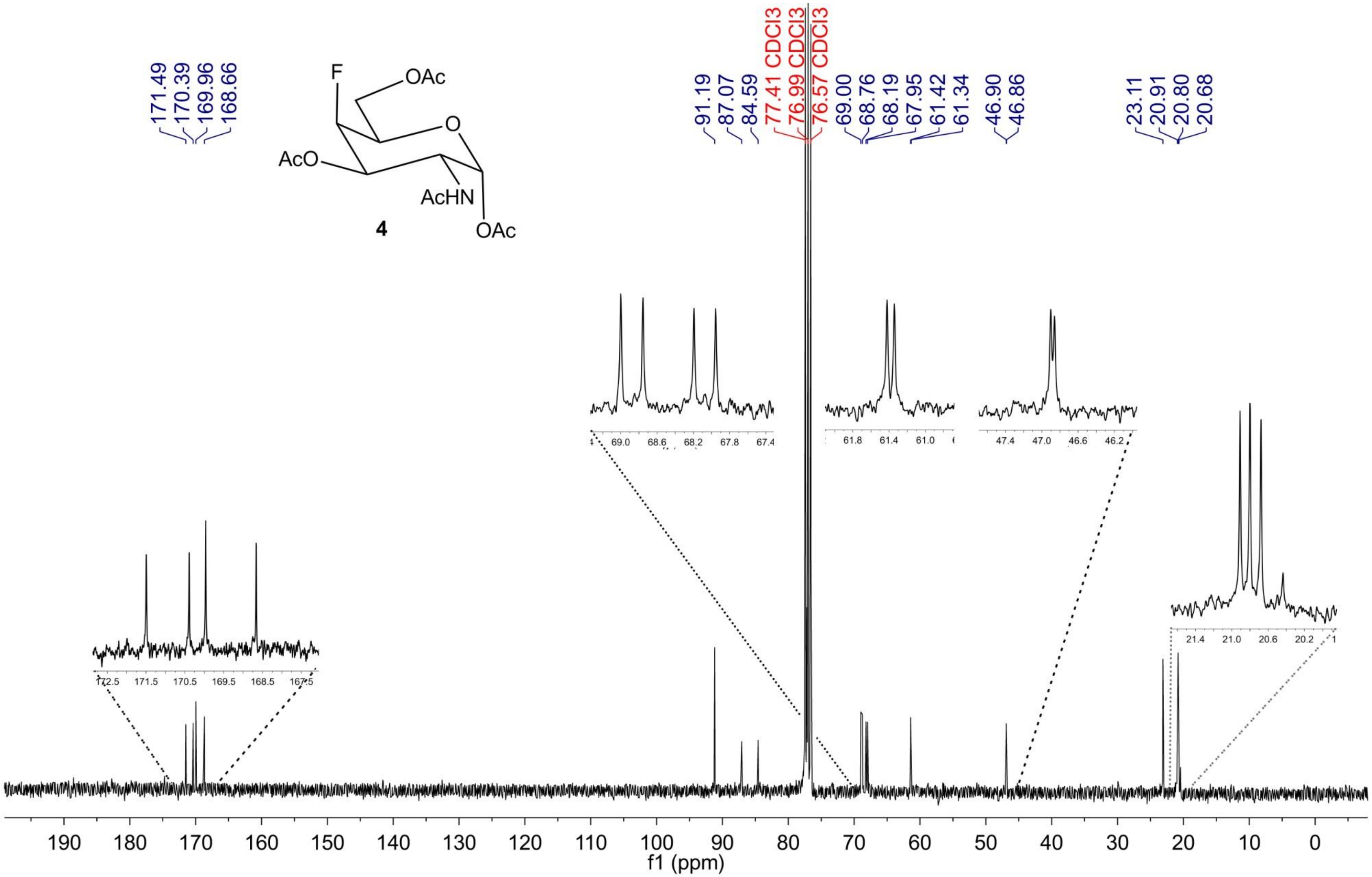

^{13}C {^{1}H} NMR (75 MHz, $CDCl_3$) of **4α** (α-anomer).

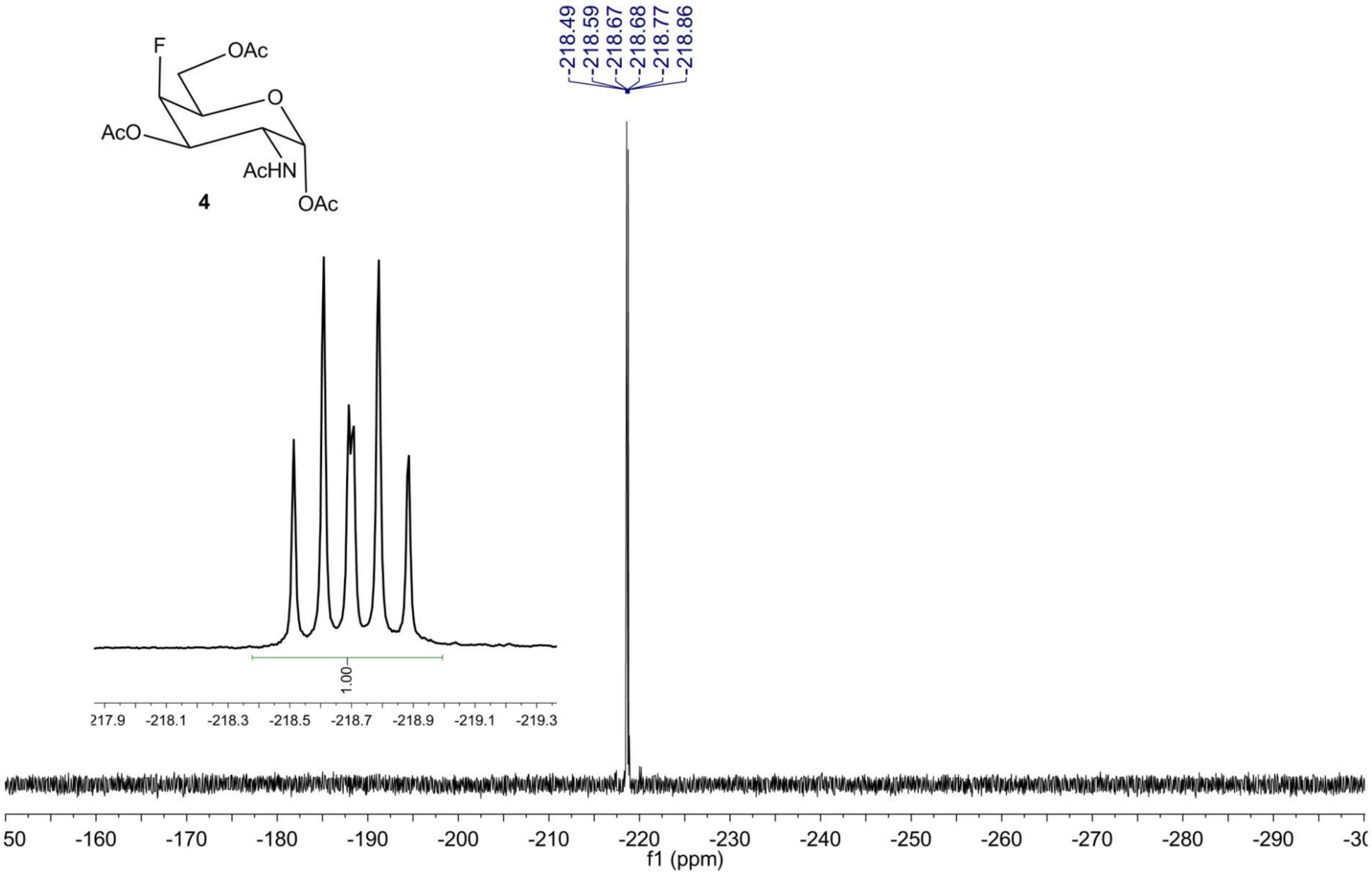

^{19}F NMR (282 MHz, $CDCl_3$) of **4α** (α-anomer).

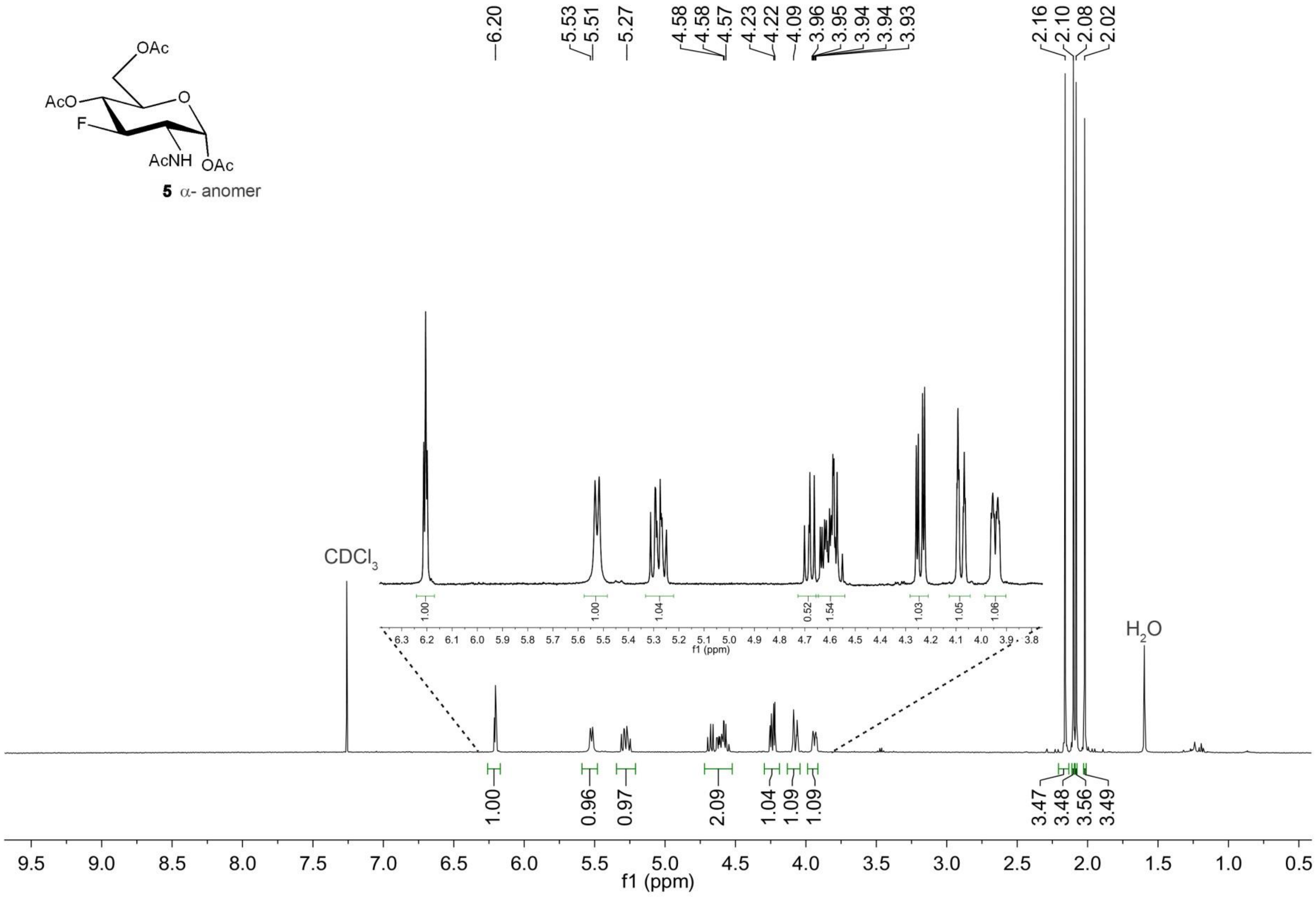

^{1}H NMR (500 MHz, $CDCl_3$) of **5α** (α-anomer).

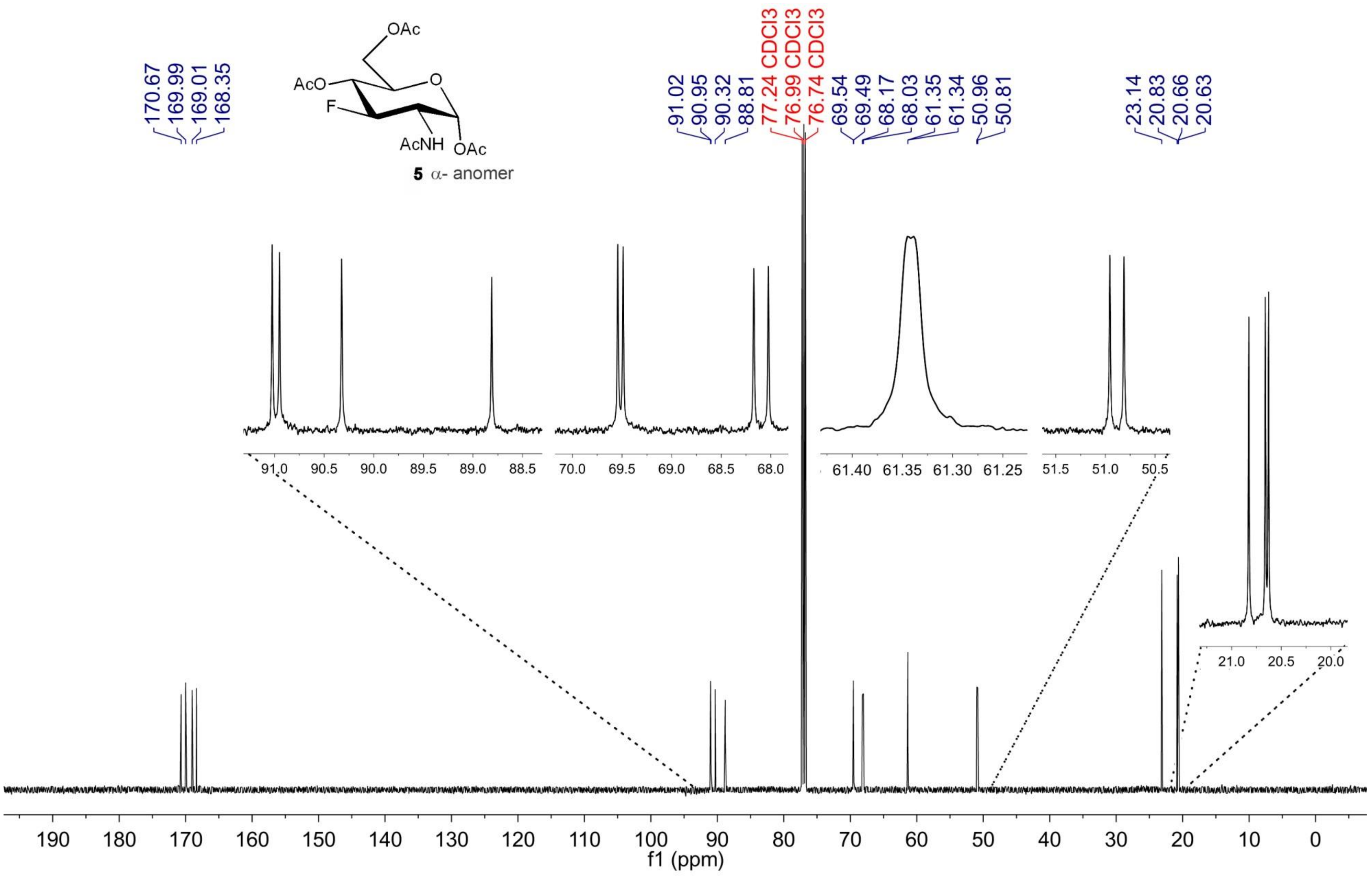

^{13}C {^{1}H} NMR (125 MHz, $CDCl_3$) of **5α** (α-anomer).

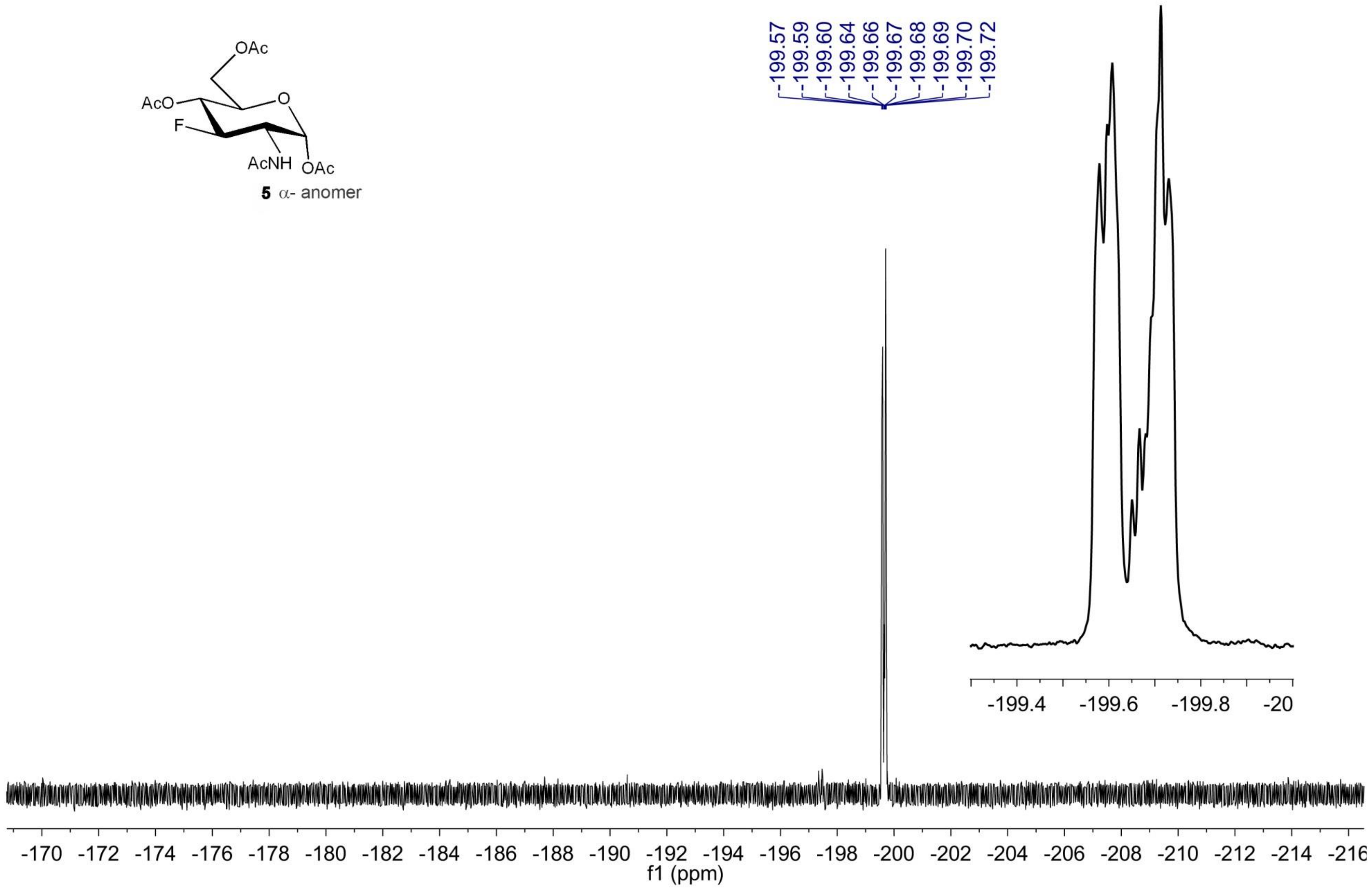

^{19}F NMR (470 MHz, $CDCl_3$) of **5α** (α-anomer).

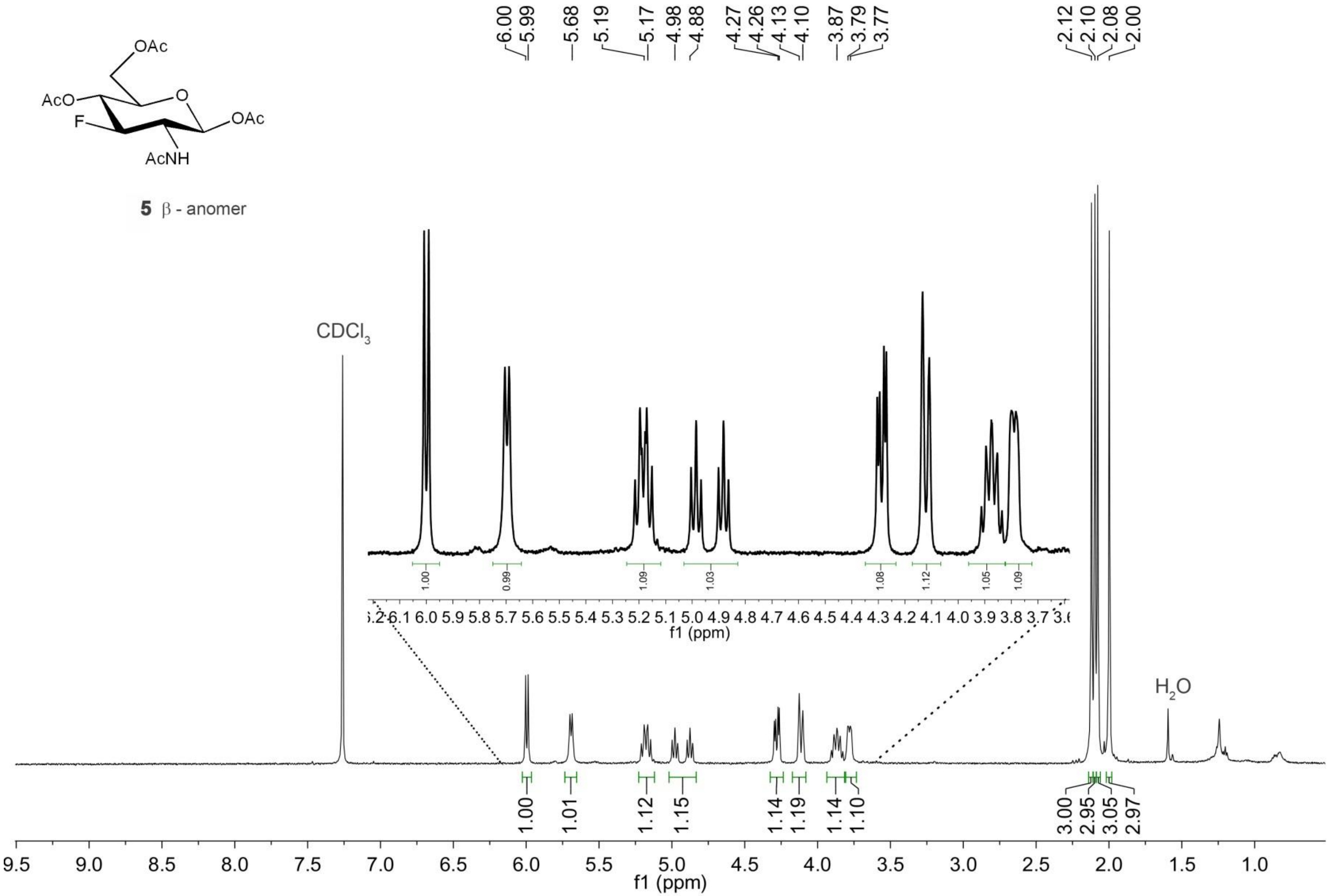

^{1}H NMR (300 MHz, $CDCl_3$) of **5β** (β-anomer).

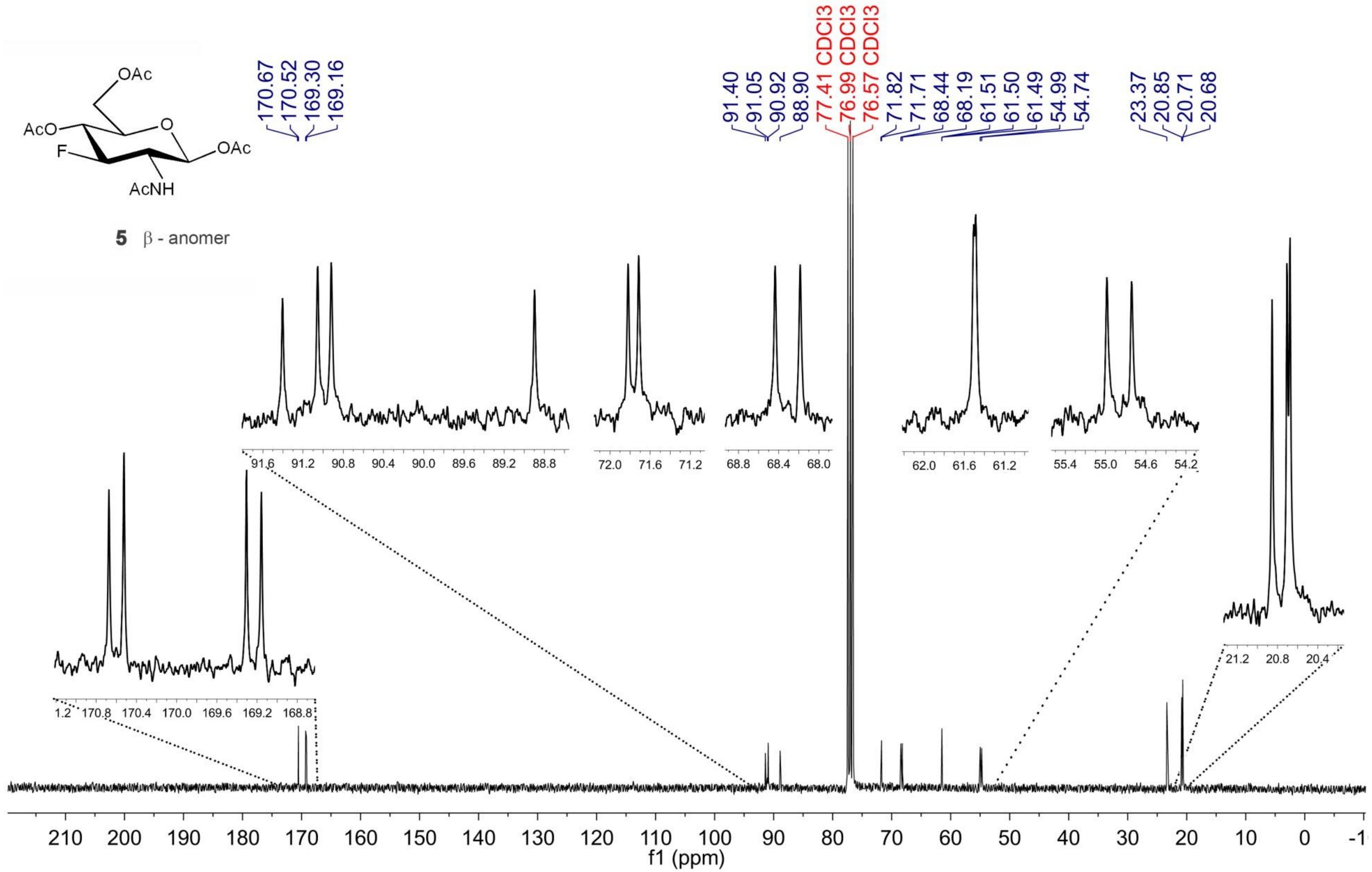

^{13}C {^{1}H} NMR (75 MHz, $CDCl_3$) of **5β** (β-anomer).

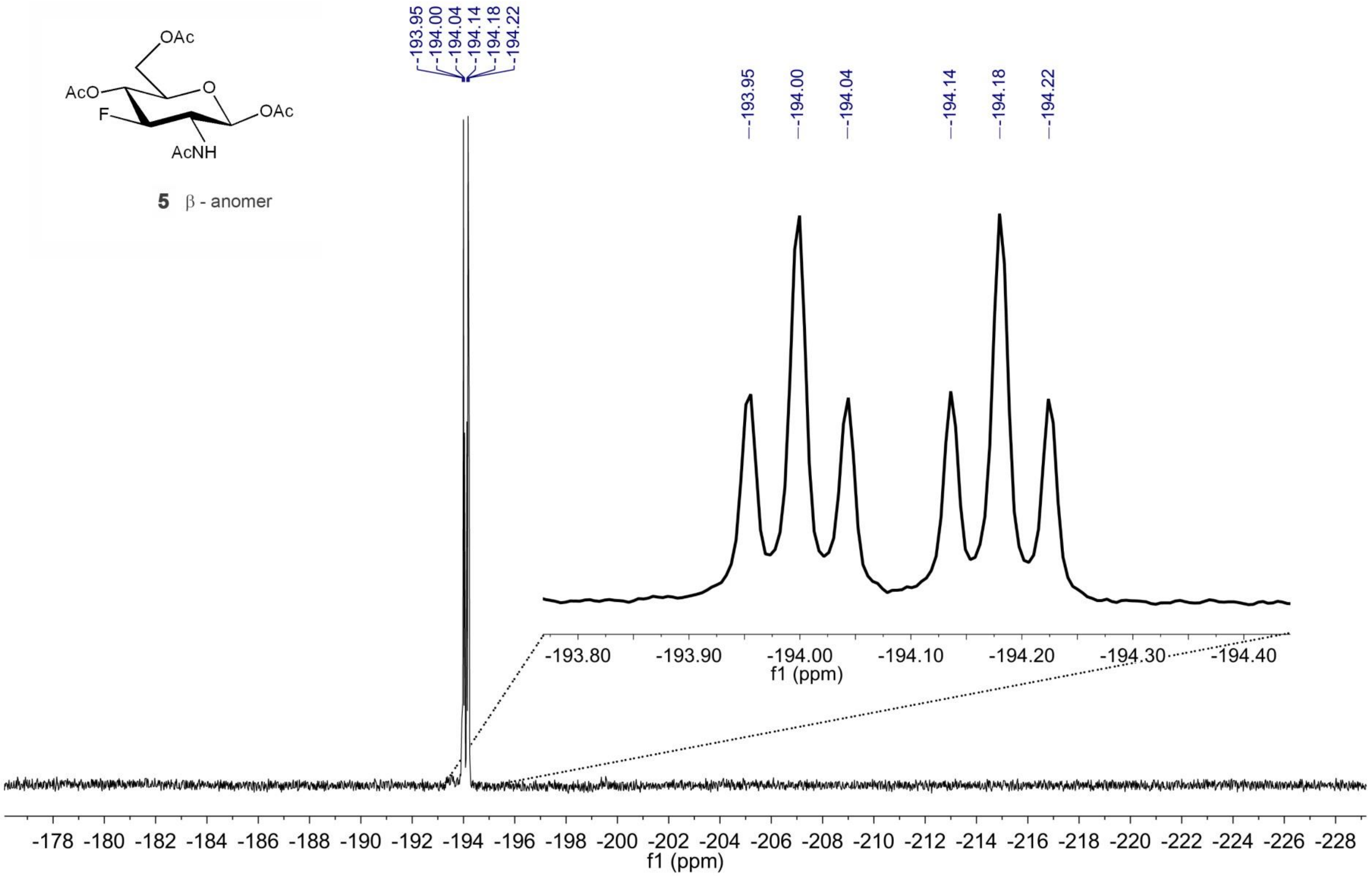

^{19}F NMR (282 MHz, $CDCl_3$) of **5β** (β-anomer).

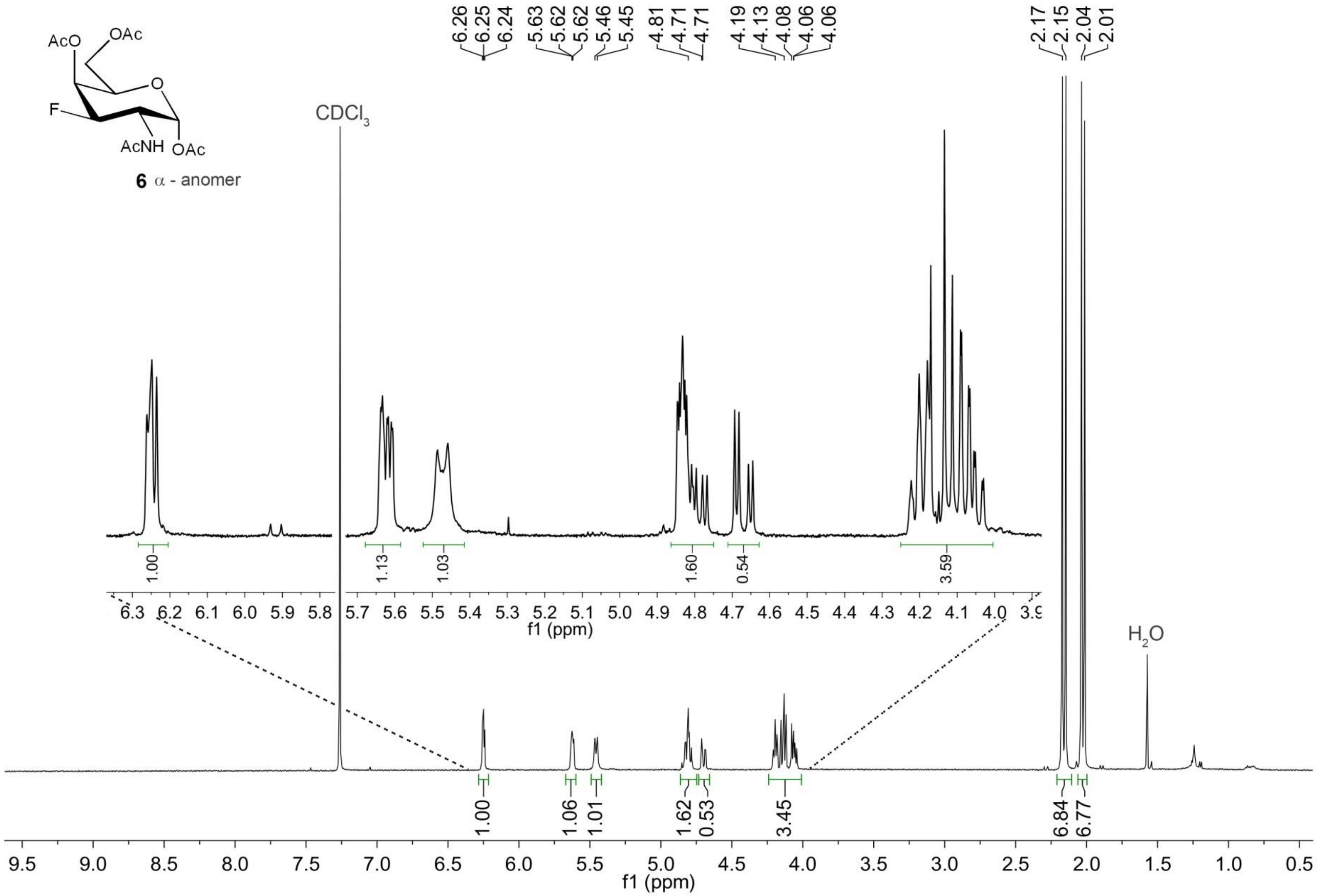

^{1}H NMR (300 MHz, $CDCl_3$) of **6** (α-anomer).

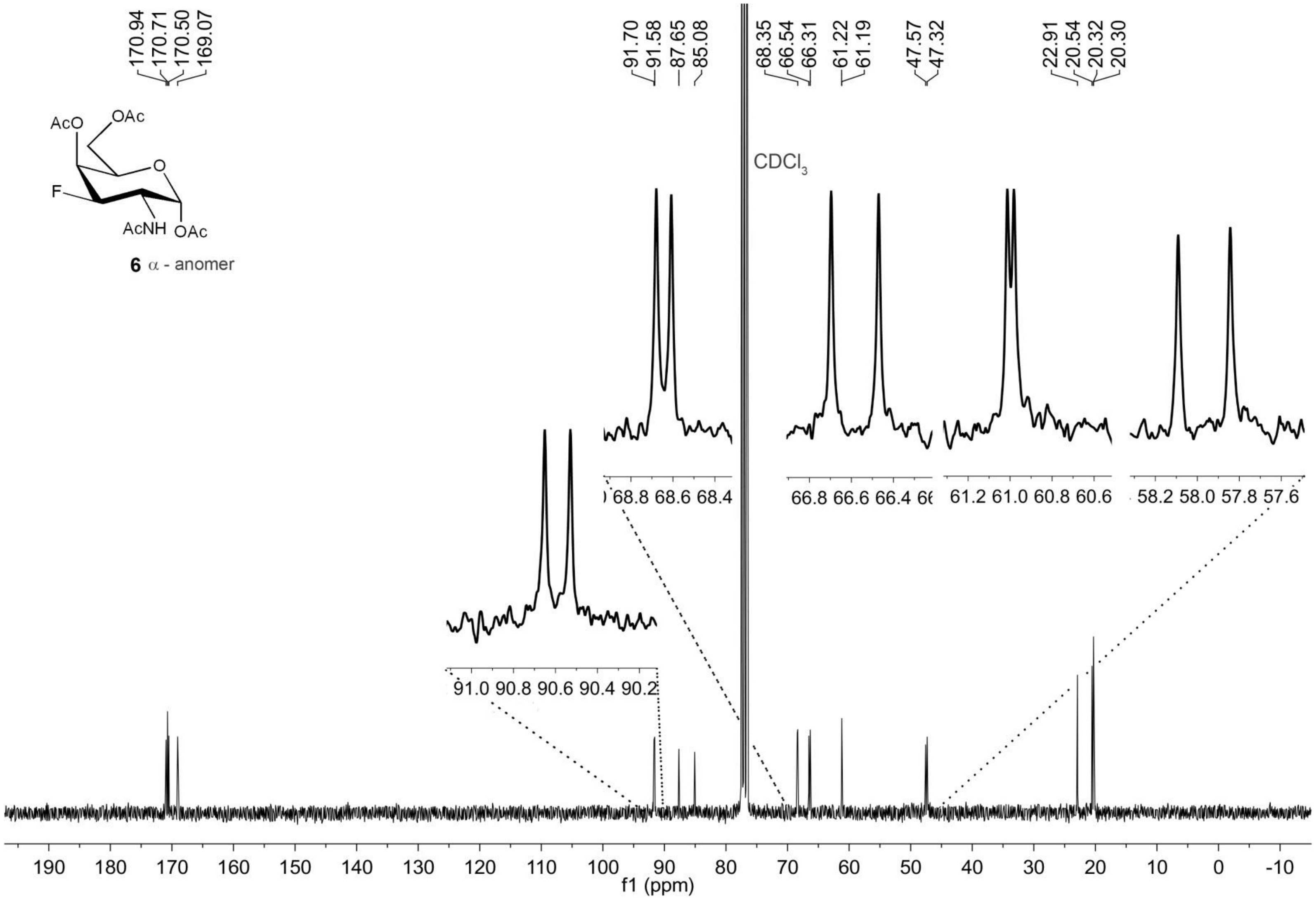

^{13}C {^{1}H} NMR (75 MHz, $CDCl_3$) of **6** (α-anomer).

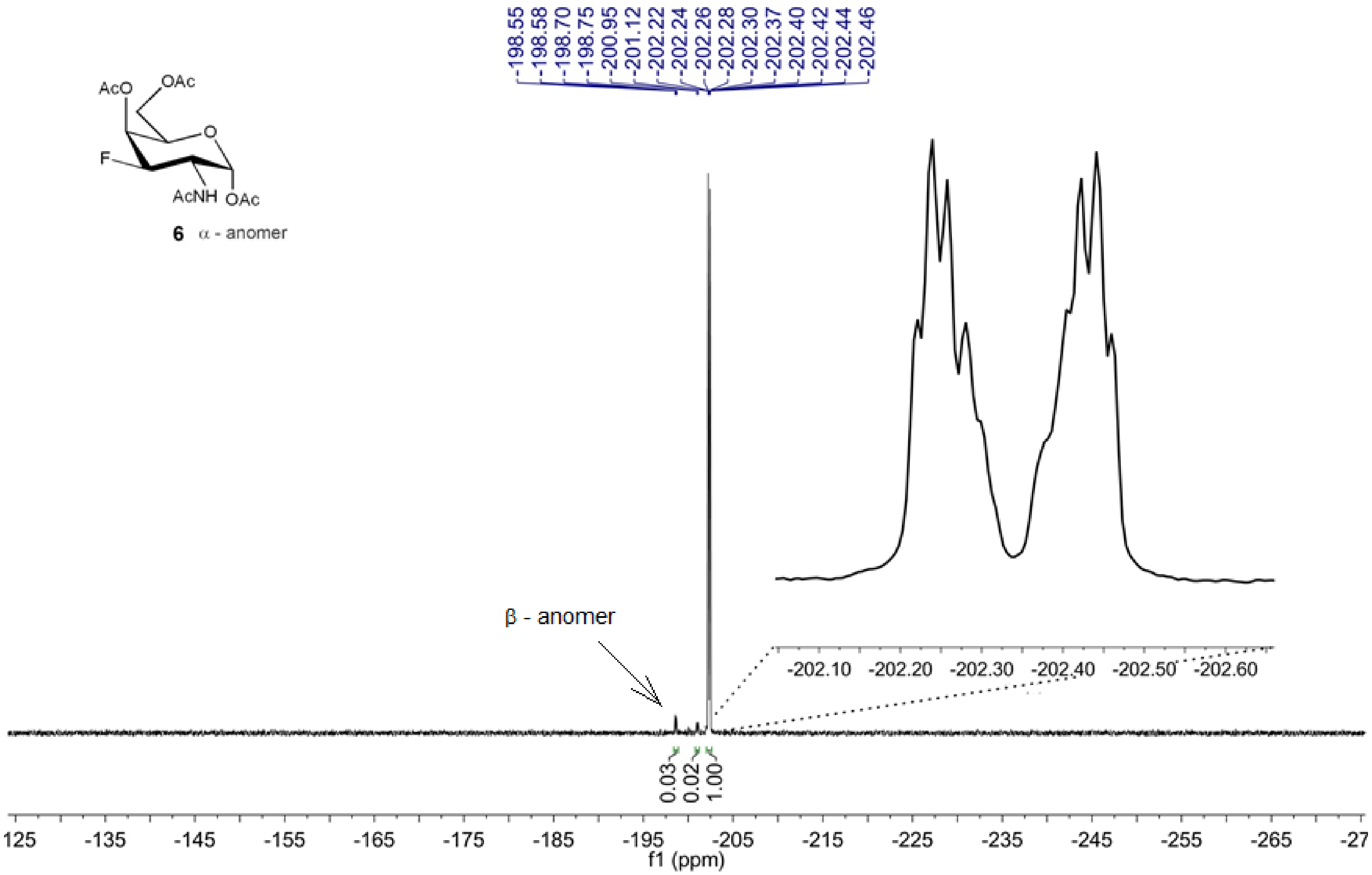

^{19}F NMR (282 MHz, $CDCl_3$) of **6** (α-anomer + cca 3 % β-anomer).

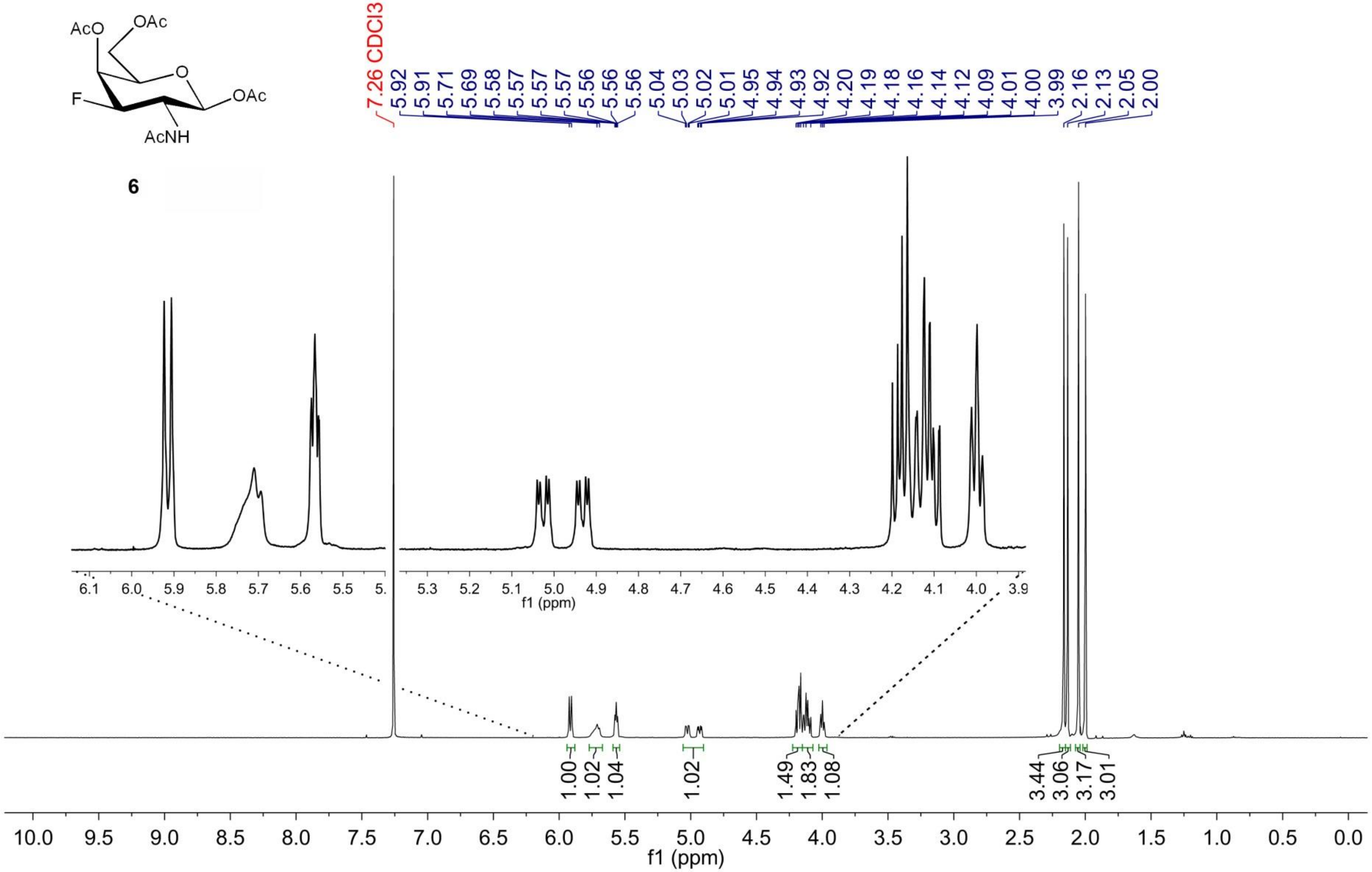

^{1}H NMR (500 MHz, $CDCl_3$) of **6** (β-anomer).

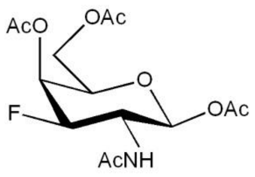

6

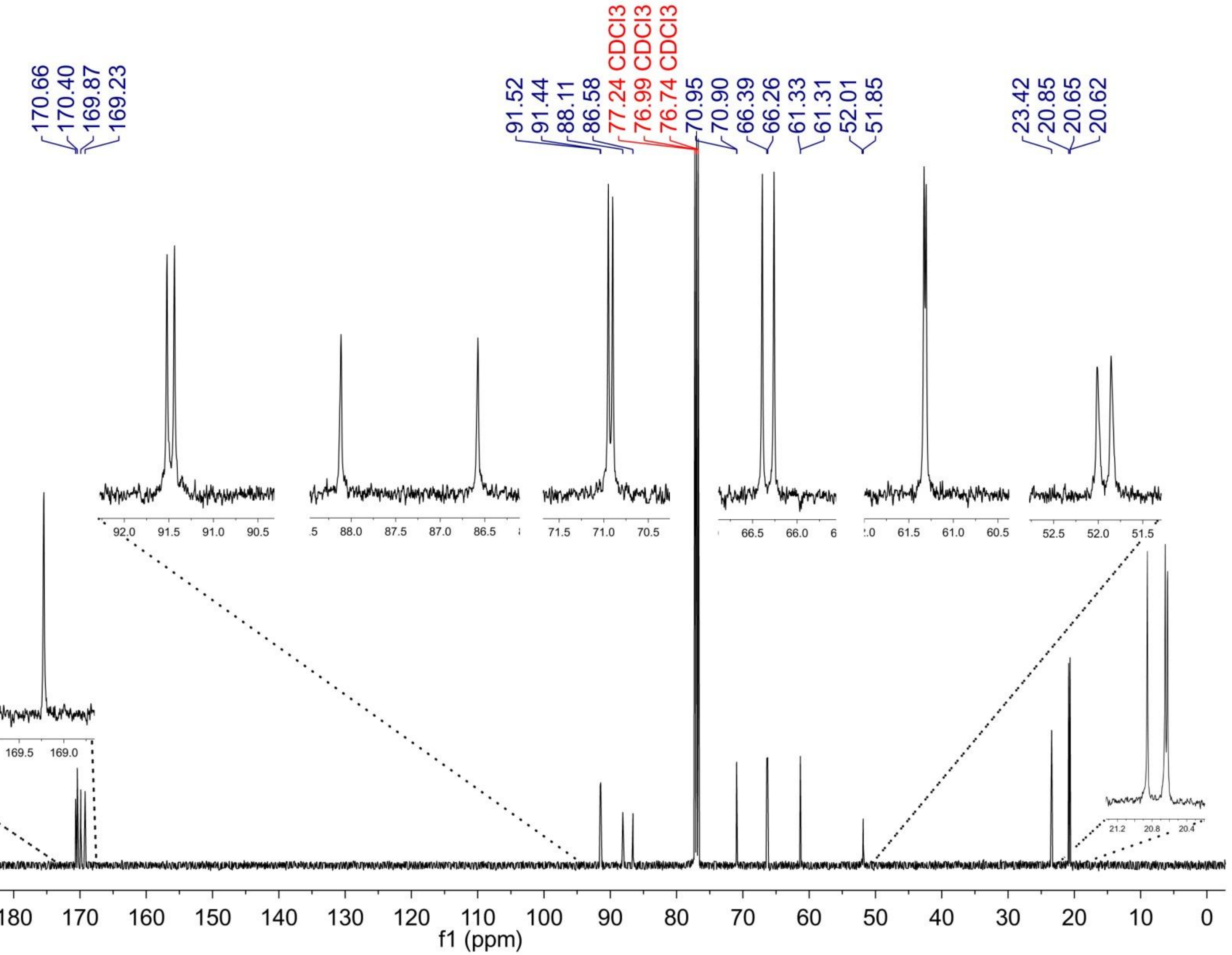

^{13}C {^{1}H} NMR (125 MHz, $CDCl_3$) of **6** (β-anomer).

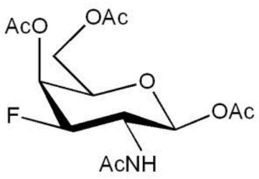

6

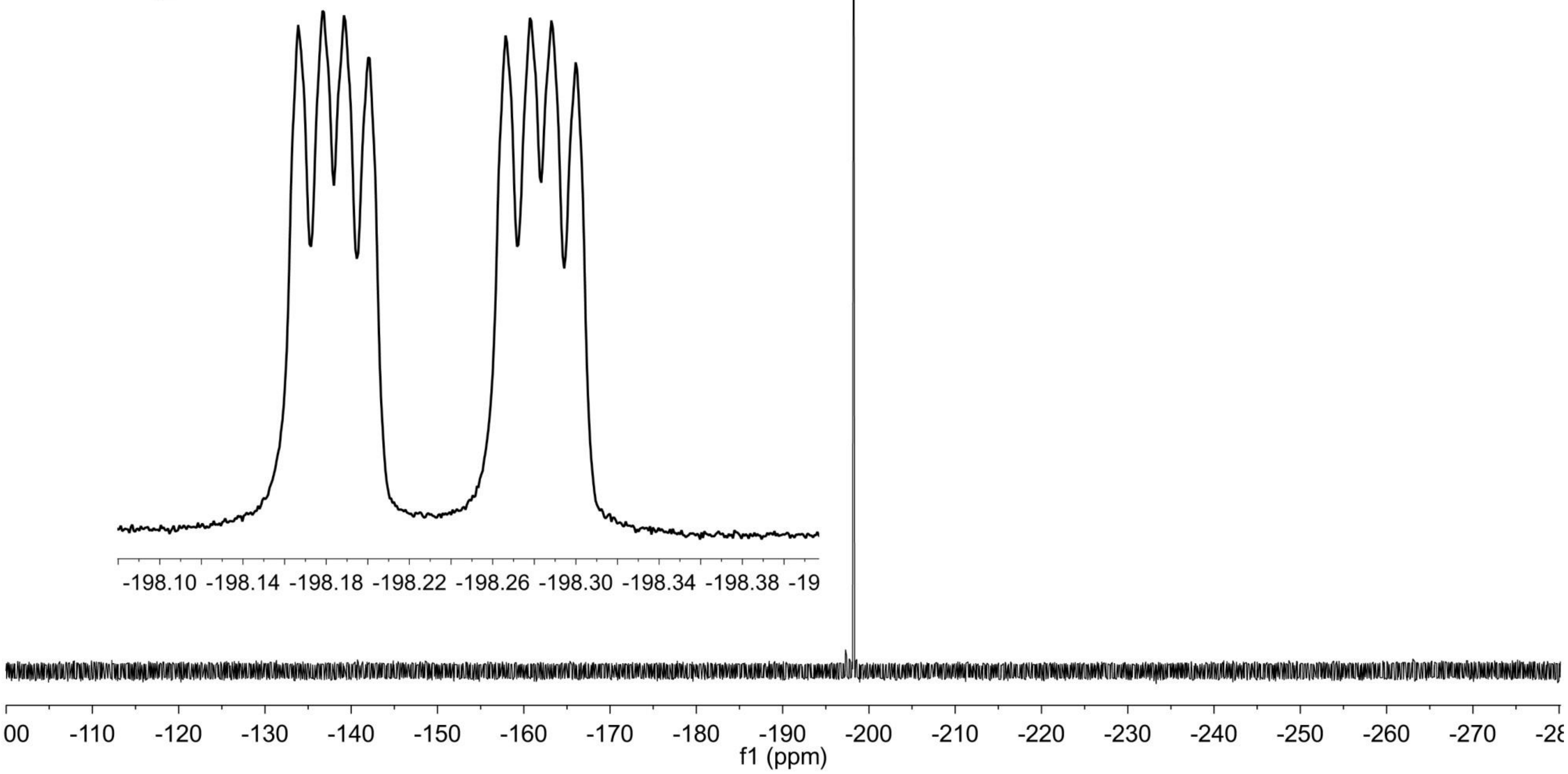

^{19}F NMR (470 MHz, $CDCl_3$) of **6** (β-anomer).

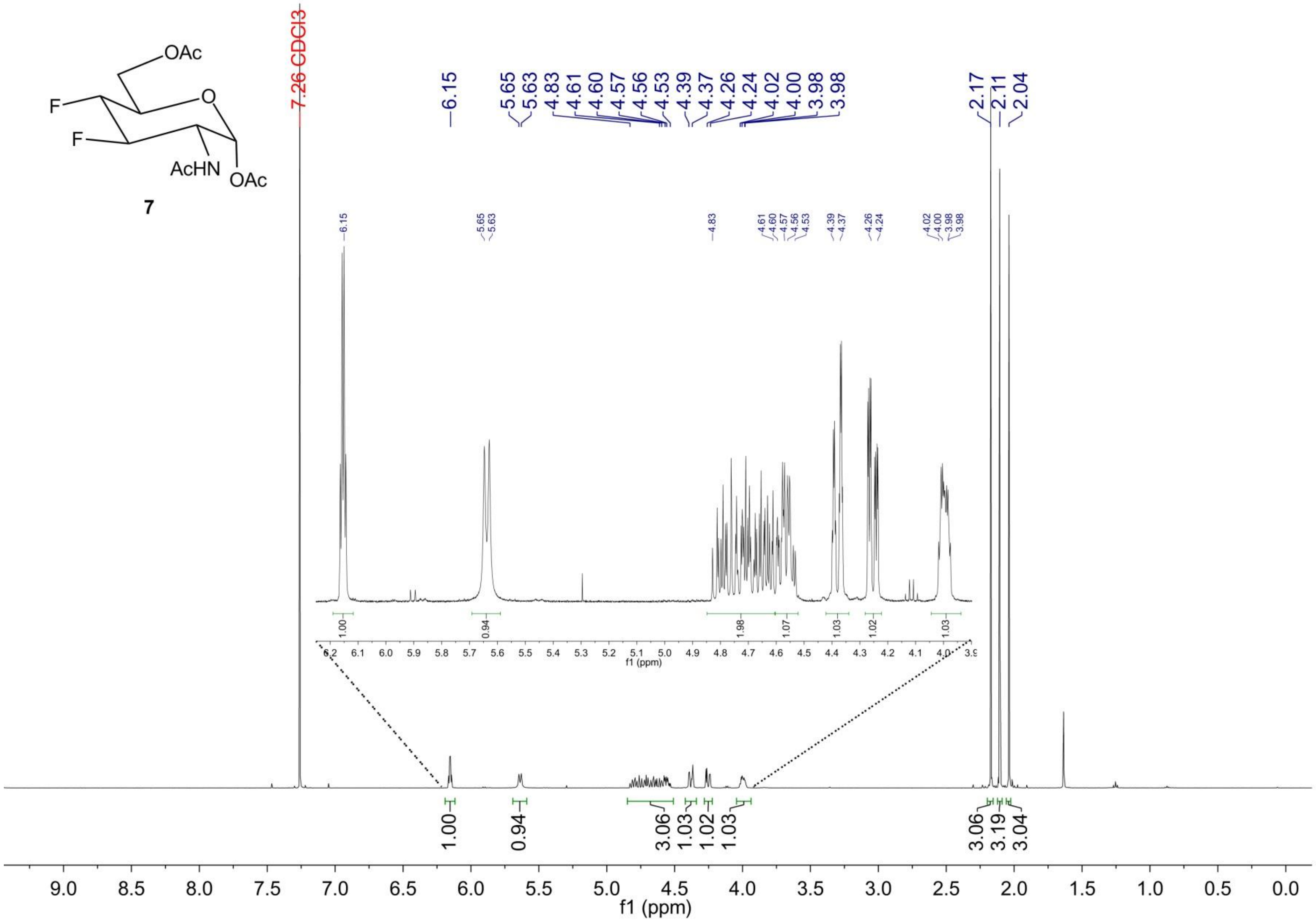

^{1}H NMR (500 MHz, $CDCl_3$) of **7** (α-anomer).

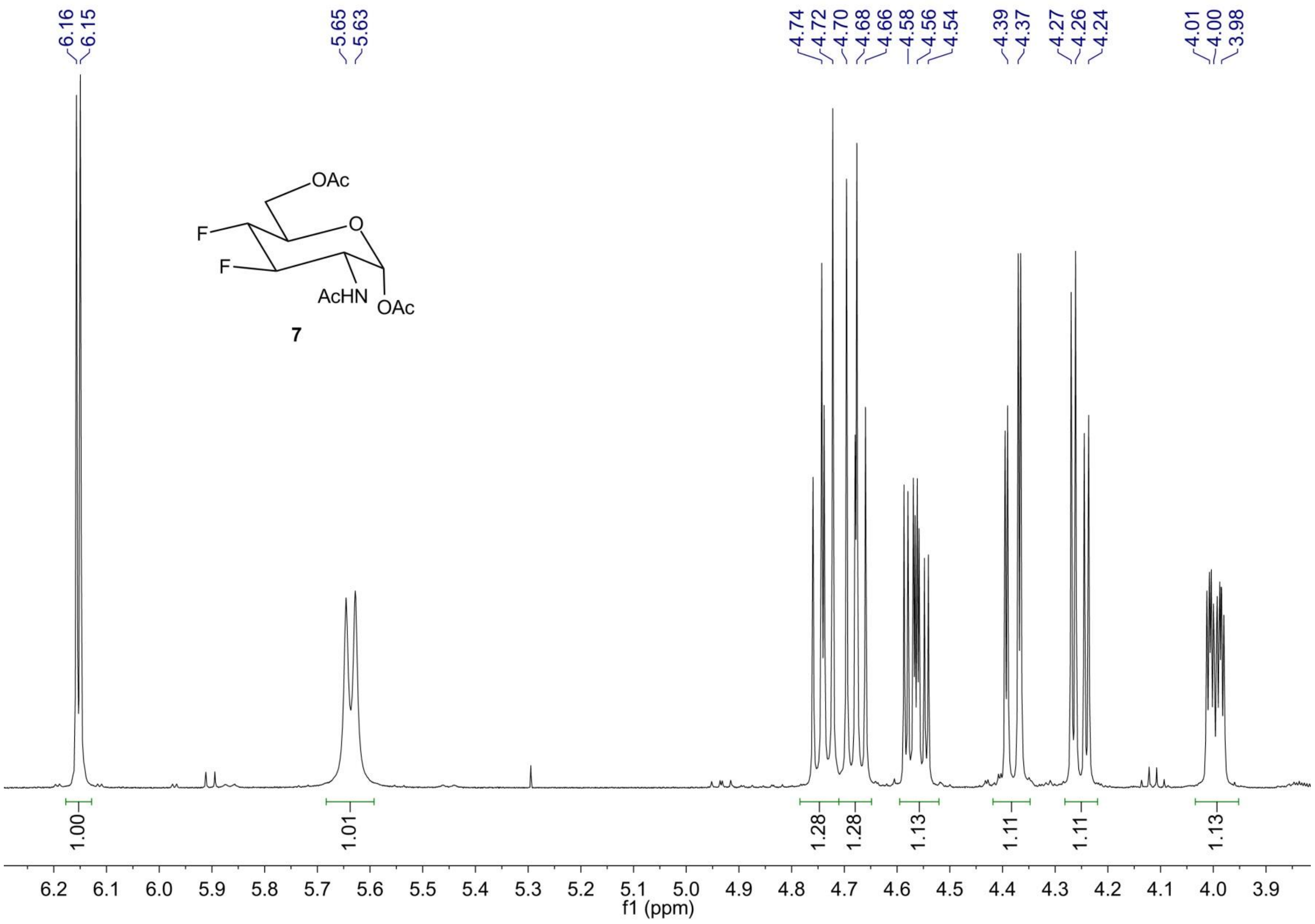

Detail of $^{1}H\ \{^{19}F\}$ NMR (500 MHz, $CDCl_3$) of **7** (α-anomer).

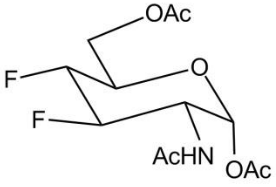

7

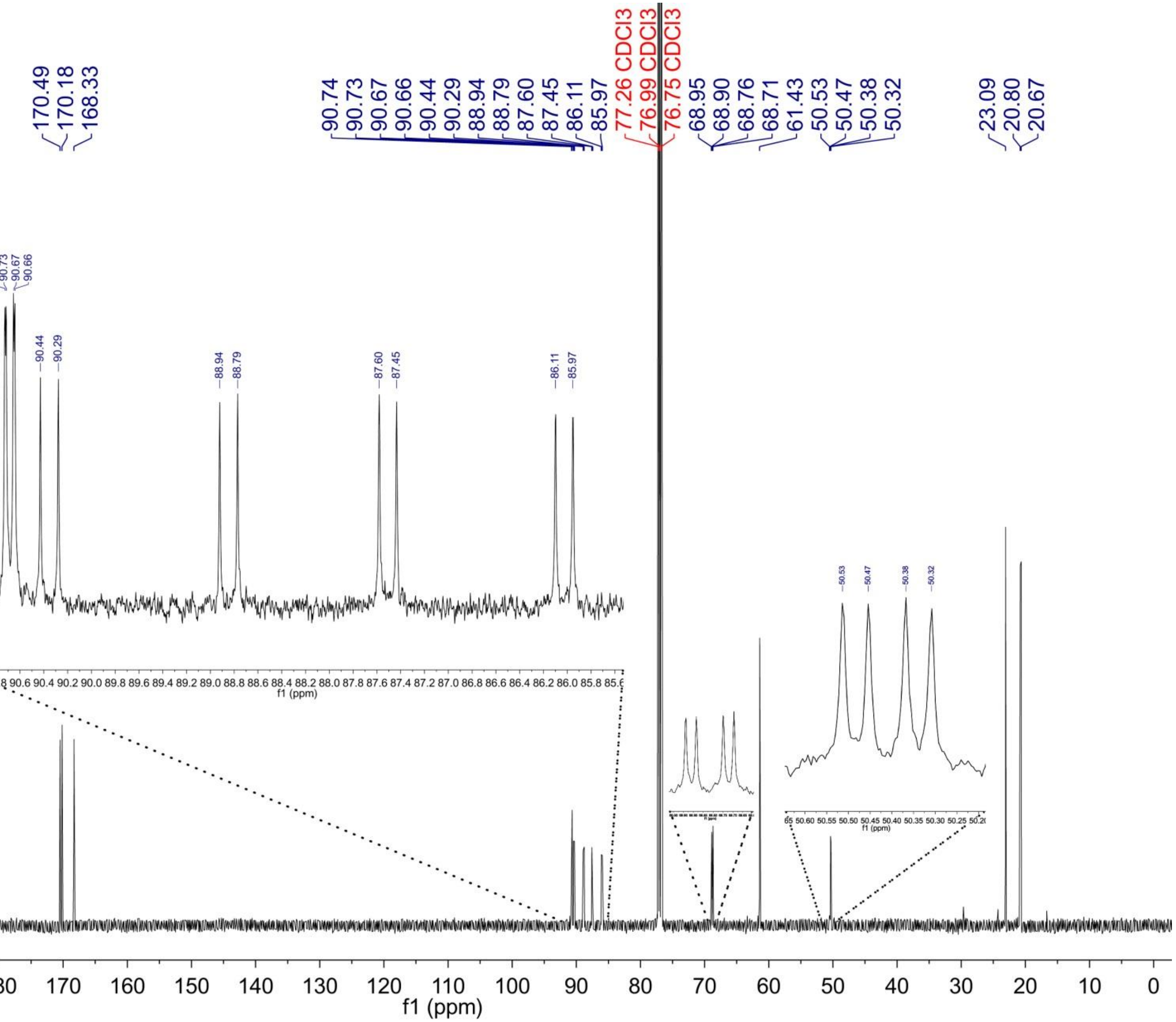

$^{13}C\ \{^{1}H\}$ NMR (125 MHz, $CDCl_3$) of **7** (α-anomer).

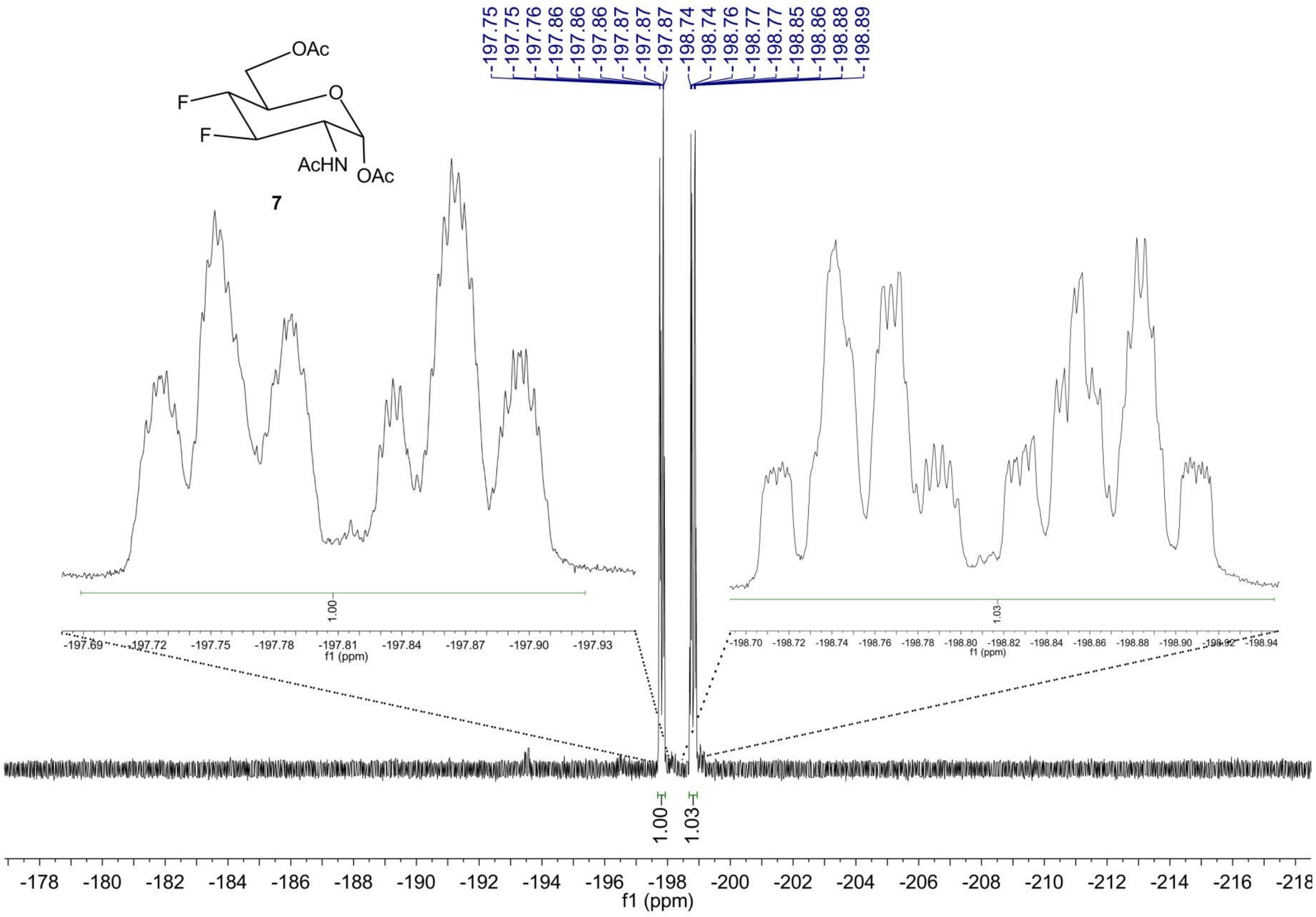

^{19}F NMR (470 MHz, $CDCl_3$) of **7** (α-anomer).

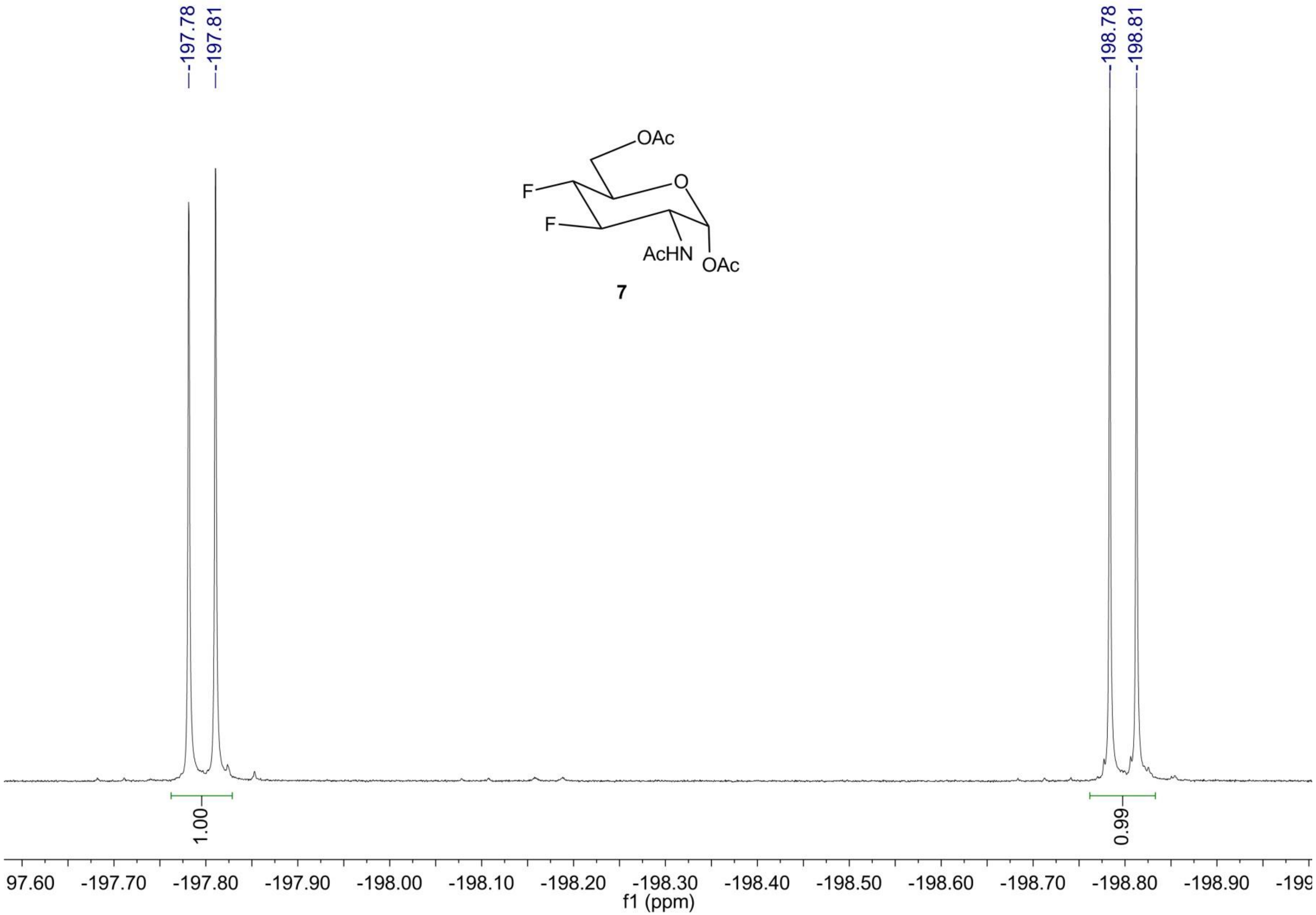

^{19}F {^{1}H} NMR (470 MHz, $CDCl_3$) of **7** (α-anomer).

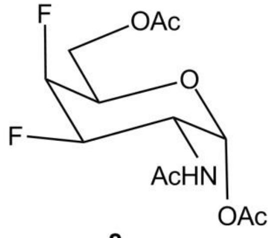

8

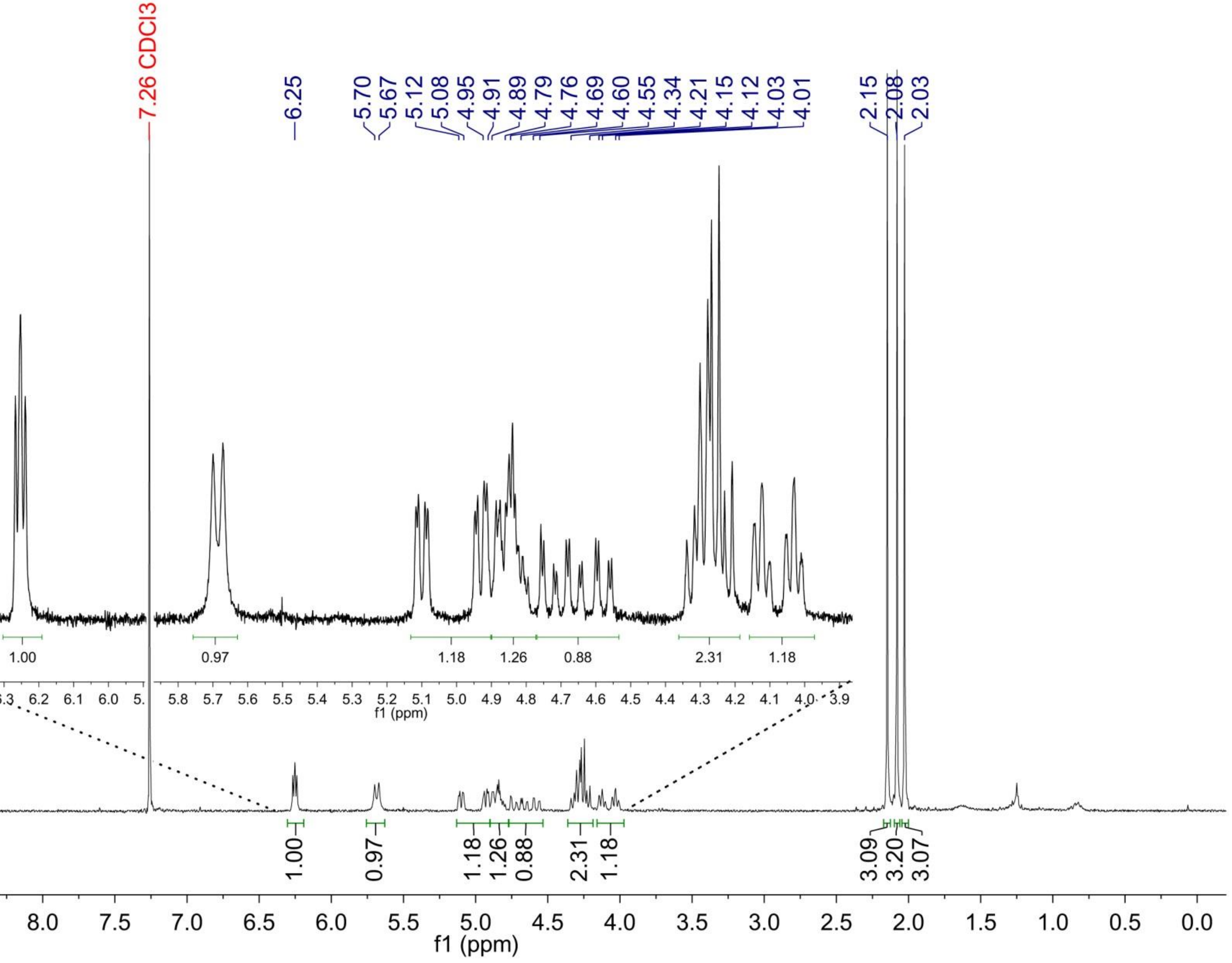

^{1}H NMR (300 MHz, $CDCl_3$) of **8** (α-anomer).

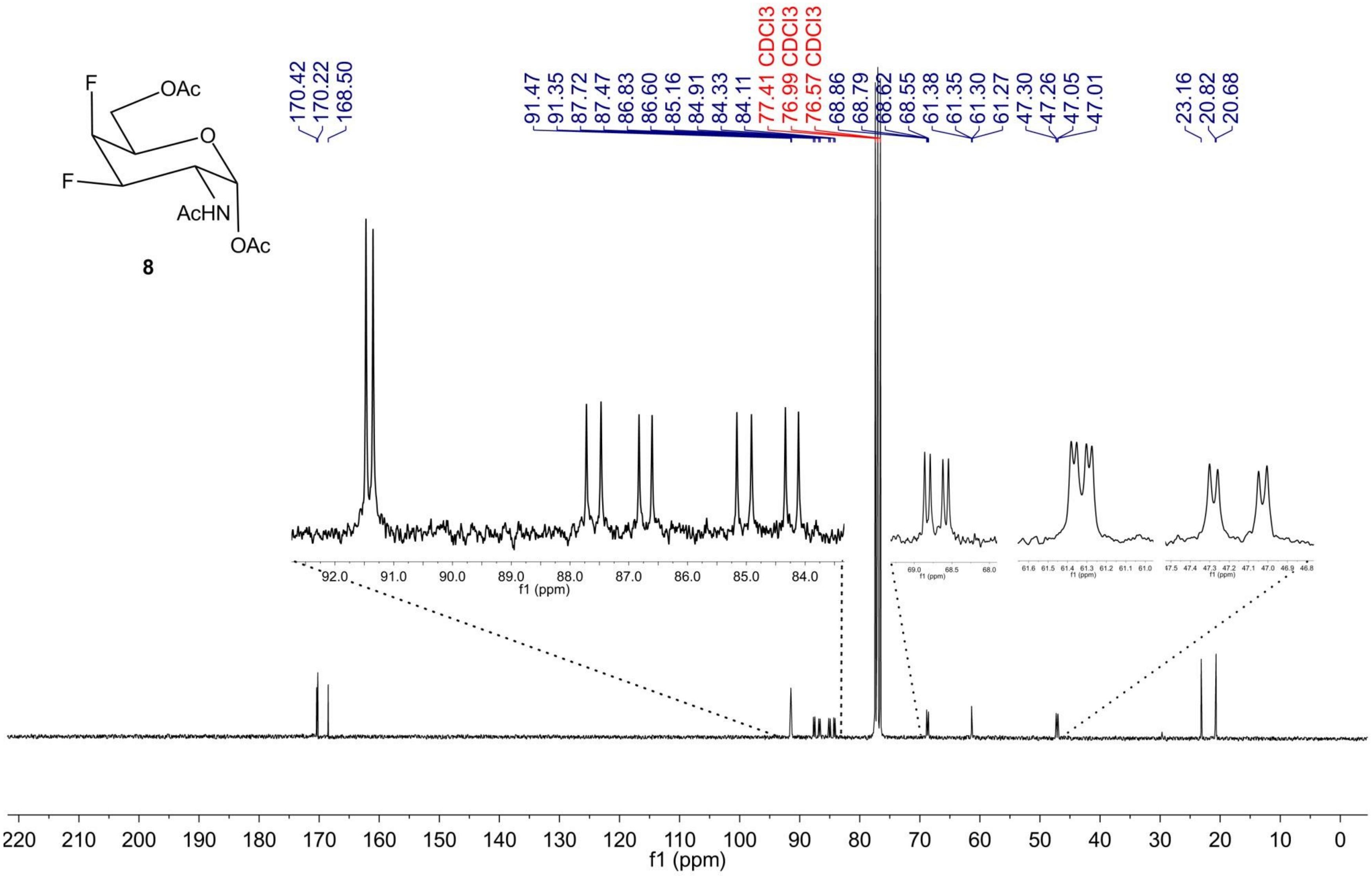

^{13}C {^{1}H} NMR (75 MHz, $CDCl_3$) of **8** (α-anomer).

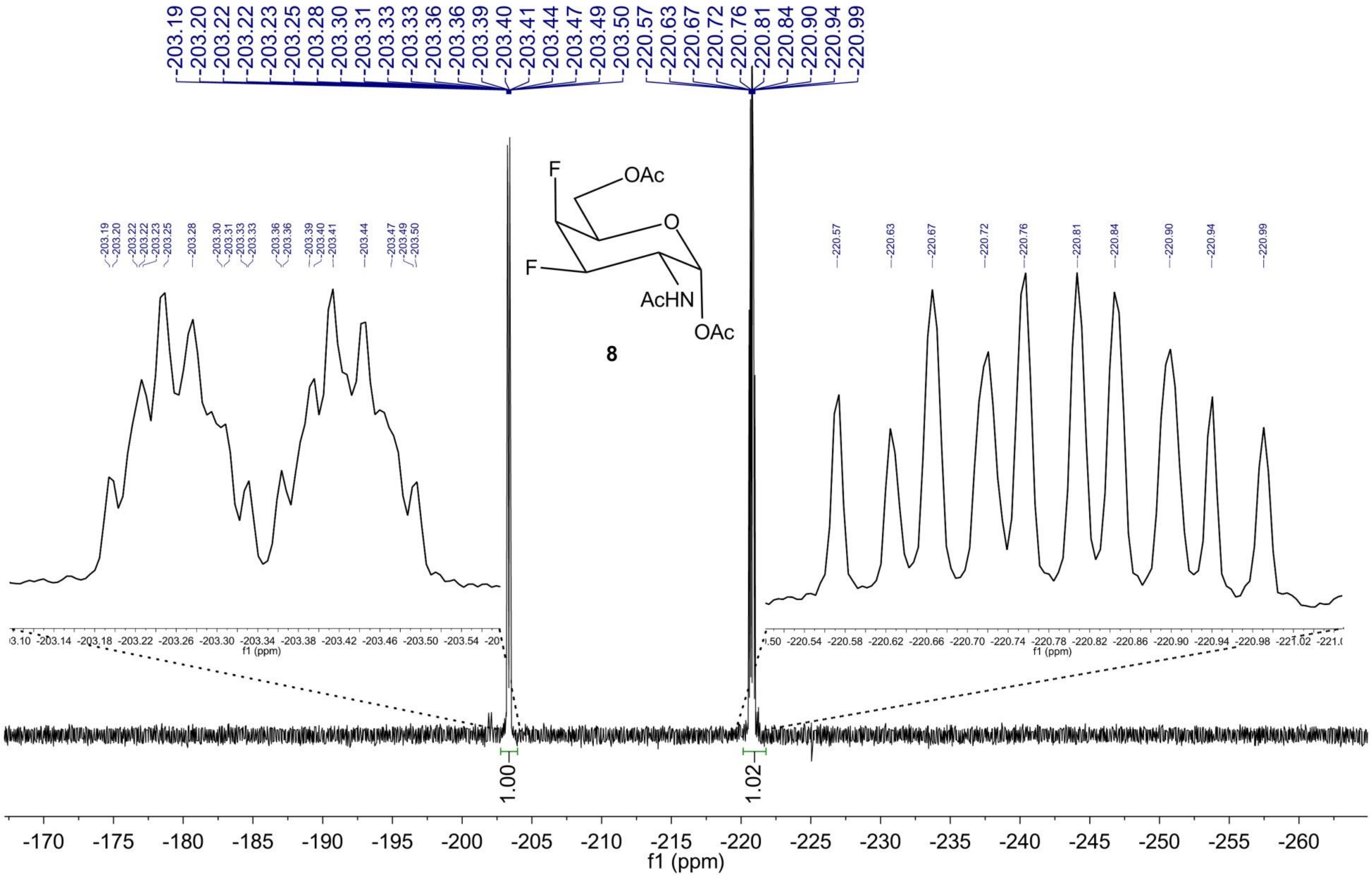

^{19}F NMR (282 MHz, $CDCl_3$) of **8** (α-anomer)

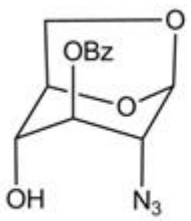

12

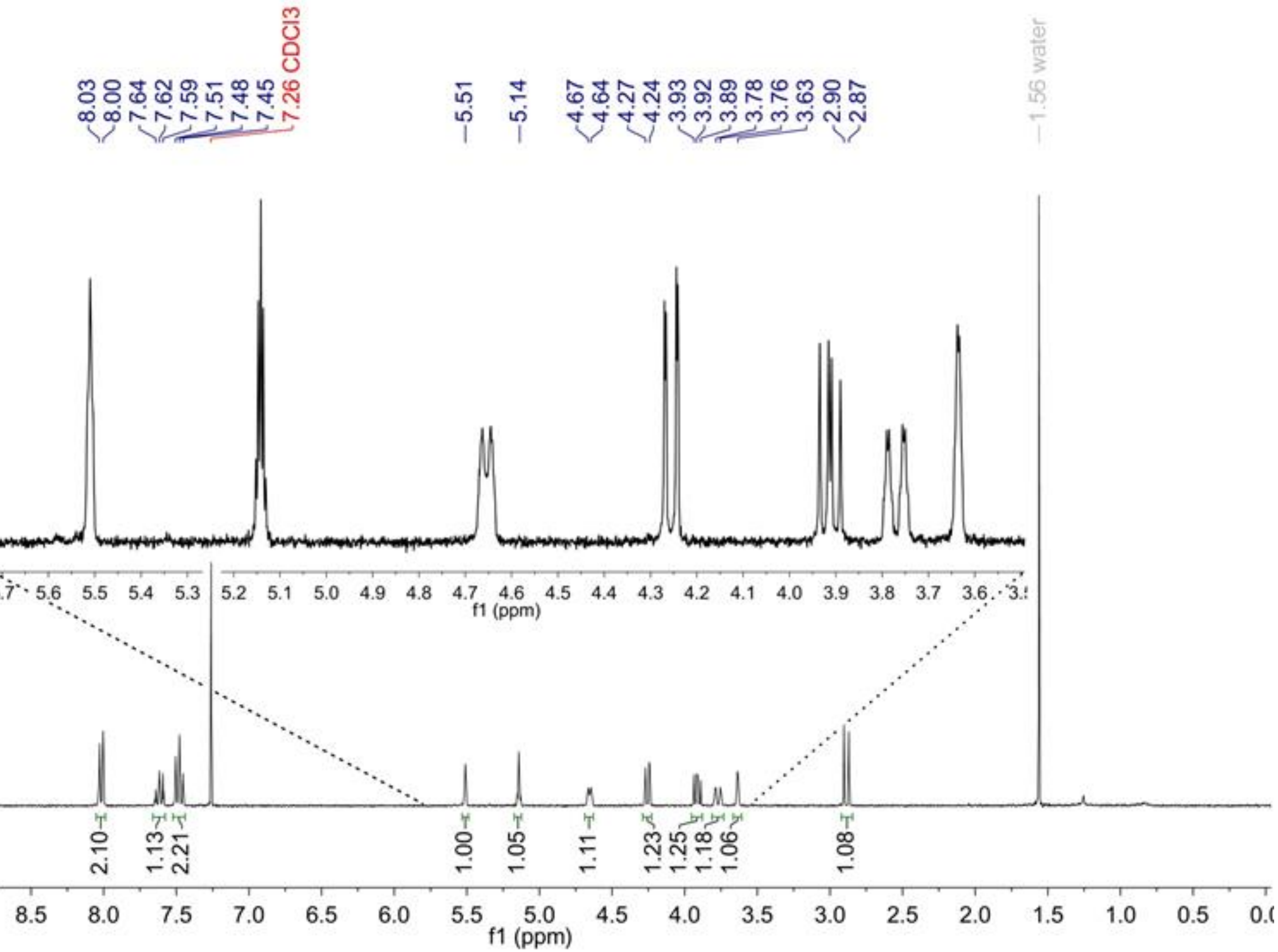

^{1}H NMR (500 MHz, $CDCl_3$) of **12**.

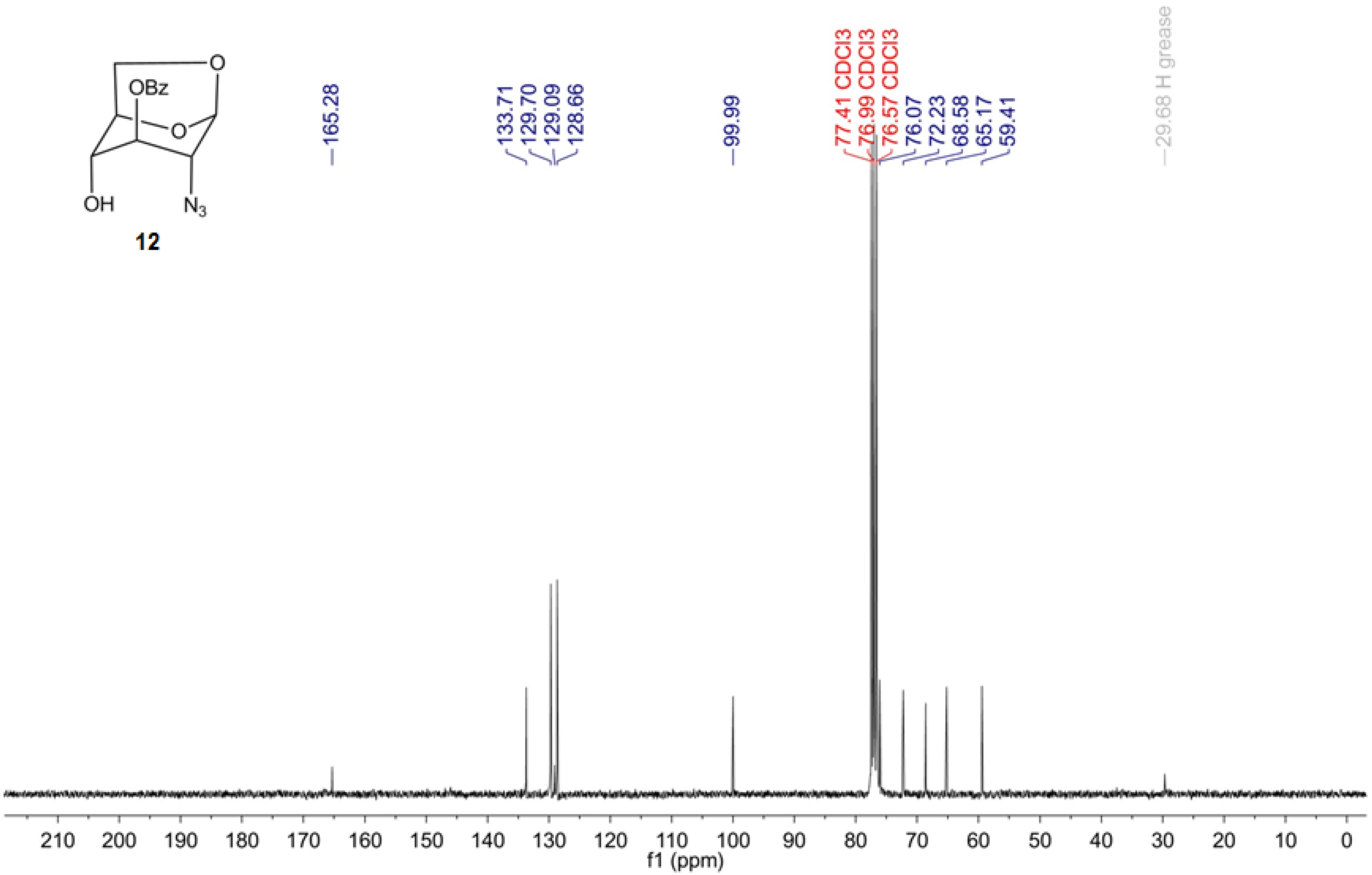

$^{13}C\ \{^{1}H\}$ NMR (125 MHz, $CDCl_3$) of **12**.

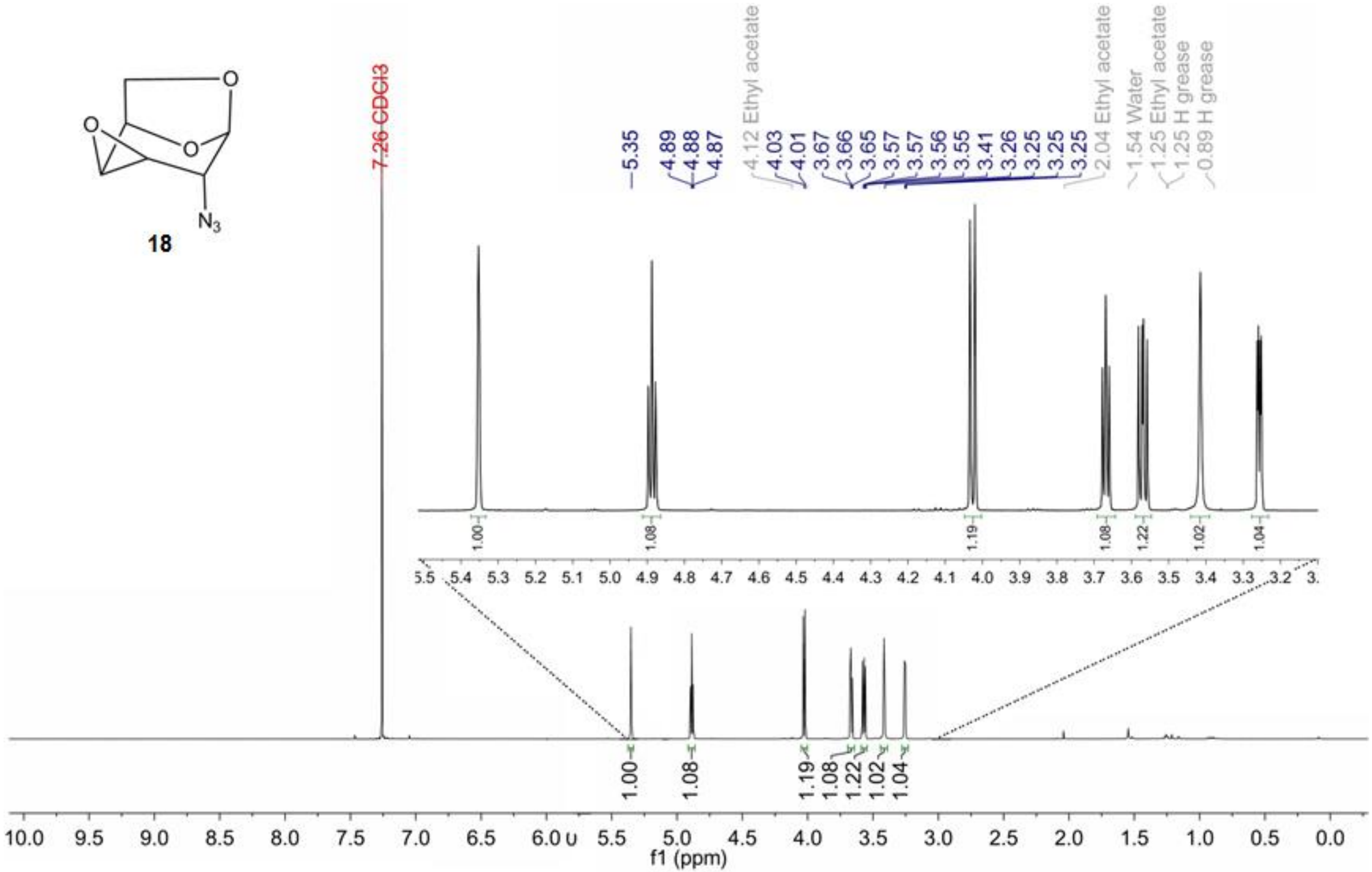

^{1}H NMR (500 MHz, $CDCl_3$) of **18**.

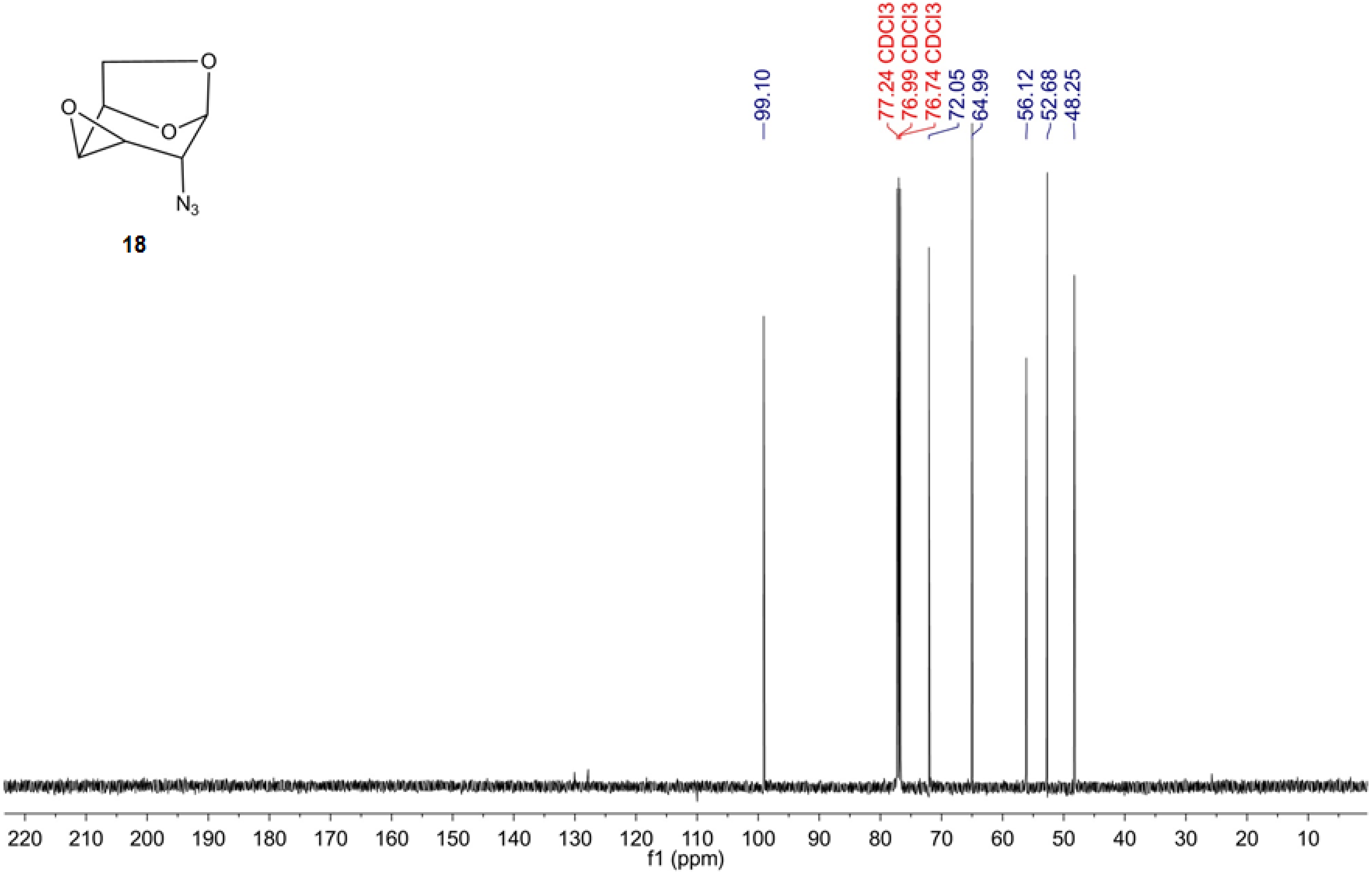
18

^{13}C NMR (125 MHz, $CDCl_3$) of **18**.

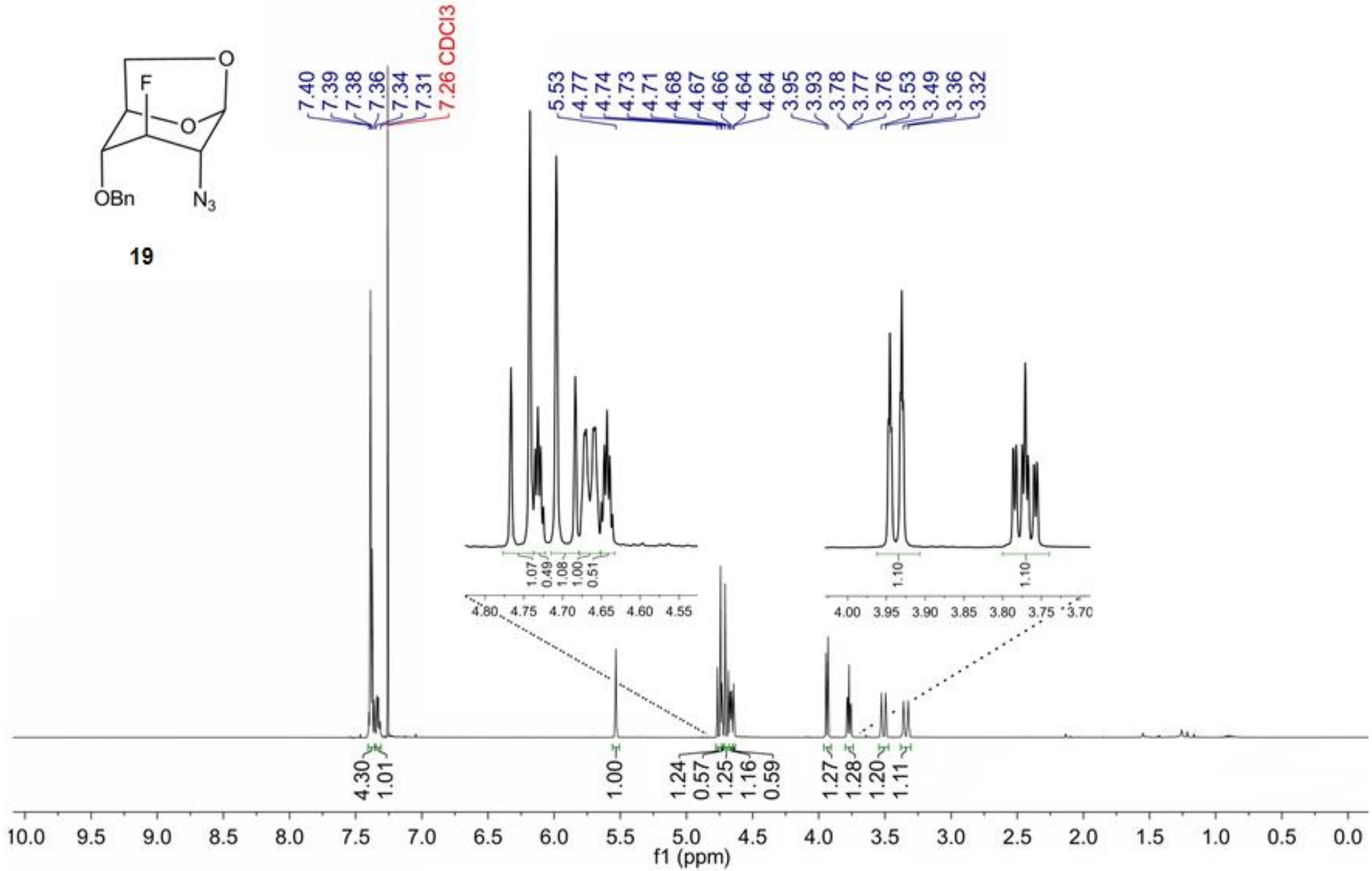

^{1}H NMR (500 MHz, $CDCl_3$) of **19**.

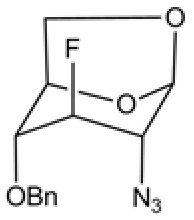

19

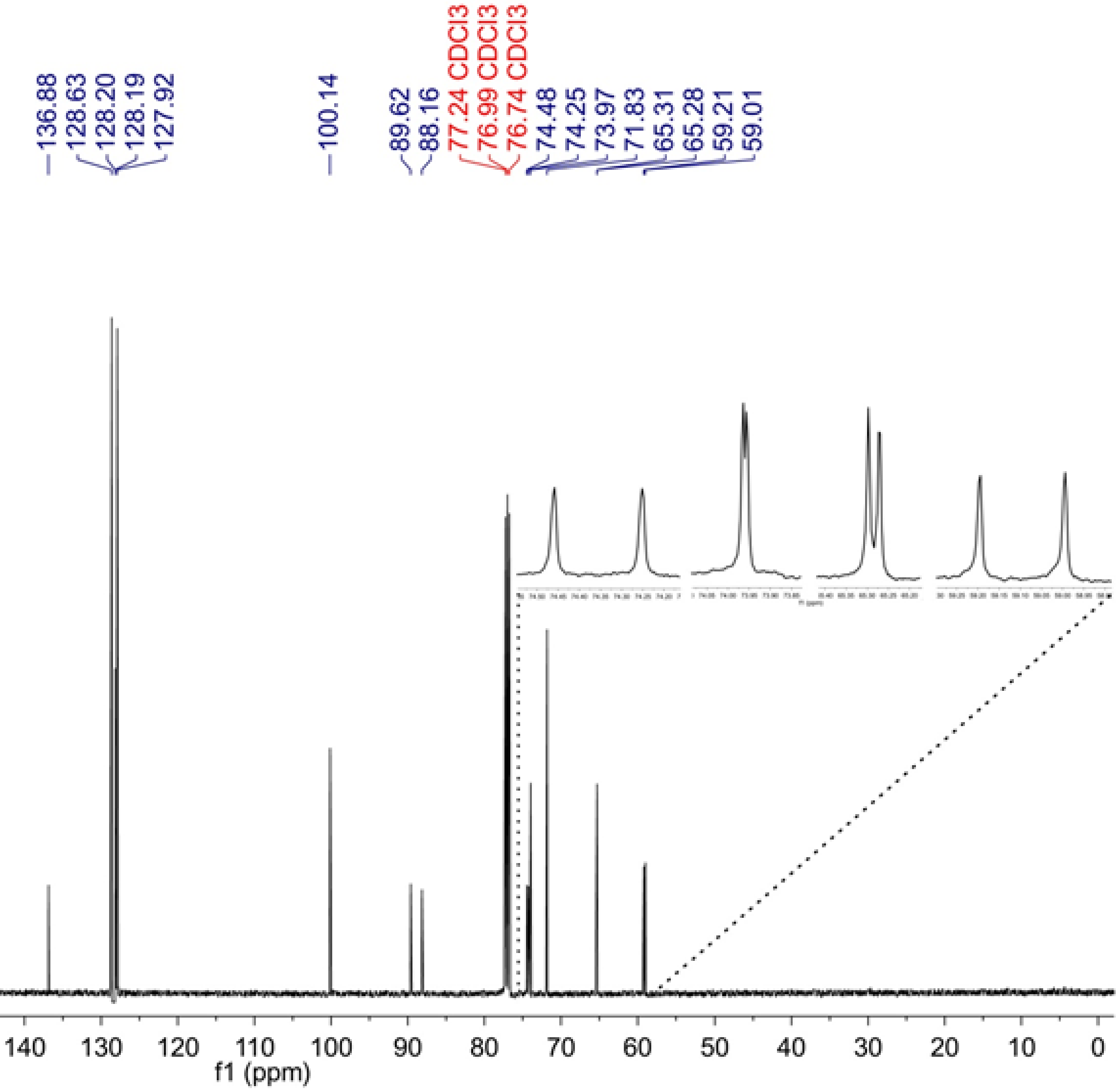

^{13}C {^{1}H} NMR (125 MHz, $CDCl_3$) of **19**

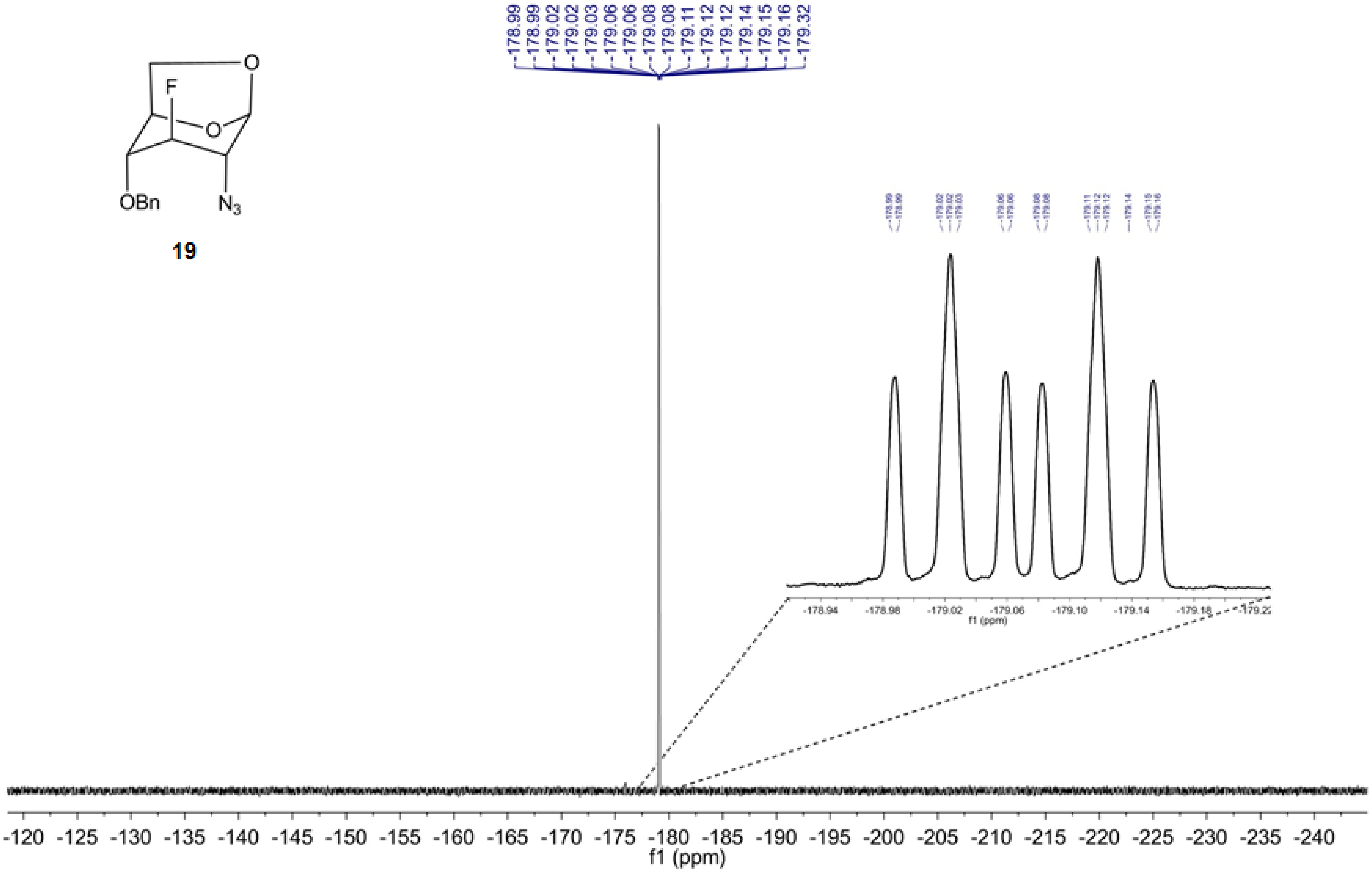

^{19}F NMR (470 MHz, $CDCl_3$) of **19**

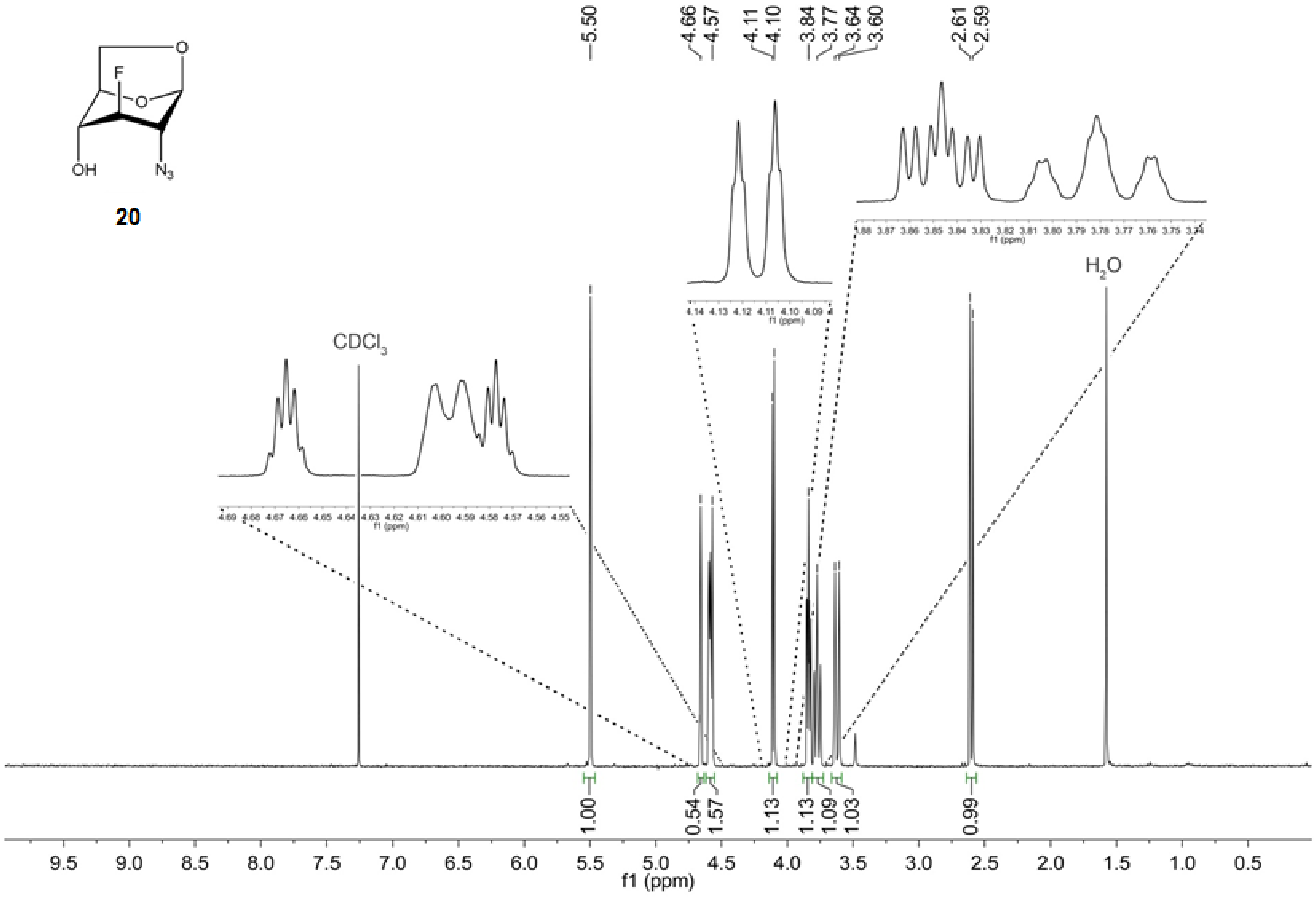

^1H NMR (300 MHz, $CDCl_3$) of **20**

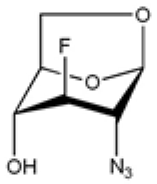

20

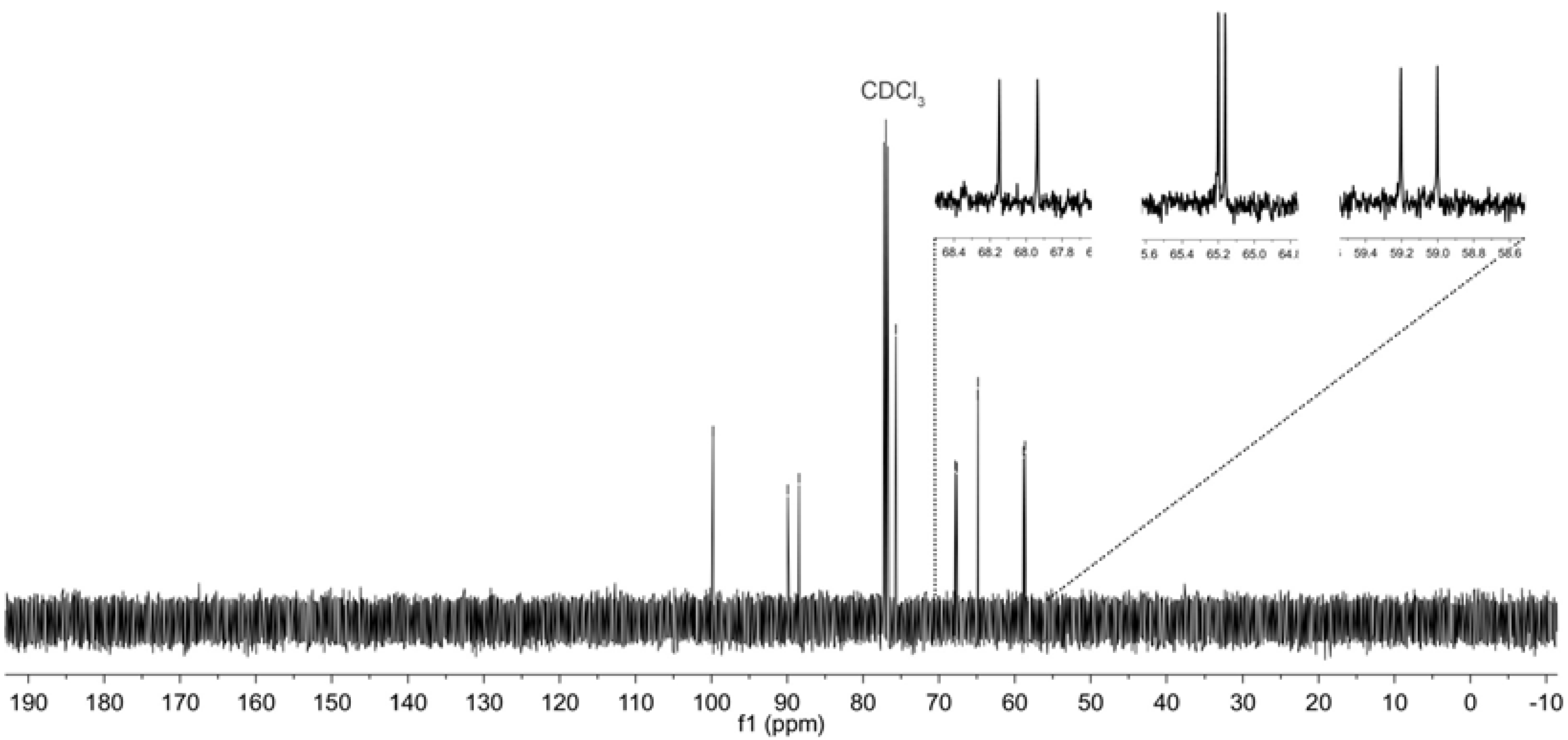

^{13}C {^{1}H} NMR (75 MHz, $CDCl_3$) of **20**

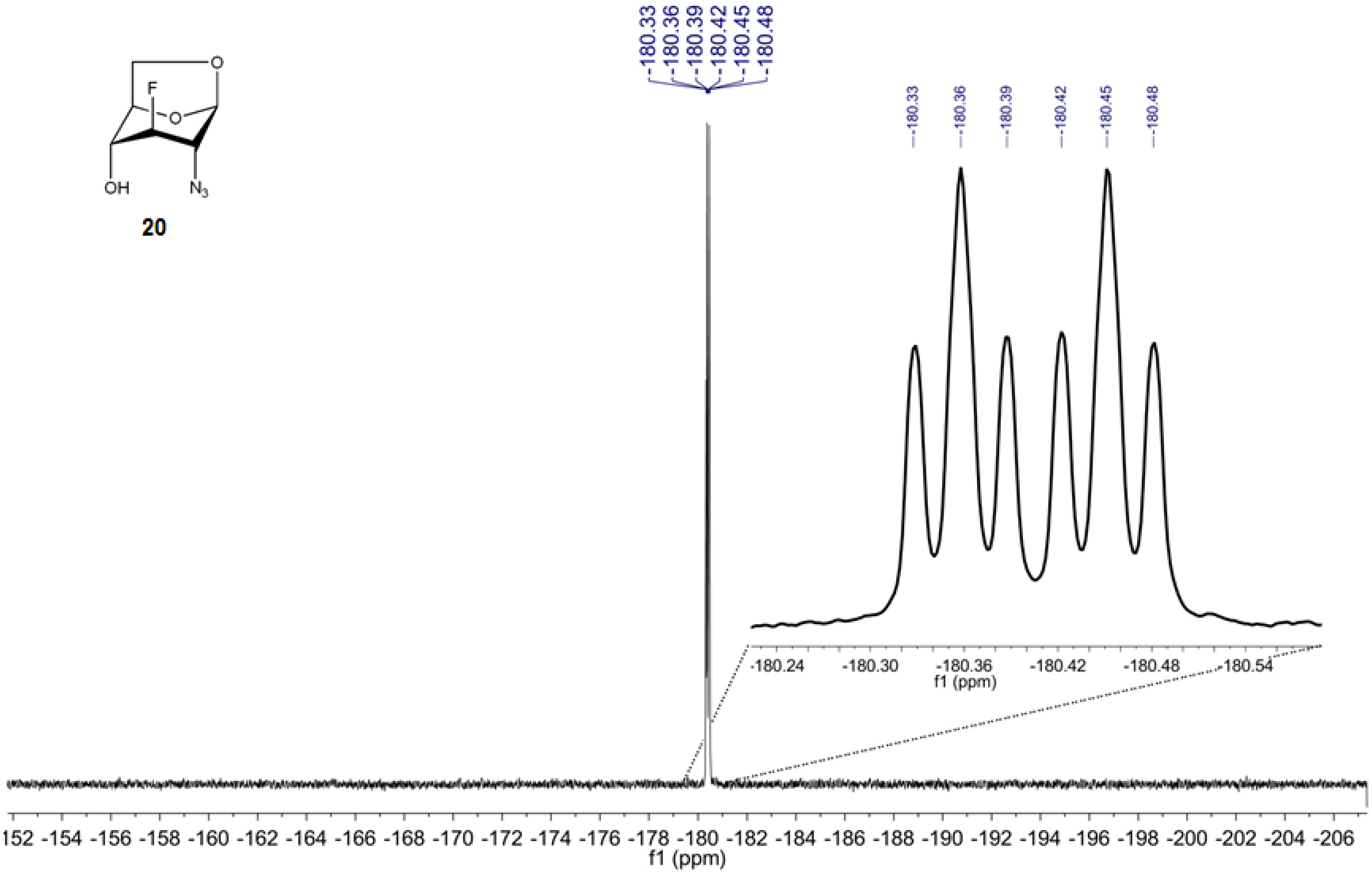

^{19}F NMR (282 MHz, $CDCl_3$) of **20**

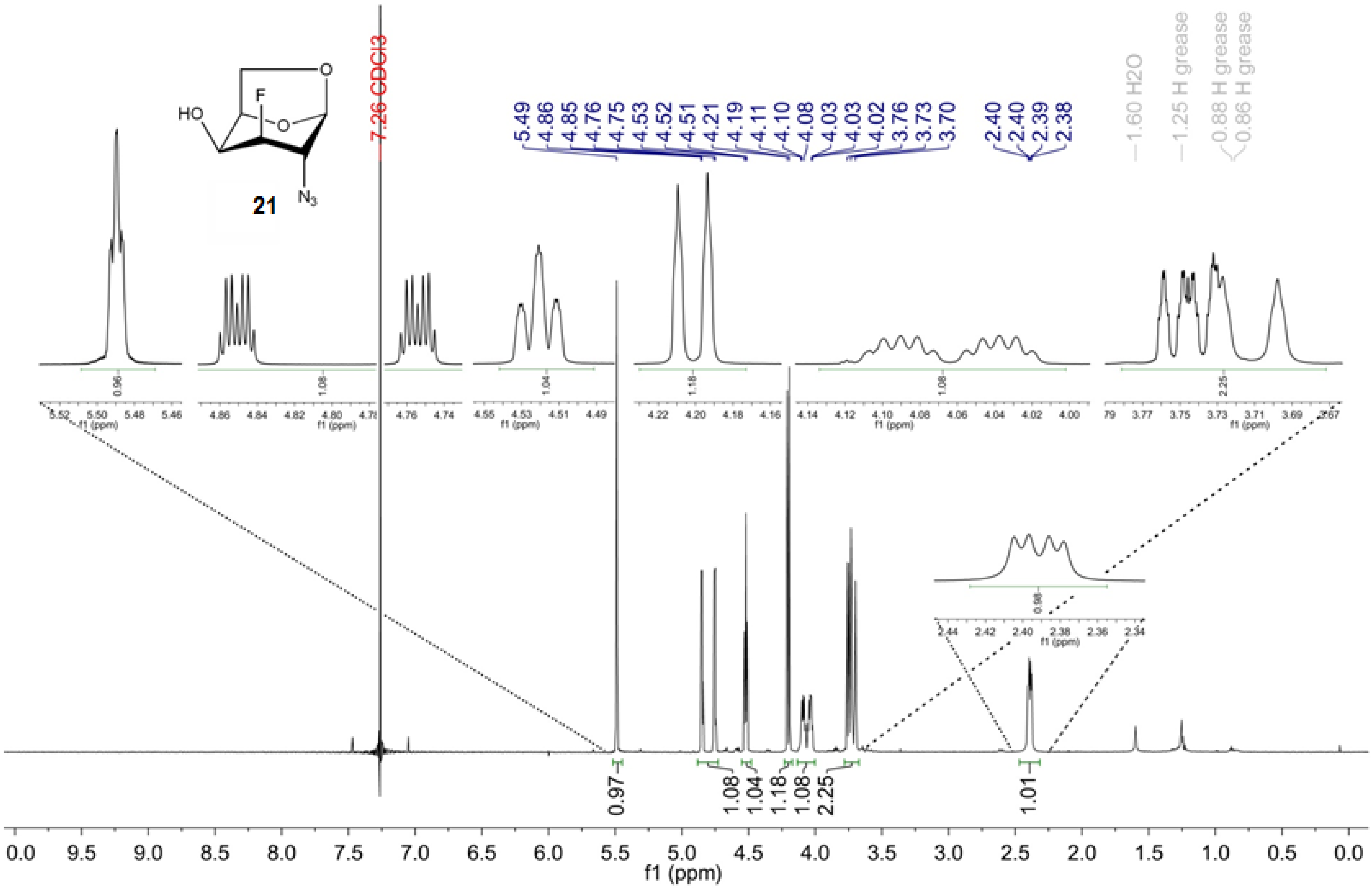

^{1}H NMR (300 MHz, $CDCl_3$) of **21**.

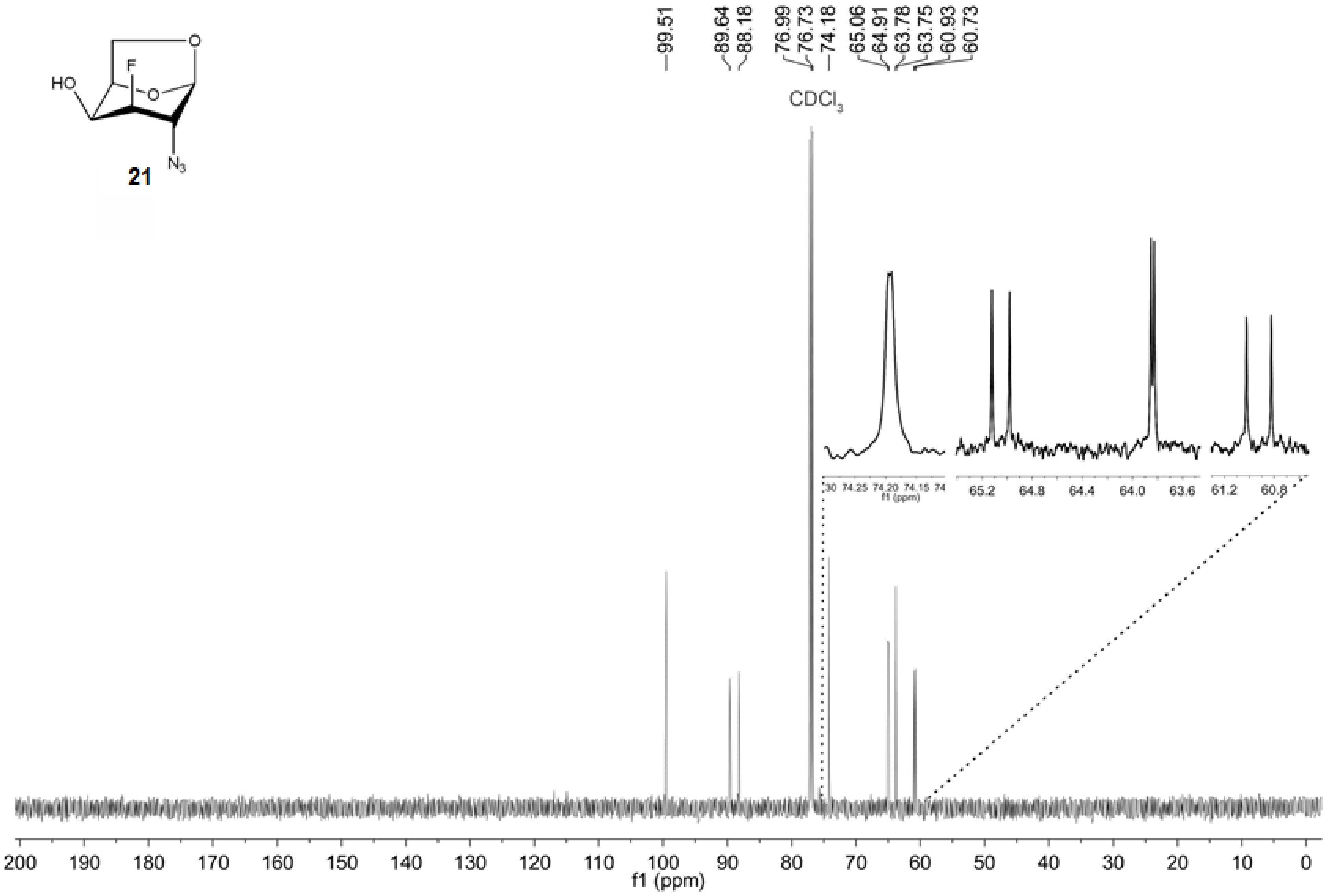

^{13}C {^{1}H} NMR (75 MHz, $CDCl_3$) of **21**

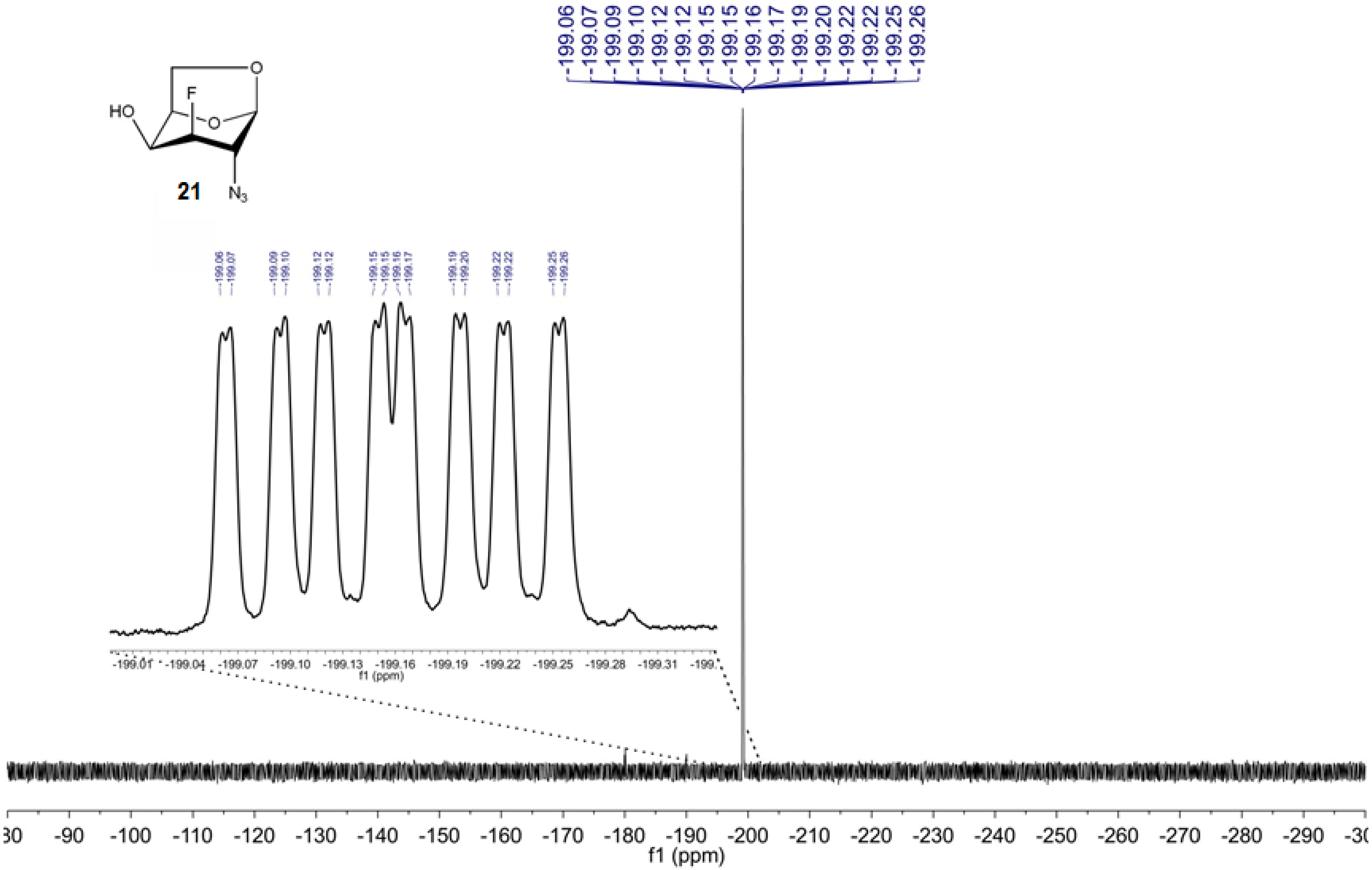

^{19}F NMR (282 MHz, $CDCl_3$) of **21**

22

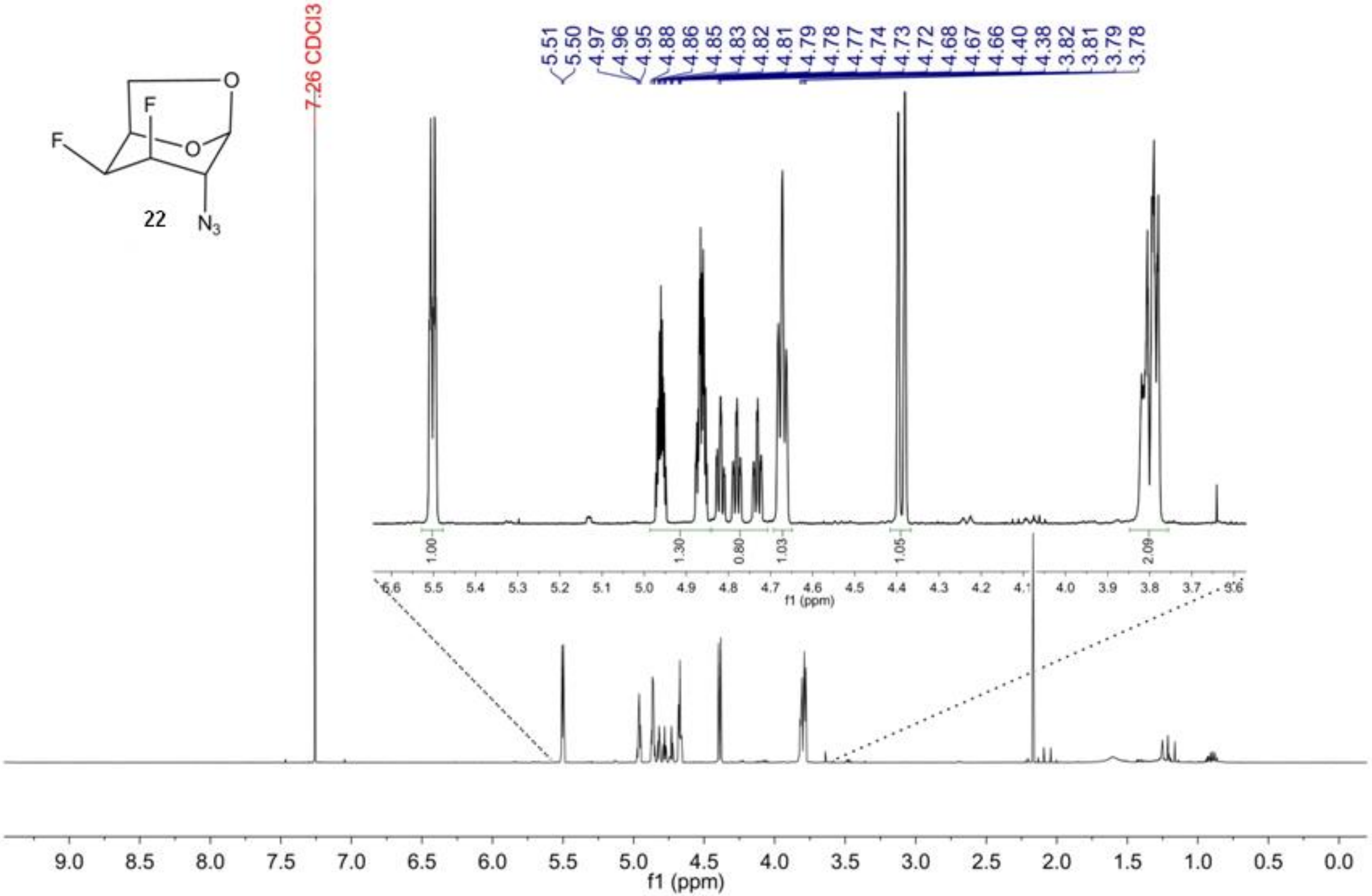

^{1}H NMR (500 MHz, $CDCl_3$) of **22**

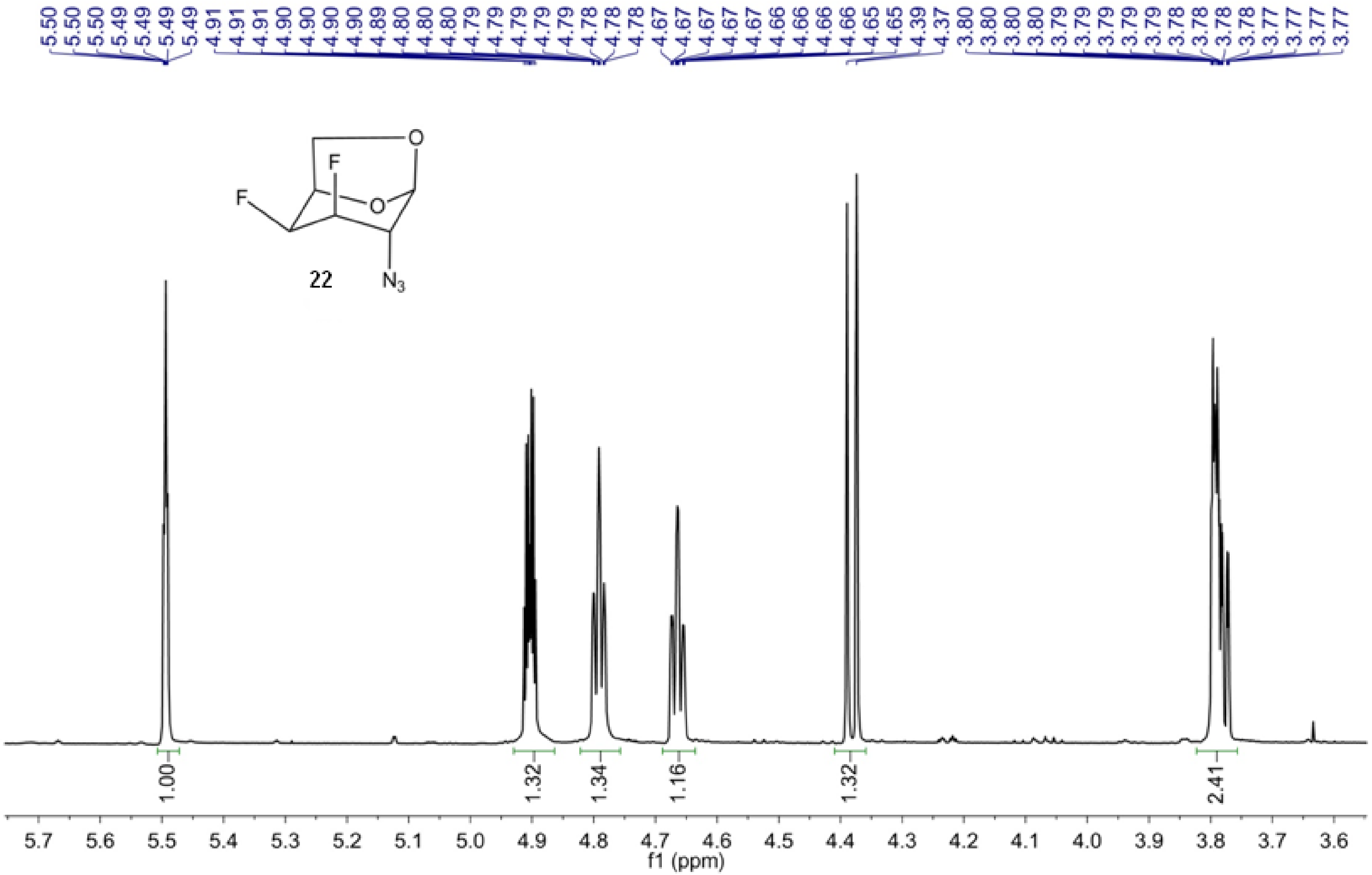

$^{1}H\ \{^{19}F\}$ NMR (500 MHz, $CDCl_3$) of **22**

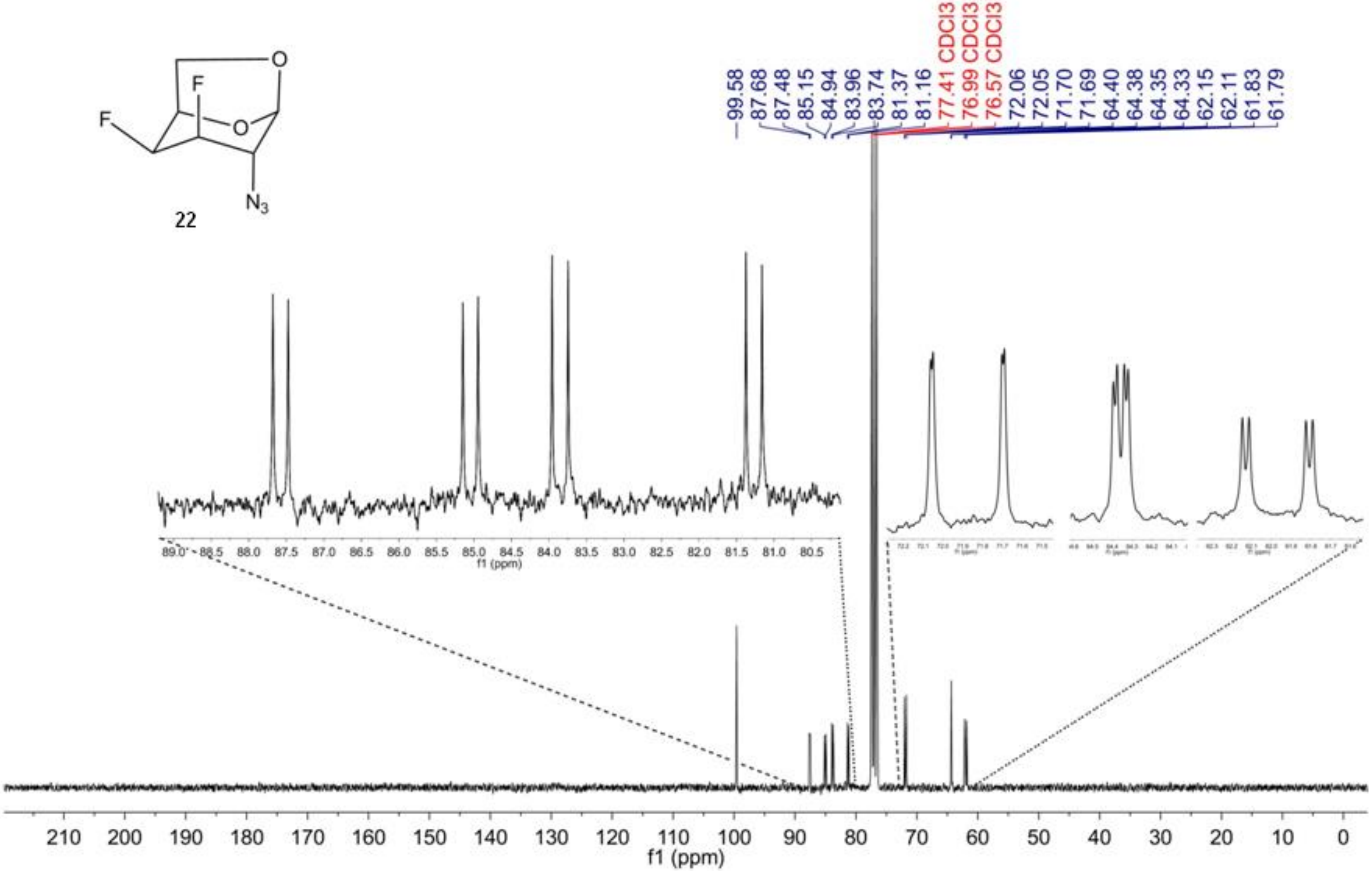

$^{13}C\ \{^{1}H\}$ NMR (75 MHz, $CDCl_3$) of **22**

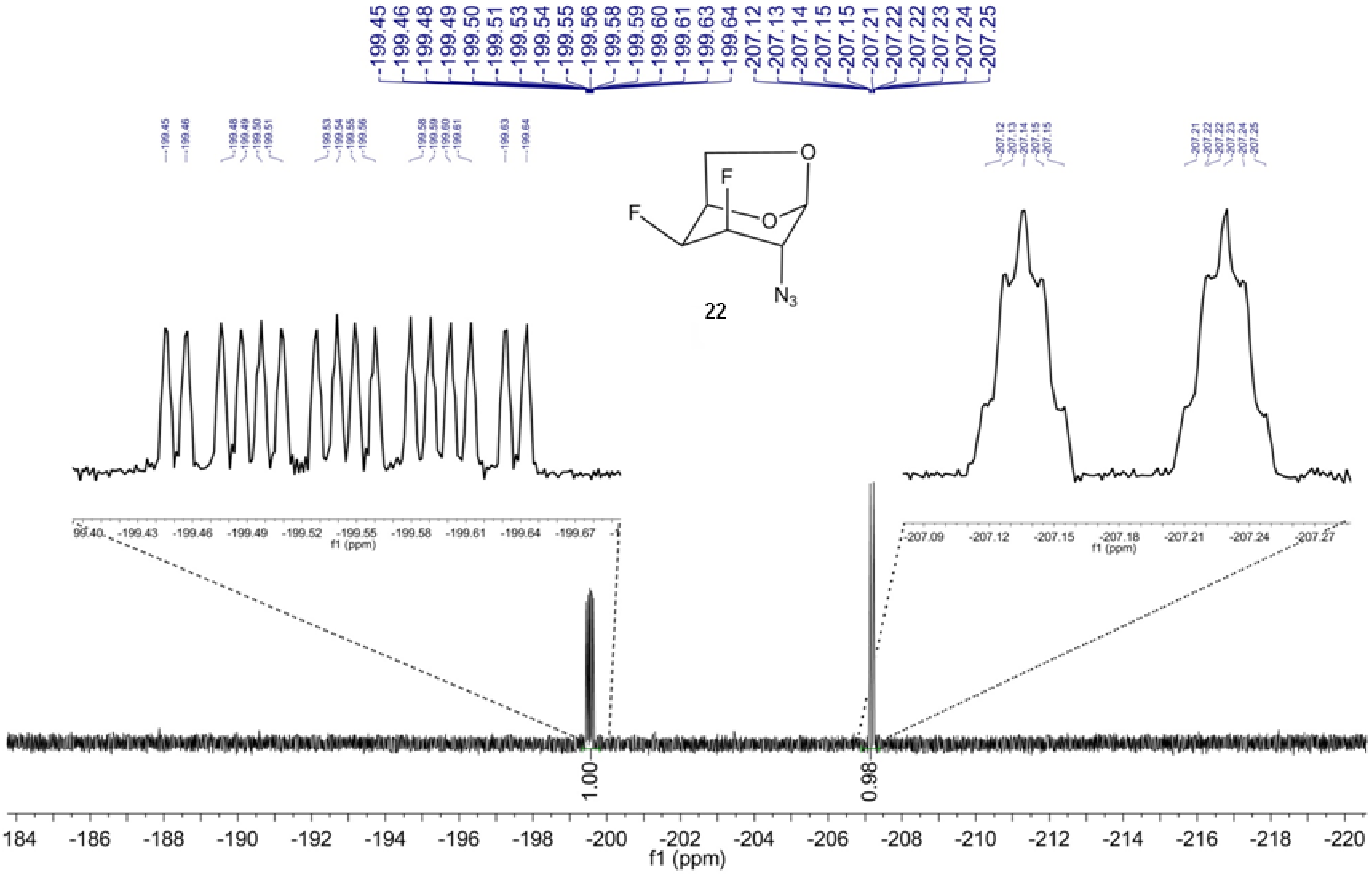

^{19}F NMR (470 MHz, $CDCl_3$) of **22**

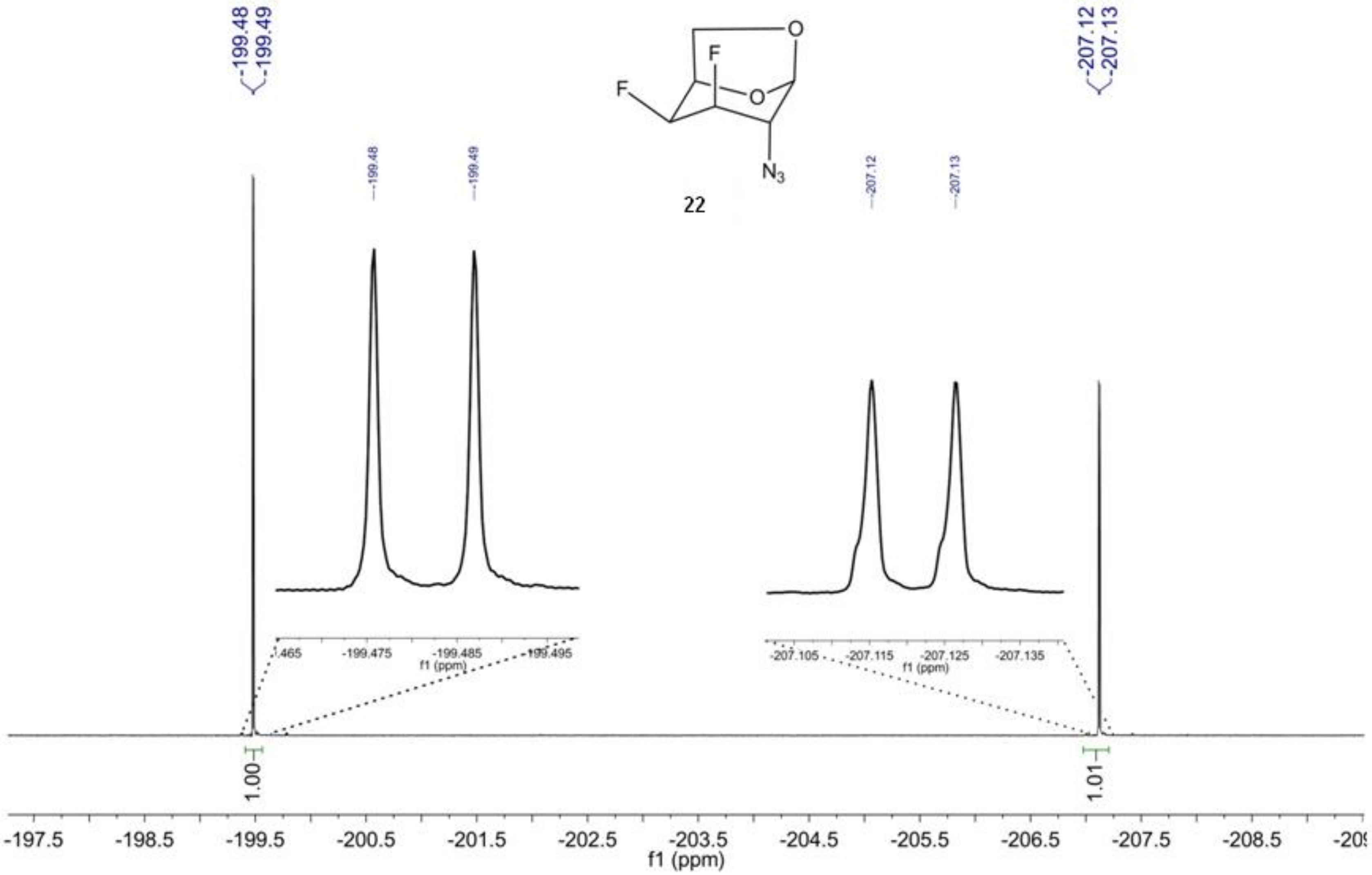

^{19}F {^{1}H} NMR (470 MHz, $CDCl_3$) of **22**

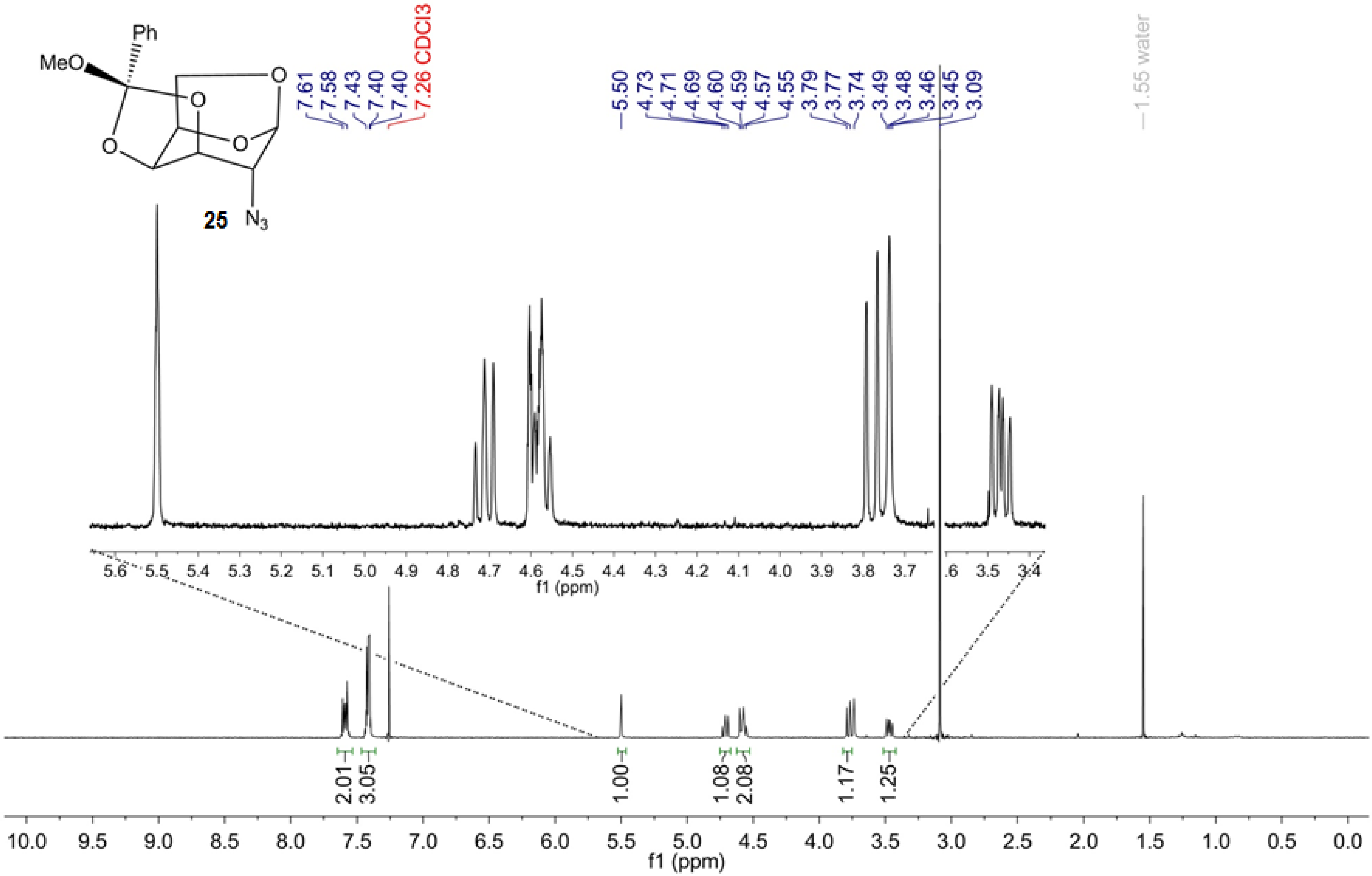

^{1}H NMR (500 MHz, $CDCl_3$) of **25**

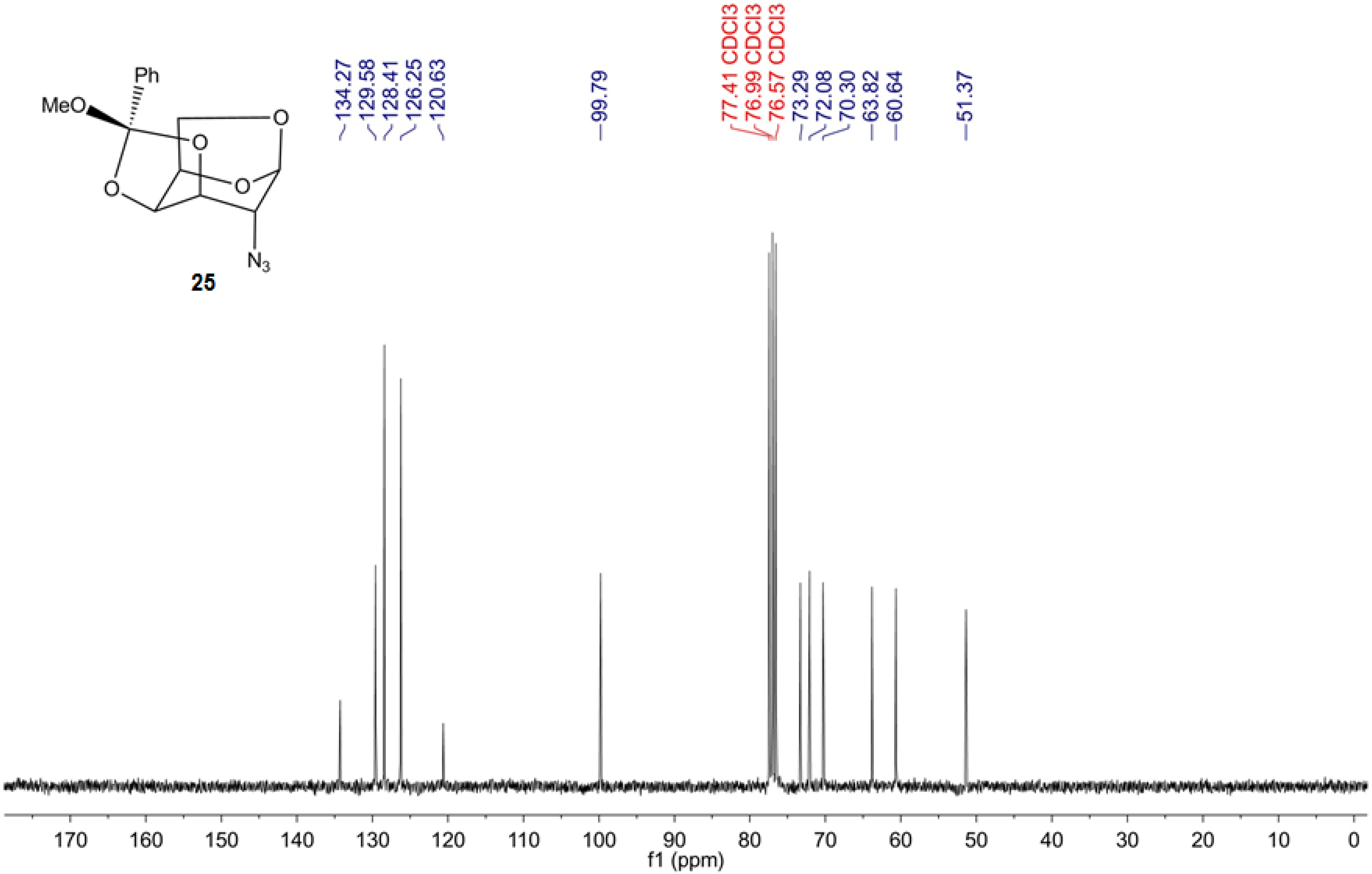

^{13}C {^{1}H} NMR (125 MHz, $CDCl_3$) of **25**

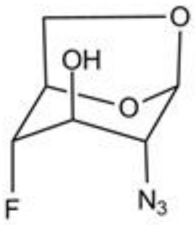

26

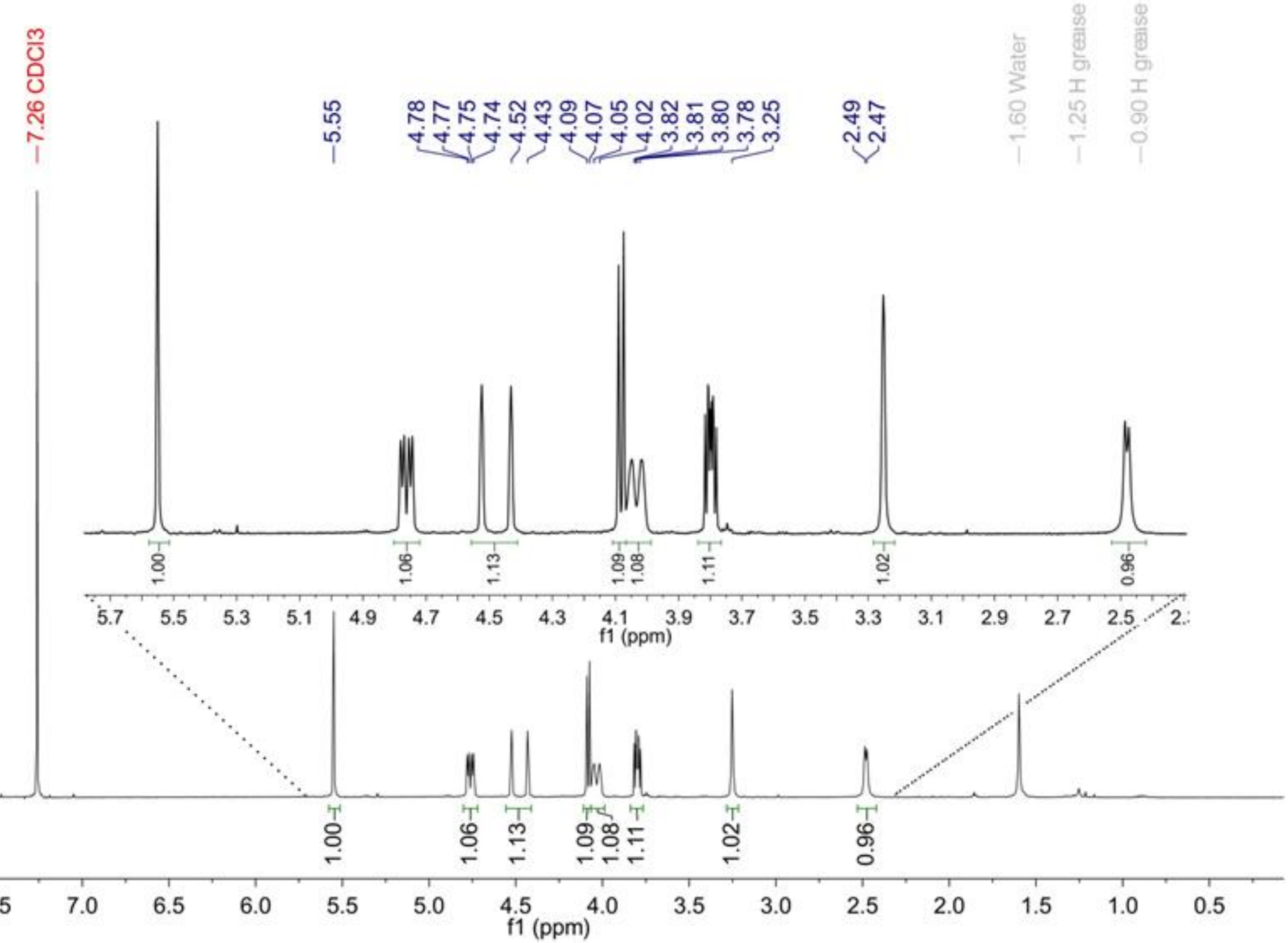

^{1}H NMR (300 MHz, $CDCl_3$) of **26**

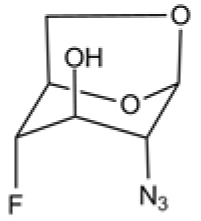

26

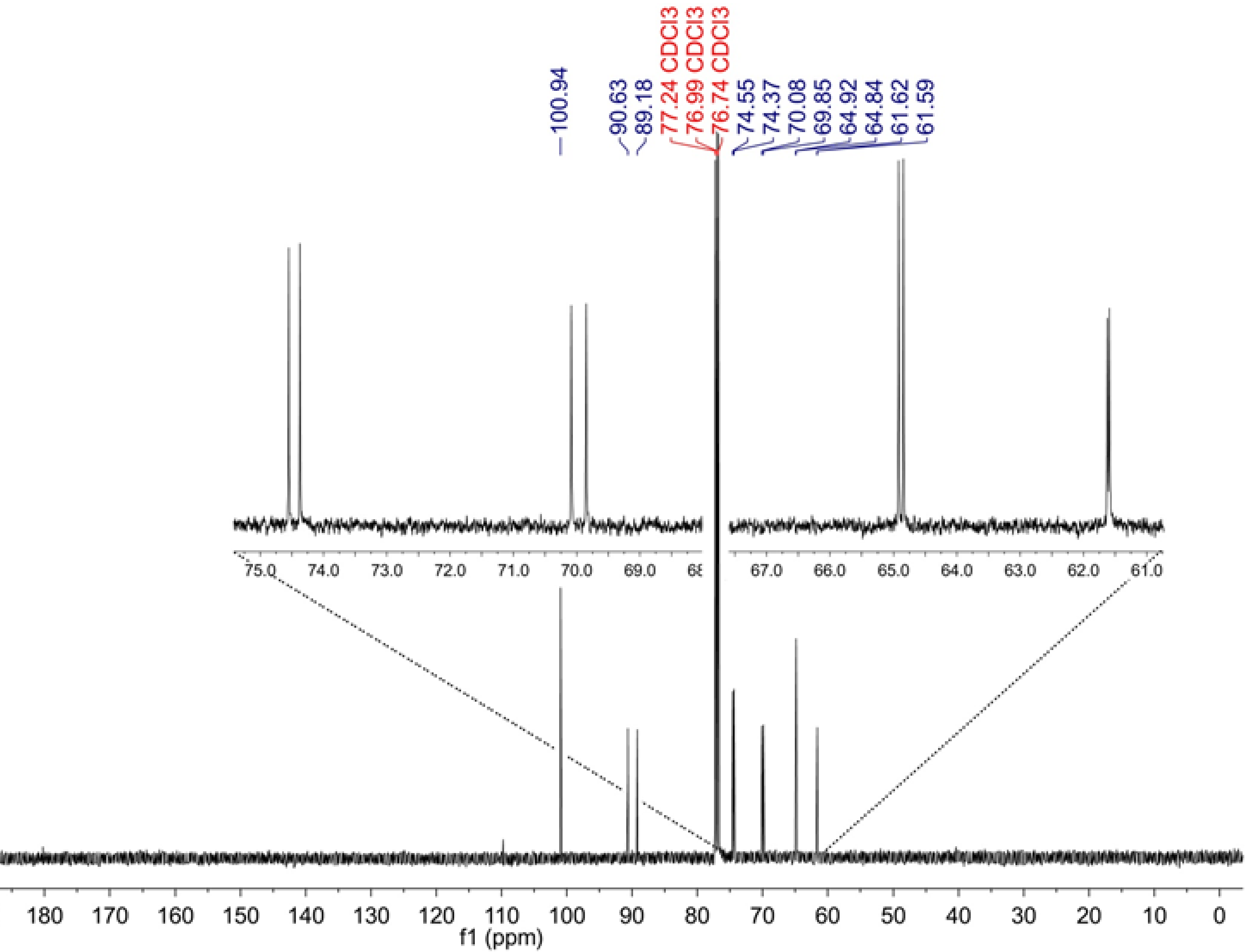

^{13}C {^{1}H} NMR (75 MHz, $CDCl_3$) of **26**

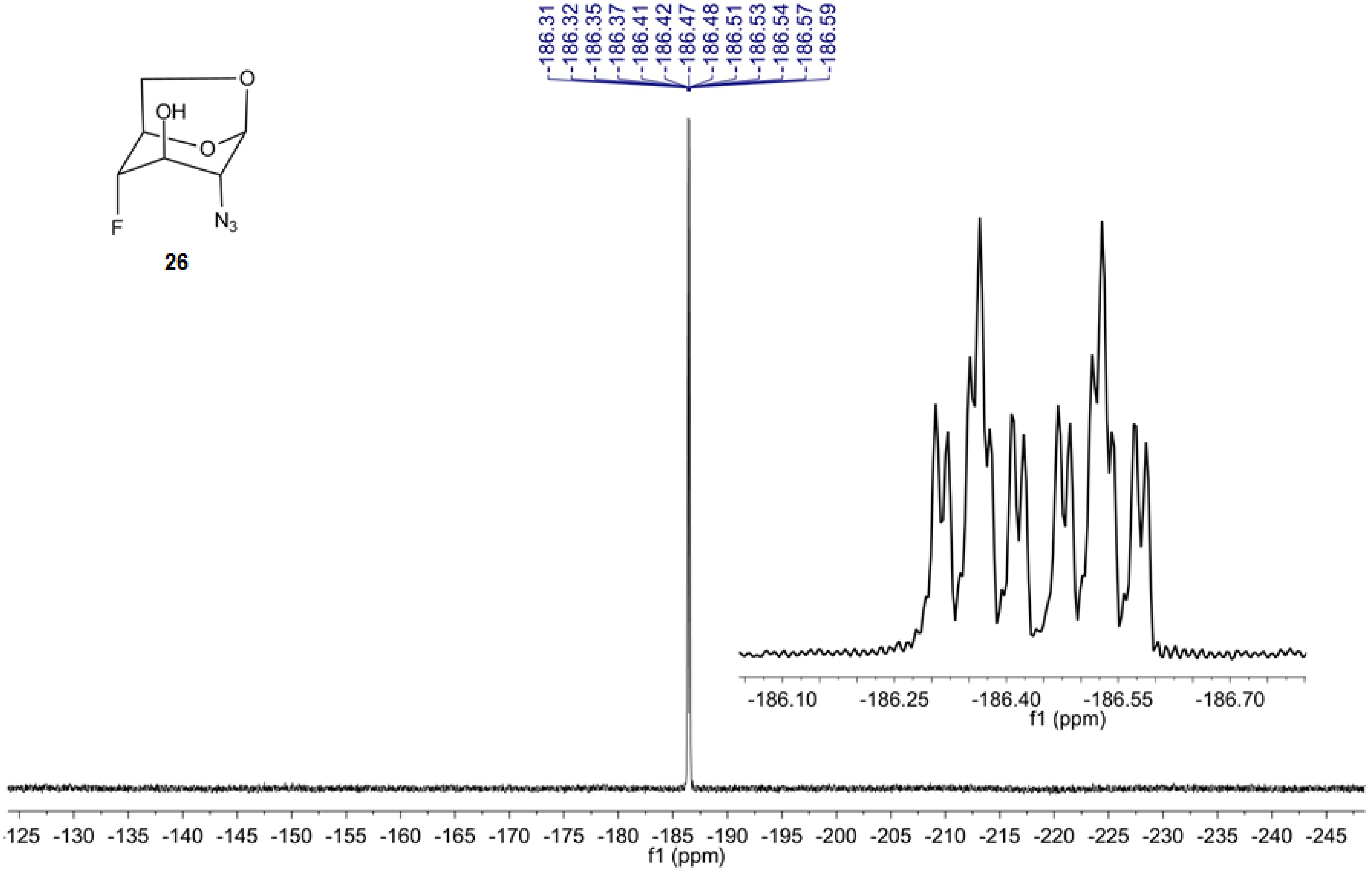

26

^{19}F NMR (282 MHz, $CDCl_3$) of **26**

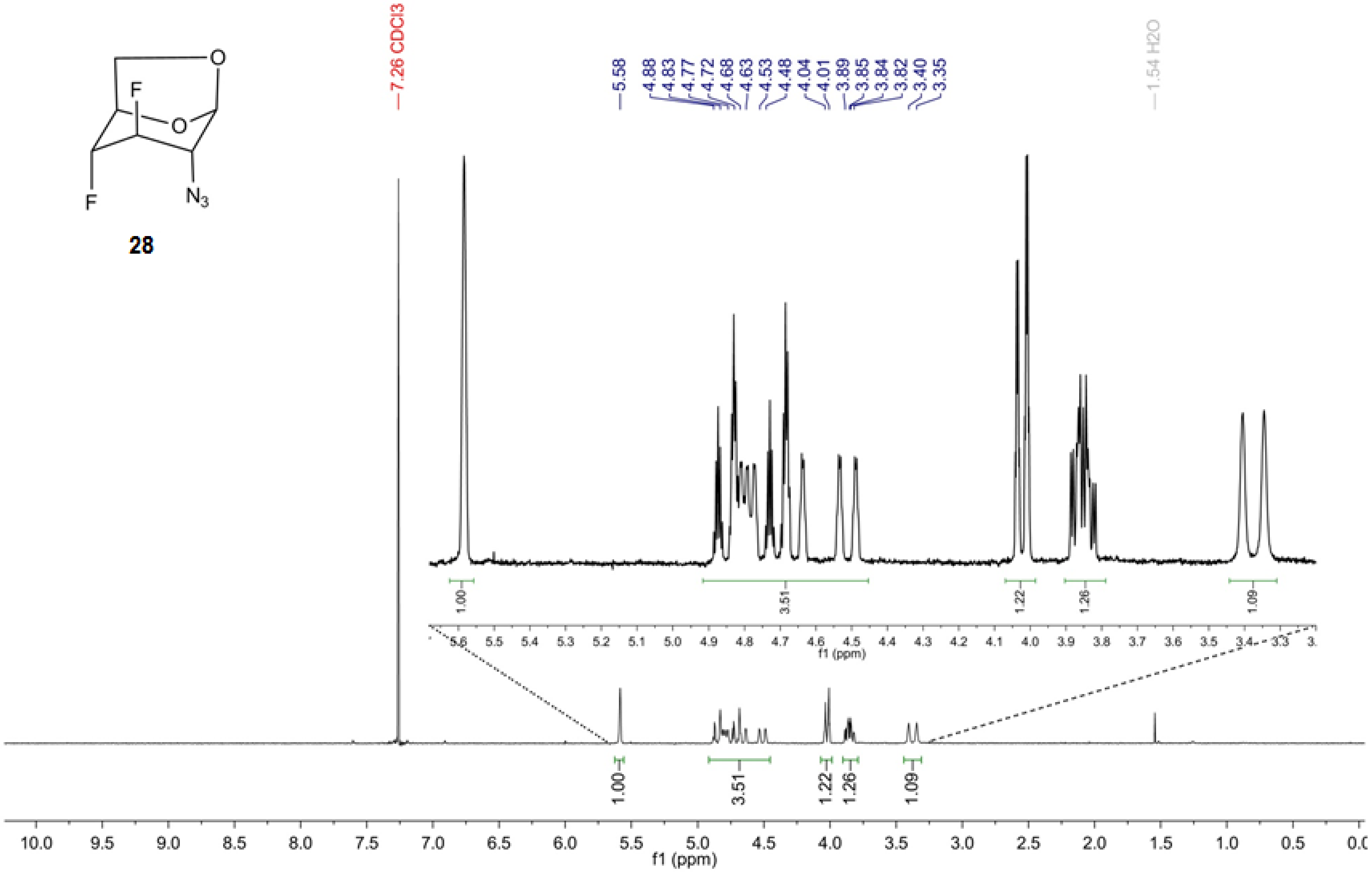

28

^1H NMR (300 MHz, $CDCl_3$) of **28**

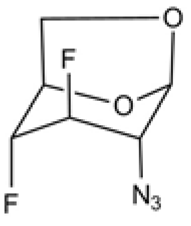

28

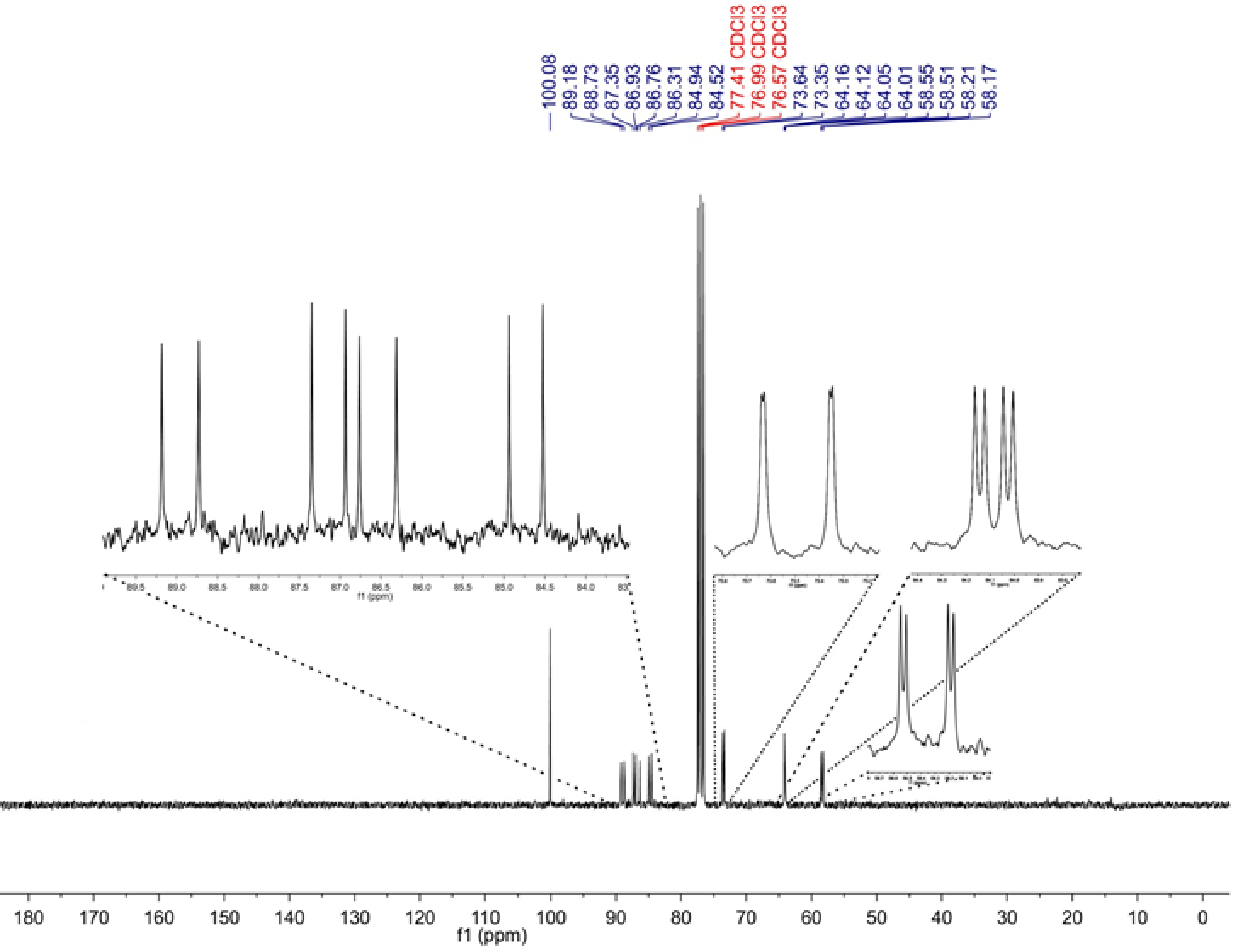

^{13}C {^{1}H} NMR (75 MHz, $CDCl_3$) of **28**

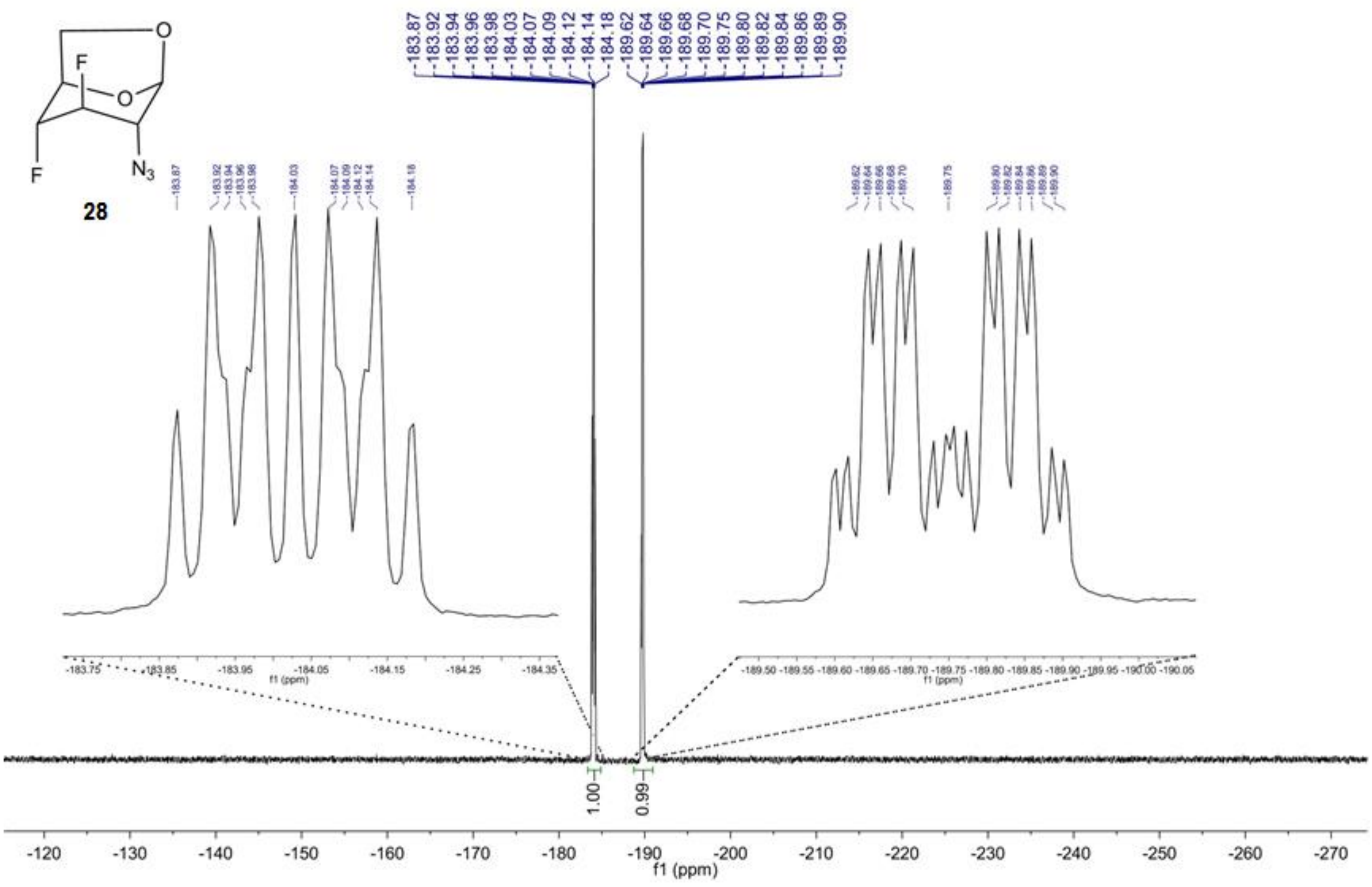

^{19}F NMR (282 MHz, $CDCl_3$) of **28**

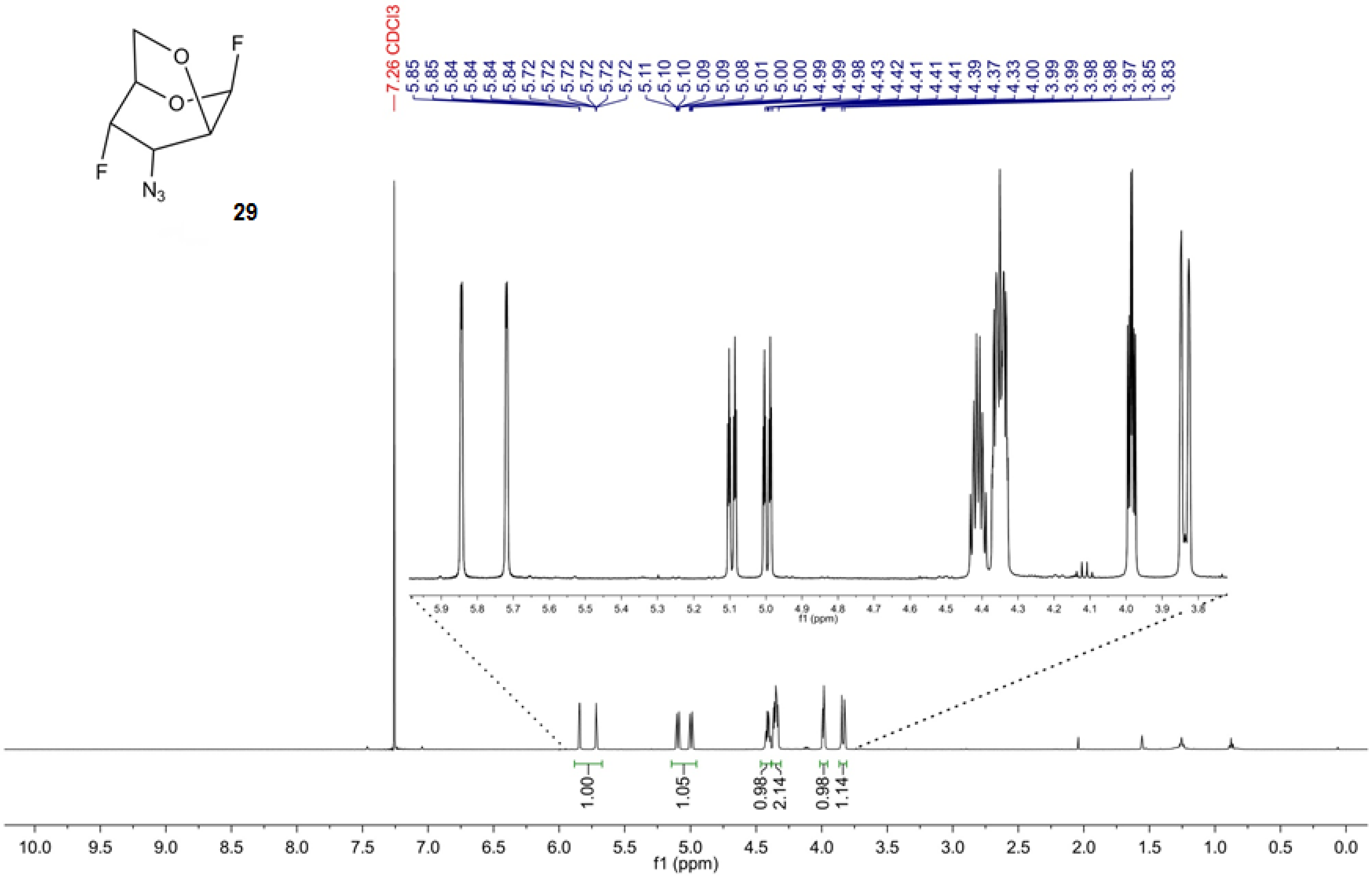

^{1}H NMR (500 MHz, $CDCl_3$) of **29**

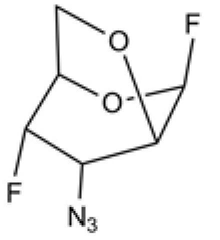

29

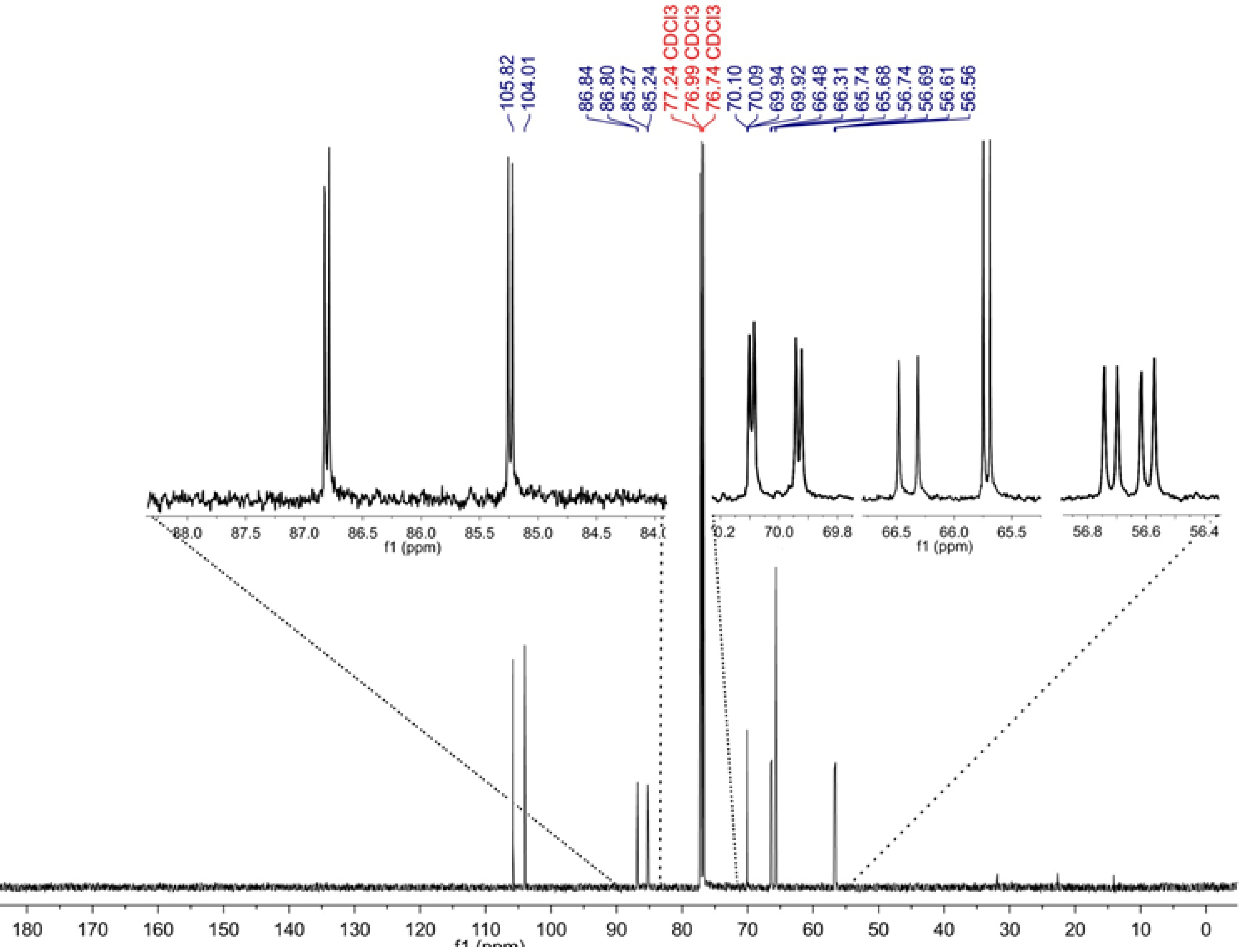

^{13}C {^{1}H} NMR (75 MHz, $CDCl_3$) of **29**

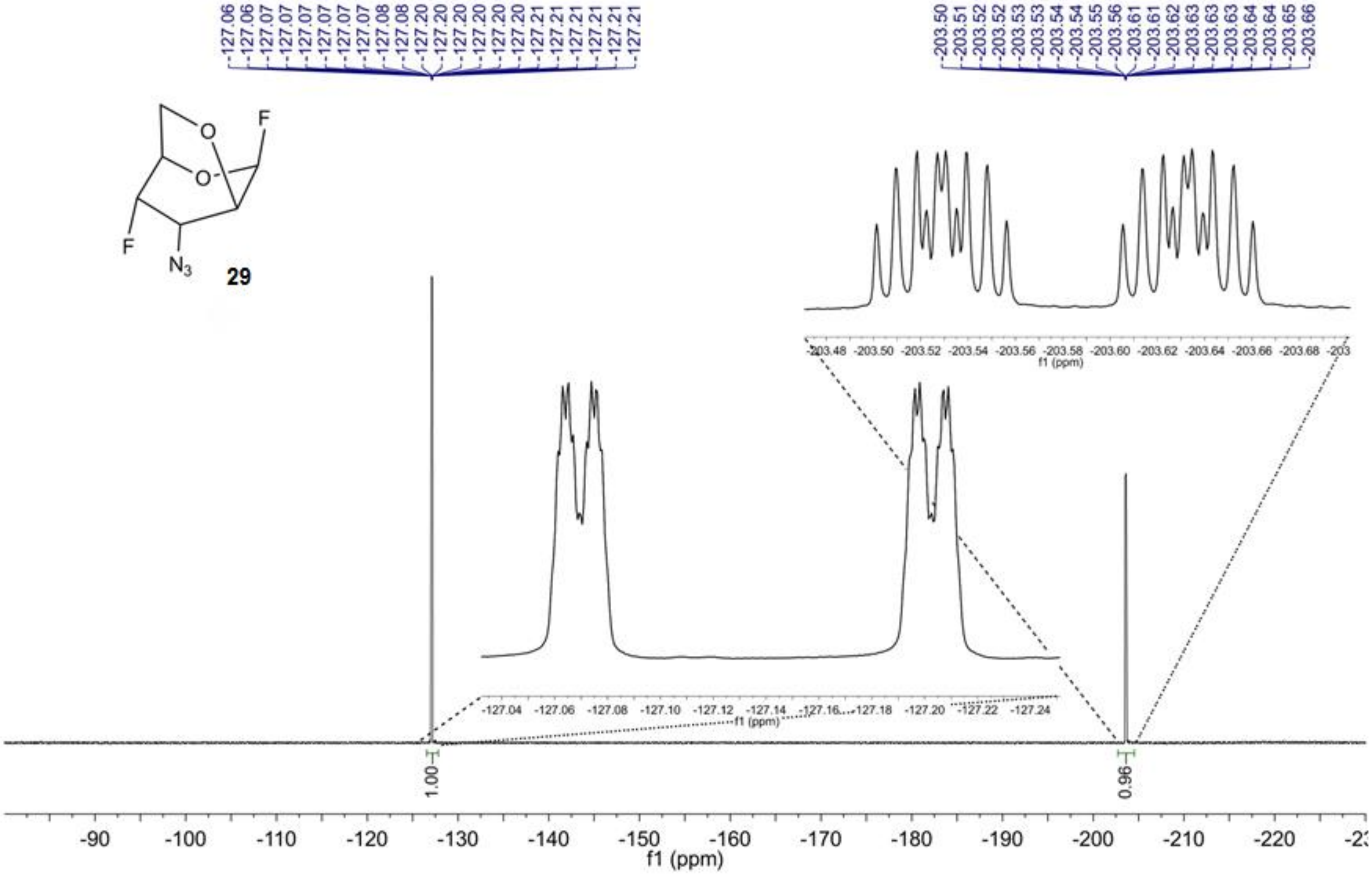

^{19}F NMR (470 MHz, $CDCl_3$) of **29**

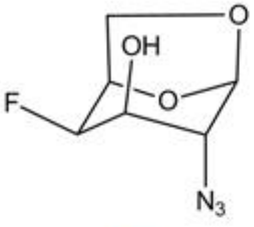

31

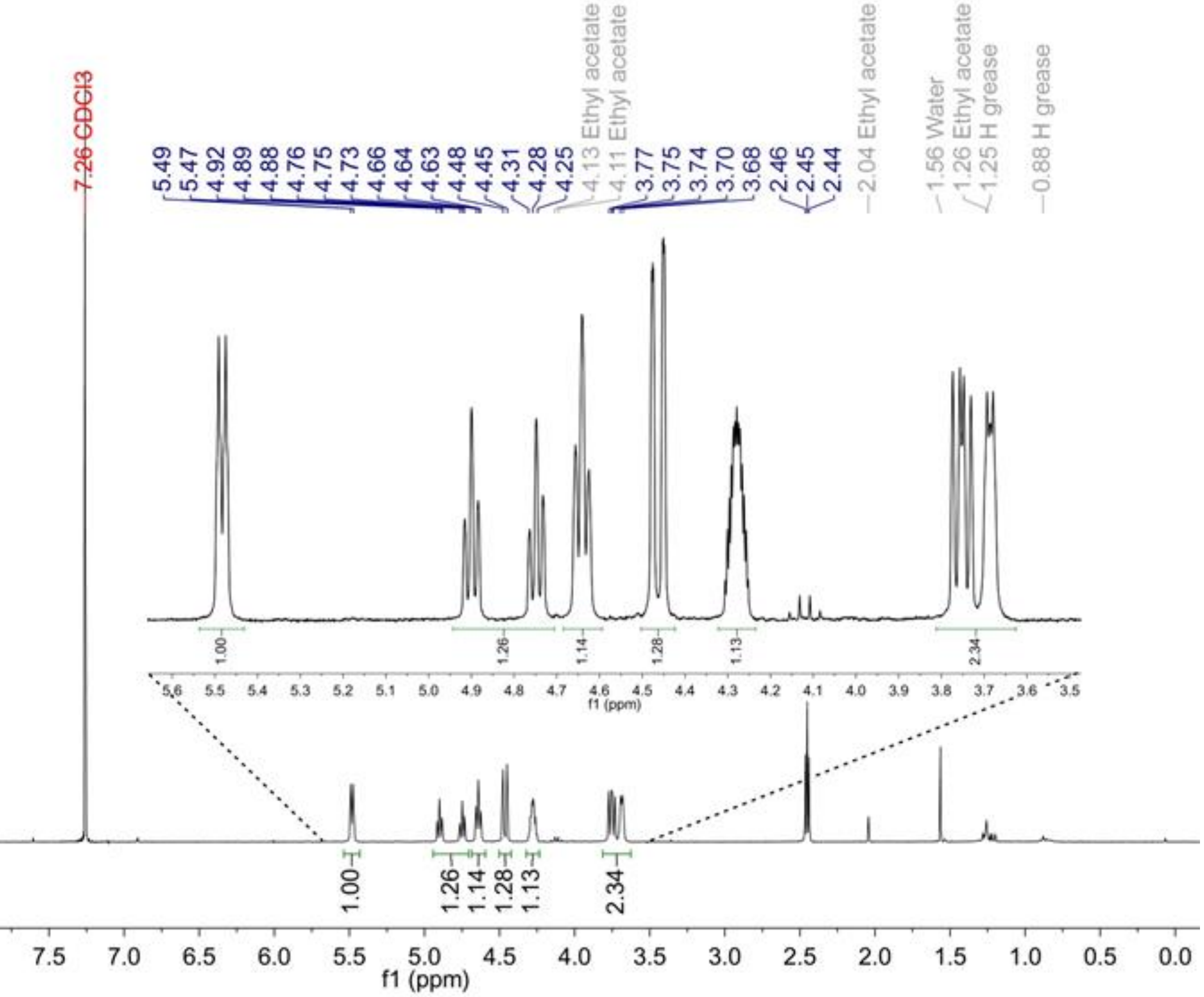

^{1}H NMR (300 MHz, $CDCl_3$) of **31**

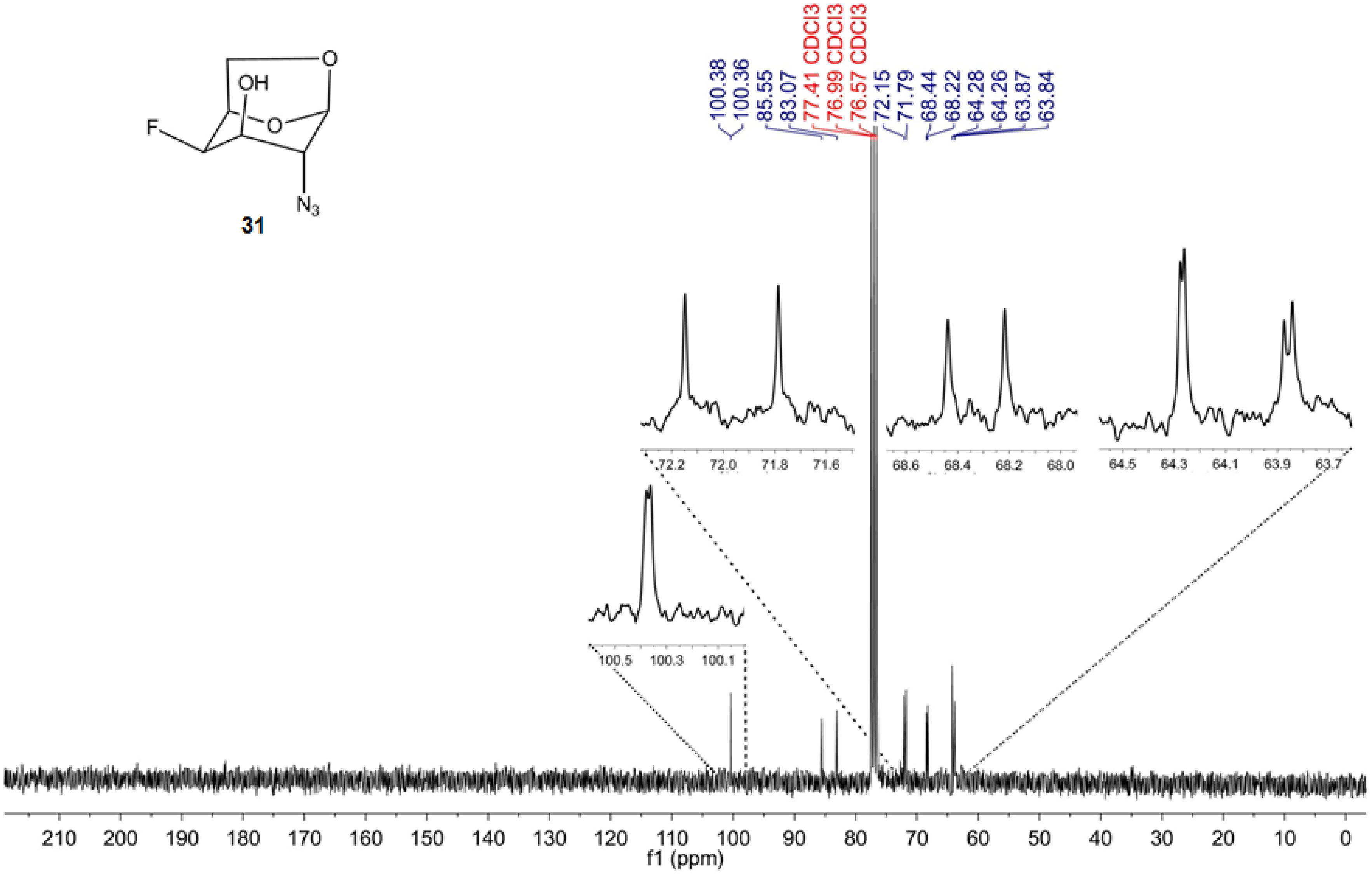

$^{13}C\ \{^{1}H\}$ NMR (75 MHz, $CDCl_3$) of **31**

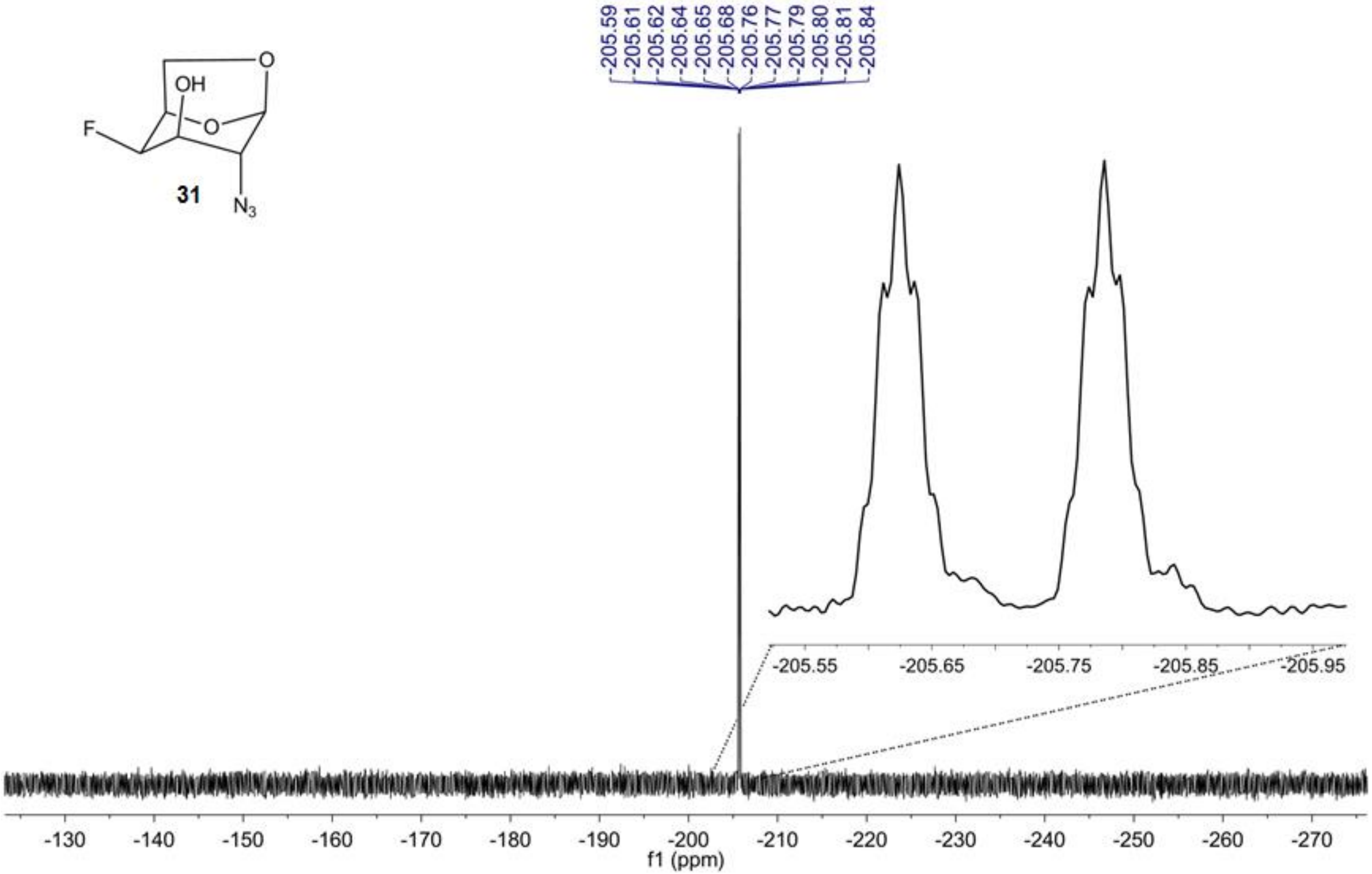

^{19}F NMR (282 MHz, $CDCl_3$) of **31**

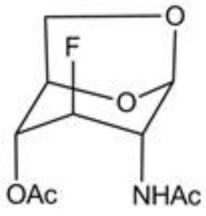

40

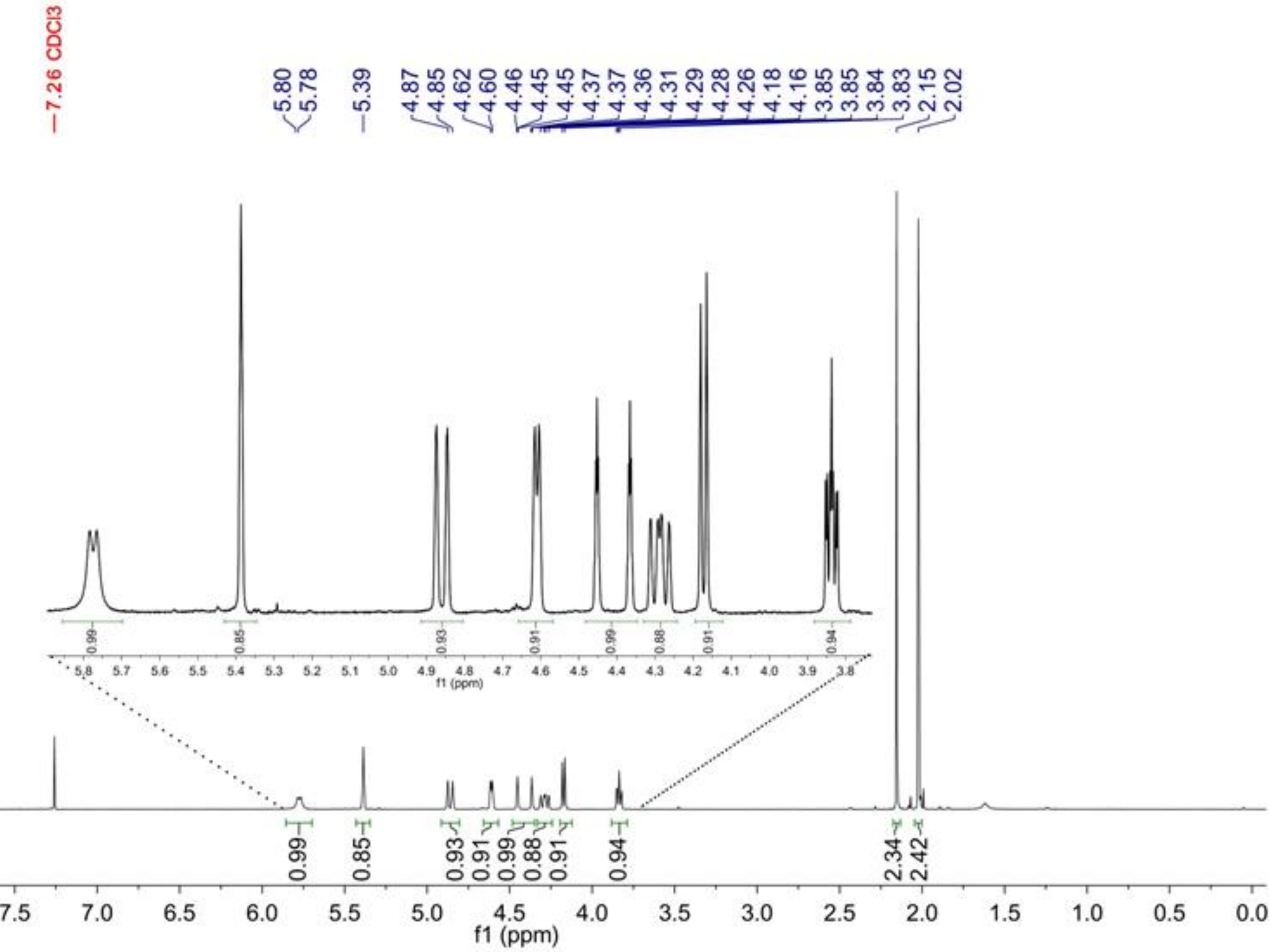

^{1}H NMR (500 MHz, $CDCl_3$) of **40**

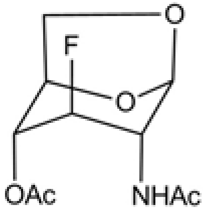

40

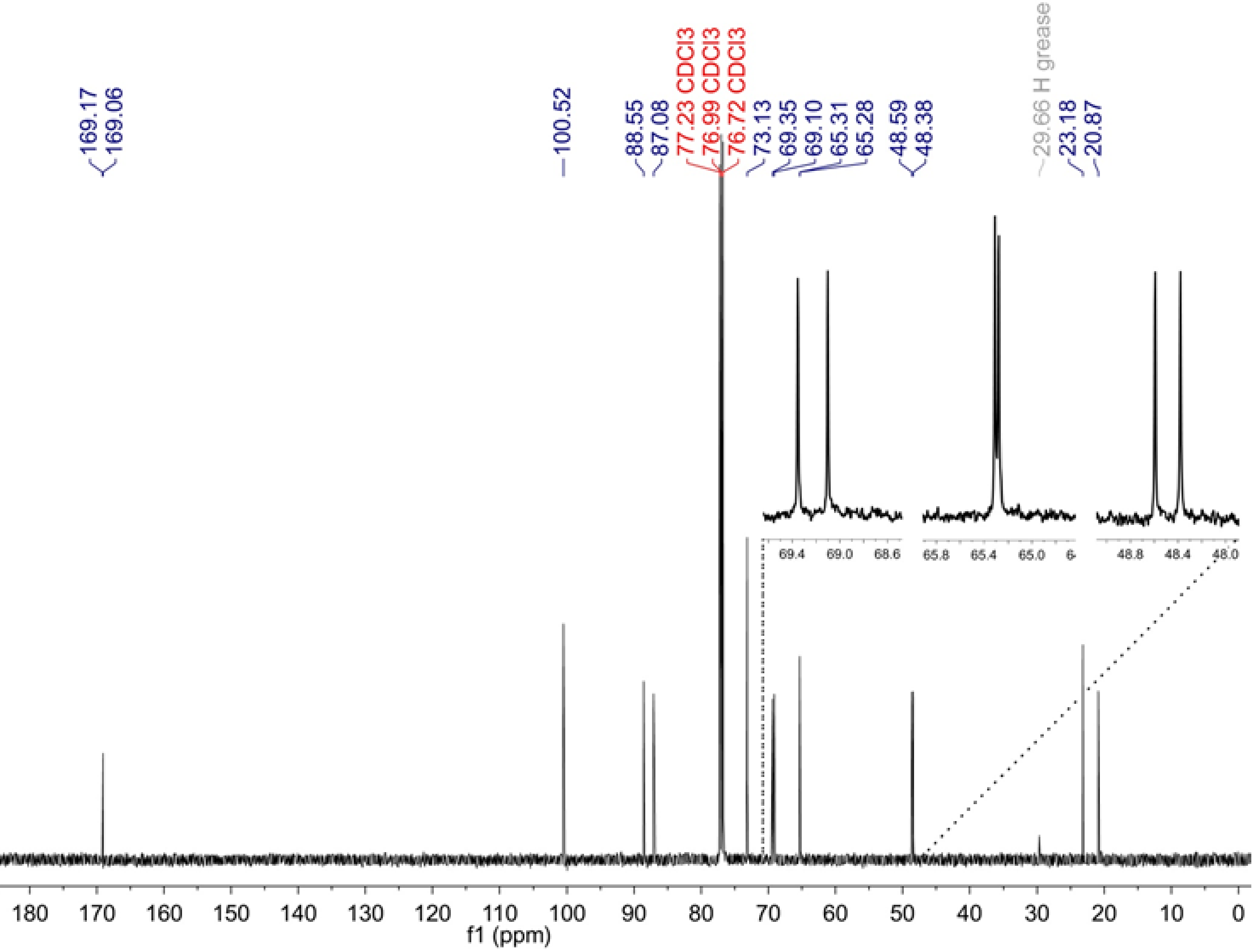

^{13}C {^{1}H} NMR (125 MHz, $CDCl_3$) of **40**

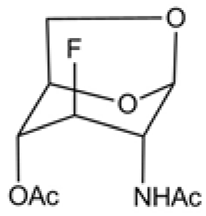

40

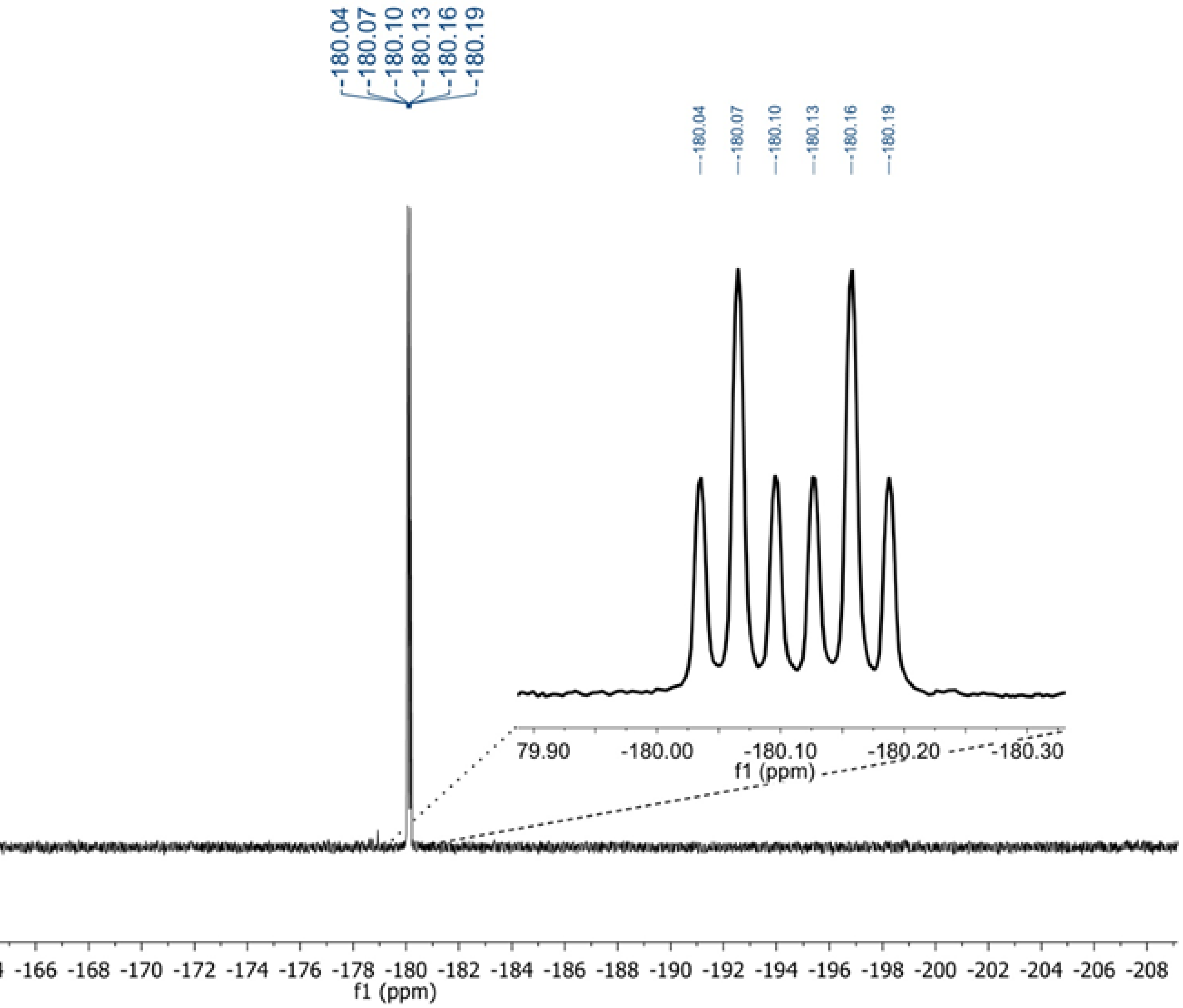

^{19}F NMR (470 MHz, $CDCl_3$) of **40**

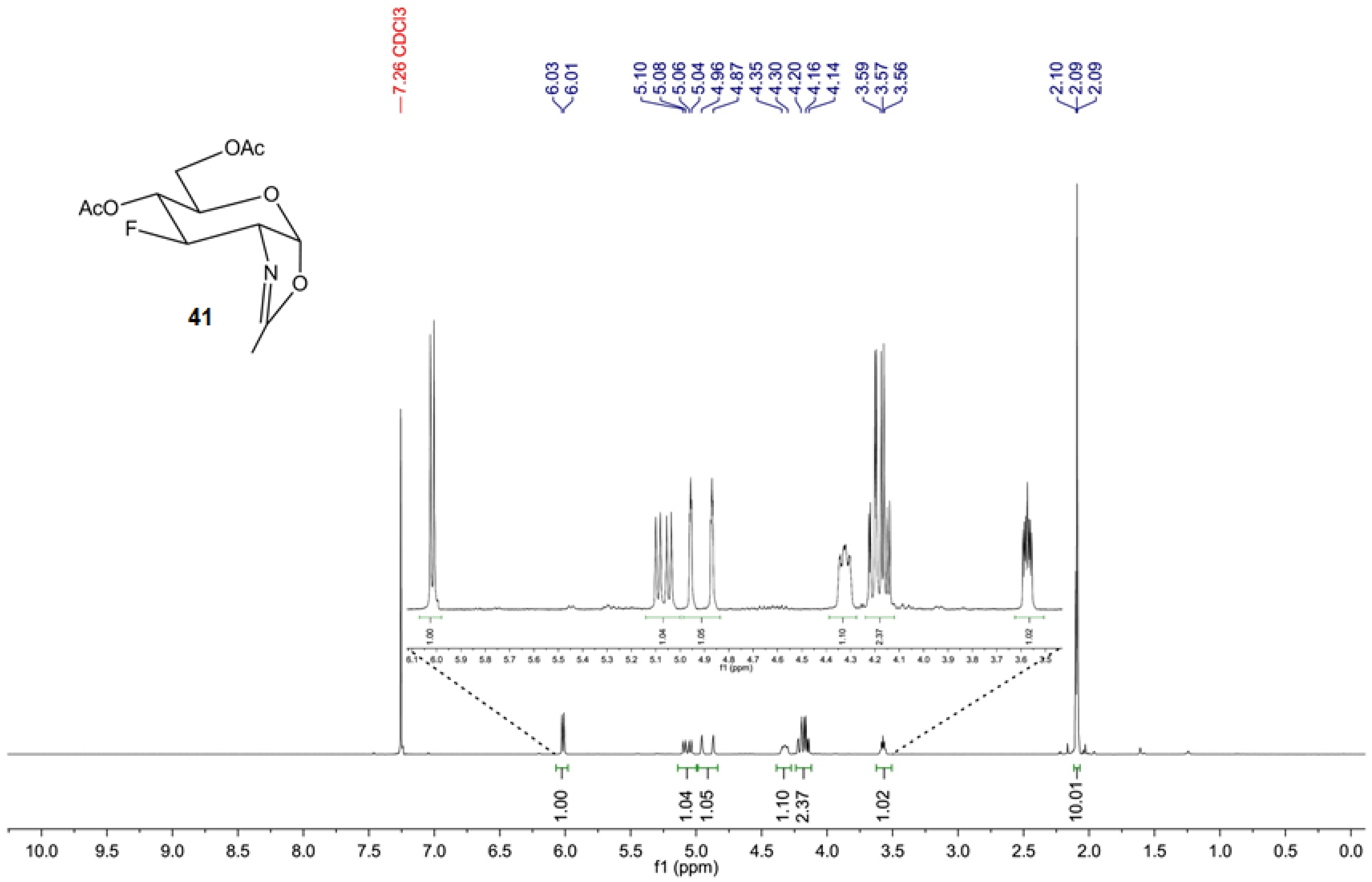

^{1}H NMR (500 MHz, $CDCl_3$) of **41**

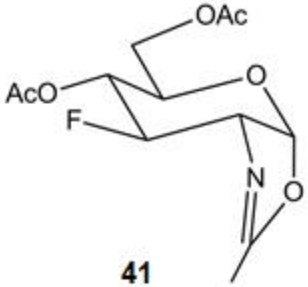

41

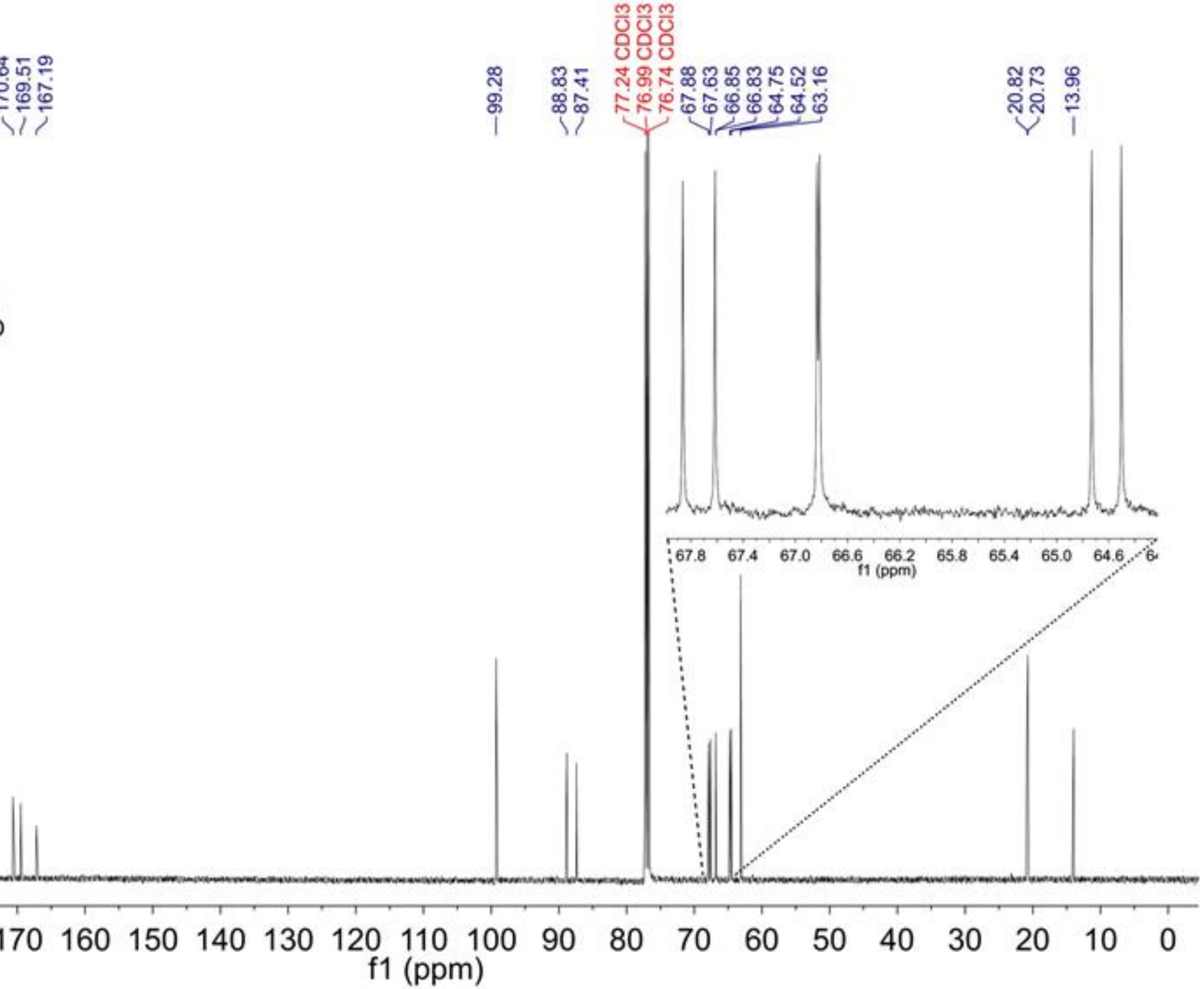

^{13}C {^{1}H} NMR (125 MHz, $CDCl_3$) of **41**

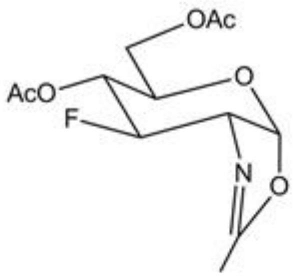

41

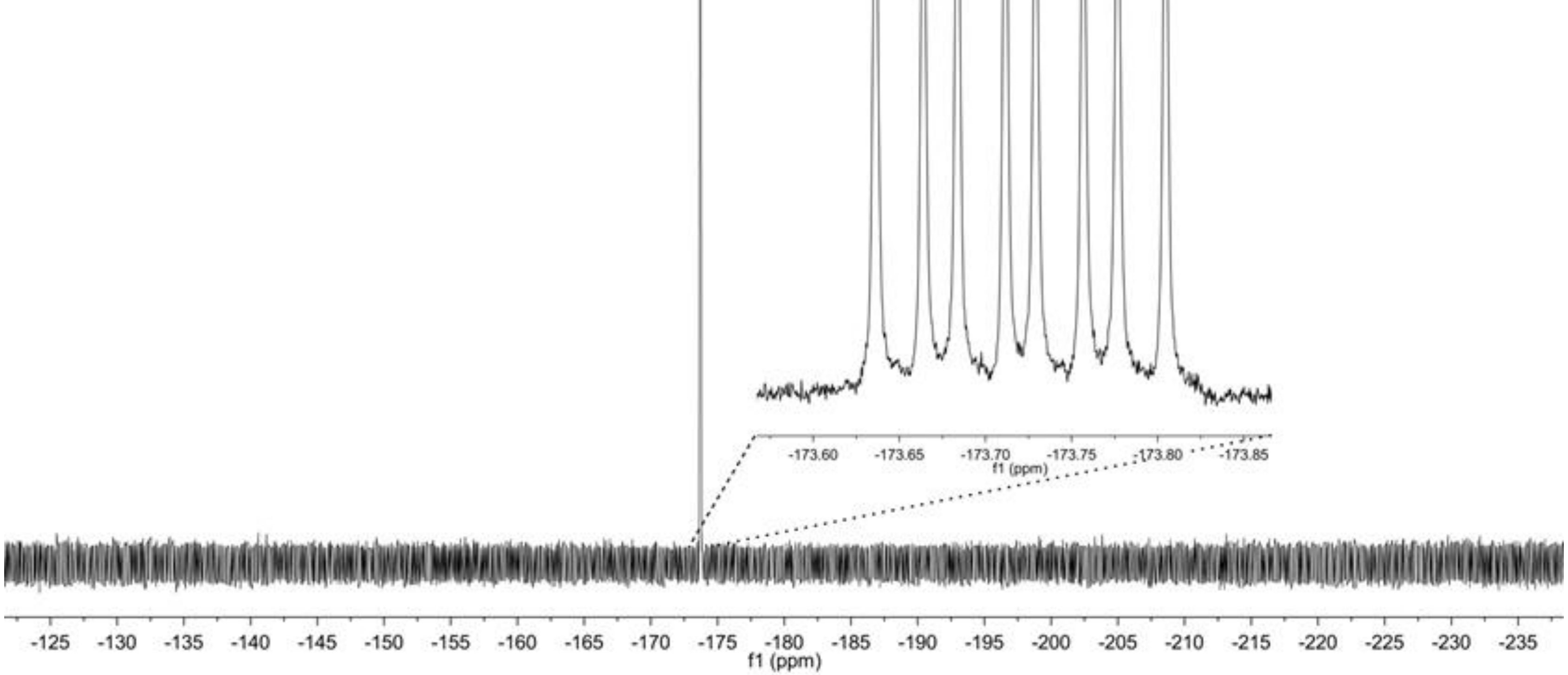

^{19}F NMR (470 MHz, $CDCl_3$) of **41**

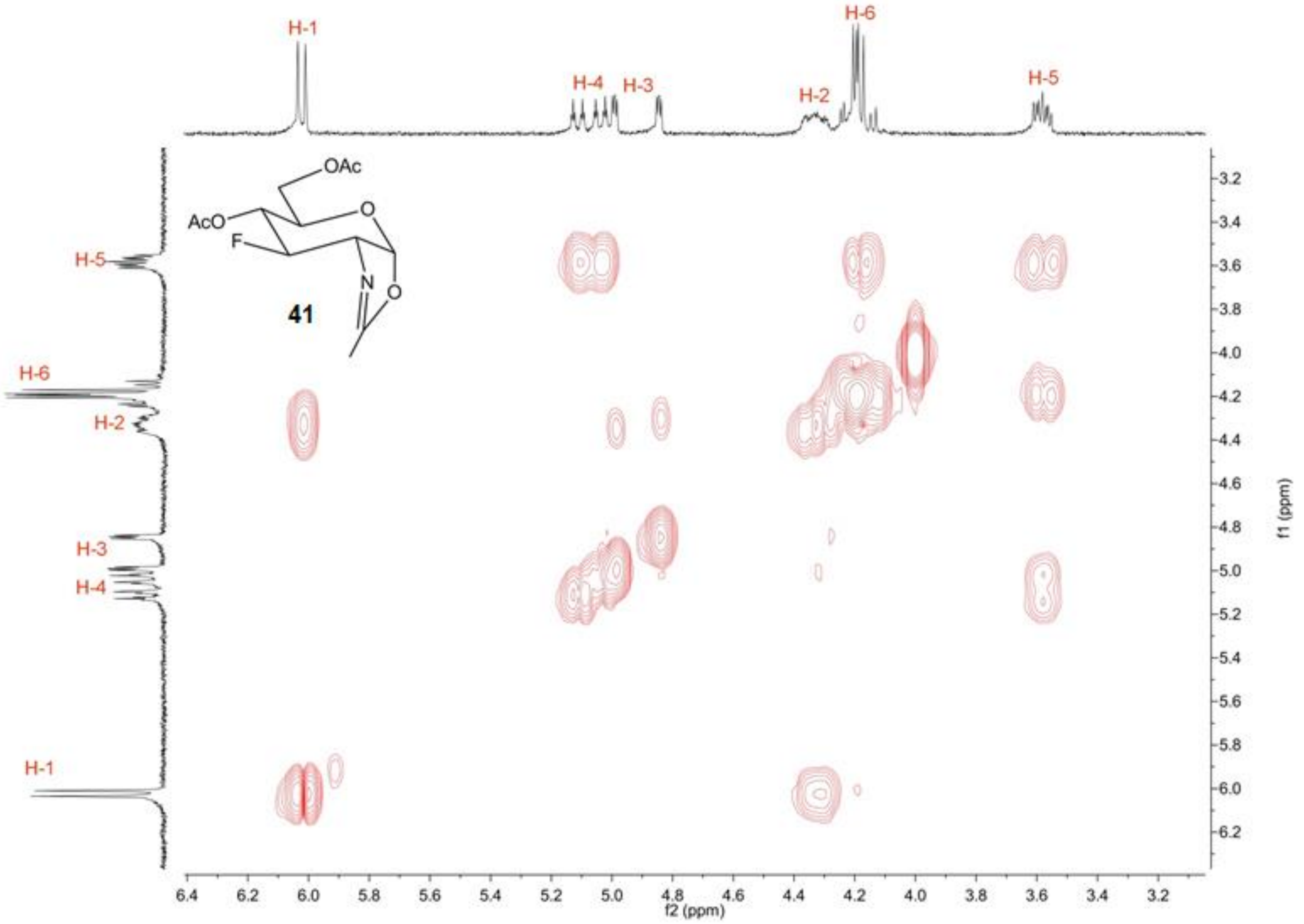

gCOSY NMR (500 MHz, $CDCl_3$) of **41**

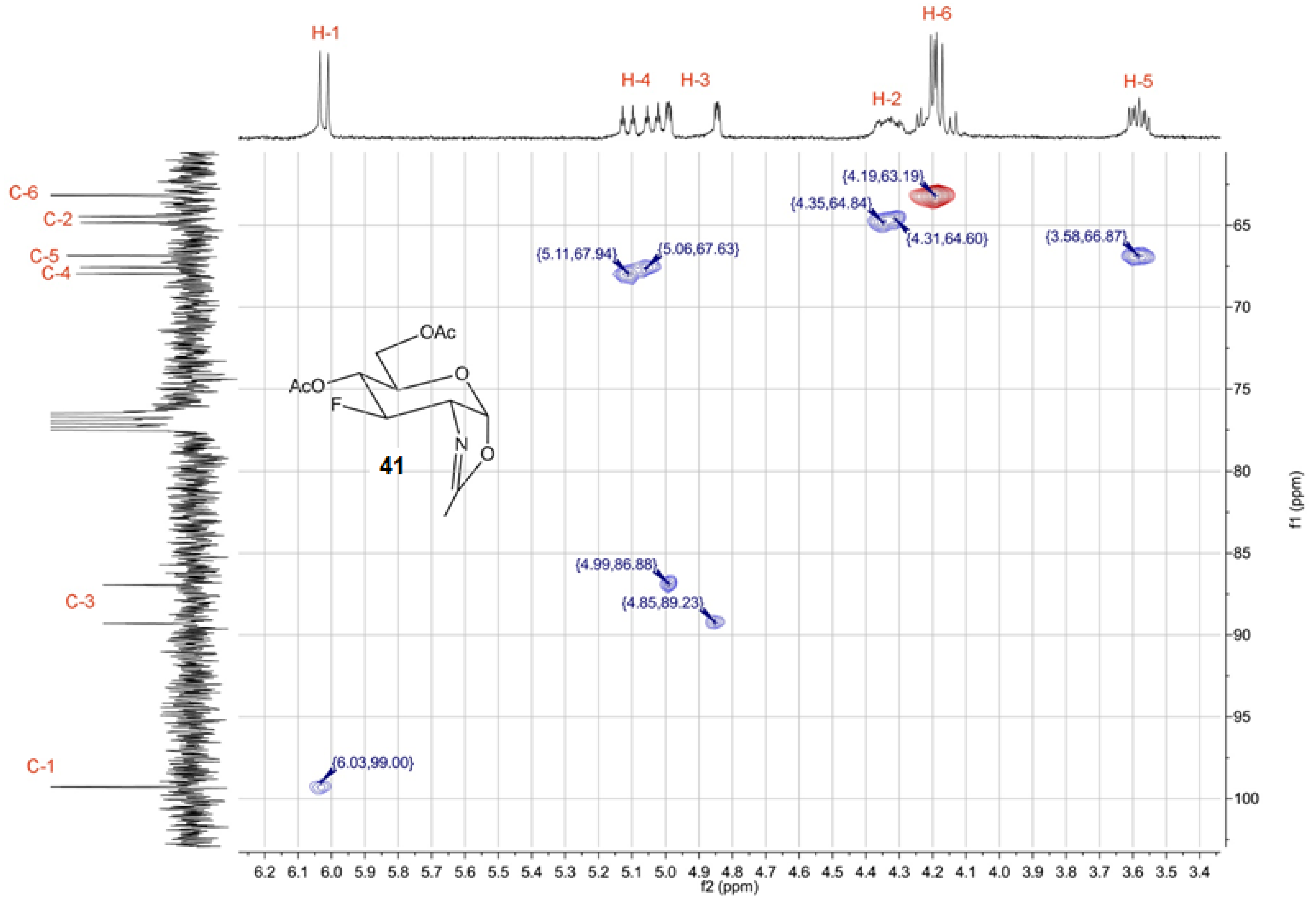

gHSQC NMR (500 MHz, $CDCl_3$) of **41**

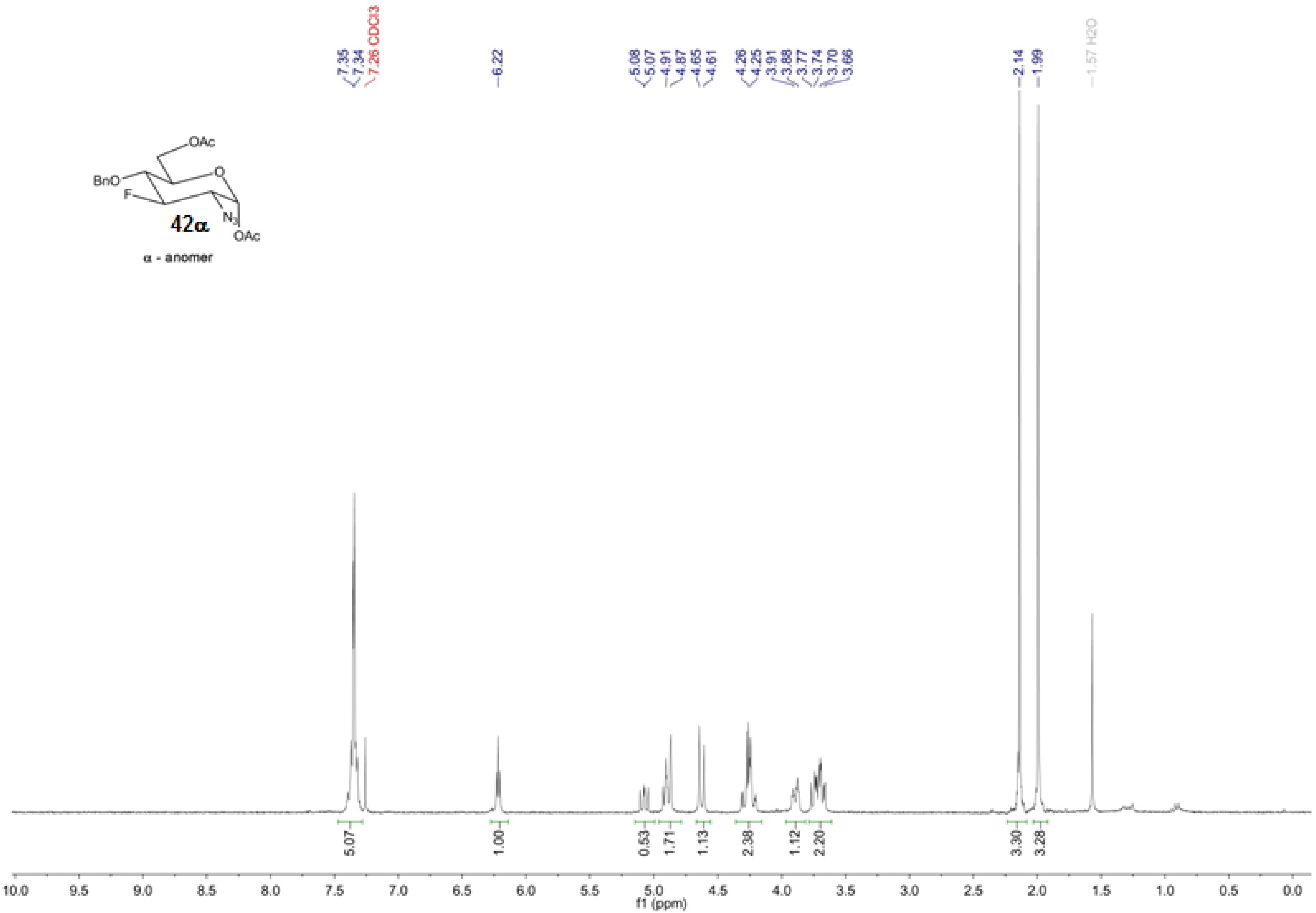

^{1}H NMR (300 MHz, $CDCl_3$) of **42α (**α-anomer)

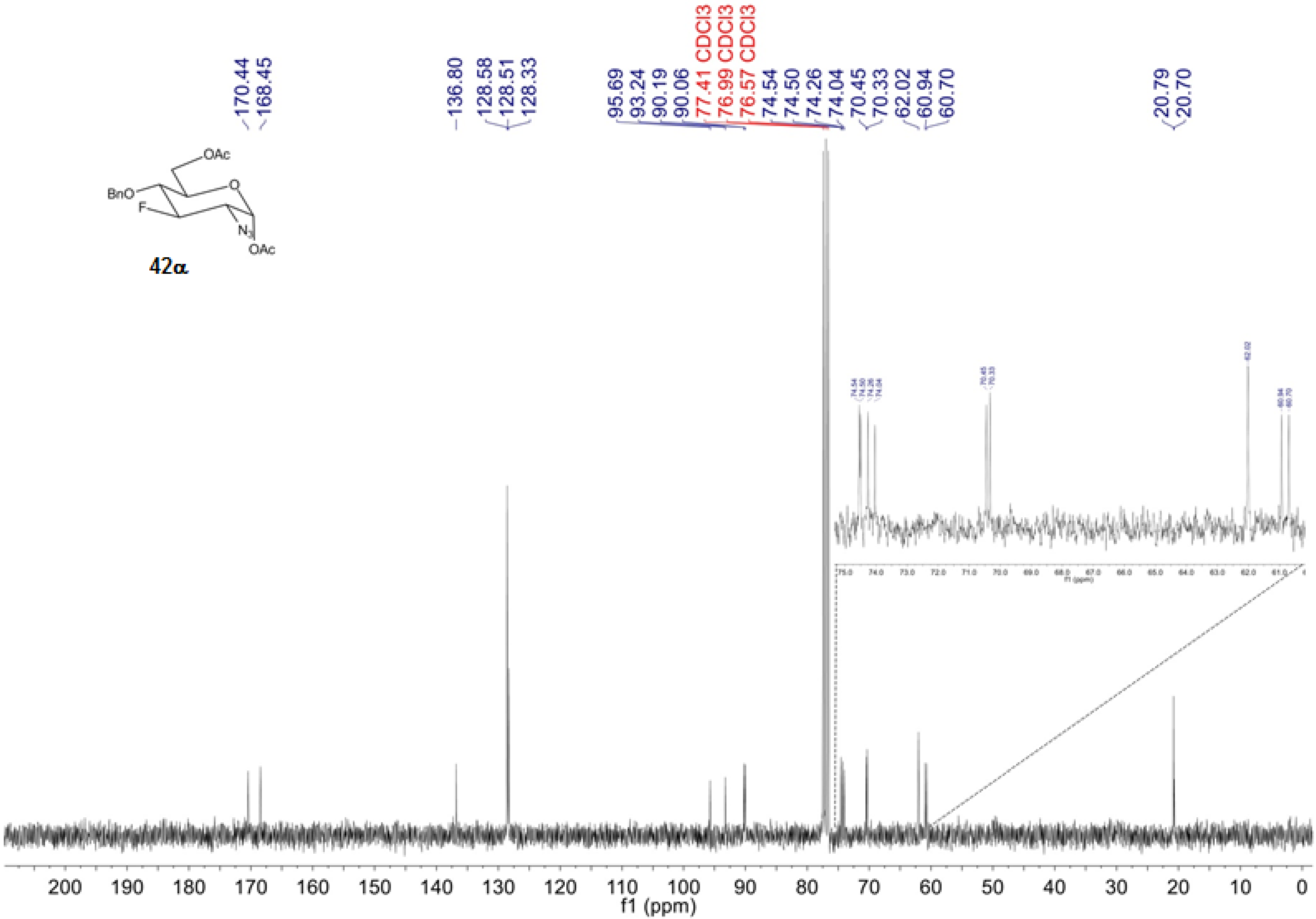

^{13}C {^{1}H} NMR (75 MHz, $CDCl_3$) of **42α** (α-anomer)

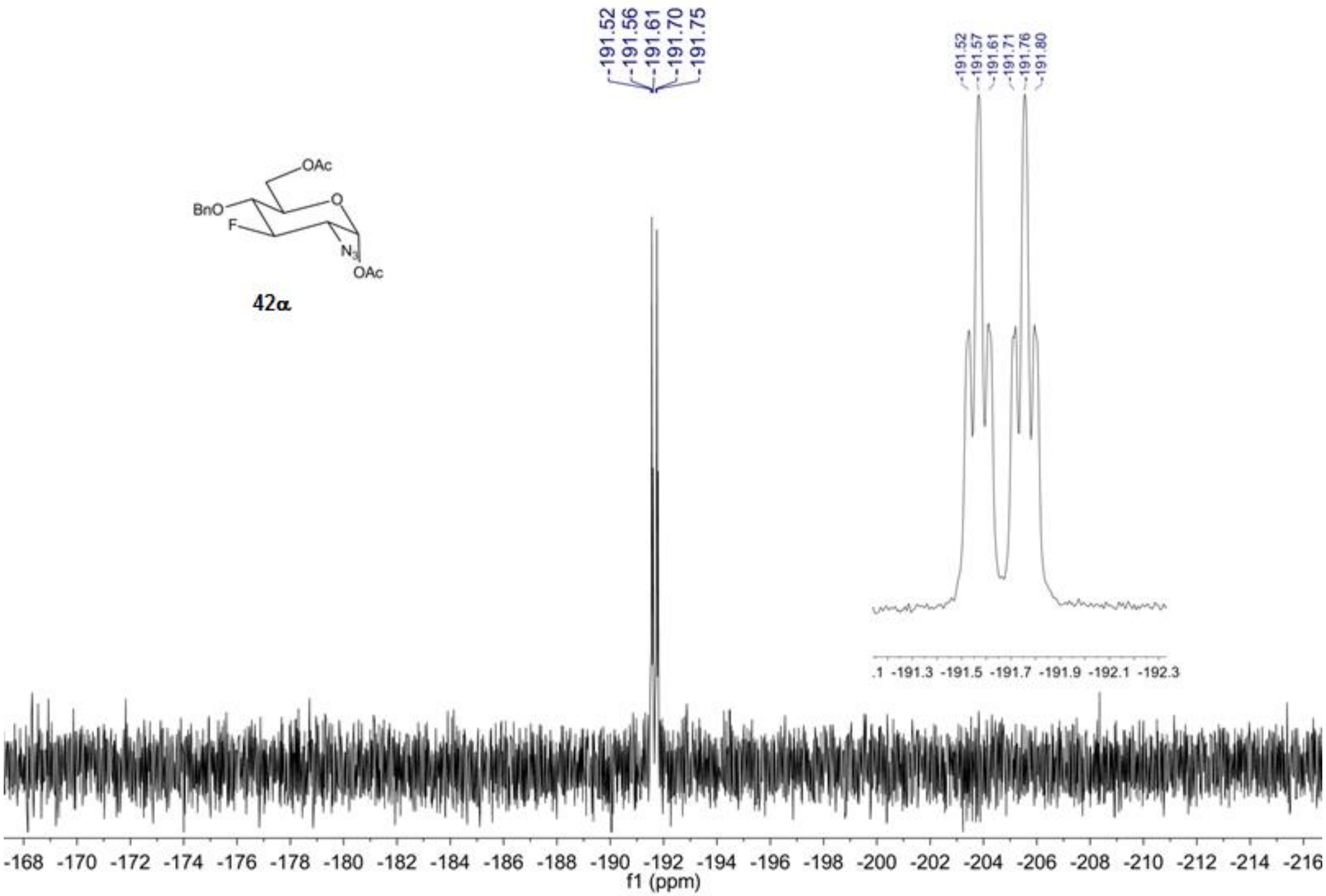

^{19}F NMR (470 MHz, $CDCl_3$) of **42α** (α-anomer)

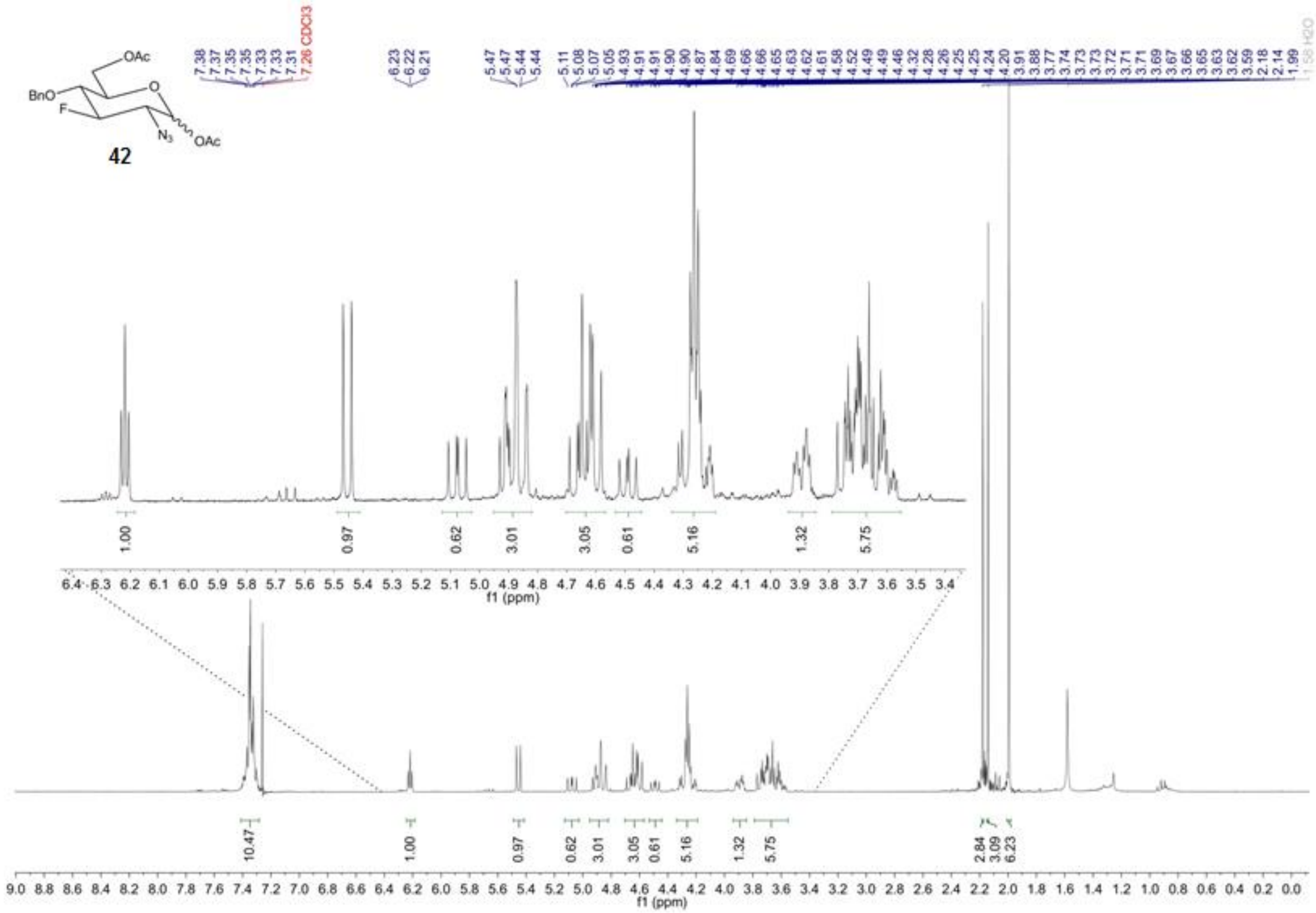

^{1}H NMR (300 MHz, $CDCl_3$) of **42** (α-anomer+β-anomer)

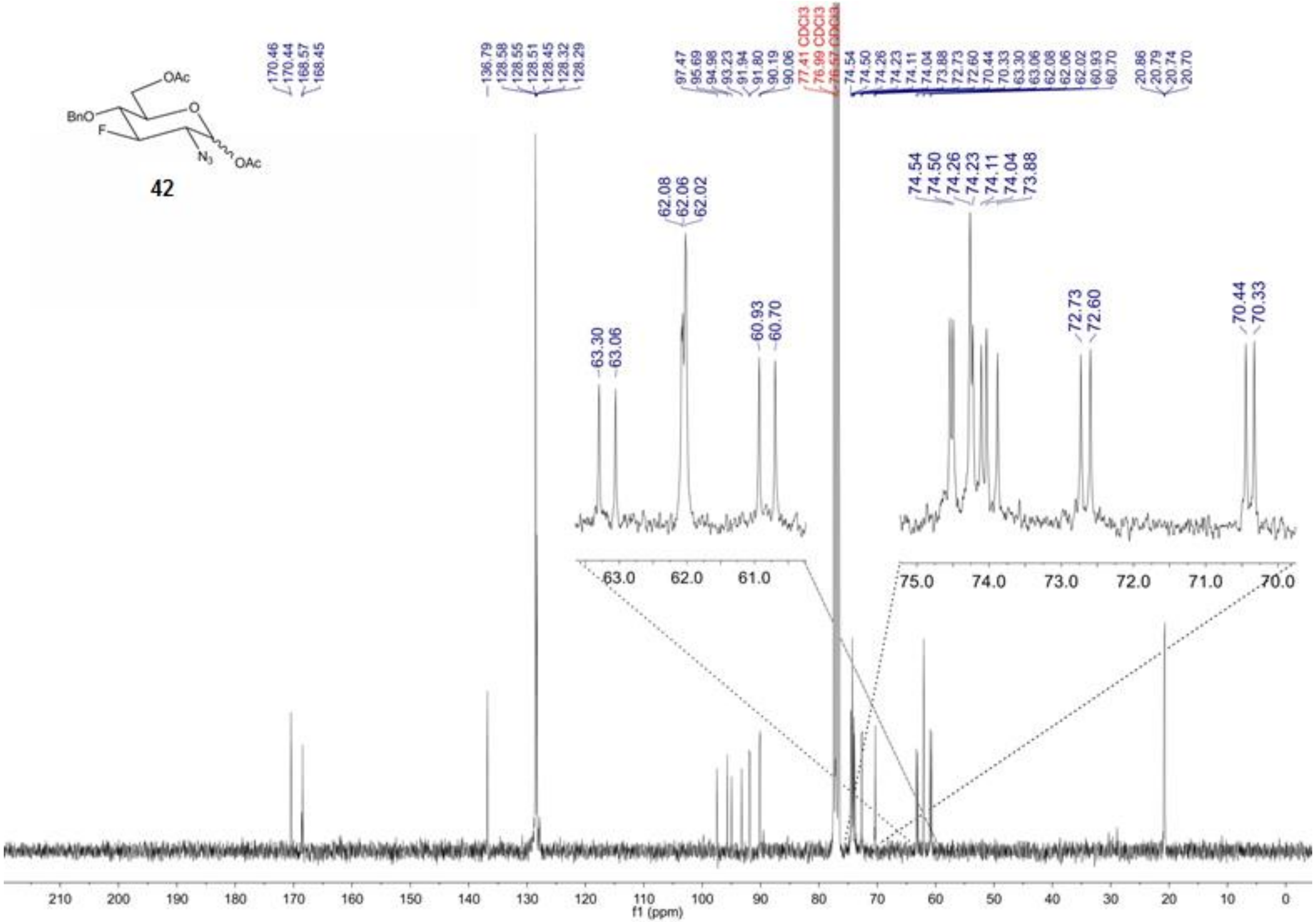

^{13}C {^{1}H} NMR (75 MHz, $CDCl_3$) of **42** (α-anomer+β-anomer)

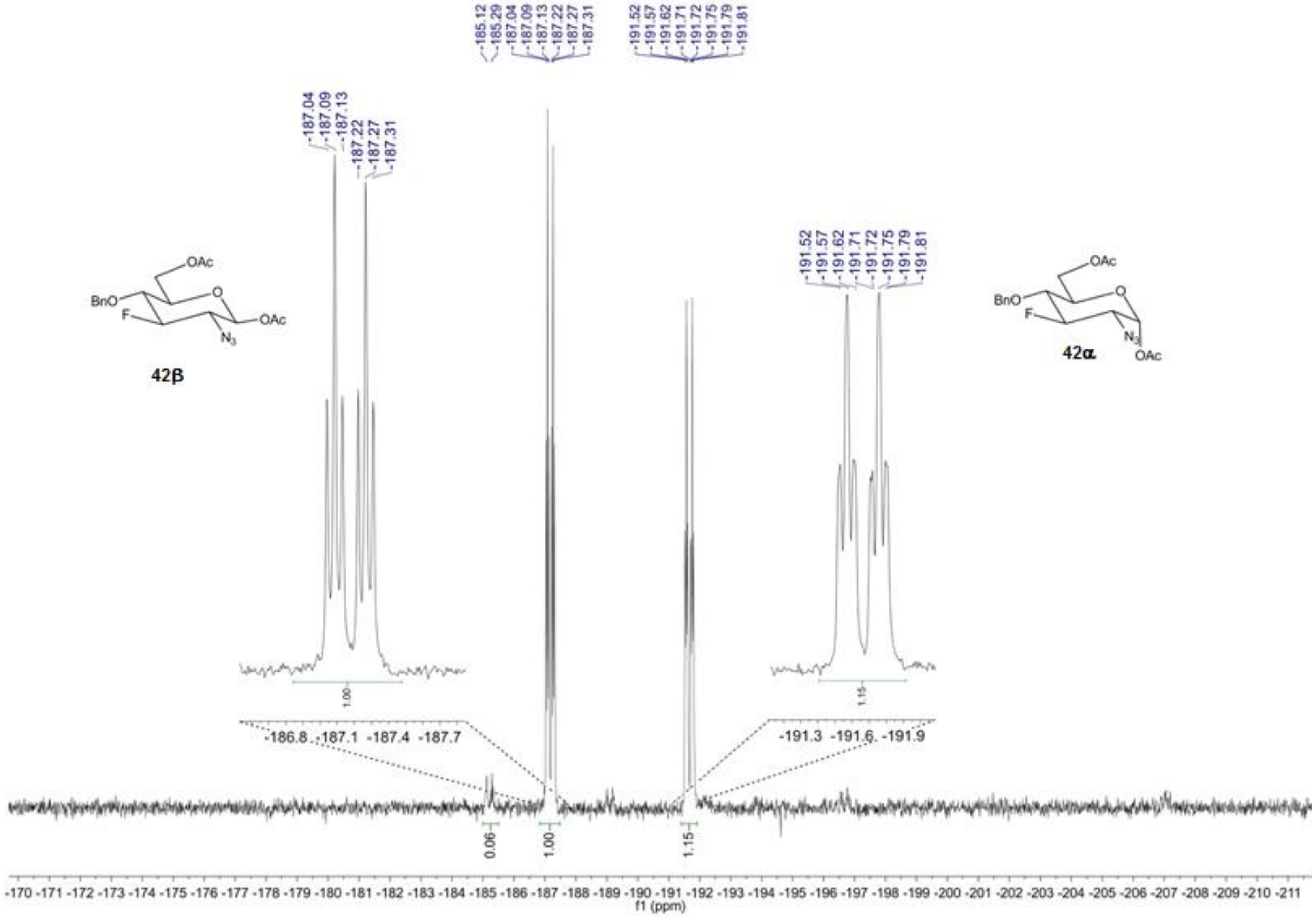

^{19}F NMR (282 MHz, $CDCl_3$) of **42** (α-anomer+β-anomer)

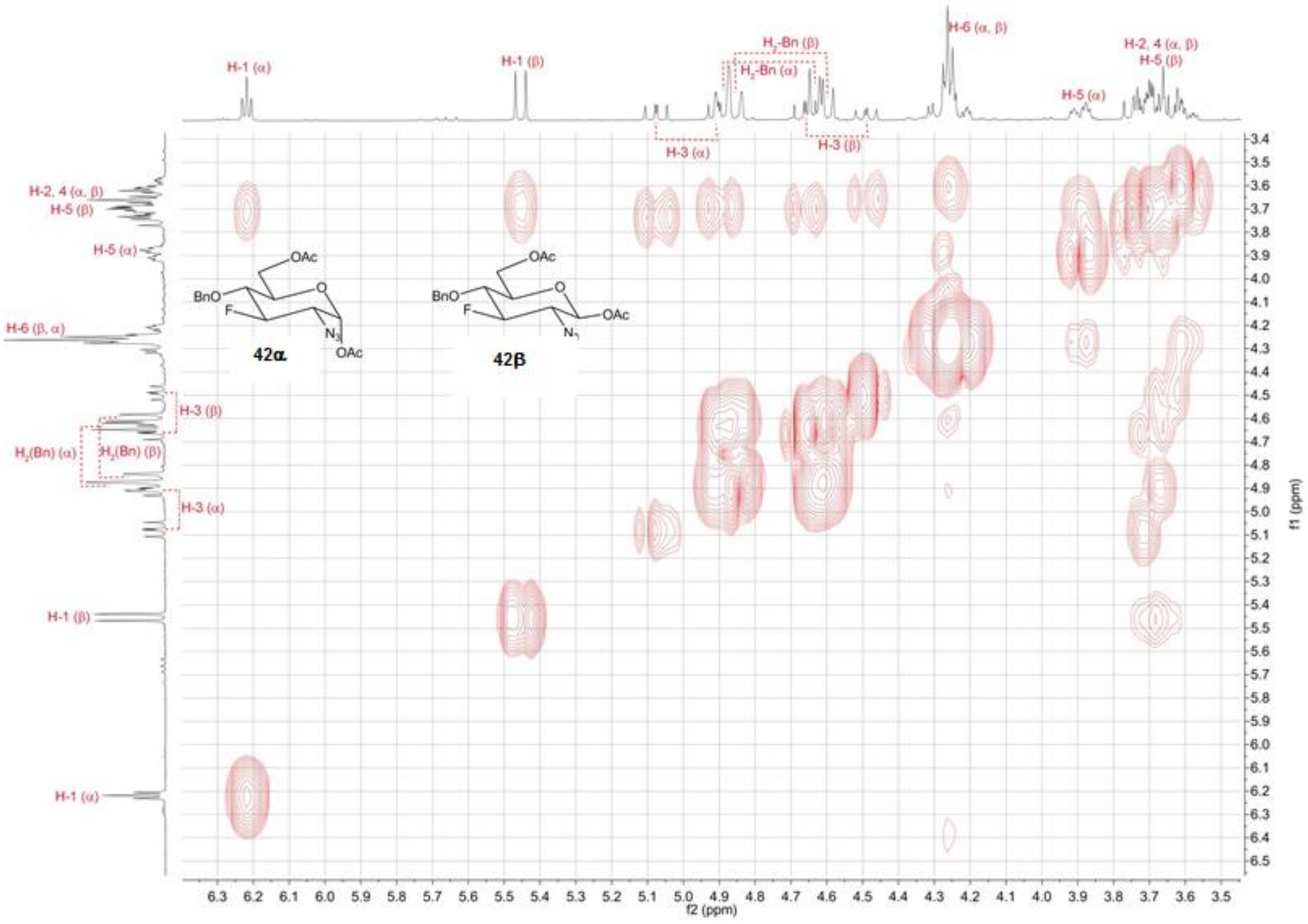

gCOSY NMR (300 MHz, $CDCl_3$) of **42** (α-anomer/β-anomer ca 1:1)

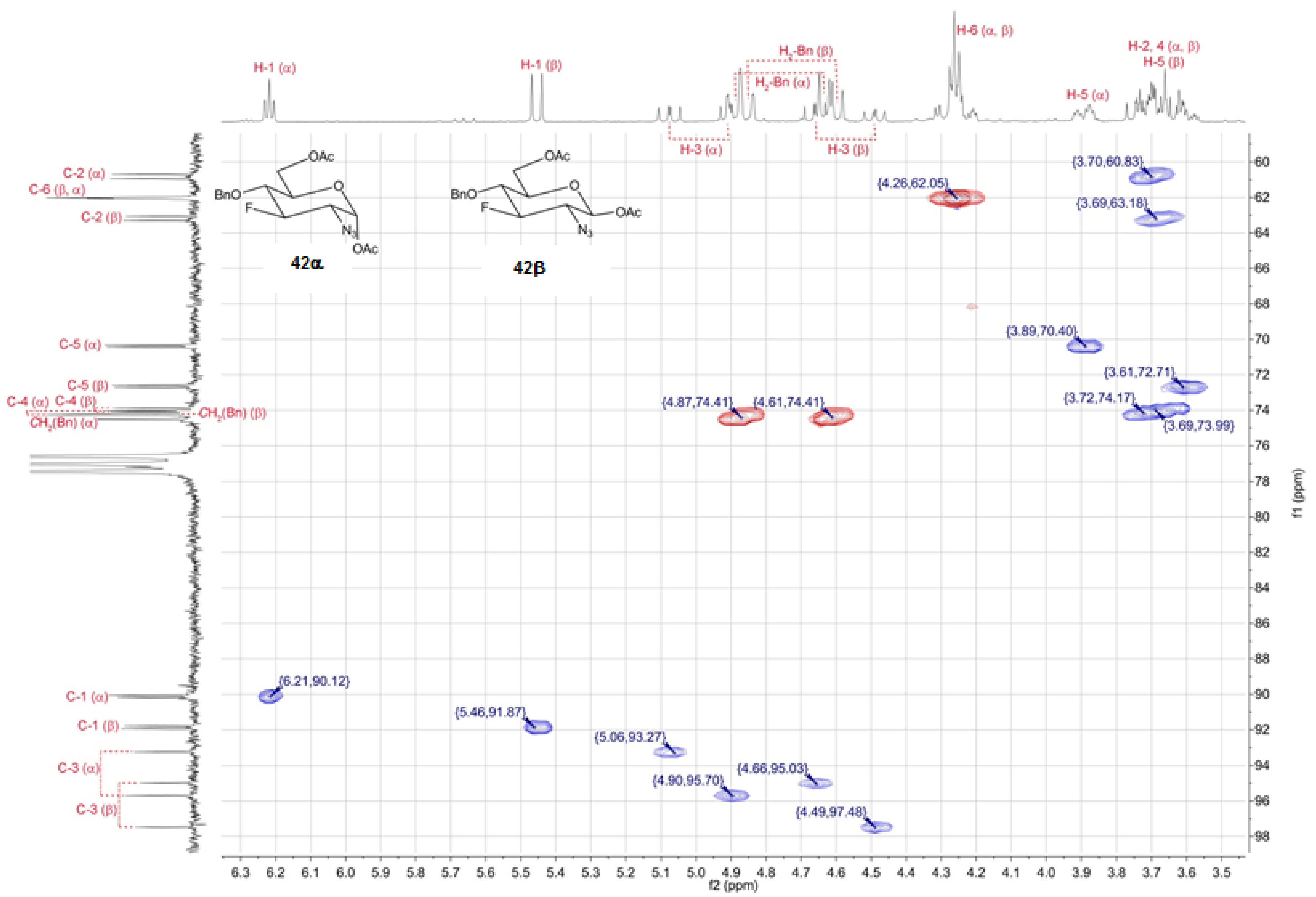

gHSQC NMR (300 MHz, $CDCl_3$) of **42** (α-anomer/β-anomer ca 1:1)

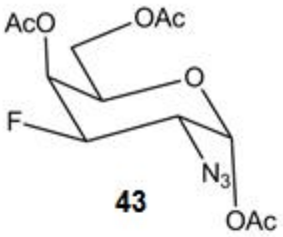

43

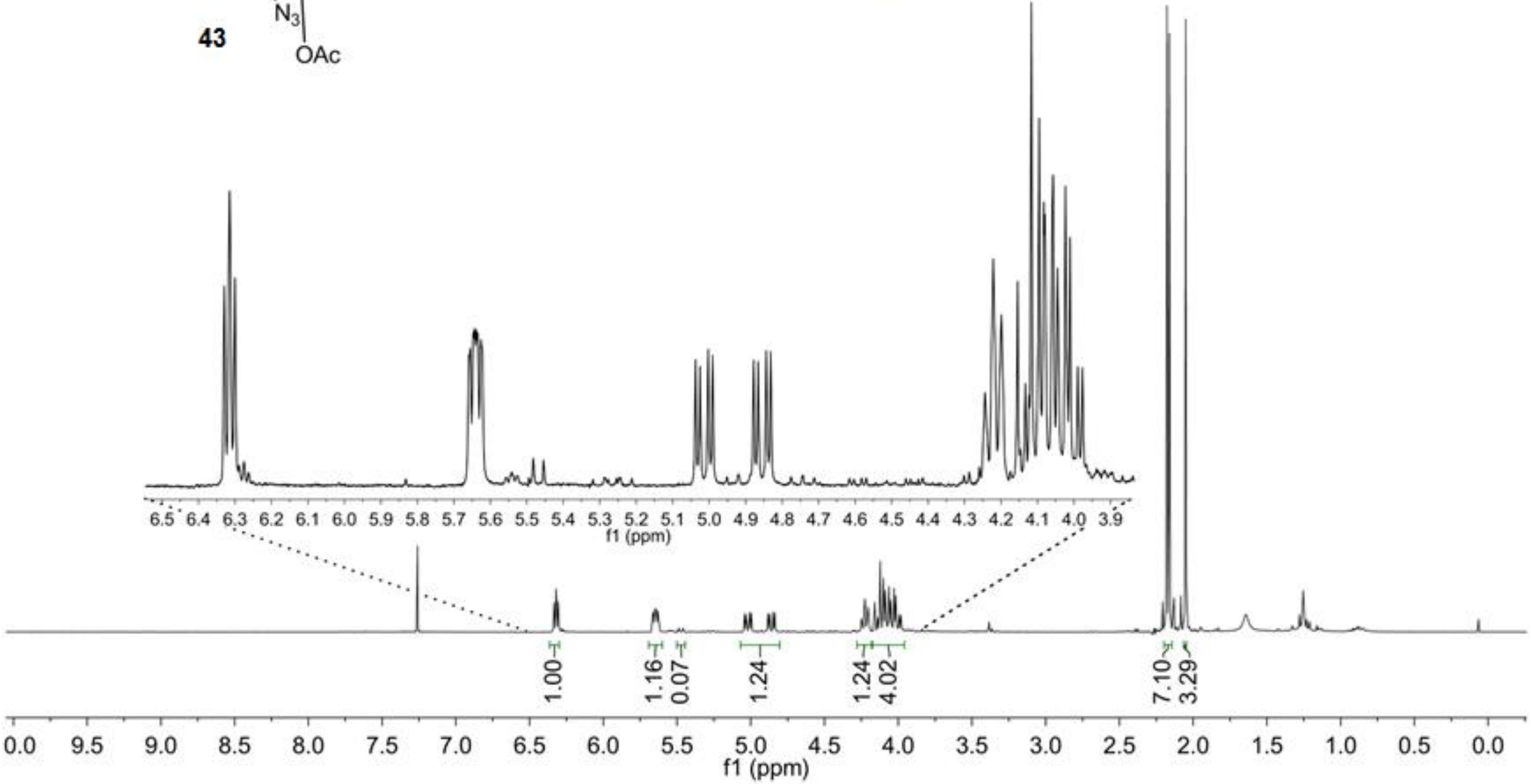

^{1}H NMR (300 MHz, $CDCl_3$) of **43** (α-anomer and ca. 10% β-anomer)

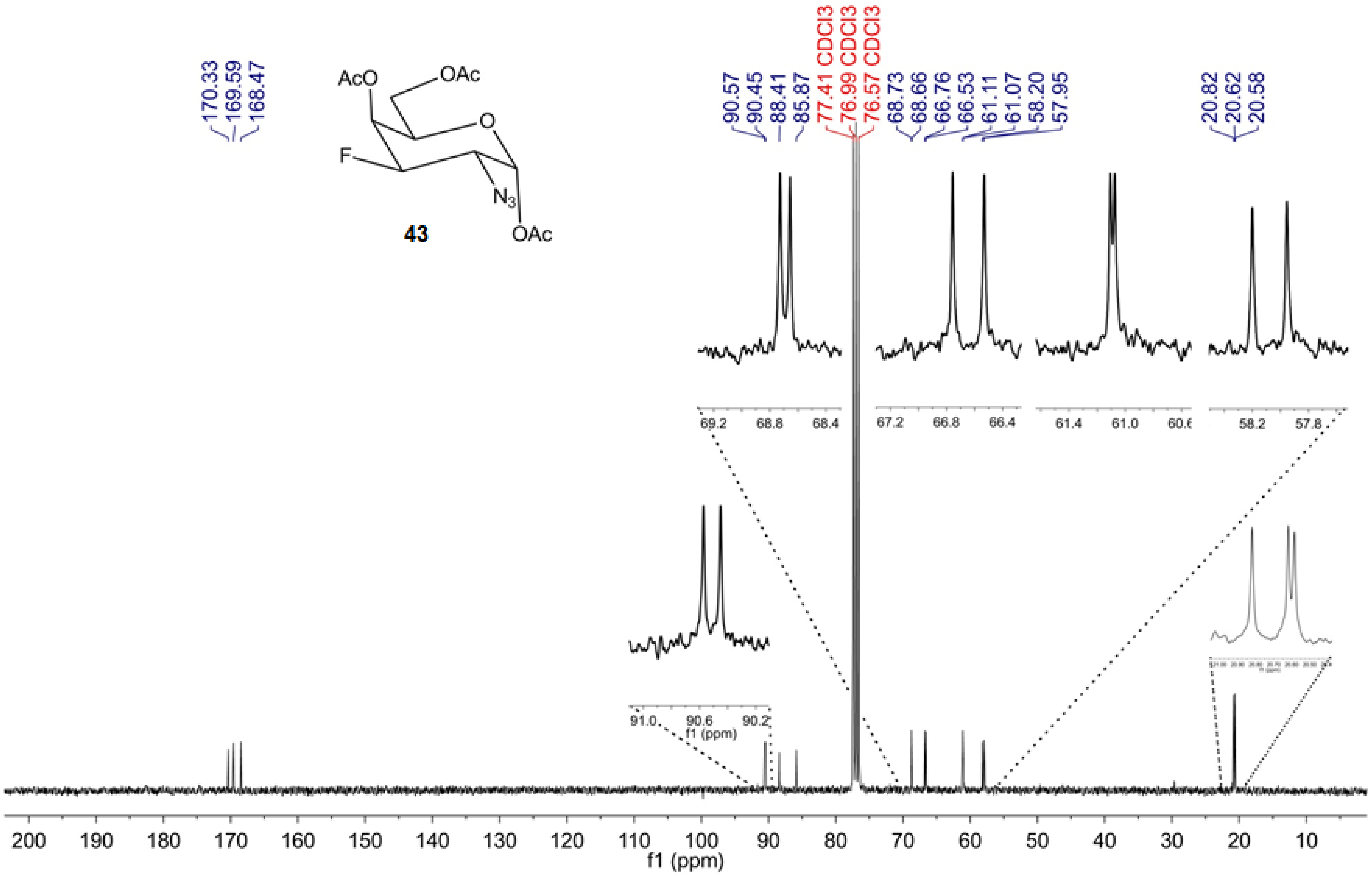

$^{13}C\ \{^{1}H\}$ NMR (75 MHz, $CDCl_3$) of **43** (α-anomer and ca. 10% β-anomer).

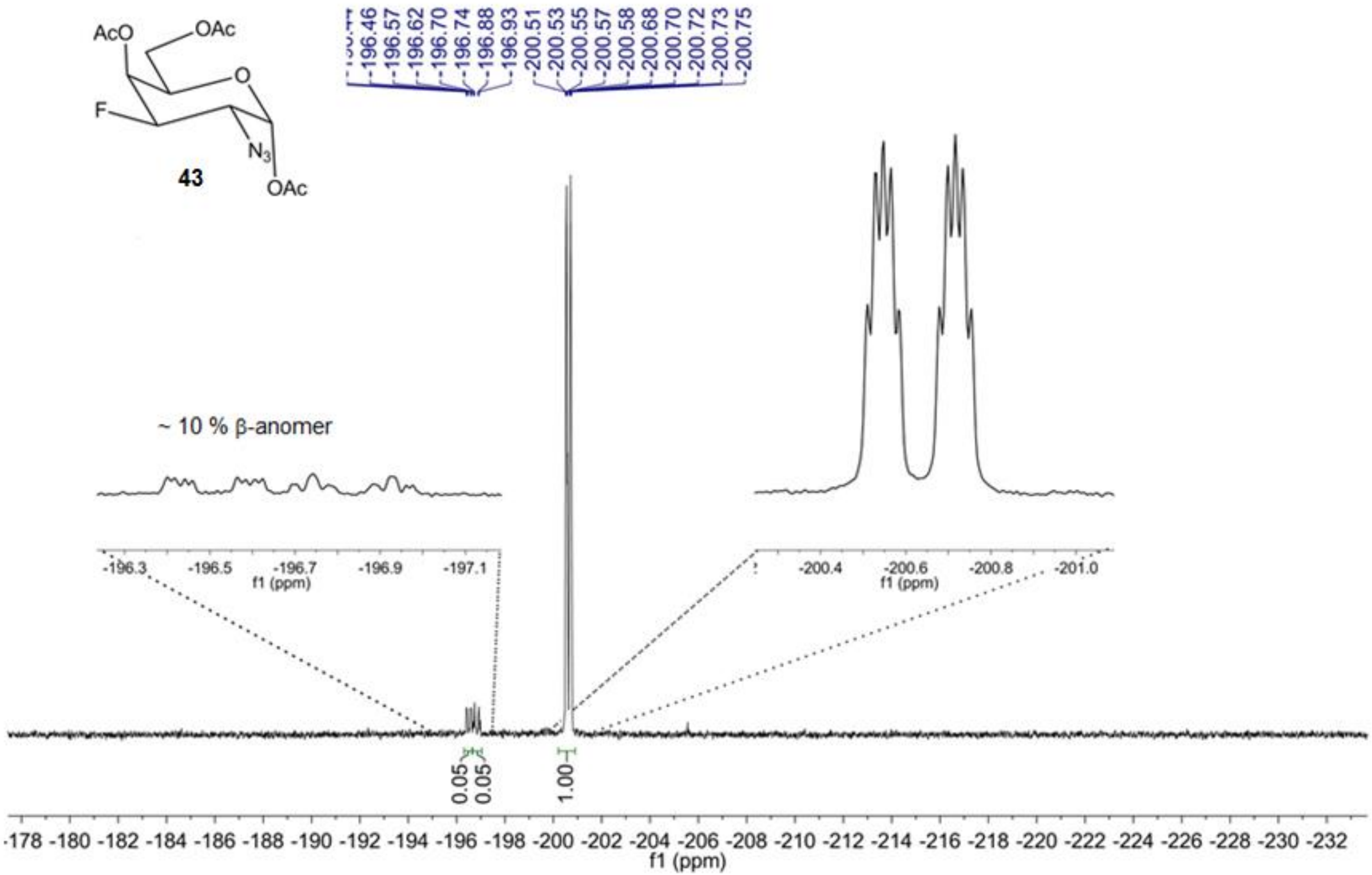

^{19}F NMR (282 MHz, $CDCl_3$) of **43** (α-anomer and ca. 10% β-anomer)

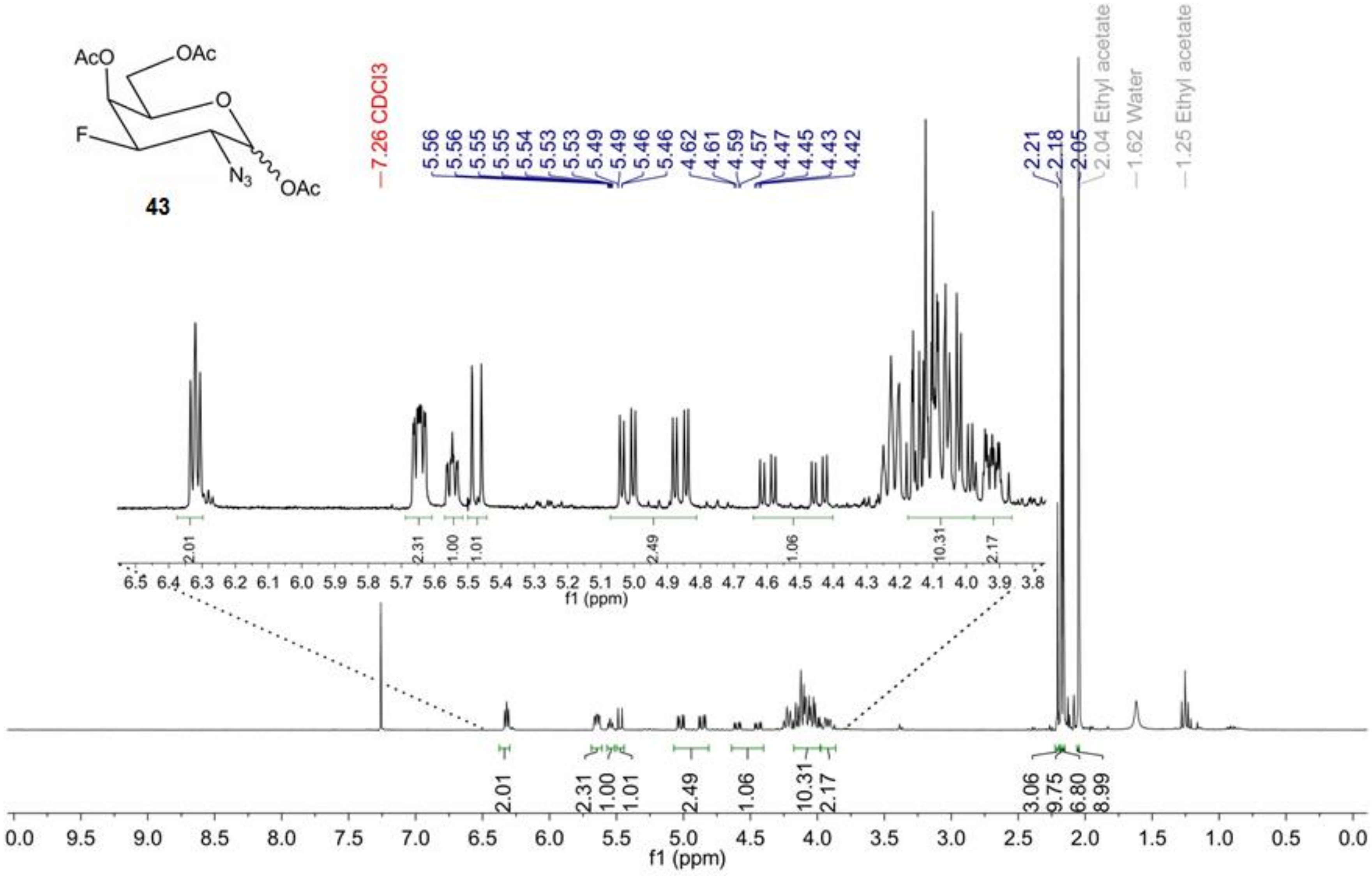

^{1}H NMR (300 MHz, $CDCl_3$) of **43** (α/β ca 3:7)

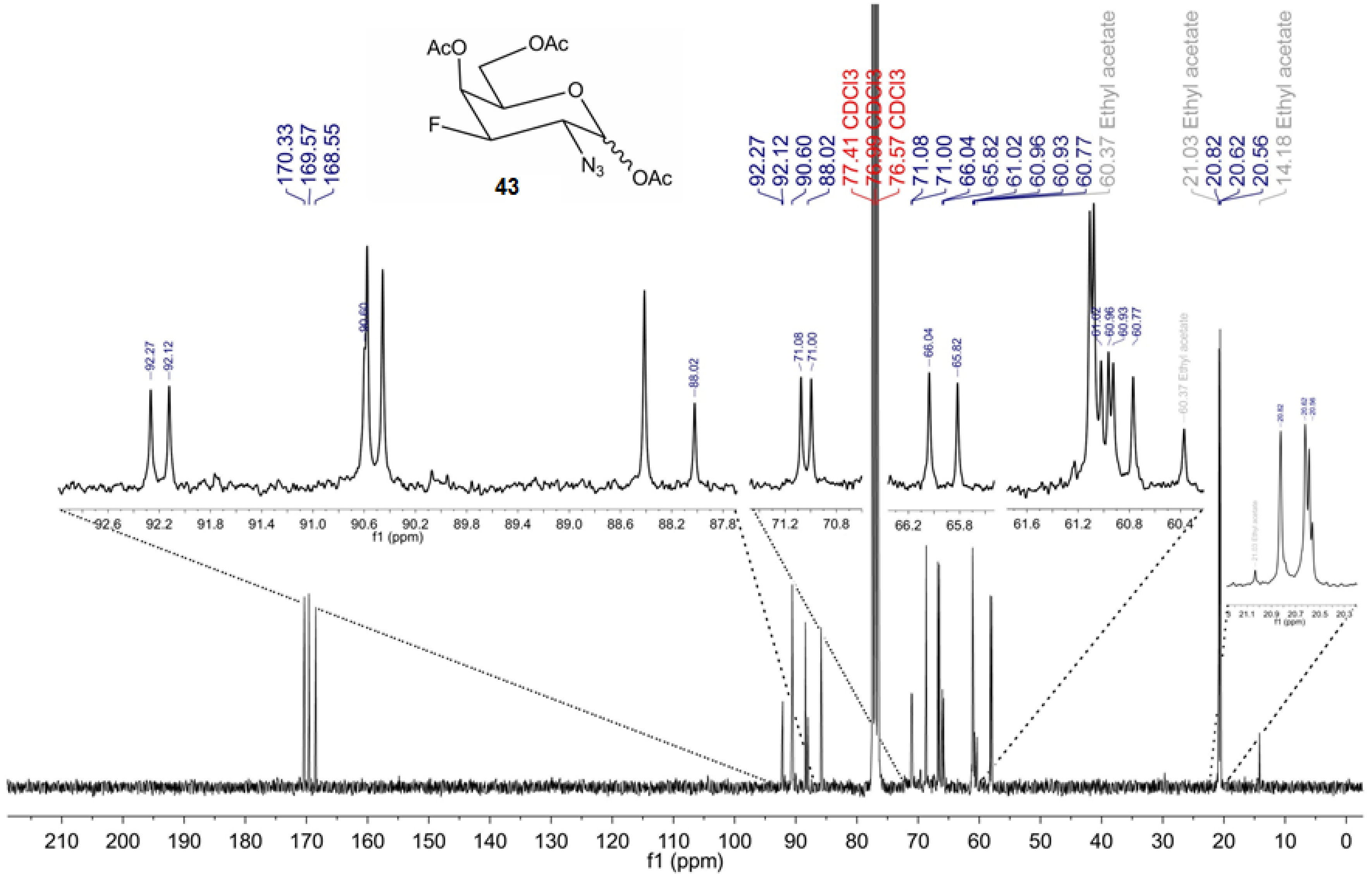

^{13}C {^{1}H} NMR (75 MHz, $CDCl_3$) of **43** (α/β ca 3:7)

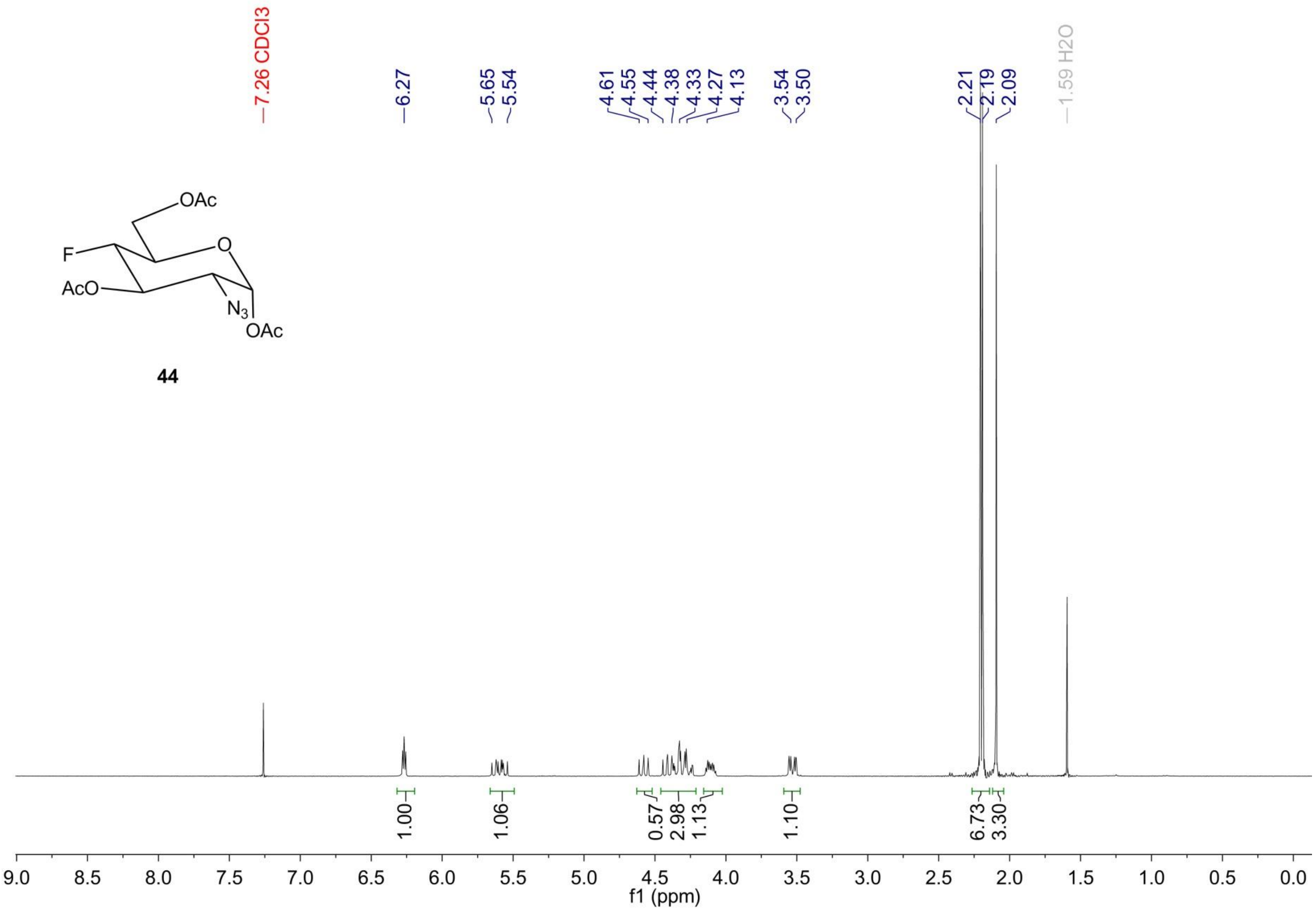

44

Figure SII-90. ^{1}H NMR (300 MHz, $CDCl_3$) of **44** (α-anomer)

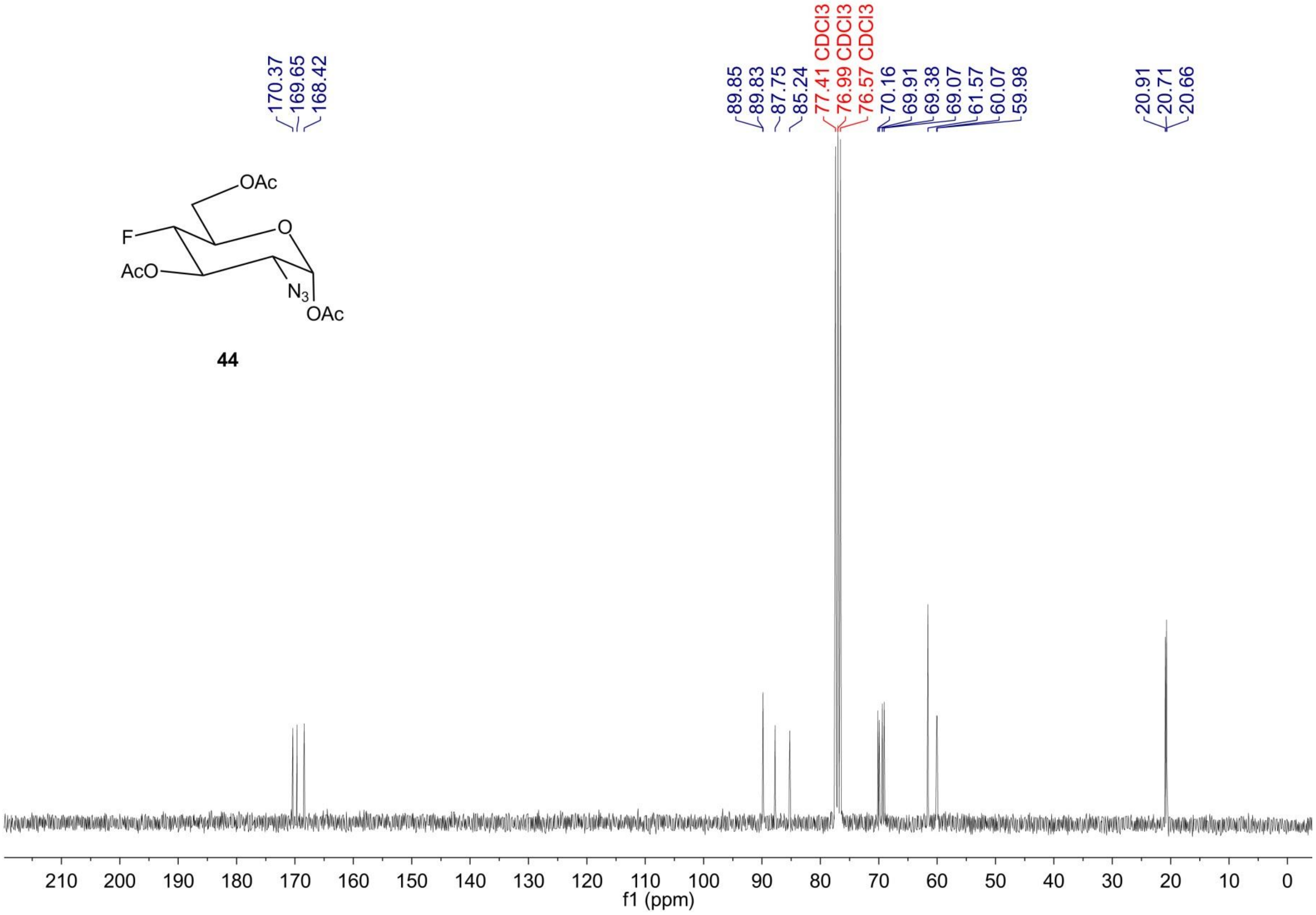

Figure SII-91. ^{13}C {^{1}H} NMR (75 MHz, $CDCl_3$) of **44** (α-anomer)

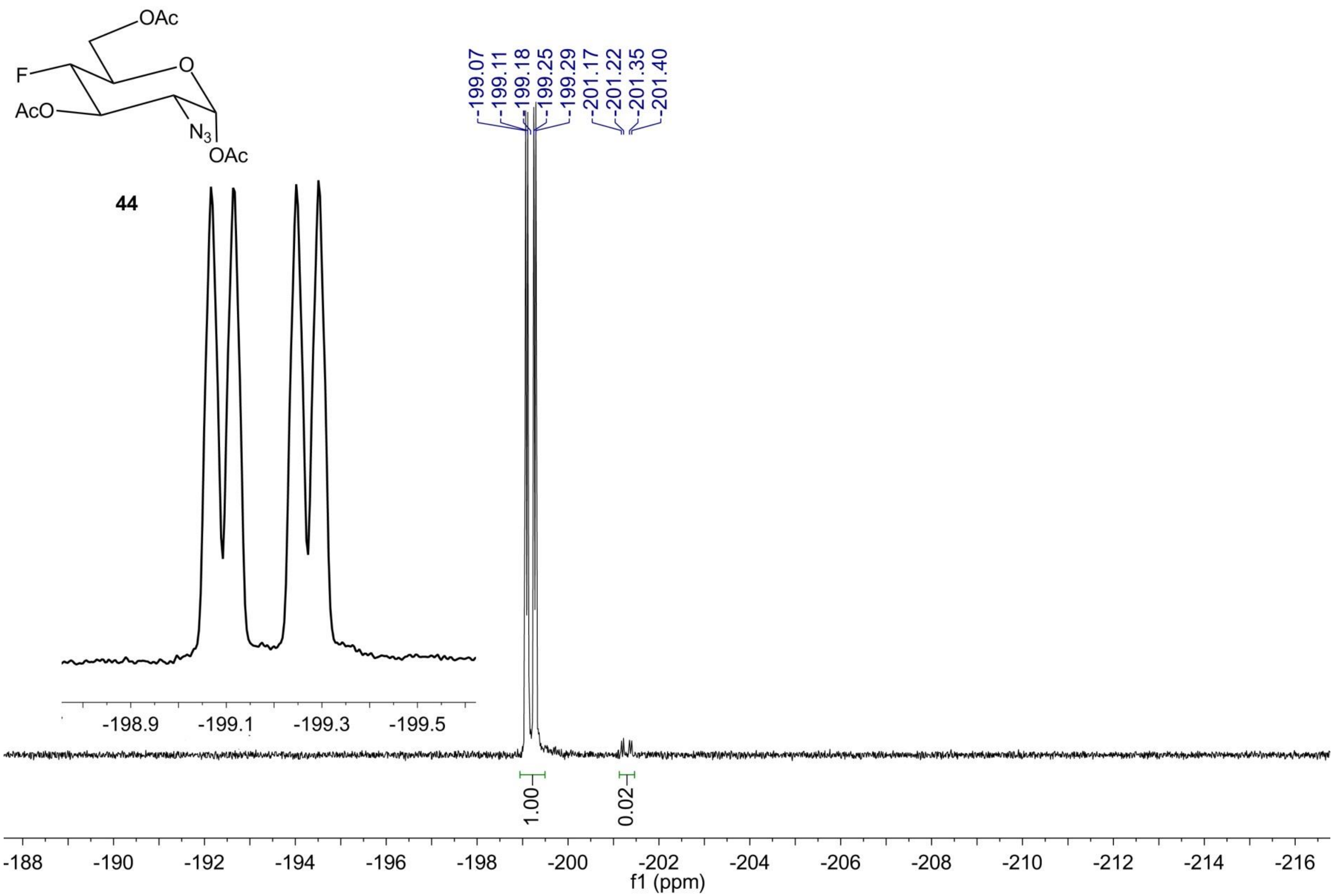

^{19}F NMR (282 MHz, $CDCl_3$) of **44** (α-anomer)

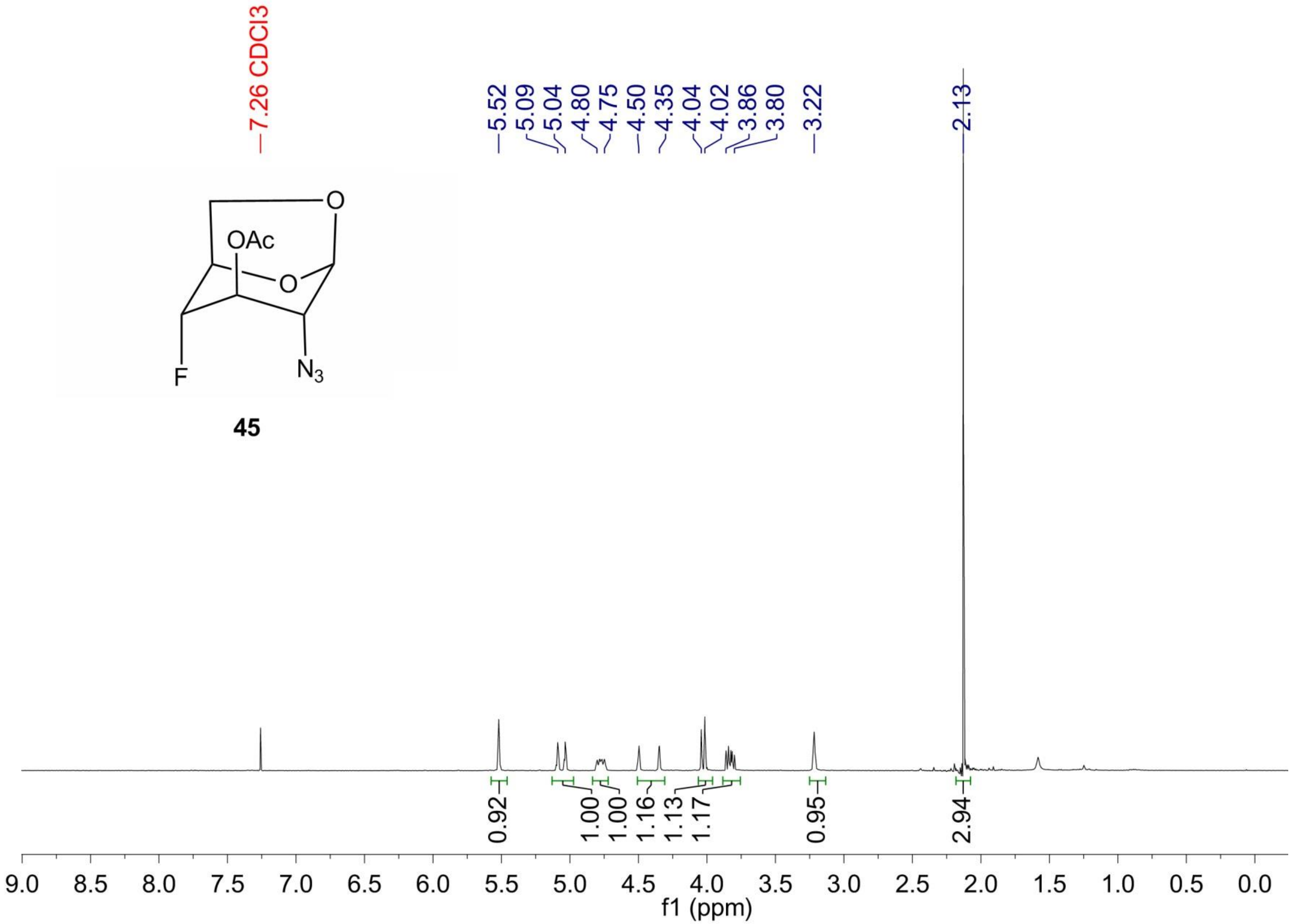

^{1}H NMR (300 MHz, $CDCl_3$) of **45**

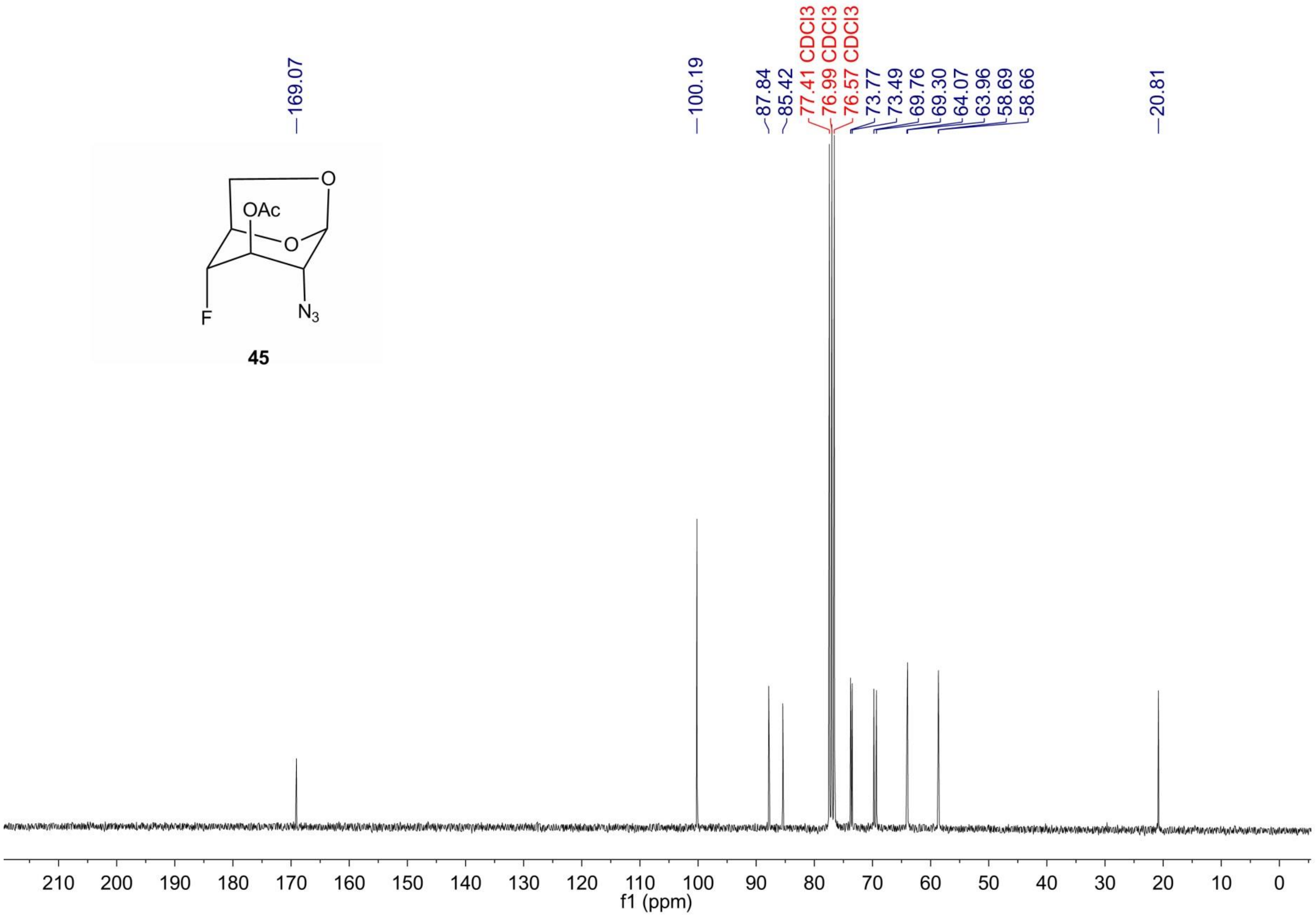

^{13}C {^{1}H} NMR (75 MHz, $CDCl_3$) of **45**

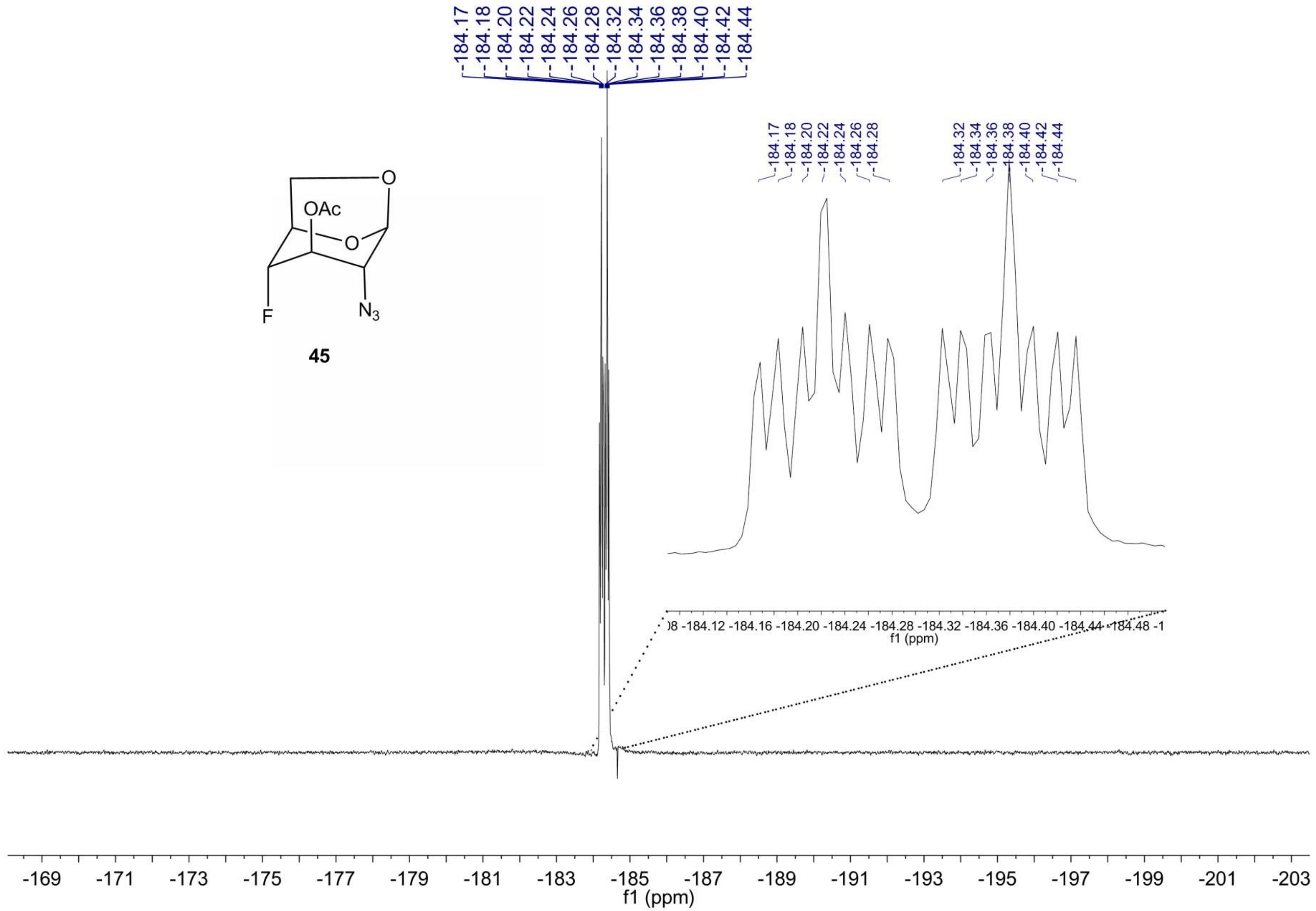

^{19}F NMR (282 MHz, $CDCl_3$) of **45**

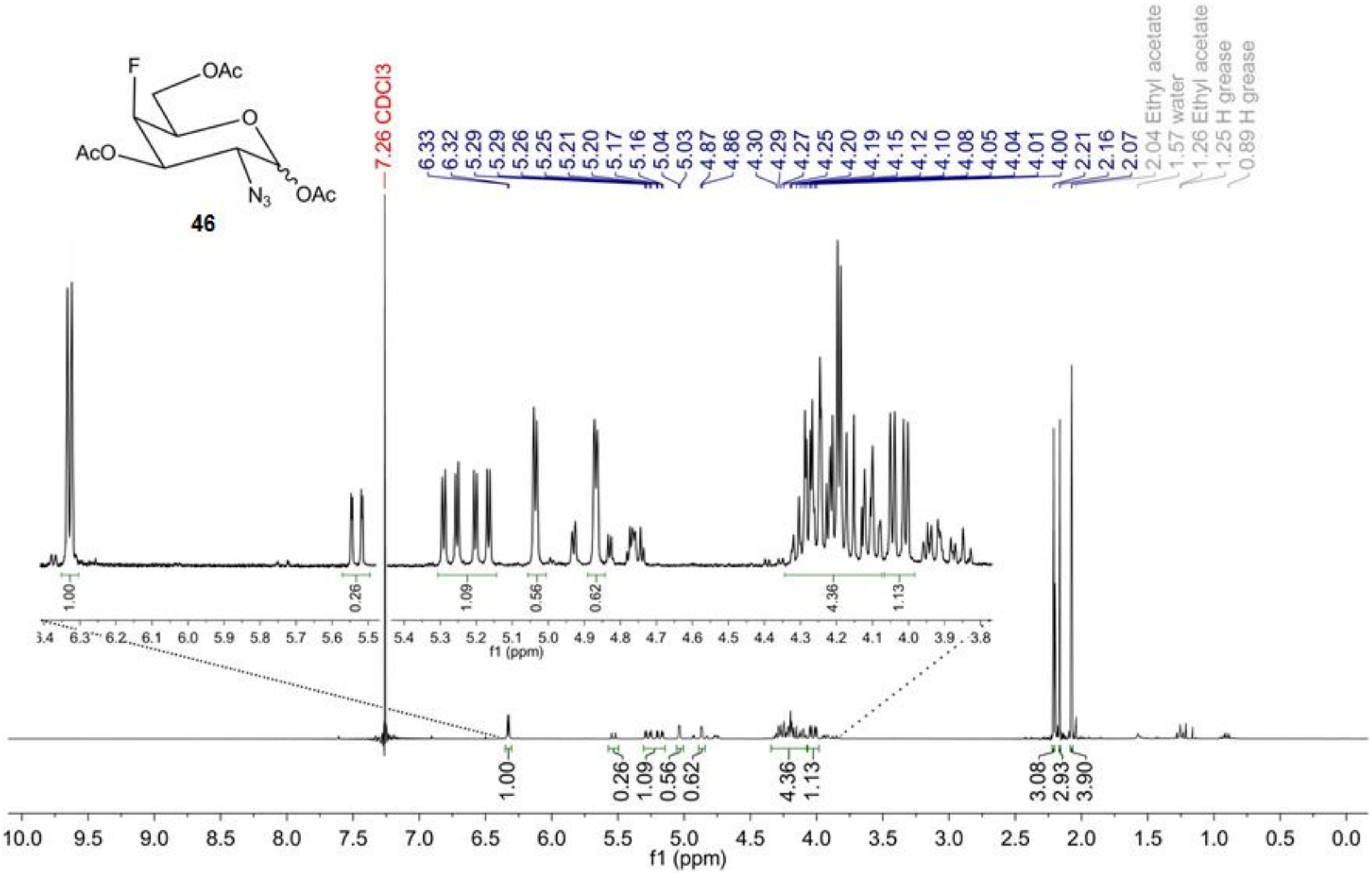

^{1}H NMR (300 MHz, $CDCl_3$) of **46** (α-anomer/β-anomer ca 1:0.3)

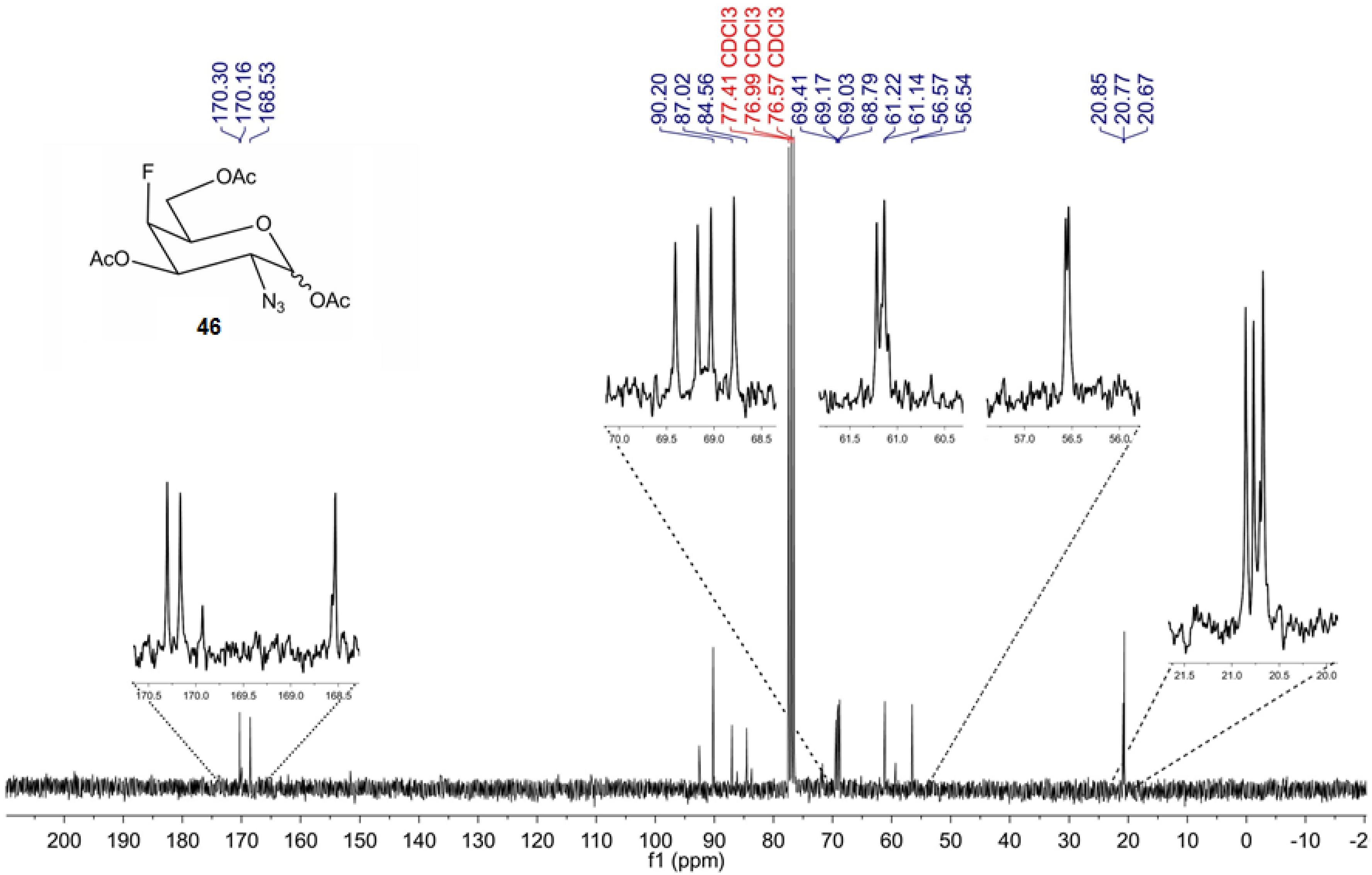

^{13}C {^{1}H} NMR (75 MHz, $CDCl_3$) of **46** (α-anomer/β-anomer ca 1:0.3)

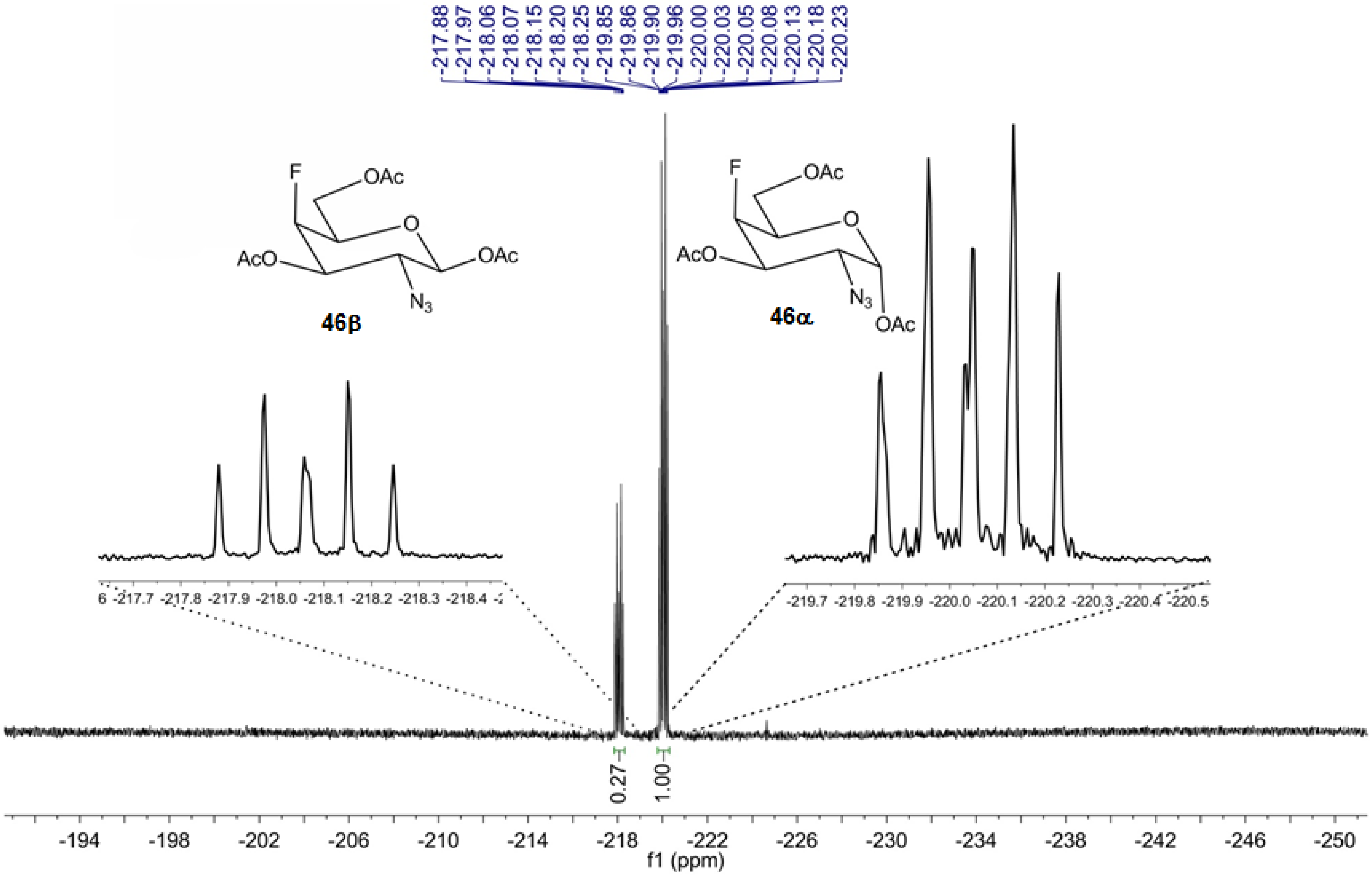

^{19}F NMR (282 MHz, $CDCl_3$) of **46** (α-anomer/β-anomer ca 1:0.3)

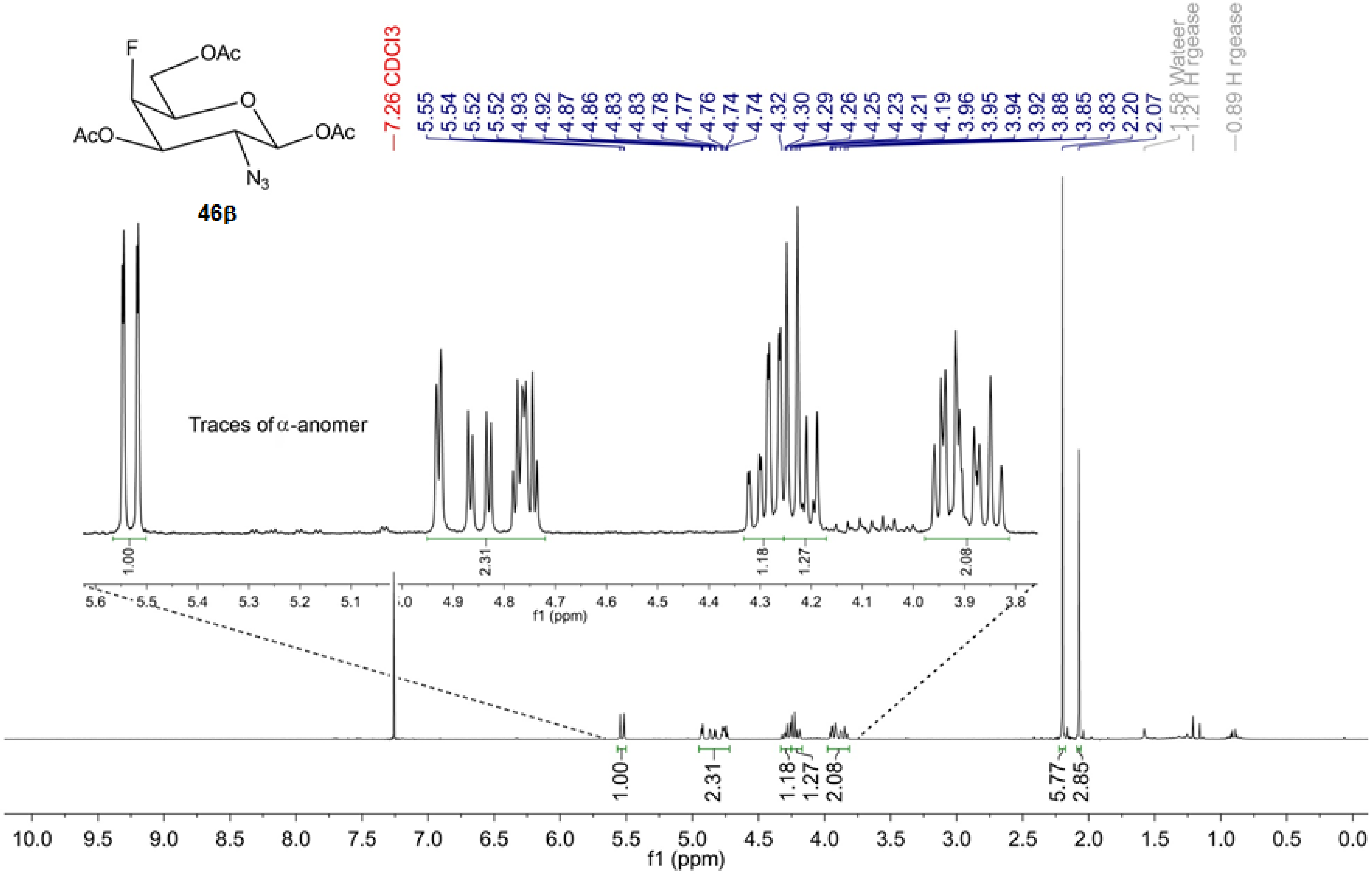

^{1}H NMR (300 MHz, $CDCl_3$) of **46β** (β-anomer)

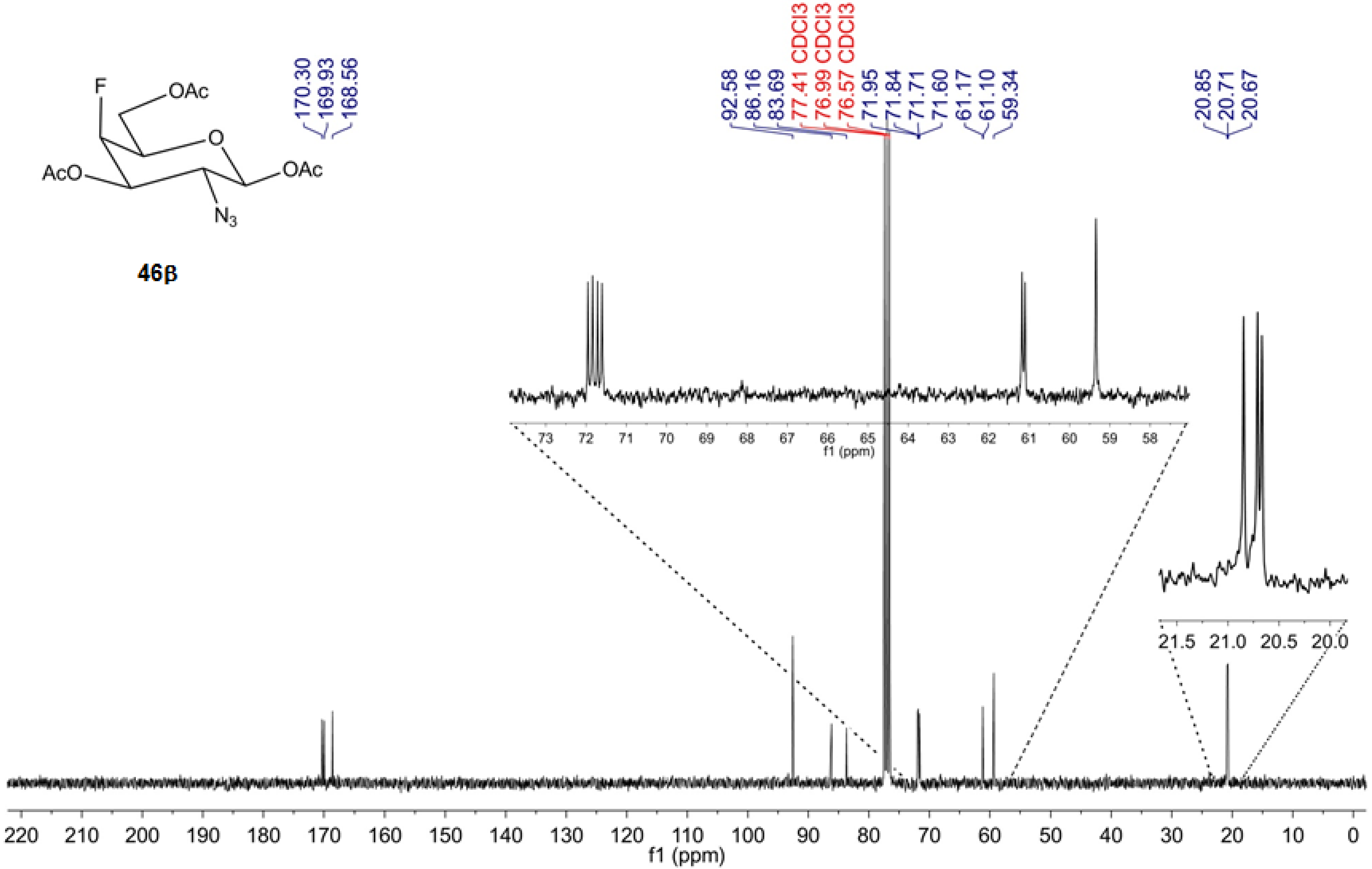

46β

$^{13}C\ \{^{1}H\}$ NMR (75 MHz, $CDCl_3$) of **46β** (β-anomer)

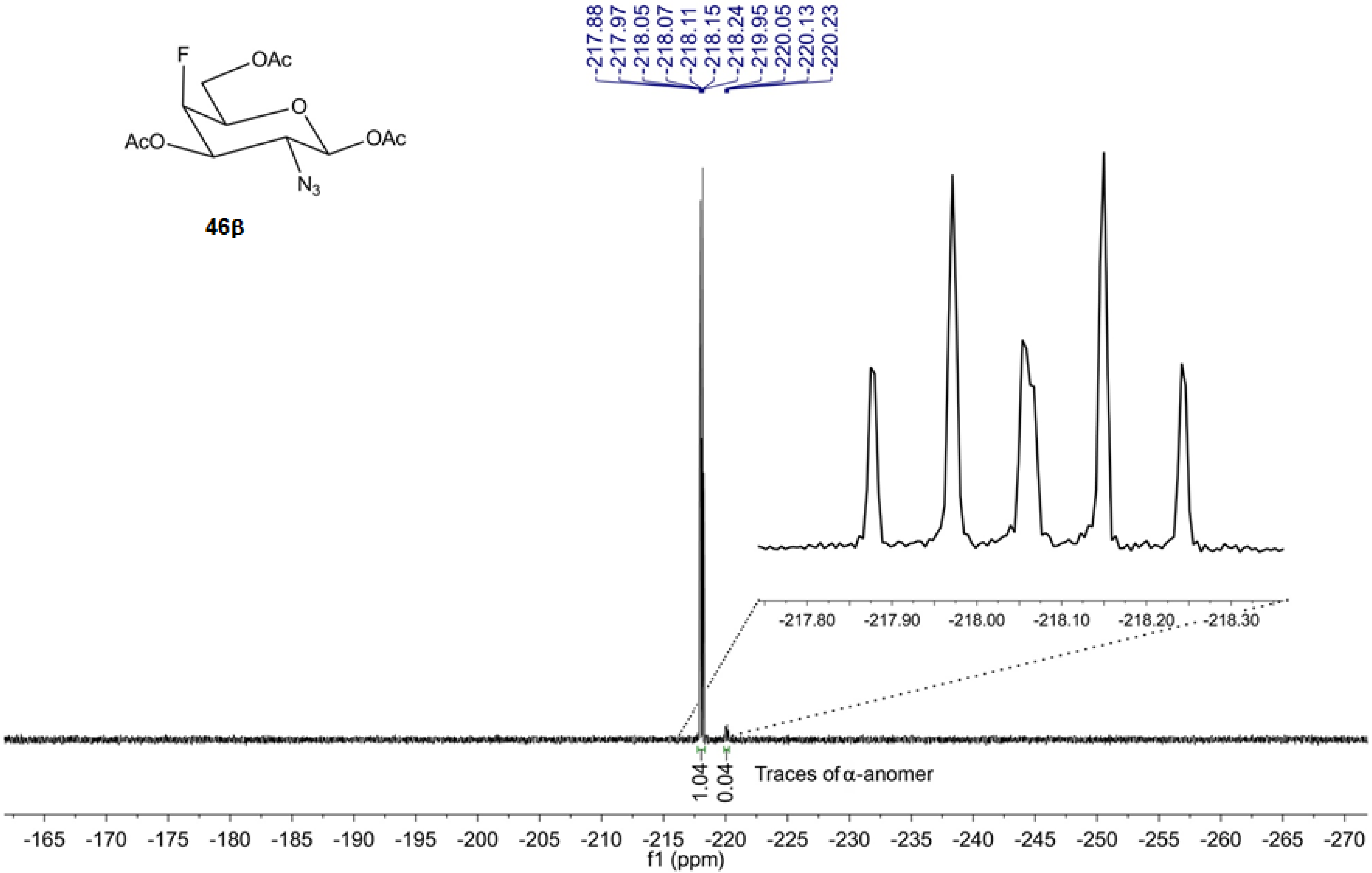

^{19}F NMR (282 MHz, $CDCl_3$) of **46** (β-anomer + ca 4% α-anomer)

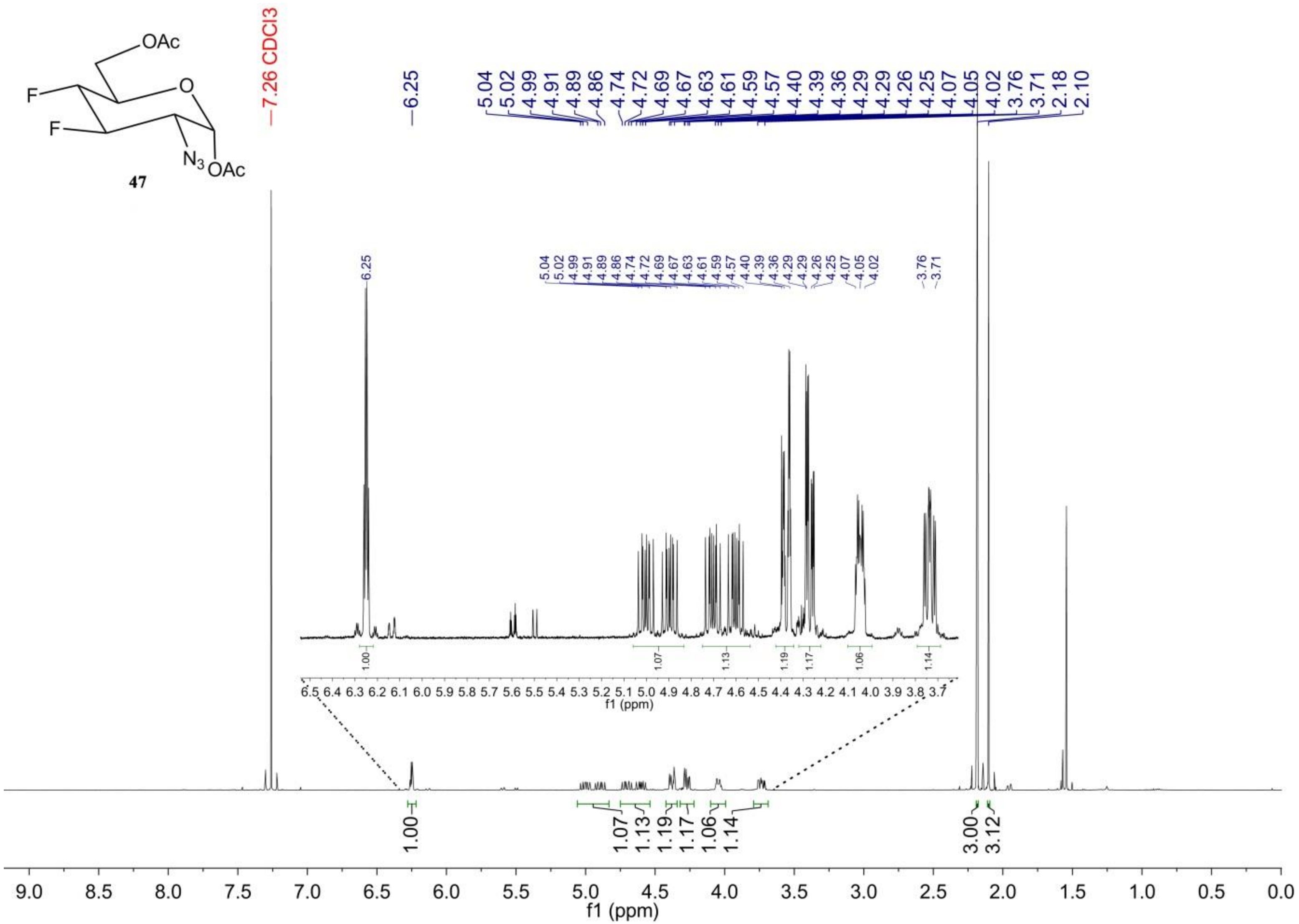

^{1}H {^{19}F} NMR (500 MHz, $CDCl_3$) of **47** (α-anomer), ca 5% impurities

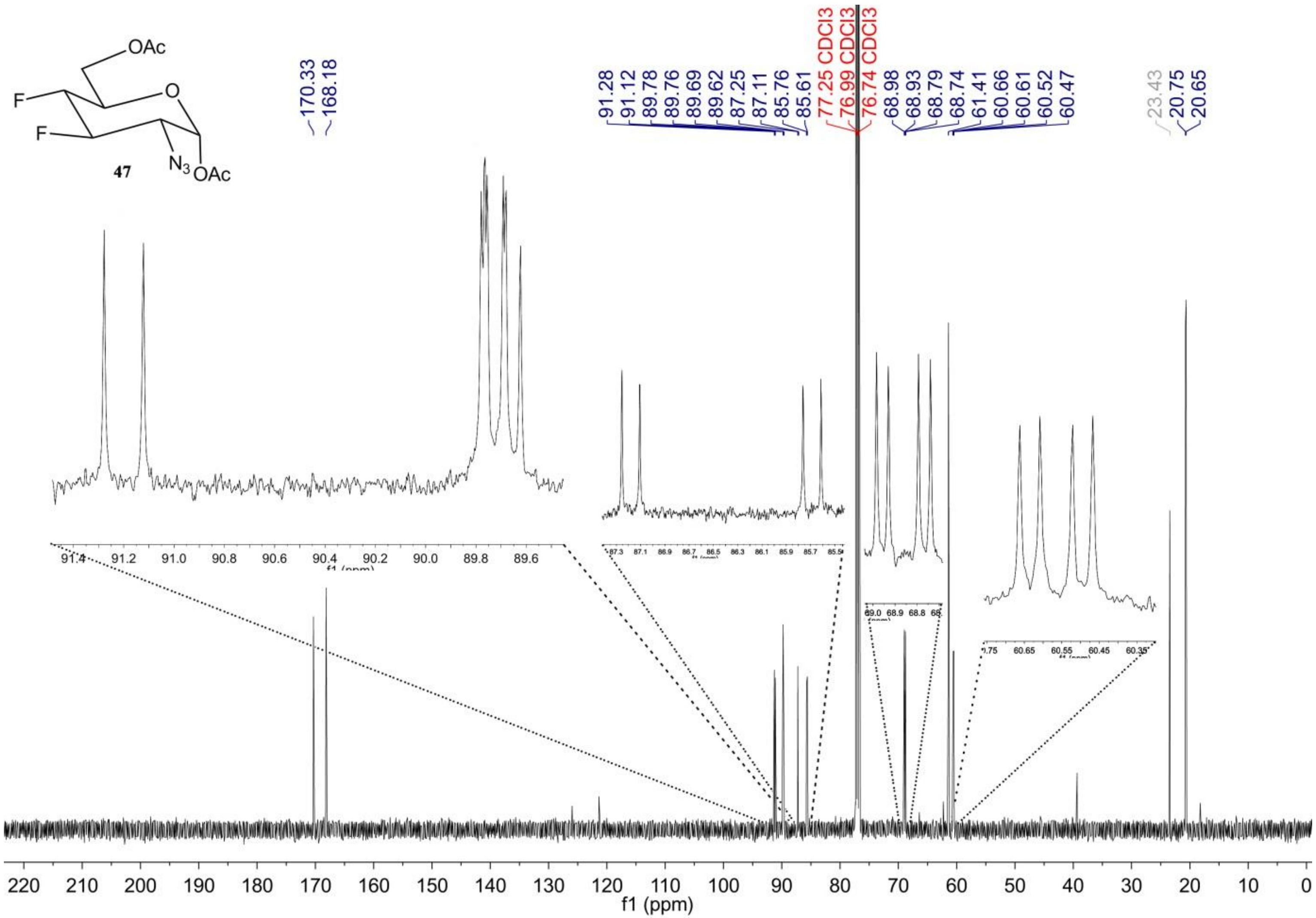

^{13}C {^{1}H} NMR (125 MHz, $CDCl_3$) of **47**. (α-anomer), ca 5% impurities

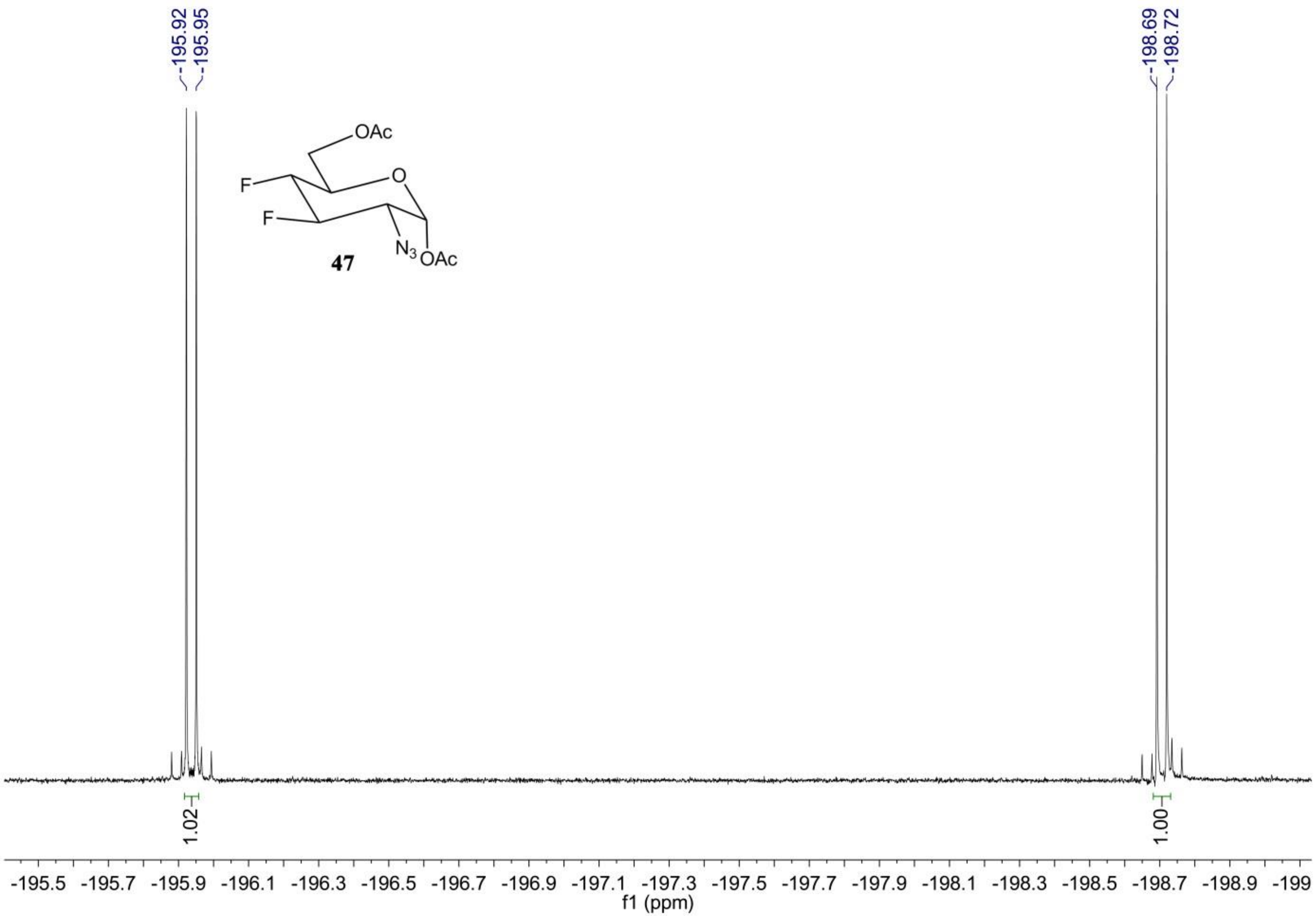

^{19}F {^{1}H} NMR (470 MHz, $CDCl_3$) of **47**. (α-anomer), ca 5% impurities

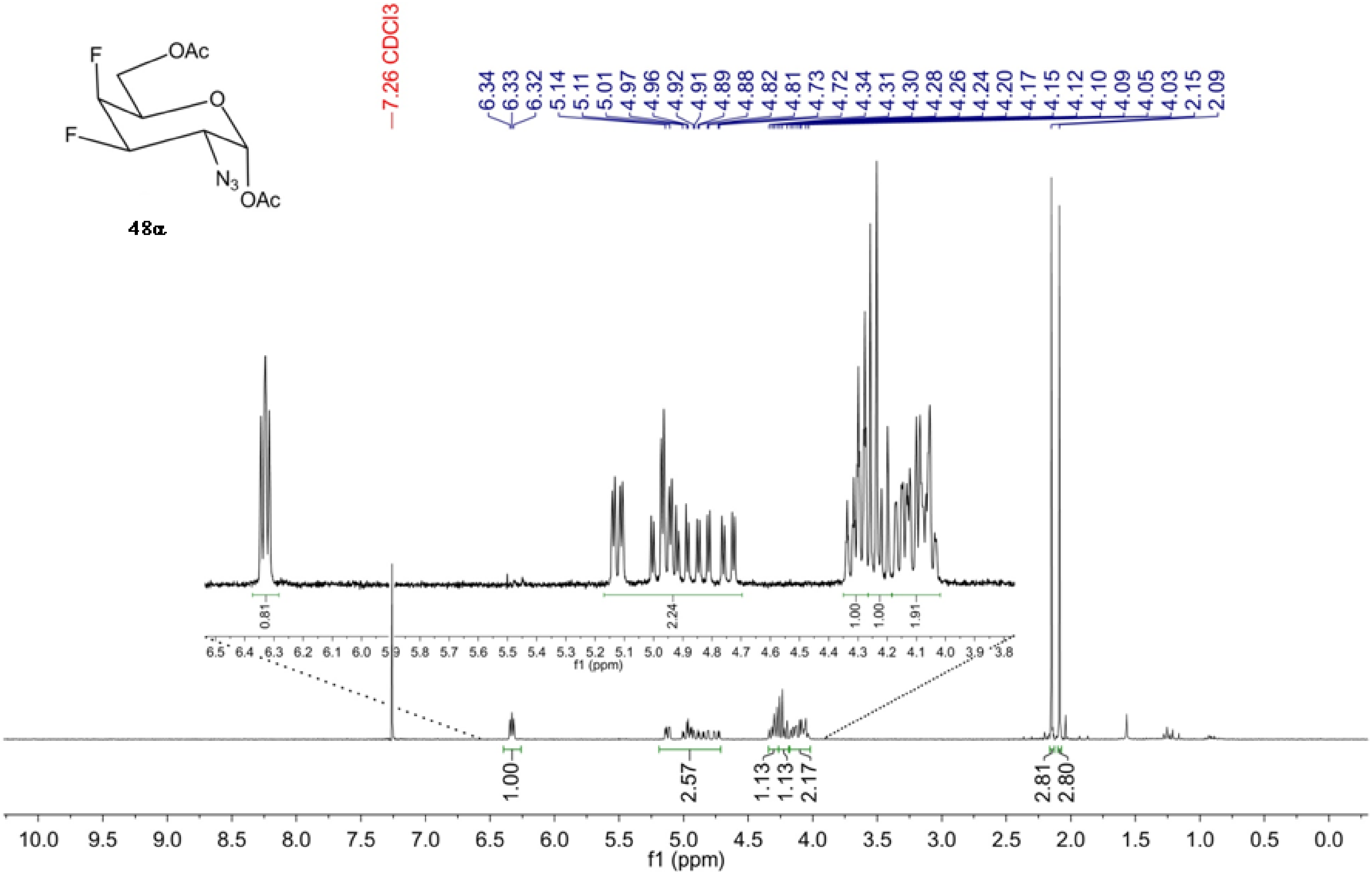

^{1}H NMR (300 MHz, $CDCl_3$) of **48** (α-anomer)

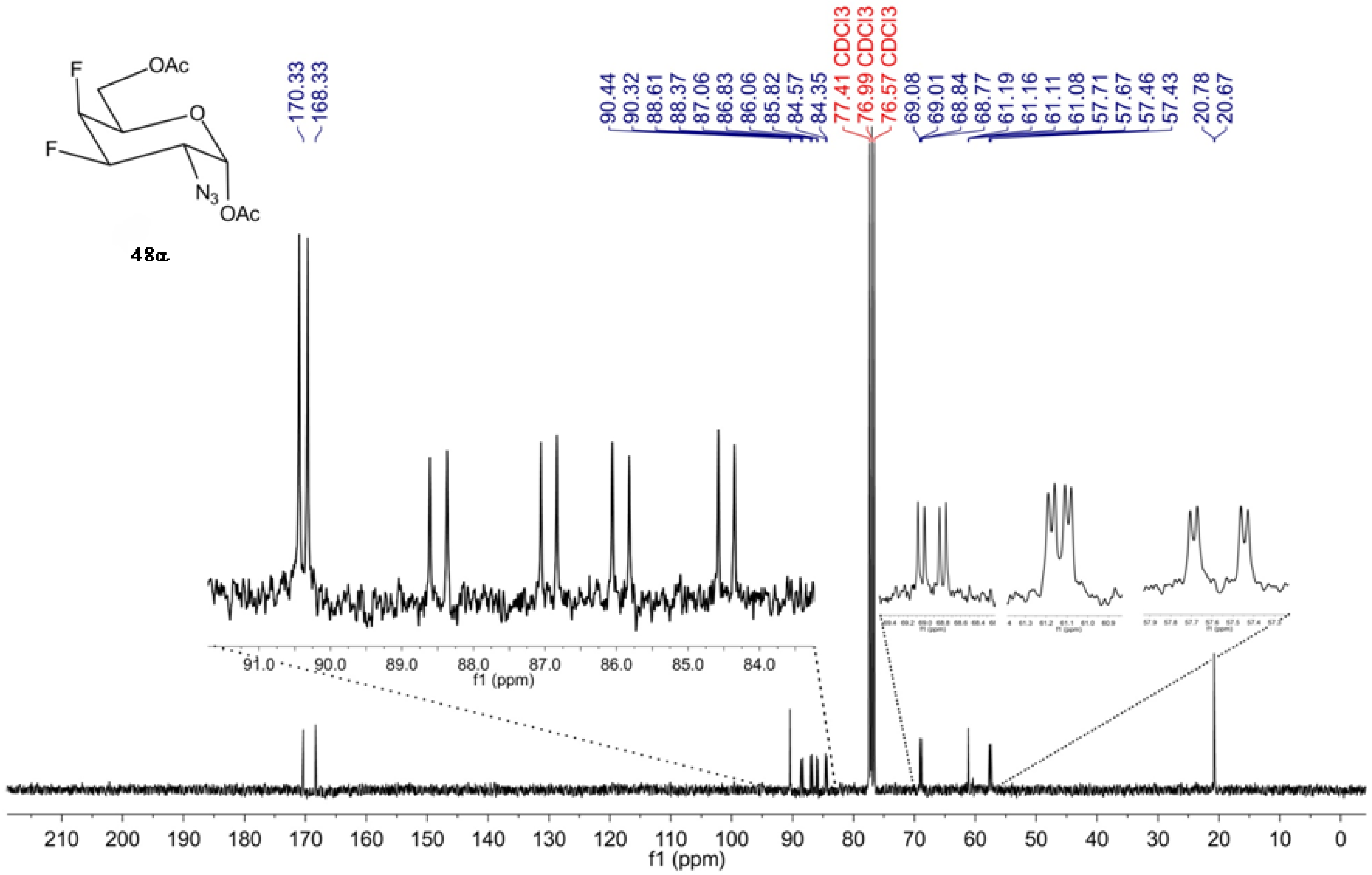

^{13}C {^1H} NMR (75 MHz, $CDCl_3$) of **48** (α-anomer)

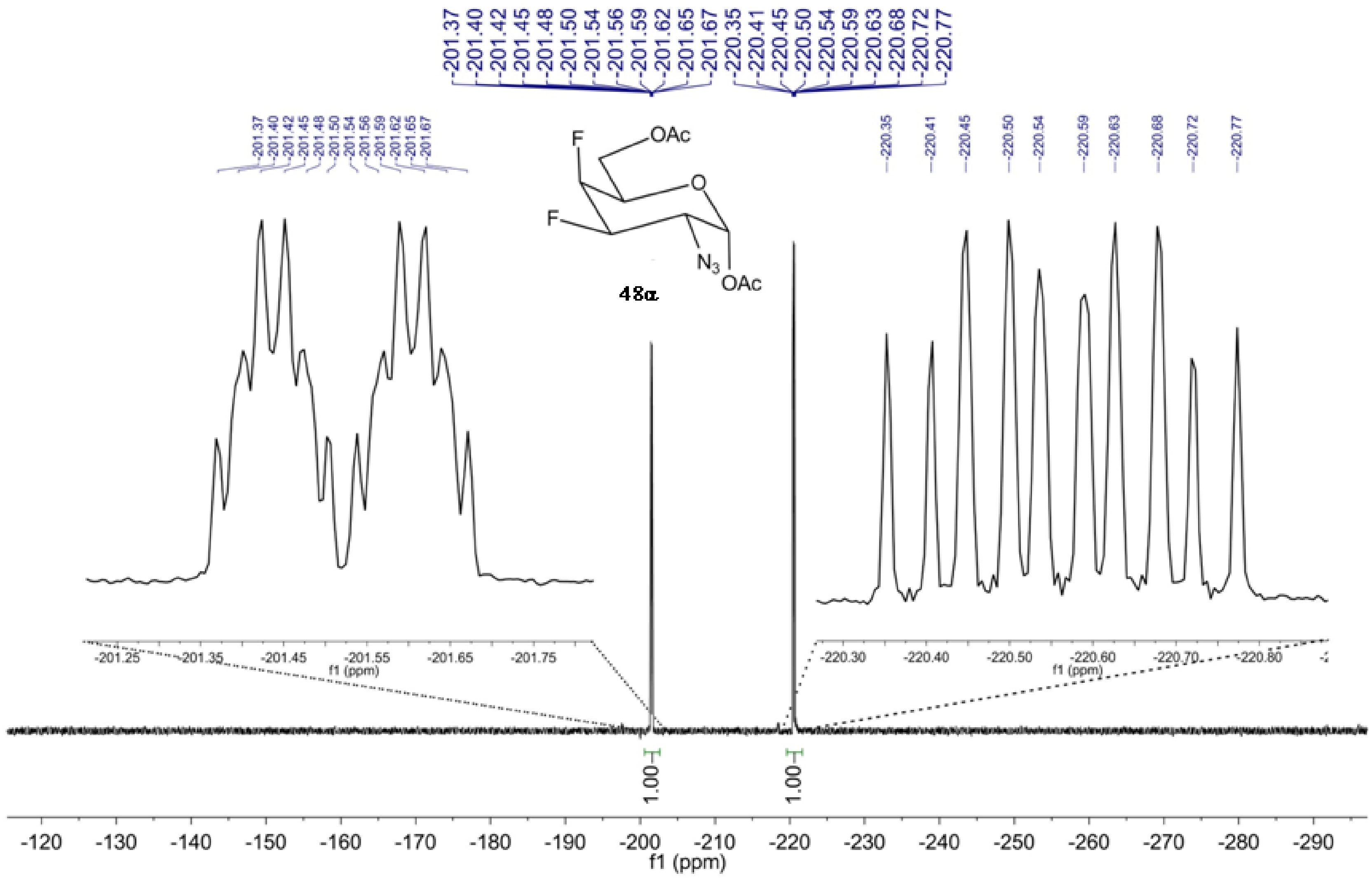

^{19}F NMR (282 MHz, $CDCl_3$) of **48** (α-anomer)

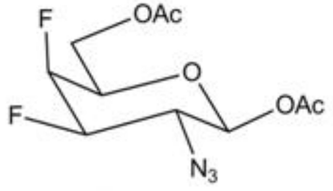
48β

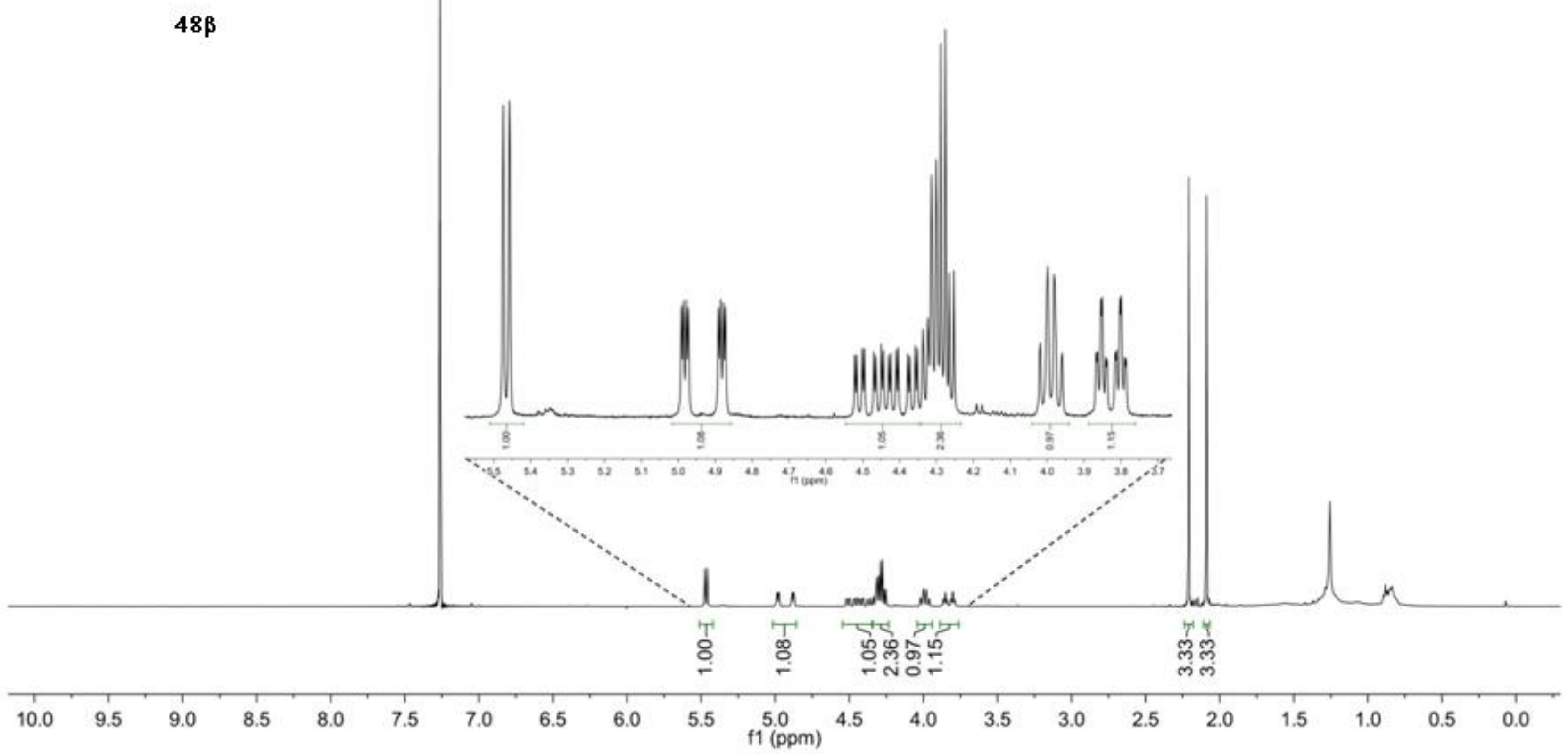

^{1}H NMR (500 MHz, $CDCl_3$) of **48** (β-anomer)

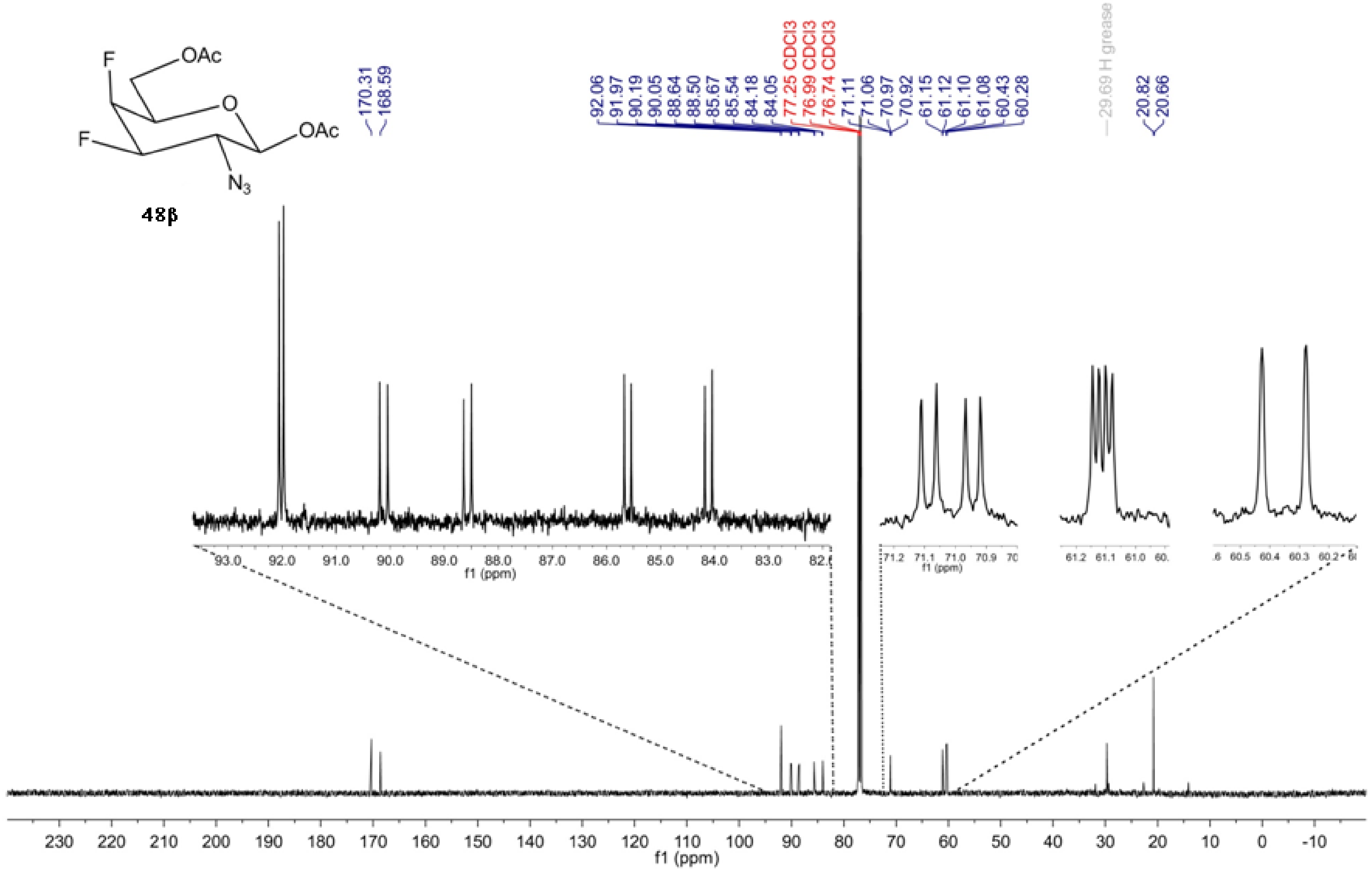

^{13}C {^{1}H} NMR (125 MHz, $CDCl_3$) of **48** (β-anomer)

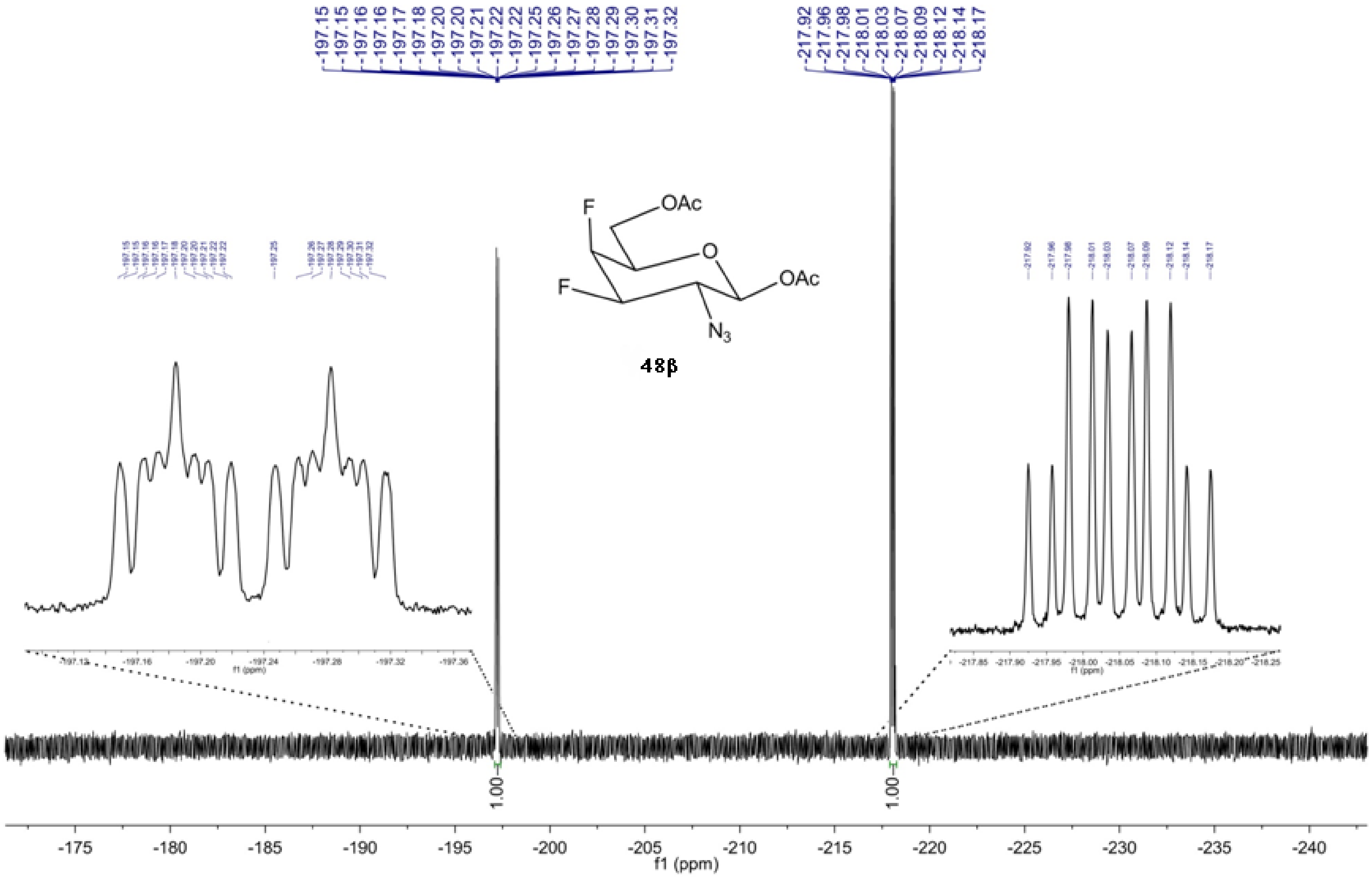

^{19}F NMR (470 MHz, $CDCl_3$) of **48** (β-anomer)

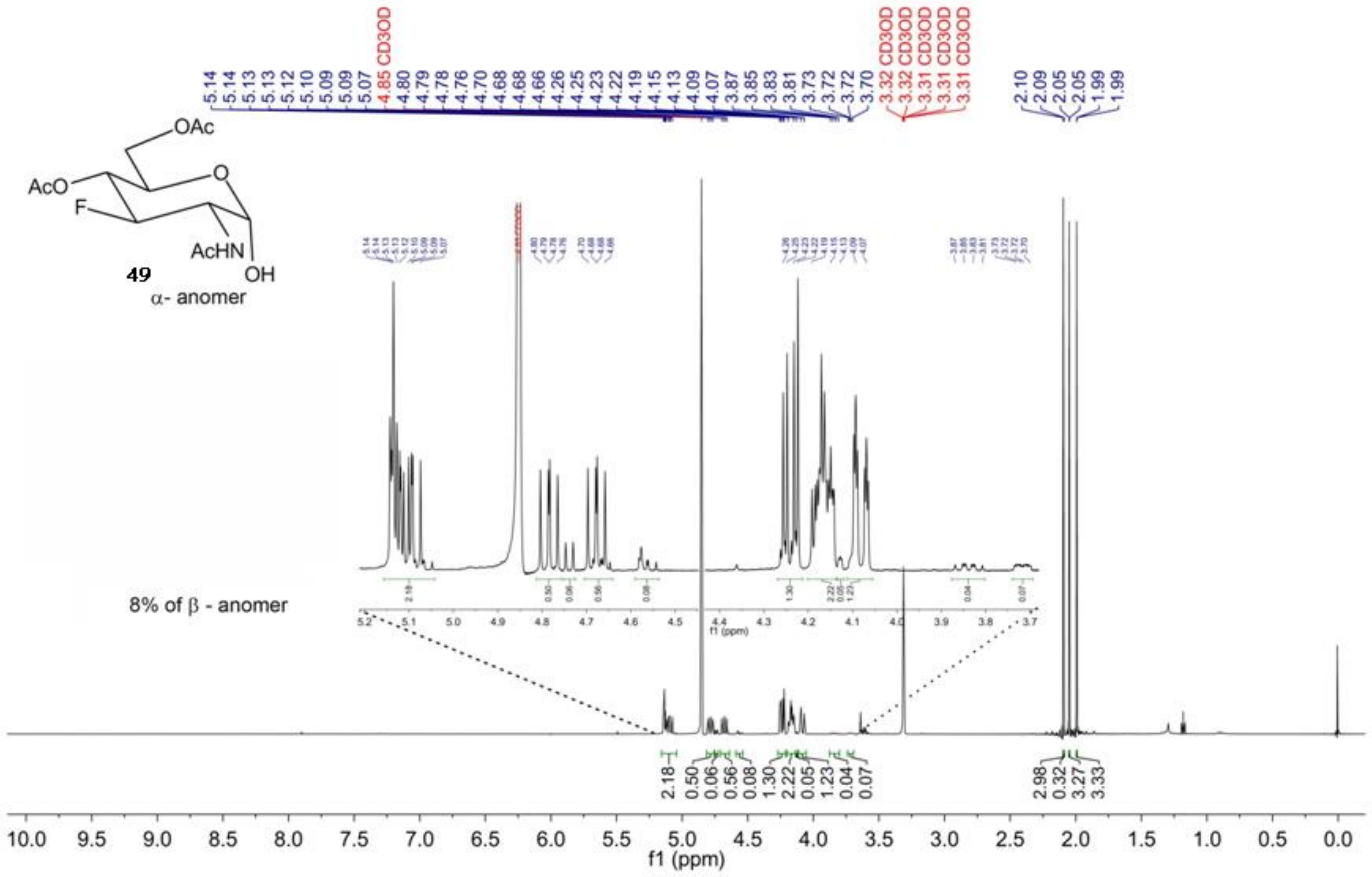

^{1}H NMR (500 MHz, CD_3OD) of **49** (α-anomer and ca 8% β-anomer)

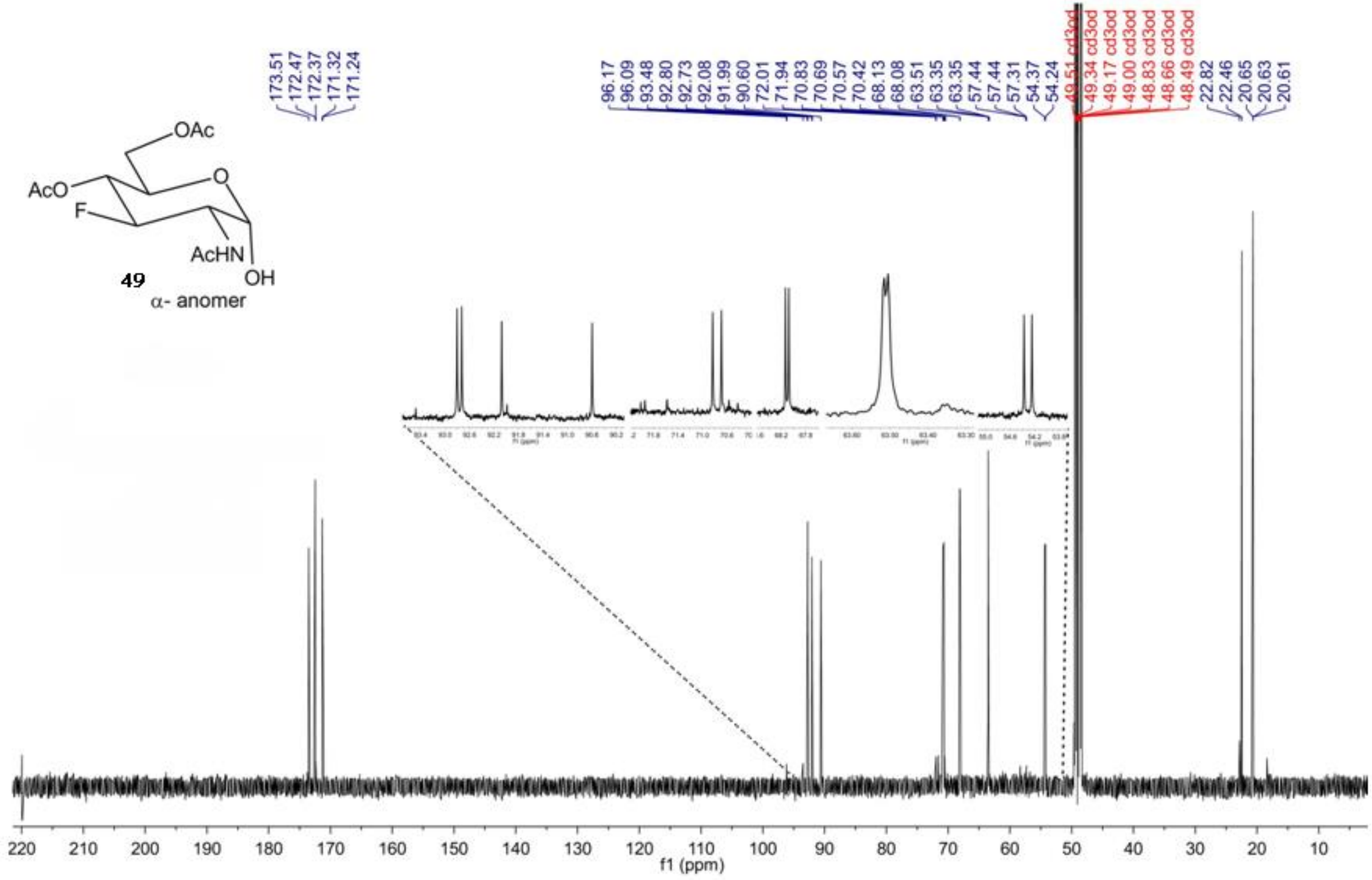

^{13}C {^{1}H} NMR (125 MHz, CD_3OD) of **49** (α-anomer and ca 8% β-anomer)

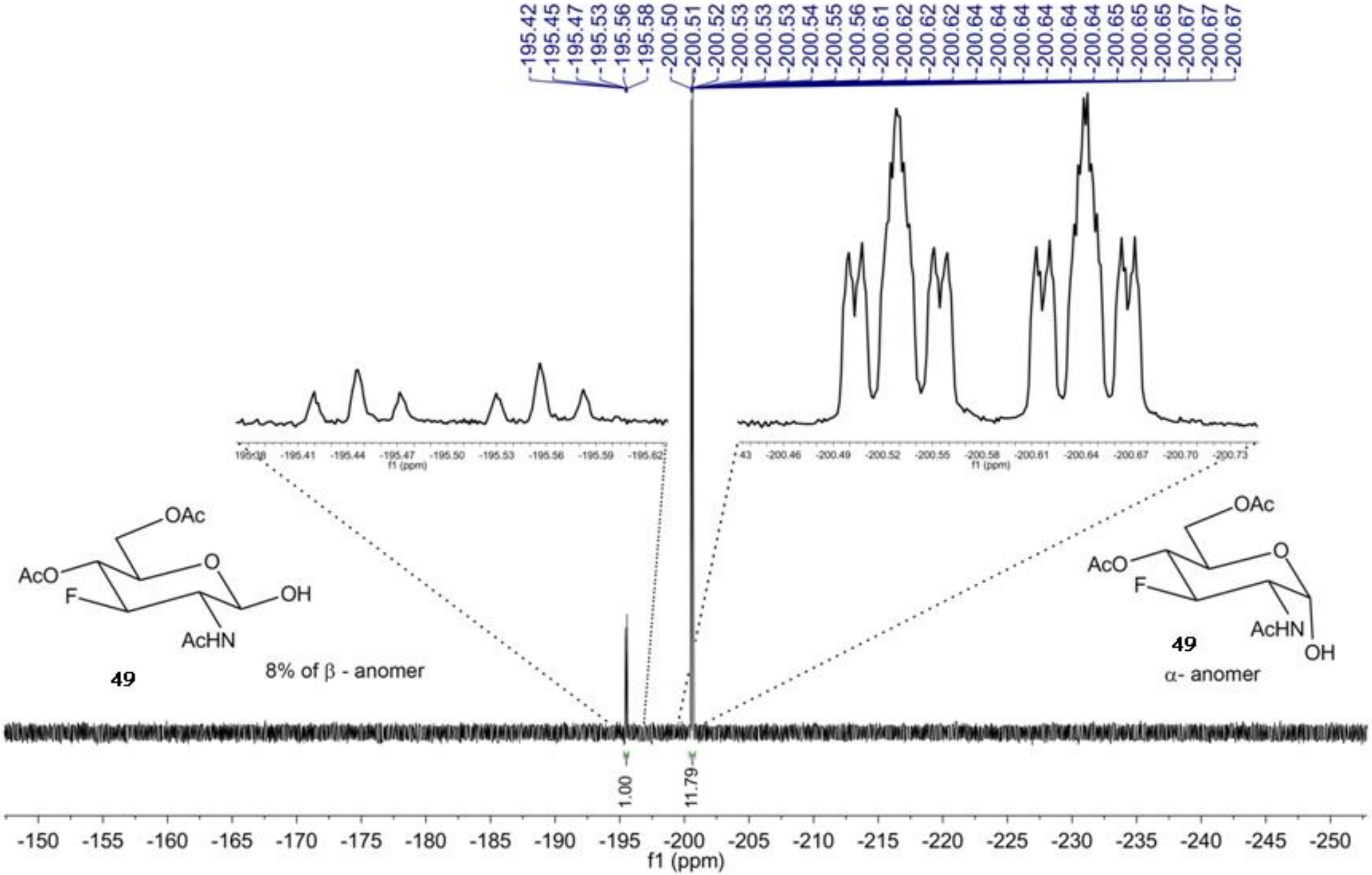

^{19}F NMR (470 MHz, CD_3OD) of **49** (α-anomer and ca 8% β-anomer)

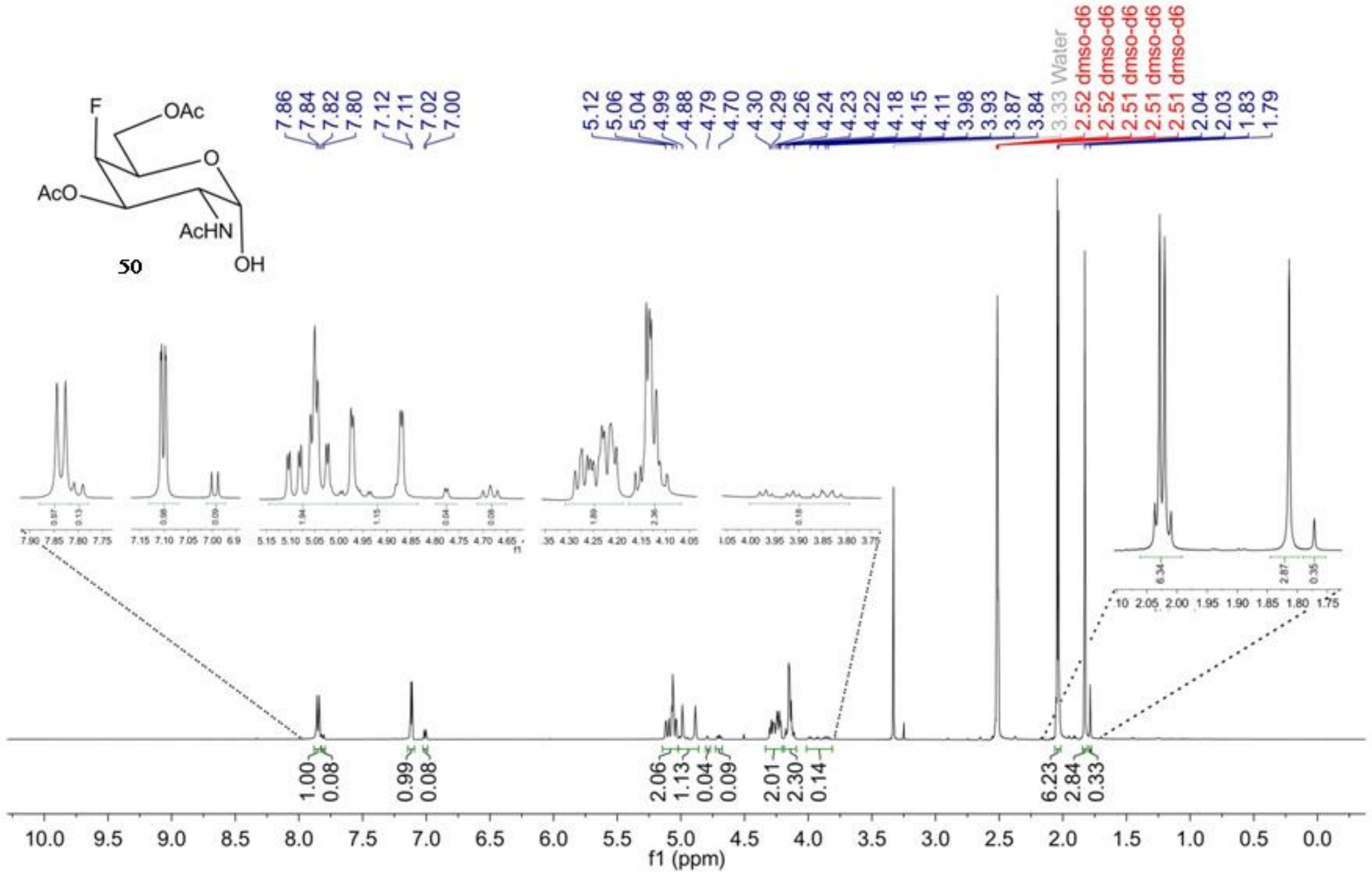

^{1}H NMR (500 MHz, $CDCl_3$) of **50** (α-anomer and ca. 10% β-anomer)

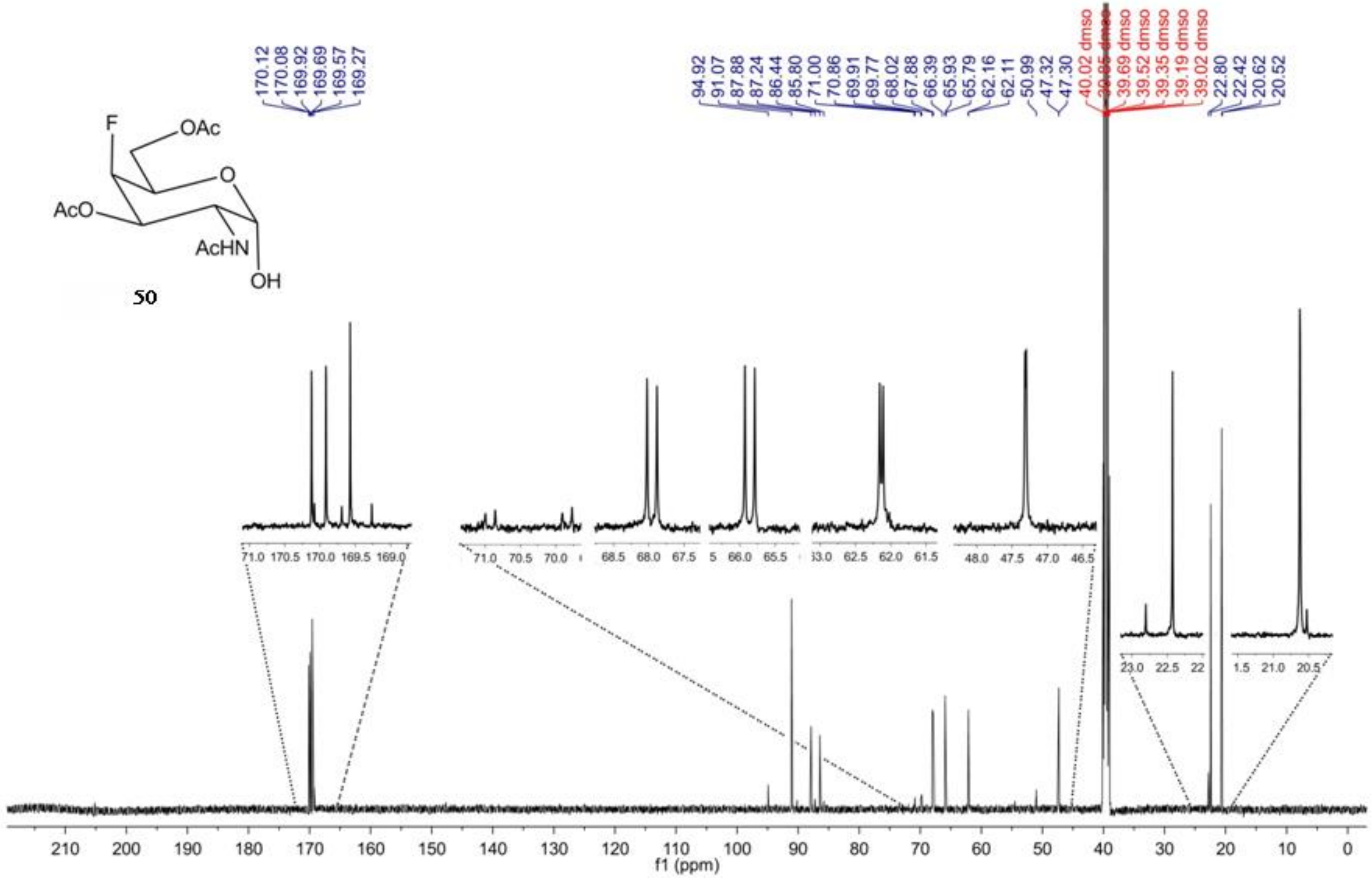

50

^{13}C {^{1}H} NMR (125 MHz, $CDCl_3$) of **50** (α-anomer and ca. 10% β-anomer)

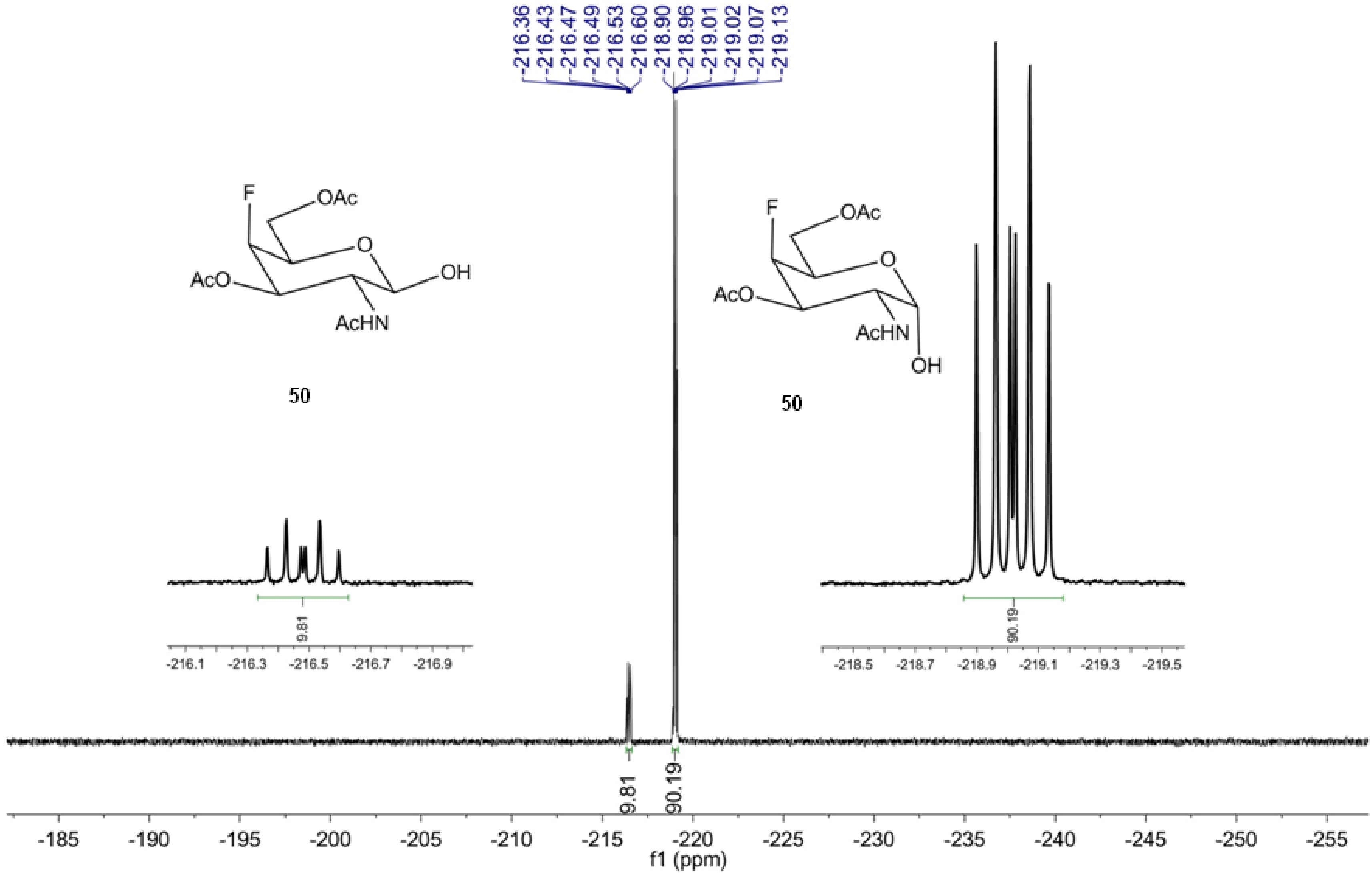

^{19}F NMR (470 MHz, $CDCl_3$) of **50** (α-anomer and ca. 10% β-anomer)

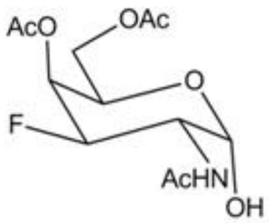

51

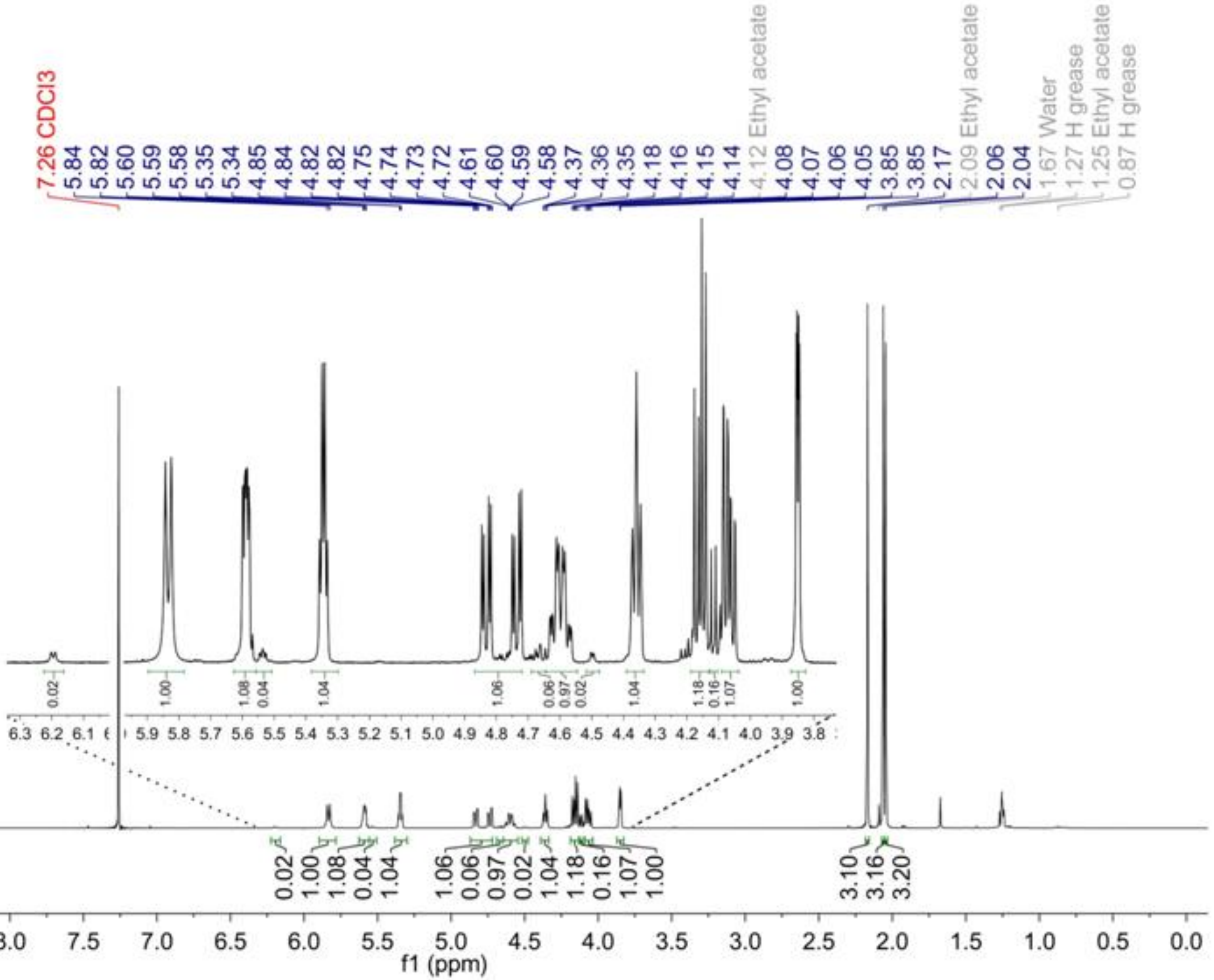

^{1}H NMR (500 MHz, $CDCl_3$) of **51** (α-anomer and ca. 5% β-anomer)

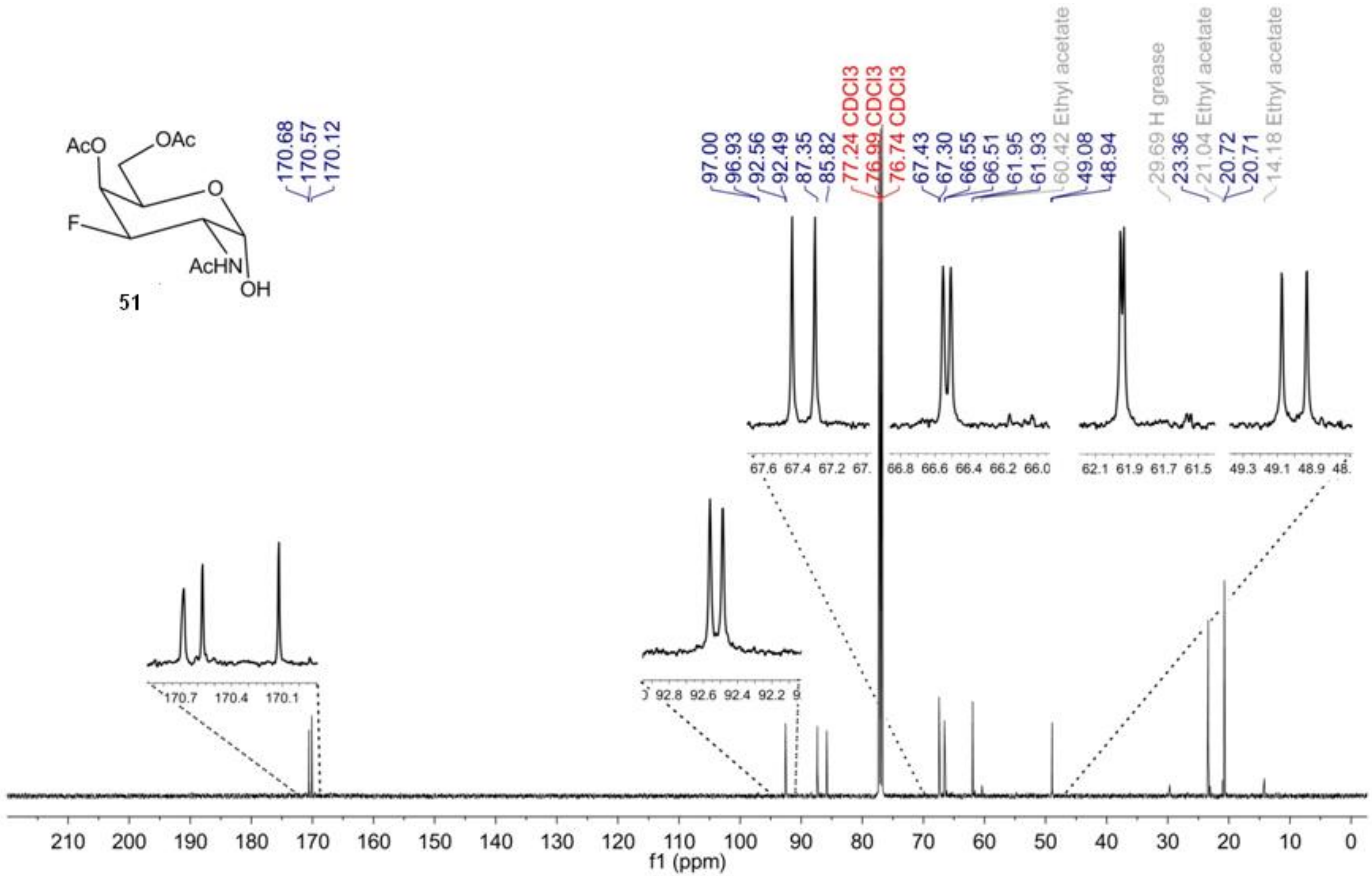

^{13}C {^{1}H} NMR (125 MHz, $CDCl_3$) of **51** (α-anomer and ca. 5% β-anomer)

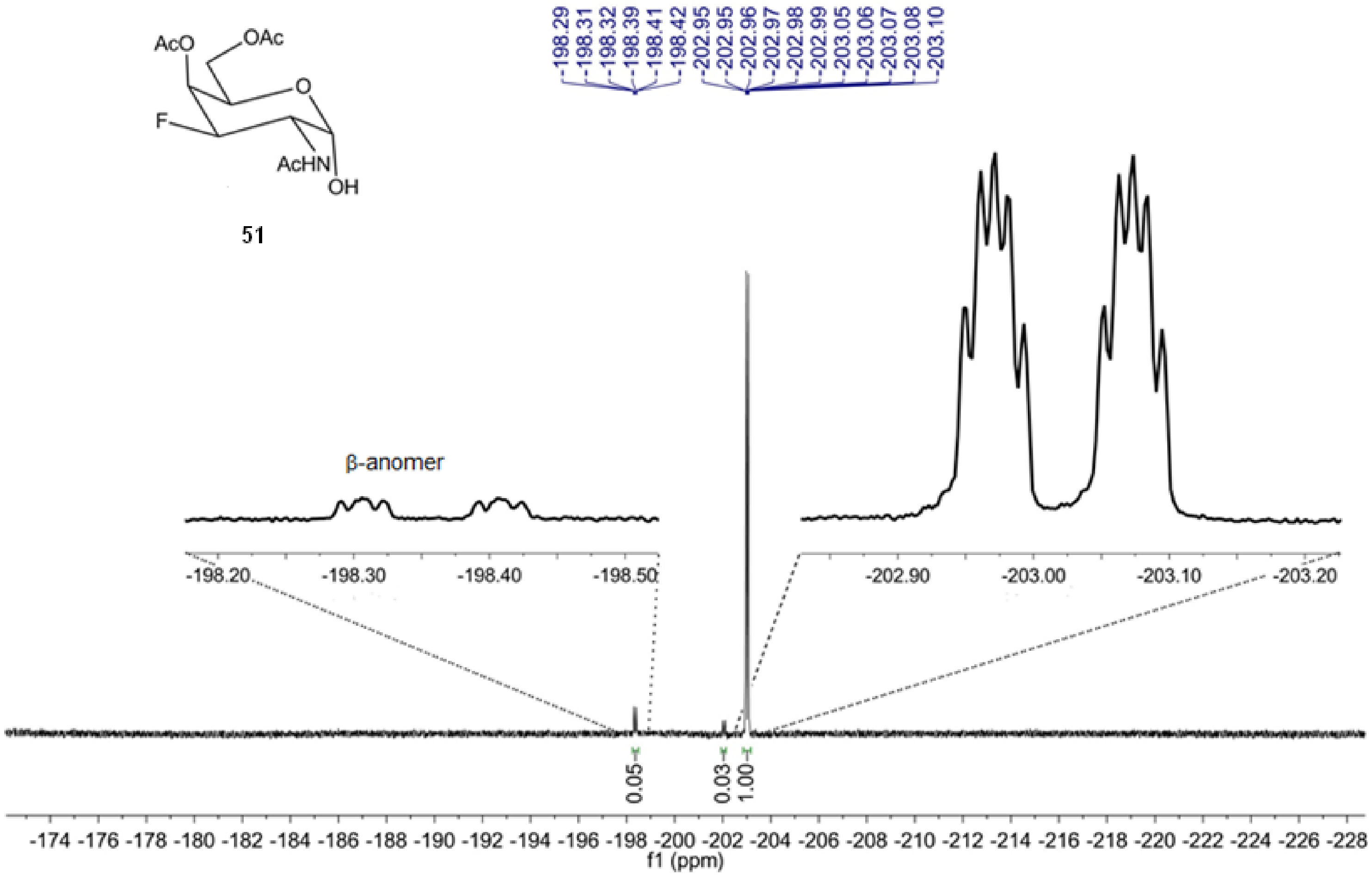

51

^{19}F NMR (470 MHz, $CDCl_3$) of **51** (α-anomer and ca. 5% β-anomer)
